# SARS-CoV-2 transmission risk for common group activities and settings: a living scoping review

**DOI:** 10.1093/eurpub/ckad195

**Published:** 2023-11-23

**Authors:** Niyati Vyas, Alexandria Bennett, Nicole Shaver, Andrew Beck, Gabriele Zitiktye, Barbara Whelan, Rhea O’Regan, Aileen Conway, Becky Skidmore, David Moher, Julian Little

**Affiliations:** Knowledge Synthesis and Application Unit, School of Epidemiology and Public Health, Faculty of Medicine, University of Ottawa, Ottawa, Ontario, Canada; Knowledge Synthesis and Application Unit, School of Epidemiology and Public Health, Faculty of Medicine, University of Ottawa, Ottawa, Ontario, Canada; Knowledge Synthesis and Application Unit, School of Epidemiology and Public Health, Faculty of Medicine, University of Ottawa, Ottawa, Ontario, Canada; Knowledge Synthesis and Application Unit, School of Epidemiology and Public Health, Faculty of Medicine, University of Ottawa, Ottawa, Ontario, Canada; Knowledge Synthesis and Application Unit, School of Epidemiology and Public Health, Faculty of Medicine, University of Ottawa, Ottawa, Ontario, Canada; Evidence Synthesis Ireland & Cochrane Ireland, School of Nursing and Midwifery, University of Galway, Galway, Ireland; Evidence Synthesis Ireland & Cochrane Ireland, School of Nursing and Midwifery, University of Galway, Galway, Ireland; Evidence Synthesis Ireland & Cochrane Ireland, School of Nursing and Midwifery, University of Galway, Galway, Ireland; Independent Information Specialist, Ottawa, Ontario, Canada; Knowledge Synthesis and Application Unit, School of Epidemiology and Public Health, Faculty of Medicine, University of Ottawa, Ottawa, Ontario, Canada; Clinical Epidemiology Program, Ottawa Hospital Research Institute, Ottawa, Ontario, Canada; Knowledge Synthesis and Application Unit, School of Epidemiology and Public Health, Faculty of Medicine, University of Ottawa, Ottawa, Ontario, Canada

## Abstract

**Background:**

While the modes of transmission of severe acute respiratory syndrome coronavirus-2 (SARS-CoV-2) are well studied, the risk of transmission in various group settings or activities is less clear. This living scoping review aims to summarize the risk factors of coronavirus disease 2019 (COVID-19) spread in common group activities (e.g. social gatherings) or settings (e.g. schools, hospitals, shared workplaces) to understand the drivers of transmission and to inform a risk assessment profile tool for use of rapid antigen detection tests.

**Methods:**

We systematically searched electronic databases, MEDLINE and Embase, from January 2019 until February 2022. We included studies that evaluated the risk of SARS-CoV-2 transmission in activities and settings, deemed strategically important to government departments in Ireland, provided by the Department of Health (Ireland) Expert Advisory Group on Rapid Testing.

**Results:**

After screening 14 052 records, data from 139 studies were narratively synthesized. The risk was consistently reported as ‘high’ for large social events (e.g. weddings) and indoor sports, working in healthcare settings and shared workplaces, working/living in residential settings and travelling via public transportation. Most studies were from healthcare settings, with common risk factors including close contact with COVID-19 cases, working in high-risk departments and inappropriate use of personal protective equipment. For other settings and activities, lack of infection prevention and control practices reportedly contributed to infection transmission.

**Conclusion:**

The heterogeneity across studies and lack of direct information on dominant variants, preventive measures, vaccination coverage necessitates further research on transmission risk within group activities to inform infection prevention and control measures.

## Introduction

The severe acute respiratory syndrome coronavirus-2 (SARS-CoV-2) emerged in China in late 2019,^1^ causing respiratory coronavirus disease 2019 (COVID-19) infection, and has since spread rapidly around the world. As of February 2023, there have been a reported 753 million confirmed cases globally and 6.8 million confirmed deaths.^2^

SARS-CoV-2 spreads from an infected individual to others through respiratory droplets produced from breathing, coughing, sneezing, singing, shouting or talking.^3^ Modes of transmission include direct contact of these infectious particles with the mucous membranes of susceptible hosts or indirect transmission via surfaces or objects infected with the virus (fomites).^3^ Reports of the outbreak investigations reveal the frequent spread of COVID-19 infection through close contact with infected individuals.^3^ Higher viral loads, a higher concentration of SARS-CoV-2 in the air, the size of virus-laden particles and higher infectious doses contribute to transmission risk between individuals.^4^ Viral transmission is also facilitated by poor ventilation and environmental conditions, although it is still unclear how easily the virus spreads in different settings.

Transmission risk has consistently been higher in some activities and settings, such as healthcare settings. This increased risk of transmission in healthcare settings was especially evident during the initial phase of the pandemic, when health systems were not adequately prepared to deal with the rapid influx of patients.^1^ While healthcare workers have been shown to be at increased risk of contracting the virus (thus exacerbating the chain of infection transmission),^1^^,^^5^ there is a lack of clarity regarding risk factors leading to COVID-19 infection as that may vary across clinical settings. Other settings known to present an increased risk of transmission include household contexts where physical distancing, mask-wearing and disinfection of shared surfaces are not feasible.^6^ A 2022 rapid review described the transmission of COVID-19 through respiratory particles in different modes of transportation and other indoor settings (e.g. courtrooms, exercise facilities and restaurants).^4^ The authors concluded that inadequate ventilation, prolonged exposure times, high viral load and attending certain activities facilitate long-range transmission. In contrast, short-range transmission is exacerbated by decreased distance from the infectious source, especially in the absence of protective measures (i.e. inadequate physical distancing and not wearing masks).^4^ However, there remains a lack of understanding surrounding transmission events and their contributing risk factors in other settings, including outdoor social settings,^7^ workplaces^7^ and educational settings.^8^

Early public health interventions focused on case isolation and contact tracing as the primary measures to contain virus spread, regardless of activity or setting. Additional measures were implemented in the subsequent stages of the pandemic, including stay-at-home orders, school closures, working from home, wearing face masks, social distancing and cross-border restrictions.^7^ Despite these measures, the number of new cases continued to climb and governments continue to face the associated social and economic harms.^7^ A better understanding of high-risk settings and risk factors for transmission within such settings could enable setting-specific public health measures. Targeted interventions with a focus on high-risk settings will be critical in future stages of the pandemic, as regular social or group activities in all areas of life resume and pandemic fatigue contributes to a lapse in personal protective measures. Furthermore, a better understanding of high-risk settings and activities will enable the prolonged COVID-19 public health response to be focused on areas most important to transmission control, especially as the increased pandemic public health resources are re-directed to other areas.

The objective of this living scoping review was to provide an overview of risk factors and risk levels of COVID-19 transmission associated with common group activities and settings, as reported by study authors. We included a ‘list of common events and activities’ deemed strategically important to government departments in Ireland provided by the Department of Health (Ireland) Expert Advisory Group on Rapid Testing, with the goal to inform a risk assessment tool for appropriate use of rapid antigen detection tests^9^ as society reopened and restrictions were relaxed.

## Methods

The review adhered to Cochrane guidance for rapid reviews and followed a pre-defined protocol publicly registered on PROSPERO (CRD42021284107) and the National Collaborating Centre for the Methods and Tools (ID#472). We used guidance from Levac and colleagues, which is an update of the Arksey and O’ Malley methodological framework and the Joanna Briggs Institute manual for scoping reviews.^10–12^

### Information sources and search strategy

The search strategy was developed by an experienced information specialist in consultation with the review team. The MEDLINE search strategy was peer-reviewed prior to execution by another senior information specialist using the PRESS Checklist (Supplementary appendix S1).^13^ Using the Ovid platform, we searched Ovid MEDLINE^®^ ALL and EMBASE Classic+Embase. The strategies utilized a combination of controlled vocabulary (e.g. ‘COVID-19’, ‘Disease Transmission, Infectious’, ‘Cluster Analysis’) and key words (e.g. ‘novel CoV’, ‘transmission’, ‘superspread’). There were no language restrictions, but results were limited to the publication years 2019 to the present and, where possible, animal-only and opinion pieces were removed. The following grey literature sources were also searched: Cochrane Covid-19, COVID-END, L-OVE, UNCOVER, ClinicalTrials.gov—Covid-19 resources and the WHO Covid-19 Database. The initial search was conducted on 29 September 2021, followed by periodic updates until February 2022. The complete search strategies are available in Supplementary appendix S2.

### Eligibility criteria

A complete summary of the eligibility criteria defined by the participants, concept and context framework is available in Supplementary appendix S3. Eligible study designs were contact tracing reports and observational studies (e.g. case-control studies, cohort studies). Individuals of any age with a laboratory-confirmed diagnosis of COVID-19, using RT-PCR, were eligible for inclusion. We included studies examining the risk of COVID-19 transmission associated with group activities (e.g. social gatherings, gyms) or settings (e.g. schools, hospitals, households) and what factors contribute to risk. A complete list of important activities provided from the Department of Health (Ireland) Expert Advisory Group on Rapid Testing is available in Supplementary appendix S4. Studies published in languages other than English were excluded at the screening stage.

### Study selection

All reviewers independently conducted a pilot training exercise based on 50 articles for the title and abstract screening and ten articles for the full-text review before beginning the study selection. A single reviewer independently screened each title and abstract for inclusion. A single reviewer completed the full-text review, with a second reviewer verifying inclusion. Any disagreements were resolved through discussion or third-reviewer consultation.

### Data charting

Before initiating data extraction, all reviewers independently conducted a training exercise on five articles. One reviewer independently extracted data from each included study, and a second reviewer verified it. Any disagreements observed were resolved through discussion or third-reviewer consultation. We extracted data on the study and participants’ characteristics, including sample size, study design, location and dates of study conduct. To identify the risk of COVID-19 transmission, we extracted data on the probable source, settings or activities, or the risk factors contributing to the transmission risk. Lastly, we collected data on the risk level associated with various activities and any COVID-19 preventive measures reported. The risk level was extracted as reported by study authors (high, medium or low) and we also extracted any details on how the authors assessed risk. In cases where the authors did not clearly label the risk level, we classified the reported risk level as ‘unclear’.

### Synthesis of included studies

We conducted a narrative synthesis and presented a visual summary of evidence from different settings and activities from all the included studies, with their associated levels of risk (i.e. high, low, moderate, unclear) based on study authors’ reporting. Activities/settings were noted to present a high risk of SARS-CoV-2 transmission if all included studies reported a high risk, low risk if all studies reported a low risk, unclear risk if the study authors failed to report the risk level or an inconsistent risk if the reported risk levels were inconsistent between included studies.

### Protocol deviations

Our protocol had outlined the conduct of a living rapid review. However, given the large number of studies included, we determined that the shift to a living scoping review was warranted, and therefore no risk of bias assessments were carried out. Additionally, the list of eligible settings/activities was amended in November 2021 to include going to a place of worship, movie theatre/cinema, cafe, children’s indoor play centre, outdoor playground, visiting a hospital and being a spectator at an indoor/outdoor match. These amendments were made after the initial searches were completed due to changing priorities from the Department of Health (Ireland) Expert Advisory Group on Rapid Testing, meaning that some studies addressing these settings may have been excluded. Thus, this scoping review is not intended to be an exhaustive synthesis of all studies addressing risk in these settings but to synthesize overall patterns from the literature.

## Results

**Figure 1:**
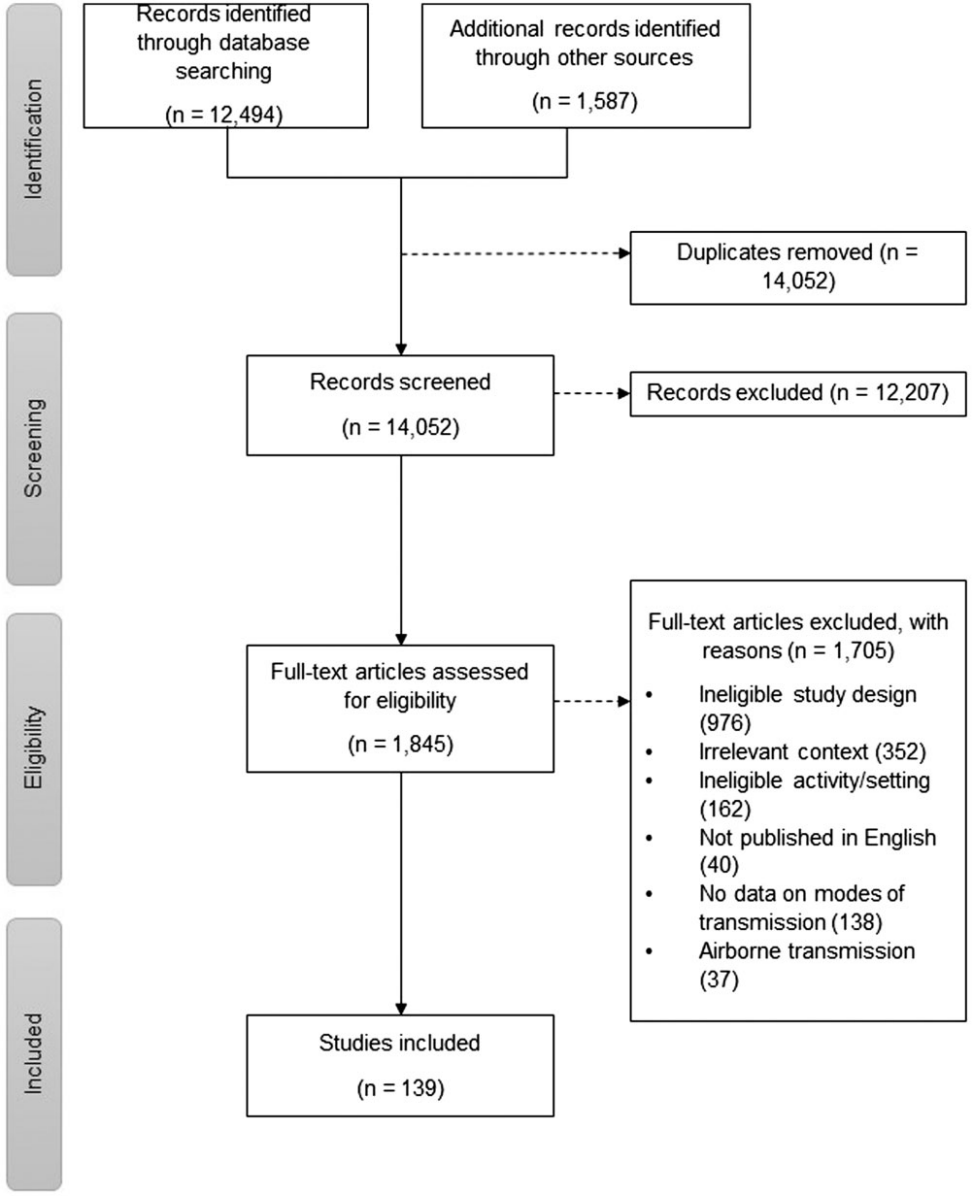
PRISMA flowchart.^14^

From the initial searches and search updates, a total of 14 081 studies were retrieved. Following de-duplication, 14 052 references were reviewed. We excluded 12 236 studies during the title and abstract screening and 1845 references during the full-text review. A total of 139 studies was included.^14–152^ The PRISMA 2020 flow chart diagram (figure 1) shows the details of the screening process. A summary of included study characteristics is presented in Supplementary appendix S5. A complete list of excluded studies at the full-text stage with the reason for exclusion is available in Supplementary appendix S6.

Included studies were conducted between 2020 and 2021. The individual countries with the largest number of reports were the USA (*n* = 26), China (*n* = 15), Germany (*n* = 11) and the UK (*n* = 9). The remaining studies were from other Western European and Asia Pacific countries. Most of the included study designs were observational (*n* = 100) in conjunction with some surveillance (*n* = 22) and contact tracing studies (*n* = 12).

### Risk factors contributing to SARS-CoV-2 transmission

**Figure 2:**
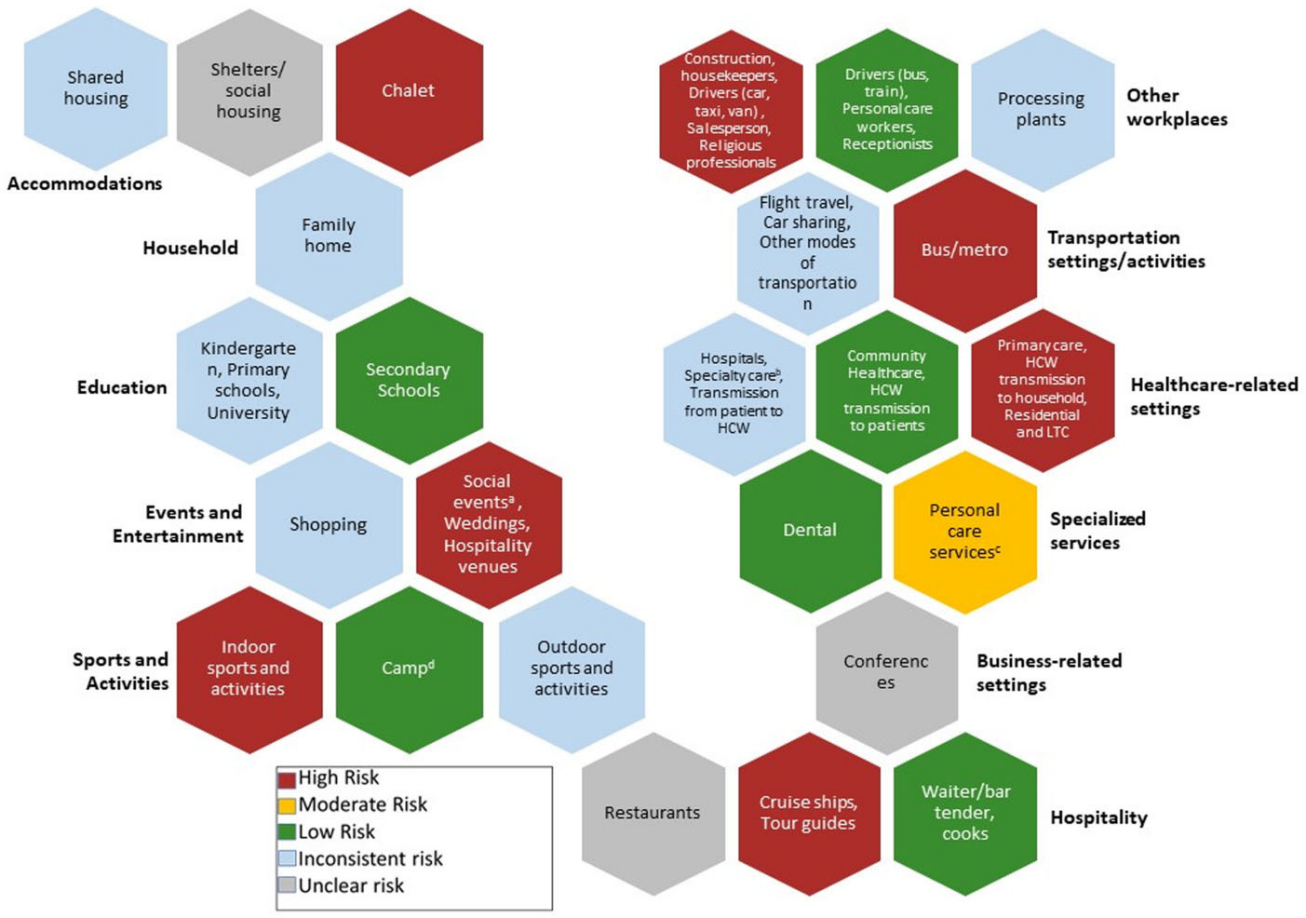
Visual summary of evidence for transmission risk of COVID-19 and different settings and activities. ^a^Social events are defined by the study authors and may include but is not limited to any social activity with one or more individuals such as dating, getting together with a neighbour or friends, banquet, dinner, karaoke, community gatherings or birthday parties. ^b^Speciality care includes specific settings outside a general hospital setting (e.g. dialysis unit, physical therapy, outpatient care). ^c^Personal services may include hair salons, beauty parlours, nail salons, spa, etc. ^d^Includes indoor/outdoor and summer camps. HCW, health care workers; LCT, long-term care.

A visual summary of evidence for the transmission risk of SARS-CoV-2 through different activities and settings is presented in figure 2 (see Supplementary appendix S7 for table summary). Overall, 17 activities were labelled as ‘high risk’, 10 activities were labelled as ‘low risk’, and one activity (personal care services) was judged to present a ‘moderate’ risk, as judged by study authors. Two activities were judged to present an ‘unclear’ risk, as the risk level was not judged by study authors and 14 activities were judged to be at inconsistent risk, as the reported risk level varied between included studies.

For household settings, we identified 17 studies evaluating risk factors for SARS-CoV-2 transmission, of which eight studies reported that this setting was associated with high-risk levels as reported by study authors and where close contact with the suspected or diagnosed COVID-19 household patients and large household size were the common factors.^14–21^ In studies assessing SARS-CoV-2 transmission risk in types of accommodation other than households, authors reported high levels of transmission risk for those living in the same chalet,^22^ whereas transmission risk for other forms of shared housing^14,23–26^ was considered to be highly variable across studies.

In a single data-linkage study of SARS-CoV-2 transmission risk in business conferences, the authors reported high levels of risk, despite the implementation of social distancing.^27^ Studies in hospitality settings reported high levels of infection transmission risk in cruise ships^28^ and restaurants,^16,29,30^ whereas working in professions such as cooking^31^ or waiting^31^ were reported to have low or unclear levels of risk. In a single study, authors judged occupations such as driving (e.g. bus, train), personal care and reception to have low risk levels of transmission.^31^ For specialized services [i.e. dental^32^ and other personal care (e.g. beauty parlour and gymnasium)^29^] the risk of transmission was reported to be low^32^ and medium.^29,32^ In nine studies relating to modes of public transportation, there was high-risk of SARS-CoV-2 transmission due to close contact, long exposure to suspected or confirmed COVID-19 cases and return from the high-risk endemic areas as the probable sources of transmission.^16–18,33–38^ For other occupations and workplaces (i.e. construction labour, domestic housekeepers, processing plants, salesperson, religious professionals) study authors reported that most have a higher risk level, despite missing information on the potential sources of infection transmission.^31,38–43^

With regard to educational settings (i.e. kindergarten, primary, and secondary schools), authors from 25 studies reported SARS-CoV-2 transmission risk to be unclear, moderate or low.^15,21,44–68^ For kindergarten and primary schools, the reported levels of risk were either unclear or low, considering already existing interventions such as hand hygiene, hybrid education, masks and social distancing. Likewise, studies of risk within secondary schools showed low risk, while also reporting putatively preventive measures such as face masks, distancing, hybrid education and improved ventilation. A single study reported high levels of transmission risk in university settings, identifying close-contact off-campus being a probable risk factor.^69^

For settings related to events and entertainment, transmission risk was consistently reported to be high by study authors, including attendance at various social events,^15,26,29,39,70–78^ weddings,^27,79^ hospitality venues^27^ and shopping (e.g. convenience stores or shops).^16,26,33,39^ For sports and activities, four studies judged indoor sports to present a high transmission risk and reported close contact while playing with the suspected or confirmed COVID-19 cases as a probable transmission source.^60,80–82^ On the contrary, outdoor sports and activities were reported to present a low or unclear transmission risk.

We included many studies reporting on SARS-CoV-2 transmission risk in different healthcare settings. Study authors across 39 studies reported increased infection/transmission risk in primary care, hospitals, and residential/long-term care settings, despite various infection preventive measures in place.^14,17–20,27,78,83–113,153^ The most probable risk factor for transmission within these studies was close contact or prolonged exposure with the suspected/confirmed COVID-19 patient. Considering health care workers (HCWs) specifically, 28 studies^17–20,83–91,93–100,102–105,109,112,113^ showed high transmission risk with the common risk factors reported as close contact/direct care of COVID-19 suspected/confirmed patients, inappropriate use of personal protective equipment (PPE), long duty hours and carrying out aerosol-generating procedures. In the patient population of the healthcare settings, we observed that there were not enough studies assessing the transmission risk except six studies.^78,92,106–109^ Transmission from HCWs to their household was consistently reported as a high-risk activity,^98–101,117^ whereas transmission from HCWs to patients was reported as ‘low’ in all studies.^129,133^ On the contrary, studies assessing SARS-CoV-2 transmission in specialty care settings showed medium^29^ or low-risk levels.^32^

To explain some of the heterogeneity between studies in transmission risks, we examined reported risk across countries. 25/40 studies from the Asia-Pacific countries,^14^^,^^16^^,27–29,35–37,39,43,70,75,77,79,85–87,89,90,97,100,111,113,115,140^ 20/53 studies from Europe^14^^,^^18,19,22,33,76,78,81,91,92,94–96,101,102,104,105,114,139^^,^^153^ and 16/37 studies from Americas^17^^,34,42,69,72,80,83,84,88,103,106–108,110,115,145^ reported higher transmission risk levels for the spread of SARS-CoV-2; however, with considerable heterogeneity across settings and reported risk factors.

## Discussion

The purpose of this scoping review was to synthesize evidence on risk factors contributing to SARS-CoV-2 transmission in various group-based settings and activities. We observed considerable variation in the reported level of transmission risk between the large number of included studies.

The largest number of included studies addressed SARS-CoV-2 transmission risk in healthcare settings. The most common risk factors for transmission risk within this setting, as noted by the study authors, were close/direct contact with the suspected/confirmed COVID-19 case, working in high-risk departments, carrying out COVID-19 identification procedures (e.g. aerosol generation), inappropriate use of PPE and use of public transport. These results aligned with a 2021 systematic review showing increased infection risk among healthcare workers compared to those living in the general community, with the greatest risk being among those working in in-patient settings and nursing homes.^5^ The review also reported that a lack of adequate PPE (i.e. N95 masks, reused/inadequate PPE) or suboptimal hand hygiene practices contributed to an increased COVID-19 infection risk. Another living systematic review highlighted the importance of PPE use and found a higher risk of SARS-CoV-2 infection in healthcare workers, especially those with inadequate PPE use.^154^ We observed similar trends in transmission risk factors in our narrative synthesis, despite variation in the overall level of reported transmission risk between clinical settings. The level of transmission risk within specific clinical settings is important for targeted infection prevention and control measures and should be explored in future research.

Our review found inconsistency in the reported level of reported SARS-CoV-2 transmission risk for educational environments, which varied by setting. Risk was judged to be low or unclear for kindergartens and primary schools, with generally low risk reported for secondary schools and high-risk levels for the university settings. Similarly, a 2022 living rapid review on the specific roles of daycare and schools in COVID-19 transmission reported low transmission risk in primary care and daycare in its recent update, especially where Infection Prevention and Control measures are in place.^155^ The rapid review authors also reported high variability in transmission activities within the secondary schools, based on minimal data.

The risk of transmission varied for modes of transportation, with the highest risk reported for taking the bus or metro. For air travel, the risk ranged from low to high, with several studies reporting an unclear risk of transmission. A 2021 systematic review on COVID-19 transmission in aircraft reported low transmission risk; however, there were no conclusive assessments considering the low quality of evidence.^156^

In household settings, the risk of infection was reported to be high or unclear. Risk factors like close contact with confirmed/suspected COVID-19 cases had a higher level of transmission risk, regardless of infection preventive measures in place. These findings align with a 2021 systematic review on SARS-CoV-2 transmission in household settings, where study authors estimated 18.9% of an overall household secondary attack rate, demonstrating households a significant infection transmission site.^6^

For the identified studies assessing SARS-CoV-2 transmission risk in settings related to events and entertainment, the risk was generally reported to be high, with close contact with the suspected/confirmed COVID-19 patient as the potential reason behind an increased transmission. For workplaces, the reported level of risk varied by the specific type of workplace, since the workplaces represented a variety of sectors across different studies. The information on the probable source of transmission within workplaces was generally missing, which demands further research to better understand what factors contribute to workplace transmission. Lastly, with the other settings such hospitality, accommodation other than households, and other specialty services, we had limited studies capturing information on a potential source of infection transmission along with heterogeneity in the reported risk levels. There is a need for further research in these areas to understand infection transmission risks.

Our review had several limitations. The assessment of risk of SARS-CoV-2 transmission for settings/activities within this review was extracted as it was reported by individual study authors, meaning that the assessment of risk was not standardized across studies. There was also likely heterogeneity in the measurement and assessment of transmission risk due to various study designs, sample size and transmission mitigation strategies. As this study was scoping in nature, we did not perform quality appraisal of the included studies to assess potential methodological limitations. In addition, included studies lacked direct information on the dominant SARS-CoV-2 variants at the time the studies were conducted, COVID-19 preventive measures and vaccination coverage among the population captured. Future research should explore how these factors affect SARS-CoV-2 transmission risk. Lastly, evidence of SARS-CoV-2 transmission risk in different settings/activities is continually emerging. Due to the rapid influx of COVID-19 literature and the amended list of eligible activities due to additional interest in certain activities/settings from the Department of Health (Ireland) Expert Advisory Group on Rapid Testing, studies addressing eligible settings may have been missed. Thus, this scoping review was not intended to be an exhaustive synthesis of all studies addressing risk in eligible settings/activities, but to synthesize overall patterns of transmission risk from the many studies that did meet the eligibility criteria. Future research should continue to explore changes in SARS-CoV transmission risk as the pandemic continues.

## Supplementary Material

ckad195_Supplementary_Data

## Data Availability

No new data were generated or analysed in support of this research.

## Registration

National Collaborating Centre for Methods and Tools https://www.nccmt.ca/covid-19/covid-19-evidence-reviews/472.

OSF (Open Science Framework) | COVID-19 rapid antigen testing and transmission risk associated with different activities or settings: Two living rapid reviews (https://osf.io/pqjyr/).

PROSPERO CRD42021284107 (https://www.crd.york.ac.uk/prospero/display_record.php?RecordID=284107).

Key points

- The risk of SARS-CoV-2 transmission was consistently reported by study authors to be ‘high’ for social events (e.g. weddings), indoor sports, working in healthcare and shared workplaces, working/living in residential settings and travelling via public transportation.
- Close contact/prolonged exposure with COVID-19 cases, working in high-risk departments and inappropriate PPE use were reported as the common risk factors for SARS-CoV-2 transmission in these settings.
- Research gaps included a lack of direct information from studies on the dominant SARS-CoV-2 variants at the time the studies were conducted, COVID-19 preventive measures and vaccination coverage among the population captured.
- Additional research on SARS-CoV-2 transmission risk within group activities is needed to inform infection prevention and control measures.
