# Supplementary material for: SARS-CoV-2 transmission risk for common group activities and settings: a living scoping review
Source: Eur J Public Health. 2023 Nov 23;34(1):196–201. doi: 10.1093/eurpub/ckad195 (PMC10843946; doi:10.1093/eurpub/ckad195)
Supplement: ckad195_Supplementary_Data [file ckad195_supplementary_data.zip › ckad195_Supplementary_Data/ejph-2023-07-om-0370-File004.docx]

# Appendix 1. PRESS Peer Review Guideline

## *PRESS Guideline* 2015— Search Submission & Peer Review Assessment

Reference: McGowan J, Sampson M, Salzwedel DM, Cogo E, Foerster V, Lefebvre C. PRESS Peer Review of Electronic Search Strategies: 2015 guideline statement. *J Clin Epidemiol* 2016;75:40-6. Available: <http://www.jclinepi.com/article/S0895-4356(16)00058-5/pdf>.

**Search submission: This section to be filled in by the searcher**

Searcher: Becky Skidmore Email: [bskidmore@rogers.com](mailto:bskidmore@rogers.com)

Date submitted:27 Sep 2021 Date requested by: 29 Sep 2021 PM

| 1. **Systematic Review Title** |  |
| --- | --- |

Activities or settings associated with a higher risk of SARS-CoV-2 transmission

| 1. **This search strategy is …** |
| --- |

| X | My PRIMARY (core) database strategy — First time submitting a strategy for search question and database |
| --- | --- |
|  | My PRIMARY (core) strategy — Follow-up review NOT the first time submitting a strategy for search question and database. If this is a response to peer review, itemize the changes made to the review suggestions |
|  | SECONDARY search strategy— First time submitting a strategy for search question and database |
|  | SECONDARY search strategy — NOT the first time submitting a strategy for search question and database. If  this is a response to peer review, itemize the changes made to the review suggestions |

| 1. **Database** (e.g., MEDLINE, CINAHL) *[mandatory]* |
| --- |

MEDLINE

| 1. **Interface** (e.g., Ovid, EbscoHost…) *[mandatory]* |
| --- |

Ovid

| 1. **Research Question** (Describe the purpose of the search)  *[mandatory]* |
| --- |

**KQ2**: What is the risk of COVID-19 transmission associated with different activities (e.g., dining, exercising etc.) or settings (e.g., educational, hospitality etc.) and what factors contribute to risk (e.g., type of contact, number of contacts, time within the risk environment)?

| 1. **PICO Format** Outline the PICOs for your question — i.e., Patient, Intervention, Comparison, Outcome, and Study Design — as applicable |
| --- |

| **P** | Individuals of any age with a laboratory-confirmed diagnosis of COVID-19, using RT-PCR. |
| --- | --- |
| **I / Exposure** | - any setting (including household, health and social care, and workplace settings) - any activity. |
| **C** | Individuals of any age, tested with RT-PCR, in whom SARS-CoV-2 ribonucleic acid (RNA) was not detected.  Control group must be matched by important covariates (for example, age and sex) to the case group. |
| **O** | - Number of cases and contacts, per activity or setting. - Number of clusters‡, outbreaks† or super-spreading events¥, per activity or setting. - Risk of infection with SARS-CoV-2, per activity or setting. |
| **S** | Include:  ♣ Evidence syntheses of outbreaks, clusters or super-spreading events (including syntheses of media reports).  ♣ National or regional retrospective contact tracing studies  ♣ Observational studies with a comparator group that aim to estimate the risk of transmission in various settings (for example, case-control studies, cohort studies).  ♣ Prospective contact tracing studies that use data from cases and non-cases to estimate the risk of onward transmission in different settings or due to different activities. |

| 1. **Inclusion Criteria** (List criteria such as age groups, study designs, etc., to be included) *[optional]*   **This search strategy is …** |
| --- |

| 1. **Exclusion Criteria** (List criteria such as study designs, date limits, etc., to be excluded) **[optional]** |
| --- |

| 1. **Was a search filter applied?** No |
| --- |

**If YES, which one(s) (e.g., Cochrane RCT filter, PubMed Clinical Queries filter)? Provide the source if this is a published filter.** *[mandatory if YES to previous question* — *textbox]*

| 1. **Notes or comments you feel would be useful for the peer reviewer**  *[optional]* |
| --- |

This is a pseudo update (not directly as the search has been adjusted significantly) of the Nov 2020 review: <https://www.hiqa.ie/sites/default/files/2020-11/Evidence-summary-activities-and-settings-at-higher-risk.pdf>

Line 68 – not trying to be exhaustive but attempting to itemize known “hot spots”

| 1. **Please copy and paste your search strategy here, exactly as run, including the number of hits per line. [mandatory]** |
| --- |

Database: Ovid MEDLINE(R) ALL <1946 to September 23, 2021>

Search Strategy:

--------------------------------------------------------------------------------

1 COVID-19/ (107361)

2 SARS-CoV-2/ (83585)

3 Coronavirus/ (4792)

4 Betacoronavirus/ (33243)

5 Coronavirus Infections/ (45123)

6 (COVID-19 or COVID19).tw,kf. (158316)

7 ((coronavirus* or corona virus*) and (hubei or wuhan or beijing or shanghai)).tw,kf. (5305)

8 (wuhan adj5 virus*).tw,kf. (254)

9 (2019-nCoV or 19nCoV or 2019nCoV).tw,kf. (1833)

10 (nCoV or n-CoV or "CoV 2" or CoV2).tw,kf. (61138)

11 (SARS-CoV-2 or SARS-CoV2 or SARSCoV-2 or SARSCoV2 or SARS2 or SARS-2 or severe acute respiratory syndrome coronavirus 2).tw,kf. (62088)

12 (2019-novel CoV or Sars-coronavirus2 or Sars-coronavirus-2 or SARS-like coronavirus* or ((novel or new or nouveau) adj2 (CoV or nCoV or covid or coronavirus* or corona virus or Pandemi*2)) or (coronavirus* and pneumonia)).tw,kf. (20274)

13 (novel coronavirus* or novel corona virus* or novel CoV).tw,kf. (10311)

14 ((coronavirus* or corona virus*) adj2 "2019").tw,kf. (36216)

15 ((coronavirus* or corona virus*) adj2 "19").tw,kf. (5818)

16 ("coronavirus 2" or "corona virus 2").tw,kf. (19240)

17 (OC43 or NL63 or 229E or HKU1 or HCoV* or Sars-coronavirus*).tw,kf. (3853)

18 COVID-19.rx,px,ox. or severe acute respiratory syndrome coronavirus 2.os. (5251)

19 (coronavirus* or corona virus*).ti. (23640)

20 COVID.ti. (124340)

21 ("B.1.1.7" or "B.1.351" or "B.1.617" or "B.1.427" or "B.1.429").tw,kf,rx,px,ox. (748)

22 ("P.1" and (Brazil* or variant?)).tw,kf,rx,px,ox. (1592)

23 (((alpha or beta or delta or eta or gamma or iota or kappa or lambda) adj3 variant?) and (coronavirus* or corona virus* or covid*)).tw,kf. (220)

24 or/1-23 [COVID-19] (193665)

25 COVID-19/tm [Transmission] (3786)

26 Coronavirus Infections/tm [Transmission] (4706)

27 exp Disease Transmission, Infectious/ (75801)

28 (transmit* or transmissi* or infectiousness* or infectivit*).tw,kf. (584538)

29 (communit* adj3 spread*).tw,kf. (1024)

30 ((COVID-19 or COVID19) adj5 (caus* or pass or passed or passes or passing or spread*)).tw,kf. (17533)

31 ((coronavirus* or corona virus*) adj5 (caus* or pass or passed or passes or passing or spread*)).tw,kf. (9007)

32 ((2019-nCoV or 19nCoV or 2019nCoV) adj5 (caus* or pass or passed or passes or passing or spread*)).tw,kf. (197)

33 ((nCoV or n-CoV or "CoV 2" or CoV2) adj5 (caus* or pass or passed or passes or passing or spread*)).tw,kf. (8300)

34 ((SARS-CoV-2 or SARS-CoV2 or SARSCoV-2 or SARSCoV2 or SARS2 or SARS-2) adj5 (caus* or pass or passed or passes or passing or spread*)).tw,kf. (8605)

35 ((virus* or infection*) adj5 (caus* or pass or passed or passes or passing or spread*)).tw,kf. (170132)

36 or/25-35 [TRANSMISSION] (792322)

37 24 and 36 [COVID-19 DISEASE TRANSMISSION] (47466)

38 cluster analysis/ (65439)

39 (cluster* adj3 (analys* or analyz*)).tw,kf. (43524)

40 (cluster* not (randomi#ed or controlled trial? or RCT or RCTs or clinical trial?)).ti. (64237)

41 (cluster* adj3 (disease? or infecti* or virus*)).tw,kf. (5242)

42 (cluster* adj3 source?).tw,kf. (731)

43 (cluster* adj3 (COVID-19 or COVID19 or coronavirus* or corona virus* or 2019-nCoV or 19nCoV or 2019nCoV or nCoV or n-CoV or "CoV 2" or CoV2 or SARS-CoV-2 or SARS-CoV2 or SARSCoV-2 or SARSCoV2 or SARS2 or SARS-2)).tw,kf. (536)

44 case cluster*.tw,kf. (334)

45 Carrier State/ (22214)

46 ((carry* or carrier?) adj2 (infect* or state?)).tw,kf. (6119)

47 (superspread* or super-spread*).tw,kf. (581)

48 over* dispers*.tw,kf. (738)

49 (transmi* adj5 (event? or hotspot? or hot spot?)).tw,kf. (2846)

50 (transmi* adj5 (chain? or cascade? or cluster?)).tw,kf. (3650)

51 (transmi* adj3 (risk* or setting?)).tw,kf. (20724)

52 Asymptomatic Infections/ep [epidemiology] (997)

53 Risk Factors/ and tm.fs. (19505)

54 Risk Factors/ and exp Disease Transmission, Infectious/ (5448)

55 Risk Factors/ and transmi*.tw,kf. (24136)

56 Risk Factors/ and exp Leisure Activities/ (13456)

57 Environmental Exposure/ and tm.fs. (653)

58 Environmental Exposure/ and exp Disease Transmission, Infectious/ (218)

59 Environmental Exposure/ and transmi*.tw,kf. (1115)

60 Environmental Exposure/ae [adverse effects] (18548)

61 (expos* adj3 (COVID-19 or COVID19 or coronavirus* or corona virus* or 2019-nCoV or 19nCoV or 2019nCoV or nCoV or n-CoV or "CoV 2" or CoV2 or SARS-CoV-2 or SARS-CoV2 or SARSCoV-2 or SARSCoV2 or SARS2 or SARS-2 or disease or infection* or virus*) adj5 (spread? or transmi*)).tw,kf. (347)

62 Occupational Exposure/ and tm.fs. (2161)

63 Occupational Exposure/ and exp Disease Transmission, Infectious/ (1418)

64 Occupational Exposure/ and transmi*.tw,kf. (1808)

65 Occupational Exposure/ae [adverse effects] (21298)

66 ((crowd* or mass or high-density or indoor or (poor* adj2 ventilat*)) adj3 (gathering? or event? or location? or setting?) adj3 risk?).tw,kf. (56)

67 ((activit* or area? or event? or gathering? or location? or setting?) adj3 risk?).tw,kf. (51799)

68 ((bar or bars or choir? or church* or cinema? or concert? or cruise or cruising or cruises or dense* congregat* or dining or factory or factories or grocery store? or gym or gyms or high occupanc* or jail or jails or mine or mines or mosque? or music event? or nightclub? or night club? or nosocomial* or office? or prison? or processing plant? or public transit* or public transport* or religious gathering? or religious event? or restaurant? or shared accommodation* or shared living or shop or shops or shopping or shout or shouting or sing or singing or sport* or studio? or supermarket? or taxi? or temple? or theatre? or theater? or tourist? or workplace? or work place?) adj3 risk?).tw,kf. (5487)

69 ((infection? or outbreak?) adj3 (hotspot? or hot spot? or source?)).tw,kf. (15371)

70 source finding.tw,kf. (14)

71 (contact? adj2 (trace or traced or traces or tracing) adj3 (enhanced or retrospective* or backward*)).tw,kf. (52)

72 secondary attack rate?.tw,kf. (382)

73 or/38-72 [TRANSMISSION SOURCES] (354707)

74 37 and 73 [COVID-19 DISEASE TRANSMISSION - SOURCES] (5088)

75 exp Animals/ not Humans/ (4889653)

76 74 not 75 [ANIMAL-ONLY REMOVED] (4990)

77 (comment or editorial or news or newspaper article).pt. (1537716)

78 76 not 77 [OPINION PIECES REMOVED] (4849)

79 limit 78 to yr="2019-current" (4594)

***************************

**Peer review assessment: this section to be filled in by the reviewer**

|  | Reviewer: Kaitryn Campbell | Email: [kcamlolo668@gmail.com](mailto:kcamlolo668@gmail.com) | Date completed: 28 Sep 2021 |
| --- | --- | --- | --- |
|  |  |  |  |

Do you wish to be acknowledged? (If yes, the review team will be advised to add an acknowledgement to any publications related to this work). Yes please.

The suggested acknowledgement is “We thank Kaitryn Campbell, MLIS, MSc (St. Joseph’s Healthcare Hamilton/McMaster University) for peer review of the MEDLINE search strategy.”

|  | **1. TRANSLATION** |  | | |  |
| --- | --- | --- | --- | --- | --- |
| A -­‐No revisions | X |  |  |  |  |
| B -­‐ Revision(s) suggested |  |  |  |  |  |
| C -­‐ Revision(s) required |  |  |  |  |  |

If “B” or “C,” please provide an explanation or example:

**2. BOOLEAN AND PROXIMITY OPERATORS**

| A -­‐No revisions | X |
| --- | --- |
| B -­‐ Revision(s) suggested |  |
| C -­‐ Revision(s) required |  |

If “B” or “C,” please provide an explanation or example:

**3. SUBJECT HEADINGS**

| A -­‐No revisions | X |
| --- | --- |
| B -­‐ Revision(s) suggested |  |
| C -­‐ Revision(s) required |  |

If “B” or “C,” please provide an explanation or example:

**4. TEXT WORD SEARCHING**

| A -­‐No revisions |  |
| --- | --- |
| B -­‐ Revision(s)suggested | X |
| C -­‐ Revision(s) required |  |

If “B” or “C,” please provide an explanation or example:

Line 68, may want to consider just including: “congregat*” , instead of “dense* congregat*” to accommodate all congregate settings

**5. SPELLING, SYNTAX, AND LINE NUMBERS**

| A -­‐No revisions | X |
| --- | --- |
| B -­‐ Revision(s)suggested |  |
| C -­‐ Revision(s) required |  |

If “B” or “C,” please provide an explanation or example:

**6. LIMITS AND FILTERS**

| A -­‐No revisions | X |
| --- | --- |
| B -­‐ Revision(s) suggested |  |
| C -­‐ Revision(s) required |  |

If “B” or “C,” please provide an explanation or example:

OVERALL EVALUATION (Note: If one or more “revision required” is noted above, the response below must be “revisions required”.)

| A -­‐No revisions | X |
| --- | --- |
| B -­‐ Revision(s) suggested |  |
| C -­‐ Revision(s) required |  |

Additional comments:

No errors or omissions detected. Looks solid, just 1 suggestion.
