# Supplementary material for: SARS-CoV-2 transmission risk for common group activities and settings: a living scoping review
Source: Eur J Public Health. 2023 Nov 23;34(1):196–201. doi: 10.1093/eurpub/ckad195 (PMC10843946; doi:10.1093/eurpub/ckad195)
Supplement: ckad195_Supplementary_Data [file ckad195_supplementary_data.zip › ckad195_Supplementary_Data/ejph-2023-07-om-0370-File005.docx]

# Appendix 2. Search Strategy

Risk of COVID-19 transmission

Final Strategy

2021 Sep 29

Ovid Multifile

Database: Embase Classic+Embase <1947 to 2021 September 28>, Ovid MEDLINE(R) ALL <1946 to September 28, 2021>

Search Strategy:

--------------------------------------------------------------------------------

1 COVID-19/ (148091)

2 SARS-CoV-2/ (100122)

3 Coronavirus/ (13334)

4 Betacoronavirus/ (40935)

5 Coronavirus Infections/ (56824)

6 (COVID-19 or COVID19).tw,kf. (320590)

7 ((coronavirus* or corona virus*) and (hubei or wuhan or beijing or shanghai)).tw,kf. (10811)

8 (wuhan adj5 virus*).tw,kf. (565)

9 (2019-nCoV or 19nCoV or 2019nCoV).tw,kf. (3685)

10 (nCoV or n-CoV or "CoV 2" or CoV2).tw,kf. (123287)

11 (SARS-CoV-2 or SARS-CoV2 or SARSCoV-2 or SARSCoV2 or SARS2 or SARS-2 or severe acute respiratory syndrome coronavirus 2).tw,kf. (125357)

12 (2019-novel CoV or Sars-coronavirus2 or Sars-coronavirus-2 or SARS-like coronavirus* or ((novel or new or nouveau) adj2 (CoV or nCoV or covid or coronavirus* or corona virus or Pandemi*2)) or (coronavirus* and pneumonia)).tw,kf. (41639)

13 (novel coronavirus* or novel corona virus* or novel CoV).tw,kf. (20616)

14 ((coronavirus* or corona virus*) adj2 "2019").tw,kf. (71061)

15 ((coronavirus* or corona virus*) adj2 "19").tw,kf. (11428)

16 ("coronavirus 2" or "corona virus 2").tw,kf. (37846)

17 (OC43 or NL63 or 229E or HKU1 or HCoV* or Sars-coronavirus*).tw,kf. (8157)

18 COVID-19.rx,px,ox. or severe acute respiratory syndrome coronavirus 2.os. (8943)

19 (coronavirus* or corona virus*).ti. (47940)

20 COVID.ti. (251662)

21 ("B.1.1.7" or "B.1.351" or "B.1.617" or "B.1.427" or "B.1.429").tw,kf,rx,px,ox. (1365)

22 ("P.1" and (Brazil* or variant?)).tw,kf,rx,px,ox. (3729)

23 (((alpha or beta or delta or eta or gamma or iota or kappa or lambda) adj3 variant?) and (coronavirus* or corona virus* or covid*)).tw,kf. (412)

24 or/1-23 [COVID-19] (393412)

25 COVID-19/tm [Transmission] (3805)

26 Coronavirus Infections/tm [Transmission] (4706)

27 exp Disease Transmission, Infectious/ (293982)

28 (transmit* or transmissi* or infectiousness* or infectivit*).tw,kf. (1271747)

29 (communit* adj3 spread*).tw,kf. (2186)

30 ((COVID-19 or COVID19) adj5 (caus* or pass or passed or passes or passing or spread*)).tw,kf. (34561)

31 ((coronavirus* or corona virus*) adj5 (caus* or pass or passed or passes or passing or spread*)).tw,kf. (18035)

32 ((2019-nCoV or 19nCoV or 2019nCoV) adj5 (caus* or pass or passed or passes or passing or spread*)).tw,kf. (418)

33 ((nCoV or n-CoV or "CoV 2" or CoV2) adj5 (caus* or pass or passed or passes or passing or spread*)).tw,kf. (16537)

34 ((SARS-CoV-2 or SARS-CoV2 or SARSCoV-2 or SARSCoV2 or SARS2 or SARS-2) adj5 (caus* or pass or passed or passes or passing or spread*)).tw,kf. (17185)

35 ((virus* or infection*) adj5 (caus* or pass or passed or passes or passing or spread*)).tw,kf. (394427)

36 or/25-35 [TRANSMISSION] (1789630)

37 24 and 36 [COVID-19 DISEASE TRANSMISSION] (96534)

38 cluster analysis/ (126067)

39 (cluster* adj3 (analys* or analyz*)).tw,kf. (99459)

40 (cluster* not (randomi#ed or controlled trial? or RCT or RCTs or clinical trial?)).ti. (126879)

41 (cluster* adj3 (disease? or infecti* or virus*)).tw,kf. (11868)

42 (cluster* adj3 source?).tw,kf. (1454)

43 (cluster* adj3 (COVID-19 or COVID19 or coronavirus* or corona virus* or 2019-nCoV or 19nCoV or 2019nCoV or nCoV or n-CoV or "CoV 2" or CoV2 or SARS-CoV-2 or SARS-CoV2 or SARSCoV-2 or SARSCoV2 or SARS2 or SARS-2)).tw,kf. (1071)

44 case cluster*.tw,kf. (739)

45 Carrier State/ (59222)

46 ((carry* or carrier?) adj2 (infect* or state?)).tw,kf. (14149)

47 (superspread* or super-spread*).tw,kf. (1139)

48 over* dispers*.tw,kf. (1544)

49 (transmi* adj5 (event? or hotspot? or hot spot?)).tw,kf. (6419)

50 (transmi* adj5 (chain? or cascade? or cluster?)).tw,kf. (7984)

51 (transmi* adj3 (risk* or setting?)).tw,kf. (47196)

52 Asymptomatic Infections/ep [epidemiology] (1280)

53 Risk Factors/ and tm.fs. (20258)

54 Risk Factors/ and exp Disease Transmission, Infectious/ (22569)

55 Risk Factors/ and transmi*.tw,kf. (43511)

56 Risk Factors/ and exp Leisure Activities/ (14910)

57 Environmental Exposure/ and tm.fs. (674)

58 Environmental Exposure/ and exp Disease Transmission, Infectious/ (1835)

59 Environmental Exposure/ and transmi*.tw,kf. (3492)

60 Environmental Exposure/ae [adverse effects] (22511)

61 (expos* adj3 (COVID-19 or COVID19 or coronavirus* or corona virus* or 2019-nCoV or 19nCoV or 2019nCoV or nCoV or n-CoV or "CoV 2" or CoV2 or SARS-CoV-2 or SARS-CoV2 or SARSCoV-2 or SARSCoV2 or SARS2 or SARS-2 or disease or infection* or virus*) adj5 (spread? or transmi*)).tw,kf. (744)

62 Occupational Exposure/ and tm.fs. (2180)

63 Occupational Exposure/ and exp Disease Transmission, Infectious/ (4227)

64 Occupational Exposure/ and transmi*.tw,kf. (4256)

65 Occupational Exposure/ae [adverse effects] (26284)

66 ((crowd* or mass or high-density or indoor or (poor* adj2 ventilat*)) adj3 (gathering? or event? or location? or setting?) adj3 risk?).tw,kf. (125)

67 ((activit* or area? or event? or gathering? or location? or setting?) adj3 risk?).tw,kf. (130728)

68 ((bar or bars or choir? or church* or cinema? or concert? or congregat* or correctional facilit* or cruise or cruising or cruises or dining or factory or factories or grocery store? or gym or gyms or high occupanc* or jail or jails or mine or mines or mosque? or music event? or nightclub? or night club? or nosocomial* or office? or "place? of worship*" or prison? or processing plant? or public transit* or public transport* or religious gathering? or religious event? or restaurant? or shared accommodation* or shared living or shop or shops or shopping or shout or shouting or sing or singing or sport* or studio? or supermarket? or taxi? or temple? or theatre? or theater? or tourist? or workplace? or work place?) adj3 risk?).tw,kf. (12761)

69 ((infection? or outbreak?) adj3 (hotspot? or hot spot? or source?)).tw,kf. (37313)

70 source finding.tw,kf. (38)

71 (contact? adj2 (trace or traced or traces or tracing) adj3 (enhanced or retrospective* or backward*)).tw,kf. (107)

72 (secondary attack? adj2 rate?).tw,kf. (839)

73 or/38-72 [TRANSMISSION SOURCES] (721107)

74 37 and 73 [COVID-19 DISEASE TRANSMISSION - SOURCES] (10790)

75 exp Animals/ not Humans/ (17676873)

76 74 not 75 [ANIMAL-ONLY REMOVED] (9377)

77 (comment or editorial or news or newspaper article).pt. (2243325)

78 76 not 77 [OPINION PIECES REMOVED] (9116)

79 limit 78 to yr="2019-current" (8666)

80 79 use medall [MEDLINE RECORDS] (4648)

81 coronavirus disease 2019/ (258467)

82 severe acute respiratory syndrome coronavirus 2/ (128757)

83 Coronavirinae/ (5207)

84 Betacoronavirus/ (40935)

85 coronavirus infection/ (57825)

86 (COVID-19 or COVID19).tw,kw. (319801)

87 ((coronavirus* or corona virus*) and (hubei or wuhan or beijing or shanghai)).tw,kw. (10725)

88 (wuhan adj5 virus*).tw,kw. (572)

89 (2019-nCoV or 19nCoV or 2019nCoV).tw,kw. (3538)

90 (nCoV or n-CoV or "CoV 2" or CoV2).tw,kw. (106585)

91 (SARS-CoV-2 or SARS-CoV2 or SARSCoV-2 or SARSCoV2 or SARS2 or SARS-2 or severe acute respiratory syndrome coronavirus 2).tw,kw. (124333)

92 (2019-novel CoV or Sars-coronavirus2 or Sars-coronavirus-2 or SARS-like coronavirus* or ((novel or new or nouveau) adj2 (CoV or nCoV or covid or coronavirus* or corona virus or Pandemi*2)) or (coronavirus* and pneumonia)).tw,kw. (39949)

93 (novel coronavirus* or novel corona virus* or novel CoV).tw,kw. (20195)

94 ((coronavirus* or corona virus*) adj2 "2019").tw,kw. (68862)

95 ((coronavirus* or corona virus*) adj2 "19").tw,kw. (10405)

96 ("coronavirus 2" or "corona virus 2").tw,kw. (35602)

97 (OC43 or NL63 or 229E or HKU1 or HCoV* or Sars-coronavirus*).tw,kw. (8139)

98 (coronavirus* or corona virus*).ti. (47940)

99 COVID.ti. (251662)

100 ("B.1.1.7" or "B.1.351" or "B.1.617" or "B.1.427" or "B.1.429").tw,kw. (1355)

101 ("P.1" and (Brazil* or variant?)).tw,kw. (3673)

102 (((alpha or beta or delta or eta or gamma or iota or kappa or lambda) adj3 variant?) and (coronavirus* or corona virus* or covid*)).tw,kw. (390)

103 or/81-102 [COVID-19] (400797)

104 disease transmission/ (105088)

105 virus transmission/ (73973)

106 (transmit* or transmissi* or infectiousness* or infectivit*).tw,kw. (1258047)

107 (communit* adj3 spread*).tw,kw. (2188)

108 ((COVID-19 or COVID19) adj5 (caus* or pass or passed or passes or passing or spread*)).tw,kw. (34621)

109 ((coronavirus* or corona virus*) adj5 (caus* or pass or passed or passes or passing or spread*)).tw,kw. (18058)

110 ((2019-nCoV or 19nCoV or 2019nCoV) adj5 (caus* or pass or passed or passes or passing or spread*)).tw,kw. (418)

111 ((nCoV or n-CoV or "CoV 2" or CoV2) adj5 (caus* or pass or passed or passes or passing or spread*)).tw,kw. (16520)

112 ((SARS-CoV-2 or SARS-CoV2 or SARSCoV-2 or SARSCoV2 or SARS2 or SARS-2) adj5 (caus* or pass or passed or passes or passing or spread*)).tw,kw. (17206)

113 ((virus* or infection*) adj5 (caus* or pass or passed or passes or passing or spread*)).tw,kw. (394573)

114 or/104-113 [TRANSMISSION] (1720568)

115 103 and 114 [COVID-19 DISEASE TRANSMISSION] (90959)

116 cluster analysis/ (126067)

117 (cluster* adj3 (analys* or analyz*)).tw,kw. (96671)

118 (cluster* not (randomi#ed or controlled trial? or RCT or RCTs or clinical trial?)).ti. (126879)

119 (cluster* adj3 (disease? or infecti* or virus*)).tw,kw. (11916)

120 (cluster* adj3 source?).tw,kw. (1451)

121 (cluster* adj3 (COVID-19 or COVID19 or coronavirus* or corona virus* or 2019-nCoV or 19nCoV or 2019nCoV or nCoV or n-CoV or "CoV 2" or CoV2 or SARS-CoV-2 or SARS-CoV2 or SARSCoV-2 or SARSCoV2 or SARS2 or SARS-2)).tw,kw. (1249)

122 case cluster*.tw,kw. (739)

123 disease carrier/ (37003)

124 ((carry* or carrier?) adj2 (infect* or state?)).tw,kw. (13671)

125 superspreader/ (82)

126 (superspread* or super-spread*).tw,kw. (1137)

127 over* dispers*.tw,kw. (1545)

128 (transmi* adj5 (event? or hotspot? or hot spot?)).tw,kw. (6414)

129 (transmi* adj5 (chain? or cascade? or cluster?)).tw,kw. (7958)

130 (transmi* adj3 (risk* or setting?)).tw,kw. (47374)

131 asymptomatic infection/ep [epidemiology] (1341)

132 risk factor/ and disease transmission/ (12548)

133 risk factor/ and virus transmission/ (6237)

134 risk factor/ and transmi*.tw,kw. (51556)

135 risk factor/ and leisure/ (3500)

136 infection risk/ and disease transmission/ (6064)

137 infection risk/ and virus transmission/ (10200)

138 virus transmission/ and transmi*.tw,kw. (42884)

139 virus transmission/ and leisure/ (67)

140 environmental exposure/ and disease transmission/ (826)

141 environmental exposure/ and virus transmission/ (411)

142 environmental exposure/ and transmi*.tw,kw. (3452)

143 environmental exposure/ae [adverse effects] (22511)

144 environmental exposure/ and attributable risk/ (150)

145 environmental exposure/ and population risk/ (369)

146 (expos* adj3 (COVID-19 or COVID19 or coronavirus* or corona virus* or 2019-nCoV or 19nCoV or 2019nCoV or nCoV or n-CoV or "CoV 2" or CoV2 or SARS-CoV-2 or SARS-CoV2 or SARSCoV-2 or SARSCoV2 or SARS2 or SARS-2 or disease or infection* or virus*) adj5 (spread? or transmi*)).tw,kw. (769)

147 occupational exposure/ and disease transmission/ (1724)

148 occupational exposure/ and virus transmission/ (926)

149 occupational exposure/ and transmi*.tw,kw. (4199)

150 occupational exposure/ae [adverse effects] (26284)

151 occupational exposure/ and attributable risk/ (86)

152 occupational exposure/ and population risk/ (152)

153 ((crowd* or mass or high-density or indoor or (poor* adj2 ventilat*)) adj3 (gathering? or event? or location? or setting?) adj3 risk?).tw,kw. (125)

154 ((activit* or area? or event? or gathering? or location? or setting?) adj3 risk?).tw,kw. (130616)

155 ((bar or bars or choir? or church* or cinema? or concert? or congregat* or correctional facilit* or cruise or cruising or cruises or dining or factory or factories or grocery store? or gym or gyms or high occupanc* or jail or jails or mine or mines or mosque? or music event? or nightclub? or night club? or nosocomial* or office? or "place? of worship*" or prison? or processing plant? or public transit* or public transport* or religious gathering? or religious event? or restaurant? or shared accommodation* or shared living or shop or shops or shopping or shout or shouting or sing or singing or sport* or studio? or supermarket? or taxi? or temple? or theatre? or theater? or tourist? or workplace? or work place?) adj3 risk?).tw,kw. (12776)

156 (bar or bars or choir? or church* or cinema? or concert? or congregat* or correctional facilit* or cruise or cruising or cruises or dining or factory or factories or grocery store? or gym or gyms or high occupanc* or jail or jails or mine or mines or mosque? or music event? or nightclub? or night club? or nosocomial* or office? or "place? of worship*" or prison? or processing plant? or public transit* or public transport* or religious gathering? or religious event? or restaurant? or shared accommodation* or shared living or shop or shops or shopping or shout or shouting or sing or singing or sport* or studio? or supermarket? or taxi? or temple? or theatre? or theater? or tourist? or workplace? or work place?).tw,kw. and (attributable risk/ or infection risk/ or population risk/) (5323)

157 ((infection? or outbreak?) adj3 (hotspot? or hot spot? or source?)).tw,kw. (37238)

158 source finding.tw,kw. (37)

159 (contact? adj2 (trace or traced or traces or tracing) adj3 (enhanced or retrospective* or backward*)).tw,kw. (107)

160 (secondary attack? adj2 rate?).tw,kw. (827)

161 or/116-160 [TRANSMISSION SOURCES] (726603)

162 115 and 161 [COVID-19 DISEASE TRANSMISSION - SOURCES] (14594)

163 exp animal/ or exp animal experimentation/ or exp animal model/ or exp animal experiment/ or nonhuman/ or exp vertebrate/ (56311550)

164 exp human/ or exp human experimentation/ or exp human experiment/ (43862214)

165 163 not 164 (12451223)

166 162 not 165 [ANIMAL-ONLY REMOVED] (13948)

167 editorial.pt. (1286318)

168 166 not 167 [OPINION PIECES REMOVED] (13562)

169 limit 168 to yr="2019-current" (12803)

170 169 use emczd [EMBASE RECORDS] (8844)

171 80 or 170 [BOTH DATABASES] (13492)

172 limit 171 to yr="2021-current" (6319)

173 172 use medall (2390)

174 remove duplicates from 173 (2342)

175 172 use emczd (3929)

176 remove duplicates from 175 (3907)

177 174 or 176 [MEDLINE, EMBASE RECORDS] (6249)

178 (202101* or 202102* or 202103* or 202104*).dt,dc. (1229168)

179 177 and 178 (2128)

180 remove duplicates from 179 (1818)

181 177 not 178 (4121)

182 remove duplicates from 181 (3360)

183 180 or 182 [TOTAL UNIQUE RECORDS - 2021] (5178)

184 limit 171 to yr="2020" (7105)

185 (202001* or 202002* or 202003* or 202004* or 202005* or 202006* or 202007*).dt,dc. (1786658)

186 184 and 185 (2445)

187 remove duplicates from 186 (2075)

188 184 not 185 (4660)

189 remove duplicates from 188 (3944)

190 187 or 189 [TOTAL UNIQUE RECORDS - 2020] (6019)

191 limit 171 to yr="2019" (68)

192 remove duplicates from 191 [TOTAL UNIQUE RECORDS - 2019] (54)

193 183 or 190 or 192 [TOTAL UNIQUE RECORDS - 2019-2021] (11251)

194 193 use medall [MEDLINE UNIQUE RECORDS] (4578)

195 193 use emczd [EMBASE UNIQUE RECORDS] (6673)

***************************
