# Supplementary material for: SARS-CoV-2 transmission risk for common group activities and settings: a living scoping review
Source: Eur J Public Health. 2023 Nov 23;34(1):196–201. doi: 10.1093/eurpub/ckad195 (PMC10843946; doi:10.1093/eurpub/ckad195)
Supplement: ckad195_Supplementary_Data [file ckad195_supplementary_data.zip › ckad195_Supplementary_Data/ejph-2023-07-om-0370-File006.docx]

# Appendix 3. Eligibility Criteria

| **Criterion** | **Inclusion** | **Exclusion** |
| --- | --- | --- |
| **Participants** | Individuals of any age with a laboratory-confirmed diagnosis of COVID-19, using RT-PCR. |  |
| **Concept** | Any setting or activity listed by by the Department of Health (Ireland) Expert Advisory Group on Rapid Testing. A complete list is provided in Appendix 4  We also included settings like:   - Healthcare - Household - Specialized services (e.g., Dental, and personal care services^2^) - Using public transportation - Working in specific professions   Risk factors that contribute to risk (as reported by authors) | - Ineligible study design - Modelling study - Review (systematic, non-systematic and others) - Letter, editorial, reply - Case study/Individual COVID-19 outbreak study. - spatial-temporal, country, county-level COVID-19 infection/death - genomic epidemiology - Epidemiological characteristics of COVID-19 - Prevalence of COVID-19 - Irrelevant context - Sewage - Vaccine effectiveness - Policy changes (i.e., closure, lockdown) - Virology laboratory - Knowledge, attitude, social trust - Testing strategies - Not related to COVID-19 - Tool for identification of COVID-19 during activities - Monitoring of CO_2_ levels as a proxy of SARS-CoV-2 transmission risk - Viral load - Not applicable (Not relevant study) - Ineligible activity/setting - Animal Shelter - Correctional facilities - Military settings - Includes household settings only - Fomites and environment - Heterogeneity in contact behaviour - Work environment and protective measures - Mobile phones as source of transmission - Maternal or vertical transmission - No data on modes of SARS-CoV-2 transmission - Not published in English |
| **Context** | No restriction on geographic location (country) |  |
| **Types of sources of evidence** | - National or regional retrospective contact tracing studies - Observational studies with a comparator group that aim to estimate the risk of transmission in various settings (e.g., case-control studies, cohort studies) - Prospective contact tracing studies that use data from cases and non-cases to estimate the risk of onward transmission in different settings or due to different activities - Studies with information on travel-related transmission  only | - Evidence syntheses of outbreaks, clusters or super-spreading events (including syntheses of media reports) - Studies with no information on the setting or activity where transmission occurred - Studies that do not investigate the source of community transmission^1^ - Studies that do not investigate potential sources of transmission up to 14 days prior to symptom onset or diagnosis (in line with the WHO incubation period). [WHO COVID-19 Situation Report - 73](https://apps.who.int/iris/handle/10665/331686) - Mathematical modelling studies - Ecological studies that examine the trajectory of the pandemic after policy changes - Individual outbreak/cluster descriptive studies. - Individual media reports - Editorials / opinion pieces / guidance documents - Animal studies |
| **Language** | English |  |
