# Supplementary material for: SARS-CoV-2 transmission risk for common group activities and settings: a living scoping review
Source: Eur J Public Health. 2023 Nov 23;34(1):196–201. doi: 10.1093/eurpub/ckad195 (PMC10843946; doi:10.1093/eurpub/ckad195)
Supplement: ckad195_Supplementary_Data [file ckad195_supplementary_data.zip › ckad195_Supplementary_Data/ejph-2023-07-om-0370-File007.docx]

# Appendix 4. List of activities provided from the Department of Health (Ireland) Expert Advisory Group on Rapid Testing

- Coach tour activity (The nature of this activity encompasses the “tourist journey”)
- arrival/departure by sea
- arrival/departure by air
- travel to accommodation from airport or port
- accommodation stay
- hospitality
- going to a restaurant
- going to a bars
- visiting visitor attractions
- Business Conferences
- Hospitality
- Going to a restaurant
- Going to a Bar
- Live entertainment – attending
- a concert seated
- a concert non-seated
- a concert indoors
- a concert outdoors
- a nightclub
- a seated theatre performance
- a children’s art class
- an adults’ art class
- a children’s dance class
- an adults’ dance class.
- Sports
- attending a match
- going to the gym to train individually
- doing an indoor exercise class
- Education setting
- Trade show or exhibition
