# Supplementary material for: SARS-CoV-2 transmission risk for common group activities and settings: a living scoping review
Source: Eur J Public Health. 2023 Nov 23;34(1):196–201. doi: 10.1093/eurpub/ckad195 (PMC10843946; doi:10.1093/eurpub/ckad195)
Supplement: ckad195_Supplementary_Data [file ckad195_supplementary_data.zip › ckad195_Supplementary_Data/ejph-2023-07-om-0370-File008.docx]

# Appendix 5. Characteristics of Included Studies

| **Country** | **Author & Year of Publication** | **Study Design** | **Dates** | **Sample Size** | **Setting/Activity** | **Reported preventive measures** | **Risk factors** | **Risk level (reported by study authors)** |  |
| --- | --- | --- | --- | --- | --- | --- | --- | --- | --- |
| International, Asia (Hong Kong, Japan, Singapore, Taiwan, Thailand, and Vietnam) | Lan 2020 | Retrospective cohort | January 2020 - March 2020 | 103 cases | Workplaces | Not reported | Not reported | High |  |
|  |  |  |  |  | (Shop salesperson, |  |  |  |  |
|  |  |  |  |  | Domestic housekeepers, |  |  |  |  |
|  |  |  |  |  | Religious professionals, |  |  |  |  |
|  |  |  |  |  | Construction labourers, |  |  |  |  |
|  |  |  |  |  | Police officers, |  |  |  |  |
|  |  |  |  |  | Fire fighter, |  |  |  |  |
|  |  |  |  |  | Security guards) |  |  |  |  |
|  |  |  |  |  | Transportation |  | Not reported | Low |  |
|  |  |  |  |  | (Car, taxi, and van drivers, |  |  |  |  |
|  |  |  |  |  | Locomotive engine drivers and related workers, |  |  |  |  |
|  |  |  |  |  | Bus and tram drivers) |  |  |  |  |
|  |  |  |  |  | Hospitality |  | Not reported | Low |  |
|  |  |  |  |  | (Tour guides, |  |  |  |  |
|  |  |  |  |  | Receptionists, |  |  |  |  |
|  |  |  |  |  | Waiter or bartenders, |  |  |  |  |
|  |  |  |  |  | Cooks) |  |  |  |  |
|  |  |  |  |  | Healthcare |  | Not reported | Low |  |
|  |  |  |  |  | (Medical doctors, |  |  |  |  |
|  |  |  |  |  | Personal care workers in health services) |  |  |  |  |
| International, Asia | Fan-yun 2021 | Cohort study | 23 January 2020 - 14 March 2020 | 690 | Workplaces (Any workplace (e.g., taxi drivers, tour guides, healthcare, etc.) | Not reported | Close contact and frequent contact with travelers | High |  |
| Australia | Macartney 2020 | Retrospective cohort | January 2020-April 2020 | 27 primary cases (1448 close contacts, 663 used in analysis) | Education (Primary & secondary schools) | Physical distancing, hygiene measures, and educational facility cleaning | Not Reported | Low |  |
|  |  |  |  |  |  |  |  |  |  |
| Australia | Ravindran 2020 | Retrospective cohort | 15 - 21 March 2020 | 41 | Events and entertainment (Wedding in Bali) | Understanding the risks and preventative measures to minimise the spread of COVID-19 | Close contact (shaking hands, kissing, dancing, sharing drinks, and sharing shisha) | High |  |
|  |  |  |  |  |  |  |  |  |  |
| Australia | Walker 2021 | Retrospective cohort | 1 March 2020 - 6 April 2020 | 223 | Hospitality (Cruise ship) | Ship based quarantine, enhanced health measures and access to onshore quarantine and isolation facilities | Close contact, cabin mate | High |  |
|  |  |  |  |  |  |  |  |  |  |
| Belgium | Meuris 2021 | Prospective cohort | September 2020 - December 2020 | 181 | Education (Kindergarten and primary school) | The standards implemented in the school in September were hand hygiene and facial mask wearing for adults (allowing breaks within the classroom, when seated at their desk). Mask wearing and social distance between children was never implemented within the classroom nor during the breaks. From 16 November, children had to eat inside the classroom and adults could take off their mask only outside while respecting 1.5m of distance. | Between children within school, observed transmission within households may have originated from someone who was infected within the school | NR, but incidence of COVID-19 among children comparable to that observed among teachers and parents. |  |
|  |  |  |  |  |  |  |  |  |  |
|  |  |  |  |  | (Kindergarten and primary school) |  | Between teachers or employees within school, observed transmission within households may have originated from someone who was infected within the school | Not Reported, but incidence of COVID-19 among children comparable to that observed among teachers and parents. |  |
|  |  |  |  |  |  |  |  |  |  |
|  |  |  |  |  |  |  |  |  |  |
|  |  |  |  |  |  |  |  |  |  |
| Brazil | Afonso 2021 | Cross-sectional | 15 June 2020 - 28 October 2020 | Not Reported | Household | Not Reported | Close contact | Not Reported |  |
| Brazil | Costa 2020 | Cross-sectional | 14 May - 28 May 2020 | 2415 | Healthcare (Hospital) | Not Reported | Distance from home to work, use of public transportation, residing in poorer neighbourhoods. | Unclear |  |
|  |  |  |  |  |  |  |  |  |  |
| Brazil | Oliveira 2021 | Cross-sectional | March 2020 - July 2020 | 1996 | Healthcare (Hospital) | N95 masks, PPE, cleaning, and disinfection | Professional category of cleaning, male gender | High |  |
|  |  |  |  |  |  |  |  |  |  |
| Canada | Bark 2021 | Descriptive study | 10 September - 18 December 2020 | 699 | Education (Kindergarten to Grade 12) | School-based IPC measures | Shared classroom, socializing, staff room | Low |  |
|  |  |  |  |  |  |  |  |  |  |
| Canada | Carazo 2021 | cross-sectional | 1 March 2020 - 14 June 2020 | 4542 HCWs | Healthcare (Acute-care hospitals (ACHs) and long-term care facilities (LTCFs)) | Hand hygiene, mask, physical distancing | Lack of preparedness, gaps in IPC, and a low perception of risk from contacts with coworkers | Not Reported |  |
|  |  |  |  |  |  |  |  |  |  |
|  |  |  |  |  | Household (Households of HCW) |  | Smaller household size | Not Reported |  |
|  |  |  |  |  |  |  |  |  |  |
| Canada | Choi 2021 | Contact tracing | 12 April 2021 - 30 June 2021 | 69 primary cases (392 close contacts) | Education (K-12 schools) | Mask | Close contact at school (secondary asymptomatic transmission) | Low |  |
|  |  |  |  |  |  |  |  |  |  |
|  |  |  |  |  | Household |  | Close contact of primary cases at households | High |  |
|  |  |  |  |  |  |  |  |  |  |
| Canada | Fisman 2020 | Retrospective cohort | 3 March 2020 - 11 April 2020 | 627 LTC facilities, 1731315 (older than 69 years living in Ontario) | Healthcare (Long term care facilities) | Not Reported | Direct exposure with infected LTC facility staff and associated mortality among LTC residents | High |  |
|  |  |  |  |  |  |  |  |  |  |
|  |  |  |  |  |  |  | Not Reported | Not Reported |  |
|  |  |  |  |  | Other (Community living) |  |  |  |  |
|  |  |  |  |  |  |  |  |  |  |
| Canada | Kain 2021 | Retrospective cohort | 1 March 2020 – 21 May 2020 | 113 | Healthcare (Long term care facilities) | Masking, PPE | Direct exposure | High |  |
|  |  |  |  |  |  |  |  |  |  |
| Canada | Kevin 2021 | Retrospective cohort | 29 March 2020 - 20 May 2020 | 78000 | Healthcare (Long term care facilities) | Testing, face masks, hand hygiene, PPE, quarantine, isolation | High crowding index (2.0-4.0), shared rooms | High |  |
|  |  |  |  |  |  |  |  |  |  |
| Canada | Lunney 2021 | Prospective cohort | 2 November 2020 - 30 November 2020 | 9535 | Transportation (International travel) | Border control, quarantine | Travellers, departure from high-risk countries | High |  |
|  |  |  |  |  |  |  |  |  |  |
| Canada | Williams 2021 | Prospective cohort | 1 October 2020 - 30 April 2021 | 42 HCW, 214 contacts | Healthcare (Acute care hospital, long-term care home and rehabilitation/complex care hospitals) | Universal masking, embedded with other infection control practices | Not reported | Low |  |
|  |  |  |  |  |  |  |  |  |  |
| Chile | Olmos 2021 | Cross-sectional | May 2020 - July 2020 | 413 | Healthcare (Private hospital clinic) | Masks, eye protection, a disposable gown with arm coverings and disposable gloves., hand hygiene, social distancing | Not Reported | Low |  |
|  |  |  |  |  |  |  |  |  |  |
| China | Ai 2021 | Surveillance | 25 January 2020 t- 29 February 2020 | 134 clusters involving 617 cases. | Household (Eating and living together, visiting relatives, etc.) | Isolation of infected patients | Not Reported | Not Reported |  |
|  |  |  |  |  |  |  |  |  |  |
|  |  |  |  |  | Events and entertainment (Playing cards with neighbour- hood friends, leisure in public bathrooms, neighbourhood, or friend communication, etc. |  |  |  |  |
|  |  |  |  |  |  |  |  |  |  |
|  |  |  |  |  | Workplaces (Meeting, co-office, etc.) |  |  |  |  |
|  |  |  |  |  |  |  |  |  |  |
| China | Chua 2021 | Cross-sectional | December 2020 - January 2021 | 397 | Education (Primary and Secondary schools) | Social distancing, universal mask wearing, travel restrictions, border control, social distancing. In schools installing plastic partitions between classroom desks or canteen tables, minimizing group activities, reinforcing infection-control measures like teaching students good hand hygiene, and monitoring symptoms, providing basic protective equipment to teachers, and incorporating digital teaching tools in classrooms. Schools should also consider asking parents to declare whether other family members have been in recent contact with infected individuals. Students with contact histories should not return to school until they test negative. Students under quarantine at home could be supported by online learning. | Close contact | Low |  |
|  |  |  |  |  |  |  |  |  |  |
| China | Hu 2021 | Contact tracing | 4 January2020 - 14 March 2020 (Jan 4, 2020 - Jan 23, 2020, before lockdown data) | 5797 airline passengers (data from 177 planes) | Transportation (airplanes) | PPE (masks and googles (only few passengers might have used them) | Relationships and contacts between passengers | High |  |
|  |  |  |  |  |  |  |  |  |  |
| China | Kwok 2021 | Retrospective cohort | January 2020-August 2020 | 3512 [1110 (wave 1) 2402 (wave 2)] | Events and entertainment  Social settings (friend, partner, neighbor, dating, meal, banquet, dinner, restaurant, bar, coffee, shopping center, gym, mah-jong and majoring, tutorial, transportation (bus and taxi), travel, birthday party, beauty parlour, chatting, hugging and contact) | Social distancing (high proportion of transmission rates occurring within same age group supports the ban on gatherings outside households) | Not Reported | High |  |
|  |  |  |  |  |  |  |  |  |  |
| China | Lai 2021 | Data linkage | 1 Jan 2020 - 9 Feb 2020 | 9684 | Healthcare (Hospital) | Isolation of HCWs, infection prevention and control guidelines, training in infection control measures | Being a nurse, working in other clinical departments than fever clinics or wards | High |  |
|  |  |  |  |  |  |  |  |  |  |
| China | Liu 2021 | Retrospective cohort | 23 January 2020 - 8 January 2021 | 9153 | Events and entertainment (Singing and dancing venues/clubs) | Not reported | Not Reported | High |  |
|  |  |  |  |  |  |  |  |  |  |
|  |  |  |  |  | Events and entertainment (Department store) |  |  |  |  |
|  |  |  |  |  |  |  | Not Reported | Not Reported |  |
|  |  |  |  |  | Healthcare (Hospitals) |  |  |  |  |
|  |  |  |  |  |  |  | Not Reported | Not Reported |  |
|  |  |  |  |  | Workplaces (Park, construction site) |  |  |  |  |
|  |  |  |  |  |  |  | Not Reported | Not Reported |  |
| China | Martín-sánchez 2021 | Retrospective cohort | January 2020-September 2020 | 2425 | Workplaces (Classified as mask-on setting) | Masking | Not reported | Low |  |
|  |  |  |  |  |  |  |  |  |  |
|  |  |  |  |  | Healthcare (Residential care homes) |  | Not reported | Low |  |
|  |  |  |  |  |  |  |  |  |  |
|  |  |  |  |  |  |  |  |  |  |
|  |  |  |  |  | Education (schools) |  | Not reported | Low |  |
|  |  |  |  |  |  |  |  |  |  |
|  |  |  |  |  |  |  |  |  |  |
|  |  |  |  |  | Transportation (Modes of transportation) |  | Not reported | Low |  |
|  |  |  |  |  | Events and entertainment (Social settings) |  | Not reported | High |  |
|  |  |  |  |  |  |  |  |  |  |
|  |  |  |  |  |  |  |  |  |  |
|  |  |  |  |  | Household (Family households and roommates) |  | Not reported | High |  |
|  |  |  |  |  |  |  |  |  |  |
|  |  |  |  |  |  |  |  |  |  |
| China | Ran 2020 | Retrospective cohort | NR (follow-up ended on January 28 (only mentioned)) | 72 | Healthcare (University hospital) | PPE | Working in high-risk departments, longer duty hours, suboptimal hand hygiene | High |  |
|  |  |  |  |  |  |  |  |  |  |
| China | Wang 2020 | Cross-sectional | 1 January 2020 – 30 February 2020 | 92 | Healthcare (University hospital) | PPE (protective mask or surgical mask) | Insufficient use of PPE and touching the cheek, nose, mouth while working | High |  |
|  |  |  |  |  |  |  |  |  |  |
| China | Wang 2021 | Surveillance | 5 January 2020 - 12 February 2020 | 35 | Healthcare (Hospital) | PPE | Direct contact with the index patients, co-workers in the department gatherings | High |  |
|  |  |  |  |  |  |  |  |  |  |
| China | Wong 2021 | Retrospective cohort | January 2020-June 2020 | 1128 | Events and entertainment (Entertainment) | Contact tracing and quarantine, and early introduction of social distancing measures | Not reported | High |  |
|  |  |  |  |  |  |  |  |  |  |
|  |  |  |  |  | Hospitality (Restaurants, personalised services, workplace) |  | Not reported | Not reported |  |
|  |  |  |  |  |  |  |  |  |  |
|  |  |  |  |  |  |  |  |  |  |
| China | Xiaoke 2021 | Cohort study | 20 January 2020 – 19 February 2020 | 1,407 | Household | Travel bans | Higher risk of being infected outside of household for male people of age between 18 and 64 years | Unclear |  |
| China | Zhang 2020 | Contact tracing | Missing | Large cluster of 22 confirmed cases from 5 families | Events and entertainment (Gatherings) | Extensive testing, isolating | Close contact (shared meals) | High |  |
|  |  |  |  |  |  |  |  |  |  |
| China | Zhao 2020 | Retrospective cohort | 18 January 2020 - 8 March 2020 | 712 cases | Household (Estate (own home/relative’s home)) | Public awareness of epidemic prevention and control | Close contacts | High |  |
|  |  |  |  |  |  |  |  |  |  |
|  |  |  |  |  |  |  |  |  |  |
|  |  |  |  |  | Hospitality (Restaurant) |  | Dining, number of contacts, long exposure times | Not reported |  |
|  |  |  |  |  |  |  |  |  |  |
|  |  |  |  |  | Transportation (Traditional train; emus; aeroplane; taxi/private car; bus/metro) |  | Close contacts, number of contacts, long exposure times | High |  |
|  |  |  |  |  |  |  |  |  |  |
|  |  |  |  |  | Events and entertainment (Supermarket, shopping) |  | Close contacts, number of contacts, long exposure times | Not reported |  |
|  |  |  |  |  |  |  | Close contacts, number of contacts, long exposure times | Not reported |  |
|  |  |  |  |  | Healthcare (Hospitals, clinics) |  |  |  |  |
|  |  |  |  |  |  |  |  |  |  |
|  |  |  |  |  |  |  |  |  |  |
|  |  |  |  |  |  |  |  |  |  |
|  |  |  |  |  |  |  |  |  |  |
|  |  |  |  |  |  |  |  |  |  |
| China | Zhong 2020 | Cross-sectional | 10 January 2020 - 24 January2020 | 21807 travellers in top 100 cities of China and 13798 travellers in 15 cities in Hubei province | Transportation (Travel before the spring festival) | NR | Travel | High |  |
|  |  |  |  |  |  |  |  |  |  |
| Denmark | Meyer 2021 | Prospective cohort | 2 March 2020 onwards | 101 | Workplaces (Co-worker as a close COVID-19 contact) | Not Reported | Not reported | Not Reported |  |
|  |  |  |  |  |  |  |  |  |  |
|  |  |  |  |  | Transportation (Travel/contacting persons from endemic regions) |  | Close contact with a person travelling from endemic region | Not Reported |  |
|  |  |  |  |  |  |  |  |  |  |
| Denmark | Munch 2021 | Cohort study | 4-6 December 2020 | 617 | Events and entertainment  (Singing, shopping centres, cultural, religious, attending bars, social) | Quarantine, hand hygiene, social distancing | Close contact with the more people while attending different social events | High |  |
|  |  |  |  |  |  |  |  |  |  |
|  |  |  |  |  | Sports and activities (Indoor fitness centres) |  | Close contact with people while attending fitness centres | High |  |
|  |  |  |  |  |  |  |  |  |  |
|  |  |  |  |  |  |  |  |  |  |
|  |  |  |  |  |  |  |  |  |  |
|  |  |  |  |  | Transportation (Public transportation) |  | Travelling using public transport | Not reported |  |
|  |  |  |  |  |  |  |  |  |  |
|  |  |  |  |  |  |  |  |  |  |
| Denmark | Rasmussen 2021 | Prospective cohort | 27 March 2020 – 3 June 2020 | Not Reported | Healthcare (hospital) | PPE, testing, additional protective measures (plexiglass and hand washing) | No procedures or areas within the department were identified as exposing hcws to a higher risk | Low |  |
|  |  |  |  |  |  |  |  |  |  |
| Egypt | Mostafa 2020 | Cross-sectional | 22 April 2020 - 14 May 2020 | 4040 | Healthcare (a large governmental public health care facility) | Screening, testing, isolation, wearing PPE | Contact with COVID-19 suspected cases, confirmed cases, duration of contact | High |  |
|  |  |  |  |  |  |  |  |  |  |
|  |  |  |  |  |  |  |  |  |  |
| France | Contejean 2021 | Cohort Study | 24 February 2020 - 10 April 10, 2020 | 1344 | Healthcare | PPE (masks, gloves, gown), social distancing | Direct covid-19 patient-facing activities, inappropriate PPE use, exposure to other HCWs (colleagues) | High |  |
| France | Danis 2020 | Contact tracing | Not Reported | Not Reported | Accommodations (chalet) | School closure (infected pediatric case), isolation | Living in a same chalet | High |  |
|  |  |  |  |  |  |  |  |  |  |
| France | Delaugerre 2021 | RCT | 11 May - 25 May 2020 | 6678 | Events and entertainment (Live indoor concert) | Masking | Not Reported | Low |  |
|  |  |  |  |  |  |  |  |  |  |
| France | Gagneux-brunon 2020 | Cross-sectional | 25 March 2020 – 28 April 2020 | 514 | Healthcare (University hospital) | PPE, social distancing, hrdo-alcoholic handrub products | HCW to HCW transmission | Low |  |
|  |  |  |  |  |  |  |  |  |  |
| France | Grant 2021 | Case-control | 23 May 2021 - 13 August 2021 | 18194 | Events and entertainment (Bars and parties) | Not Reported | Not Reported | Not Reported |  |
|  |  |  |  |  |  |  |  |  |  |
|  |  |  |  |  |  |  |  |  |  |
|  |  |  |  |  |  |  |  |  |  |
|  |  |  |  |  |  |  |  |  |  |
|  |  |  |  |  |  |  |  |  |  |
|  |  |  |  |  | Household (Children going to schools) |  |  | Not Reported |  |
|  |  |  |  |  |  |  |  |  |  |
|  |  |  |  |  | Transportation (Taxi, carpooling, flying) |  |  | Not Reported |  |
|  |  |  |  |  | Accommodations (Shelters and social housing) |  |  | High |  |
| France | Gras-le guen 2021 | Retrospective cohort | August 2020-October 2020 | 12,400,000 students and 1,162,850 staff | Education (childcare, kindergarten, primary, middle, high school) | Hygiene and social distancing | Not reported | Low |  |
|  |  |  |  |  |  |  |  |  |  |
| France | Landoas 2021 | Prospective cohort | March 2020 - May 2020 | 259 patients | Healthcare (hospital) | Surgical masks, PPE, no visits, isolation of suspected cases, testing | No mask (roommates, shared activities etc.). | Low |  |
|  |  |  |  |  |  |  |  |  |  |
| Germany | Bahrs 2021 | Prospective cohort | 19 May 2020 - 19 June 2020 | 660 | Healthcare (Hospital as workplace) | Mandatory masking, PPE, infection prevention programs | Direct patient contact in areas with confirmed COVID-19 cases | High |  |
|  |  |  |  |  |  |  |  |  |  |
| Germany | Brandl 2020 | Surveillance | March - May 2020 | 110 cases | Healthcare (Long term care facilities) | Isolation, ban of visitors, PPE | Not Reported | High |  |
|  |  |  |  |  |  |  |  |  |  |
|  |  |  |  |  |  |  |  |  |  |
|  |  |  |  |  | Events and entertainment (Mass gathering events (Bavarian beer festivals)) |  | Not Reported | High |  |
|  |  |  |  |  | Events and entertainment (Birthday parties, funerals, or religious services) |  | Not Reported | Not reported |  |
|  |  |  |  |  |  |  |  |  |  |
|  |  |  |  |  |  |  |  |  |  |
|  |  |  |  |  |  |  |  |  |  |
| Germany | Kirsten 2021 | Prospective cohort | May 2020 -October 2020 | 2045 [1538 students, 507 teachers (first study visit)]  1779 [1334 students, 445 teachers (second study visit)] | Education (Secondary schools) | Not reported | Not Reported | Low |  |
|  |  |  |  |  |  |  |  |  |  |
| Germany | Loenenbach 2021 | Surveillance | January 2021 –February 2021 | 171 in childcare centres, 92 in households | Education (Childcare centres) | (i) cohort grouping with a reduced number of children and designated staff, and access to separate bathroom areas, (ii) playing outside only within same groups in assigned playground areas, (iii) staff wearing masks at least outside of the group rooms, (iv) parents not allowed to enter the building and mandatory mask-wearing during children’s drop off and pick up, and (v) conducting meetings digitally with staff and/or parents, if possible. | Encounter to the primary case (PC) of≥15 min within a distance of<1.5 m. | High (with VOC compared not non-VOC period) |  |
|  |  |  |  |  |  |  |  |  |  |
| Germany | Pauser 2021 | Cohort study | Unclear (November 2020 only mentioned) | 61 (as per the receipt of consent form) | Sports and activities | Masks (medical, filter), distancing | Close contact with the probable/diagnosed COVID-19 patient | High |  |
| Germany | Pokora 2021 | Cross-sectional | June 2020-September 2020 | 19072 employees | Other (Meat and poultry plants) | Masking, testing, ventilation, physical barriers, distancing, disinfection | Not Reported | Not reported |  |
|  |  |  |  |  |  |  |  |  |  |
|  |  |  |  |  | Other (ventilation) |  | Not Reported | Low |  |
|  |  |  |  |  |  |  |  |  |  |
|  |  |  |  |  | Other (Lower temperature conditions) |  | Not Reported | High |  |
|  |  |  |  |  |  |  |  |  |  |
|  |  |  |  |  |  |  |  |  |  |
| Germany | Schepers 2021 | Surveillance | 17 August 2020 – 10 November 2020 | 113 | Household | Masking, distancing | Clusters with more than one household | High |  |
| Germany | Schoeps 2021 | Surveillance | August 2020 - December 2020 | 14594 | Education (Schools and daycare centers) | Not reported | Association of teacher-indexes with teacher-secondaries, children-/student-index cases, schoolteachers were close contacts to student-indexes | Low |  |
|  |  |  |  |  |  |  |  |  |  |
| Germany | Schreiber 2021 | Cross-sectional | August 2020 – March 2021 | 1247 | Sports and activities (Amateur, youth and professional football (soccer) athletes) | Hygiene measures | On-field transmission | Low |  |
|  |  |  |  |  |  |  |  |  |  |
| Germany | Theuring 2021 | Prospective cohort | Nov-20 | 1119 | Education (Primary & secondary schools) | Vary between schools but include hygiene measures, distancing, absence rules if ill, ventilation, cohorting, staggering of teaching hours, and online teaching | Not reported | Low |  |
|  |  |  |  |  |  |  |  |  |  |
| Germany | Walker 2021 | Surveillance | August 2020 - December 2020 | 320 samples | Healthcare (University hospital) | Staff re-training, improved room ventilation, and upgrades to patient protective equipment; | Close contact, family members | Not reported |  |
|  |  |  |  |  |  |  |  |  |  |
| Greece | Maltezou 2021 | Prospective cohort | February 2020 - May 2020 | 1287 in public, 860 in referral | Healthcare (hospitals) | PPE, surgical masks | Working in a non-referral hospital compared with a coronavirus disease 2019 (COVID-19) referral hospital, working in a hospital with a high number of employees, and working in a hospital with an increased number of patients with COVID-19 | High |  |
|  |  |  |  |  |  |  |  |  |  |
|  |  |  |  |  |  |  |  |  |  |
|  |  |  |  |  |  |  |  |  |  |
| India | Chatterjee 2020 | case-control | April 2020 - May 2020 | 650 | Healthcare | PPE (masks, caps, gowns, gloves) | Close contact with COVID-19 suspected or confirmed patients, use of PPE, handling clinical specimens | High |  |
| India | Dutta 2021 | Prospective cohort | 31 March 2020 - 20 July 2020 | 5553 outpatients, 1224 healthcare workers | Healthcare (hospital) | Social distancing, masking, PPE, hygiene measures, waste, and disinfection, contact tracing, shorter shifts, testing | Not following social distancing and universal masking norms, when they either attended mass gatherings, social events, had tea or lunch breaks with their office colleagues who later tested positive | Low [*after Healthcare Personnel (HCP) policy was implemented] |  |
|  |  |  |  |  |  |  |  |  |  |
|  |  |  |  |  |  |  |  |  |  |
|  |  |  |  |  |  |  |  |  |  |
| India | Mansoor 2021 | Contact tracing | 1 March 2020 - 31 July 2020 | 106 | Healthcare (Hospital) | Donning and doffing procedures, PPE, training | Inappropriate PPE, direct contact | High |  |
|  |  |  |  |  |  |  |  |  |  |
| India | Pandrowala 2021 | Cohort study | March 2020 - Aug 2020 | 530 | Healthcare (Hospital) | Improvised infection control measures and reinforced basic preventive measures throughout the pandemic, PPE | Positive contact, public transport | High |  |
|  |  |  |  |  |  |  |  |  |  |
| India | Sharma 2021 | Prospective cohort | 30 March - 30 April 2020 | 256 | Healthcare (Tertiary care hospital) | Screening, testing, isolation, wearing PPE | COVID-19 cases, direct contact with the surfaces which were in contact with the patients | Moderate |  |
|  |  |  |  |  |  |  |  |  |  |
| India | Sundar 2021 | Contact tracing | 1 - 20 August 2020 | 18 | Workplaces (Construction labourers) | Masks | Areas with restricted air circulation | Low |  |
|  |  |  |  |  |  |  |  |  |  |
|  |  |  |  |  | Workplaces (Open environmental construction work contacts) |  | Not reported | Low |  |
|  |  |  |  |  |  |  |  |  |  |
|  |  |  |  |  |  |  |  |  |  |
|  |  |  |  |  | Workplaces (Closed environmental construction work contacts) |  | Not reported | High |  |
|  |  |  |  |  |  |  |  |  |  |
|  |  |  |  |  |  |  |  |  |  |
|  |  |  |  |  |  |  |  |  |  |
| International (11 countries) | Robinson 2021 | Prospective cohort | July 2020-December 2020 | 195 | Sports and activities (Outdoor sports) | Social distancing | Not Reported | Low |  |
|  |  |  |  |  |  |  |  |  |  |
| International (67 countries) | Lentz 2021 | Case-control | 20 April 2020, - 5 May 2020 | 1130 | Healthcare (hospitals) | Institutional policies regarding PPE | Non–aerosol-generating contact with COVID-19 patients | Not Reported |  |
|  |  |  |  |  |  |  |  |  |  |
|  |  |  |  |  | Events and entertainment (gatherings >10, bars/restaurants) |  | Not Reported | Not Reported |  |
|  |  |  |  |  |  |  |  |  |  |
|  |  |  |  |  |  |  |  |  |  |
|  |  |  |  |  | Transportation (Public transportation) |  | Not Reported | Not Reported |  |
|  |  |  |  |  |  |  |  |  |  |
|  |  |  |  |  |  |  |  |  |  |
| Ireland | Heavey 2020 | Case series | Mar-20 | 6 | Education (Primary school (1 case) and Secondary school (2 cases)) | Restrict movements and active surveillance | Close contact, outside school environment | Low |  |
|  |  |  |  |  |  |  |  |  |  |
|  |  |  |  |  |  |  |  |  |  |
| Israel | Natapov 2021 | Prospective cohort | May 2020-September 2020 | 1,871,356 patients; 14,825 dental staff members | Specialized services (Dental clinic) | PPE | Not Reported | Low |  |
|  |  |  |  |  |  |  |  |  |  |
| Italy | Calvani 2021 | case-control | October 2020-December 2020 | 162 | Education (Nursery, primary, secondary, high school) | Preventive measures; face masks; using a different towel/room/bedroom/table; hand hygiene; environmental hygiene | Not reported | Low |  |
|  |  |  |  |  |  |  |  |  |  |
| Italy | Cattelan 2020 | prospective cohort | February 2020 - April 2020 | 60 | Healthcare (Advanced Triage of the Infectious Diseases Unit) | Infection control-integrated surveillance system implemented to prevent and monitor infection transmission among HCWs; 1. The predisposition of a fast triage prior to entering the hospital; 2. The creation of separated and dedicated areas to avoid the interaction between potentially infected and non-infected patients; 3. The predisposition of multiple installations for hand disinfection; 4. The application of strict requirements for PPE usage and the implementation of training protocols directed to HCWs; 5. The implementation of an integrated surveillance system to prevent and monitor infection transmission among HCWs. | High number of patients visiting per day, high proportion of performed positive nasopharyngeal swabs | Low |  |
|  |  |  |  |  |  |  |  |  |  |
|  |  |  |  |  |  |  |  |  |  |
| Italy | Gianola 2021 | Cross-sectional | April 2020 - May 2020 | 15,566 | Healthcare (Physical therapists working in inpatient and outpatient care) | Availability of NPS testing, use of PPE, quarantine period, and readmission to work | Unavailability of PPE, relocating or changing job tasks | Unclear |  |
|  |  |  |  |  |  |  |  |  |  |
| Italy | Larosa 2020 | Surveillance | 1 September 2020 - 15 October 2020 | 1,248 individuals (209 teachers/staff and 1,039 children) | Education (Infant-toddler centres (age 0–3 years), preschools (age 3–5 years), elementary schools (age 6–10 years), middle schools (age 11–13 years) and high schools (age 14–19 years) | Masking, physical distancing | Physical classroom does not promote distancing, masks not worn | Low/moderate: for preschools and elementary schools, moderate for secondary schools |  |
|  |  |  |  |  |  |  |  |  |  |
|  |  |  |  |  | Education (pre-school, elementary, secondary schools) |  | Household contact, outside school transmission | Low (in-school transmission) |  |
|  |  |  |  |  |  |  |  |  |  |
|  |  |  |  |  |  |  |  |  |  |
| Italy | Squeri 2021 | Surveillance | 9 March 2020 - 19 June 2020 | 17 HCW cases | Healthcare (University Hospital) | PPE, hand sanitization, Self-isolation | Intra-hospital and intra-family transmission, return from the high COVID-19 risk areas | High |  |
|  |  |  |  |  |  |  |  |  |  |
|  |  |  |  |  | Household (Own home) |  | Close contact, family members | High |  |
|  |  |  |  |  | Transportation |  | Return from high-risk areas before the epidemic onset in Italy | High |  |
| Japan | Akaishi 2021 | Cross-sectional | July 2020-May 2021 | 4550 | Household (Dormitories) | Masking, disinfection, accessibility of alcohol pumps | Not Reported | High |  |
|  |  |  |  |  |  |  |  |  |  |
|  |  |  |  |  |  |  |  |  |  |
| Japan | Nakajo 2021 | Contact tracing | January 2020 - March 2020 | 36 confirmed cases (from the earliest observed cluster) | Events and entertainment (Night party) | Not Reported | Close contact with a tourist from Wuhan | High |  |
|  |  |  |  |  |  |  |  |  |  |
| Japan | Ogata 2021 | Cohort study | Nov-20 | 160 | Household (household) | Not Reported | Not reported | Not reported |  |
|  |  |  |  |  |  |  |  |  |  |
|  |  |  |  |  | Healthcare (Long term care) |  | Not reported | Not reported |  |
|  |  |  |  |  |  |  |  |  |  |
|  |  |  |  |  | Hospitality (Restaurants) |  | Not reported | Not reported |  |
|  |  |  |  |  |  |  |  |  |  |
|  |  |  |  |  | Workplaces |  | Not reported | Not reported |  |
|  |  |  |  |  |  |  |  |  |  |
| Japan | Toyokawa 2021 | Cohort study | 23-Mar-20 | 146 | Transportation (Domestic flight) | Masks | Non-use of face masks | Not Reported |  |
|  |  |  |  |  |  |  |  |  |  |
| Japan | Wada 2020 | Prospective cohort | 1 June 2020 - 31 July 2020 | 207 cases | Education (Elementary school (ages 6-12) and Junior high school (ages 13-15)) | Physical distancing, wearing face masks, frequent hand washing | Close contact | Low |  |
|  |  |  |  |  |  |  |  |  |  |
| Korea | Jung 2021 | Prospective cohort | March 2020 - March 2021 | 36 secondary cases from 14 SARS-cov-2 infected individuals | Healthcare (Tertiary care hospital in Korea) | Masking, PPE | Close contact or non close contact (short talk, sharing space, entering space after index case had left) | Not Reported |  |
|  |  |  |  |  |  |  |  |  |  |
|  |  |  |  |  |  |  | Close contact with infected COVID-19 hcws in hospital staff cafeterias | Low |  |
|  |  |  |  |  |  |  |  |  |  |
| Luxembourg | Mossong 2021 | Retrospective cohort | March 2020 – July 2020 | 101817 | Education (Primary & secondary schools) | Face masks; distancing; screening | Family or household was the most frequently observed setting of sources of infection (42.5%), followed by school (11.6%) as described hereafter. | Not reported |  |
|  |  |  |  |  |  |  |  |  |  |
| Malaysia | Ng 2021 | Retrospective cohort | February 2020 - December 2020 | 261 children (53 isolated with uninfected 47 guardians) | Healthcare (hospitals) | Surgical masks, hand hygiene, avoid sharing feeding utensils | Not Reported | Low |  |
|  |  |  |  |  |  |  |  |  |  |
| New Zealand | James 2021 | Data linkage | February 2020-March 2020 | 1499 confirmed cases (population of NZ) | Education (High school) | Border control and social distancing | Not Reported | High |  |
|  |  |  |  |  |  |  |  |  |  |
|  |  |  |  |  | Events and entertainment (Wedding, Hospitality venues) |  |  | High |  |
|  |  |  |  |  |  |  |  |  |  |
|  |  |  |  |  | Healthcare (Long term care) |  |  | High |  |
|  |  |  |  |  |  |  |  |  |  |
|  |  |  |  |  | Business (Conference) |  |  | High |  |
|  |  |  |  |  |  |  |  |  |  |
|  |  |  |  |  |  |  |  |  |  |
| Poland | Sierpinski 2021 | Cross-sectional | 17 – 18 April 2020 | 2122 | Healthcare (Healthcare facilities) | PPE | Work-related infections | Not Reported |  |
|  |  |  |  |  |  |  |  |  |  |
|  |  |  |  |  |  |  |  |  |  |
|  |  |  |  |  | Workplaces (Large companies (>100 employees)) |  | Occupationally active and became infected at work | Not Reported |  |
|  |  |  |  |  |  |  |  |  |  |
|  |  |  |  |  |  |  |  |  |  |
|  |  |  |  |  | Transportation (drivers) |  | Not reported | Not Reported |  |
|  |  |  |  |  |  |  |  |  |  |
| Qatar | Yorck 2021 | Cohort study | 8 June 2020 - 2 September 2020 | 1337 | Sports and activities (Qatar soccer league) | Temperature checks, social distancing, wearing a mask outside training and matches, and frequent hand hygiene | Social contact with friends, family or social events | Low |  |
|  |  |  |  |  |  |  |  |  |  |
| Saudi Arabia | Barry 2021 | Retrospective cohort | 2 March 2020 - 31 December 2020 | 4462 | Healthcare (hospital) | Not Reported | Regular close contract | High |  |
|  |  |  |  |  |  |  |  |  |  |
| Singapore | Ng 2020 | Retrospective cohort | January 2020-April 2020 | 1114 index cases; 7770 close contacts | Transportation (Sharing a vehicle with an index case) | Masking | Not Reported | High |  |
|  |  |  |  |  |  |  |  |  |  |
|  |  |  |  |  | Events and entertainment (Being spoken to by an index case for 30 min or longer) |  | Not Reported | High |  |
|  |  |  |  |  |  |  |  |  |  |
|  |  |  |  |  |  |  |  |  |  |
| Singapore | Wong 2021 | Cross-sectional | 23 January 2020 17 April 2020 | 88 | Healthcare (Hospital/workplace) | PPE, IPC strategies | Close contact with the infected patients, transmission from other infected hcws, community (social interactions, religions gatherings) | High |  |
|  |  |  |  |  |  |  |  |  |  |
| South Korea | Jung 2021 | Contact tracing | January 2020 - September 2021 | 33 (Index patients) | Healthcare | Masking, PPE | Close contact with infected COVID-19 hcws in hospital staff cafeterias | Low (side-by-side eating and in the absence of conversation) |  |
| South Korea | Kang 2020 | Contact tracing | 30 April 2020 - 6 May 2020 | 41612 tests | Events and entertainment (Social settings (Night clubs)) | Not reported | Reopening of night club, night club visitors | High |  |
|  |  |  |  |  |  |  |  |  |  |
| Spain | Alonso 2021 | Retrospective cohort | September 2020 - December 2020 | 1.09 million students | Education (Primary and middle school) | Isolation | Age | Not reported |  |
|  |  |  |  |  |  |  |  |  |  |
|  |  |  |  |  | Education (Preschool to high school) |  | Not reported | Low |  |
|  |  |  |  |  |  |  |  |  |  |
| Spain | Aranaz-Andres 2021 | Cross-sectional | February 2020 - April 2020 | 323 | Healthcare (Tertiary hospital) | Changes of uniform, sanitation of personal objects before the workday, disinfection of shared material | Public transport, carrying out aerosol generating procedures, and belonging to one of the specialties | High |  |
|  |  |  |  |  |  |  |  |  |  |
| Spain | Domènech-montoliu 2021 | Retrospective cohort | May 2020 -June 2020 | 1338 | Events and entertainment (Mass gathering events (community dinner; gala dinner; fireworks; dance; awards gala; queen's offering)) | Not reported | Not reported | High |  |
|  |  |  |  |  |  |  |  |  |  |
| Spain | Jordan 2021 | Prospective cohort | June 2020-July 2020 | 39 index cases (253 contacts) | Sports and activities (Summer camp) | Bubble groups, hand washing, facemasks and conducting activities mostly outdoors | Not reported | Low |  |
|  |  |  |  |  |  |  |  |  |  |
|  |  |  |  |  | Education (Children aged 3–15 years and staff of any age (≥16 years)) |  |  | Low |  |
|  |  |  |  |  |  |  |  |  |  |
|  |  |  |  |  |  |  |  |  |  |
| Spain | Mendez-echevarria 2021 | Cross-sectional | March 2020 - May 2020 | 69 HCW, 113 children | Healthcare (Households of HCW (staff to child)) | Not reported | Household family transmission | High |  |
|  |  |  |  |  |  |  |  |  |  |
| Sweden | Froberg 2021 | Prospective cohort | March 2020 - June 2020 | 839 | Healthcare (22 primary care centers, 2 care homes, and one palliative care unit) | In March to June 2020, in Sweden, protective measures for healthcare workers in Sweden were limited: postponing meetings that could wait, keeping a safe distance, when possible, limit the time of close contact with care takers and to wear a surgical mask, a gown and gloves—but only if the patient had symptoms suggestive of COVID-19. Testing and contact tracing of patients with possible COVID-19 was not available for these settings during the first wave. | Age, sex | High (young HCW women (ages 20-29)) |  |
| Sweden | Lidstrom 2020 | Cross-sectional | May 2020 -- June 2020 | 8679 | Healthcare (Inpatient care | Not reported | The age-dependent association likely represents two factors, both a higher level of community transmission among the young, and a correlation between age and level of physical contact with patients during inpatient care, which relates to differences in length of training and the pyramid-shaped age-related hierarchy among different categories of clinical staff. | High |  |
|  |  |  |  |  |  |  |  |  |  |
|  |  |  |  |  |  |  |  |  |  |
|  |  |  |  |  |  |  |  |  |  |
|  |  |  |  |  | Outpatient care |  | Not Reported | Not Reported |  |
|  |  |  |  |  |  |  |  |  |  |
|  |  |  |  |  | Primary health care |  | Not Reported | Not Reported |  |
|  |  |  |  |  |  |  |  |  |  |
|  |  |  |  |  | Covid-19 specific care Covid-19 possible unit) |  | Not Reported | Not Reported |  |
|  |  |  |  |  |  |  |  |  |  |
| Sweden | Nygren 2021 | Cross-sectional | 8 September 2020 - 10 November 2020 | Uni employees: 192   hcws: 271 | Healthcare (University Hospital) | PPE, testing, additional protective measures (plexiglass and hand washing) | Direct contact with COVID-19 patients, working in a COVID-19-unit, exposure with colleagues who tested COVID-19 positive | Not Reported |  |
|  |  |  |  |  |  |  |  |  |  |
|  |  |  |  |  | Household (Household (hcws and University employees)) |  | Confirmed/suspected COVID-19 case in the same household. |  |  |
|  |  |  |  |  |  |  |  |  |  |
|  |  |  |  |  |  |  |  |  |  |
|  |  |  |  |  |  |  |  |  |  |
|  |  |  |  |  | Transportation (Public transportation/International travel (hcws and University employees)) |  | Viral transmission while using public transport to work/International travel |  |  |
| Switzerland | Ulyte 2021 | Prospective cohort | June 2020-November 2020 | 2603 children (275 classes in 55 schools) | Education (Lower school level (grades 1 and 2; children aged 6-9 years); middle school level (grades 4 and 5; children aged 9-13 years); and upper school level (grades 7 and 8; children aged 12-16 years)) | Masks, physical distancing, no mixing of classes, reduction of large groups, requirement to stay home when not feeling well | Not reported | Low |  |
| Taiwan | Huang 2021 | Surveillance | Not Reported | 127 HCW, 57 in-patients admitted to ward 5C, 27 accompanying persons, 25 environmental samples from the emergency room | Healthcare (Tertiary care hospital in Taiwan) | Mask, hospital staff screening (listed as Index case's close contact), restricting visitors, reinforcement to adhere to IPC measures, environmental cleansing and disinfection, quarantine for the close contacts | Close contact, direct care (multiple modes of transmission) | Not Reported |  |
| Taiwan | Su 2021 | Cohort study | 14 January 2020 7 March 2020 | 6 patients (138 hcws contacts) | Healthcare (Hospital) | PPE, masks | Close contact with the COVID-19 diagnosed patients, PPE use (surgical mask, N95), contact time with the patients | High |  |
| Thailand | Atsawawaranunt 2021 | Surveillance | Not reported | Not reported | Healthcare (Quarantine facility and hospital) | Masks, PPE | Infected quarantined individuals, close contact | High |  |
| Turkey | Aydin 2021 | Retrospective cohort | 20 March 2020 - 20 May 2020 | 128 | Healthcare (Research and training hospital) | PPE | Close contact with the infected patients or other HCWs | High |  |
| Turkey | Celebi 2020 | case-control | March 2020 - May 2020 | 703 | Healthcare (Teaching and tertiary care hospital) | PPE, masking, social distancing | Presence of a SARS-CoV-2 positive person in the household, inappropriate use of PPE, staying in the same personnel break room as an HCW without a medical mask for more than 15 minutes, consuming food within 1 m of an HCW, and failure to keep a safe social distance from an HCW with COVID-19 infection | High |  |
| Turkey | Guner 2021 | Contact tracing | Not Reported | 23 | Transportation (International travel) | Border control | Family, workplace, hospital contacts | Not Reported |  |
| Turkey | Pinarlik 2021 | Cohort study | May 2020 December 2020 | 1732 HCWs | Healthcare (Hospital) | PPE, masks | Close contact with the probable/diagnosed COVID-19 patient, inappropriate PPE use | High |  |
|  |  |  |  |  |  |  |  |  |  |
|  |  |  |  |  |  |  |  |  |  |
|  |  |  |  |  | Household (household) |  | Diagnosed COVID-19 patient in household, larger household size | High |  |
|  |  |  |  |  |  |  |  |  |  |
| UK, London | Tang 2021 | Prospective cohort | 18 April – 27 April 2020 | 4 care homes, 359 permanent staff and residents | Healthcare (Long-term care facilities) | enhanced cleaning, closing to visitors, isolating residents in single rooms where possible, and restricting use of shared spaces | Resident profile, staff occupational risk factors such as working across different care homes, compliance with recommended IPC measures | High |  |
|  |  |  |  |  |  |  |  |  |  |
| UK, Wales | Thompson 2021 | Data linkage | August 2020-December 2020 | 464531 | Education | Unclear: "Strong mitigation measures over the whole of the study period may have reduced wider spread within the school environment." | Not reported | Not Reported |  |
| UK, England | Ben 2021 | Contact Tracing | 1 July 2020 - 4 October 2020 | 8 index participants (28 contacts) | Sports and activities (Rugby league match) | Isolation | Player to player interactions whilst infectious with SARS-CoV-2 | Low |  |
| UK, England | Blomquist 2021 | Retrospective cohort | January 2020-March 2020 | 2368 (18 flights) | Transportation (Airplane) | Post-flight quarantine | Not reported | Low |  |
| UK, England | Ismail 2021 | Cross-sectional | June 2020-July 2020 | 928 000 (median attendance) | Education (Early, primary, secondary) | Not Reported | Not Reported | Low |  |
|  |  |  |  |  |  |  |  |  |  |
| UK, England | Ladhani 2021 | Prospective cohort | June 2020-December 2020 | 11 966 | Education (Primary schools) | Physical distancing, reduced class sizes, bubbles | Not Reported | Low |  |
|  |  |  |  |  |  |  |  |  |  |
| UK, England | Leeman 2021 | Retrospective cohort | 6 March 2020 - 3 May 2020 | 153 | Healthcare (hospital) | PPE | Working with COVID19 infected patients. And inadequate spacing between beds | Not Reported |  |
|  |  |  |  |  |  |  |  |  |  |
| UK, England | Wenlock 2021 | Prospective cohort | October 2020 - December 2020 | 575 | Healthcare (University hospital) | Testing, wearing PPE, isolation | Sharing the same bay and time exposed | High |  |
|  |  |  |  |  |  |  |  |  |  |
|  |  |  |  |  |  |  |  |  |  |
| UK, England, and Wales | Susan 2021 | Cohort study | Two behavioural surveys (December – October 2020 and February 2021) | 10,858 | Transportation (Public transportation) | Unspecified restrictions and non-pharmaceutical interventions | Using public transport for work or other chores (shopping) | High |  |
|  |  |  |  |  |  |  |  |  |  |
|  |  |  |  |  |  |  |  |  |  |
| USA | Baker 2021 | Cohort study | March 2020 - June 2020 | 226 | Healthcare (patients; Academic medical center) | Masking, PPE | Healthcare workers diagnosed with COVID-19 | Low |  |
|  |  |  |  |  |  |  |  |  |  |
|  |  |  |  |  | Healthcare (community; Academic medical hospital) |  | Patient exposures to infected HCWs in different settings (inpatient, emergency, outpatient) | Low |  |
| USA | Bjorkman 2021 | Retrospective cohort | 17 August 2020 – 25 November 2020 | 6408 | Accommodations (Residence Hall at the University of Colorado Boulder) | Self-isolation | Household contact | Low |  |
|  |  |  |  |  |  |  |  |  |  |
| USA | Burke 2020 | Contact Tracing | 19 January 2020 - 30 January 2020 | 338 | Household (Households of case patients) | Masks, PPE | Family, friends | High |  |
|  |  |  |  |  |  |  |  |  |  |
|  |  |  |  |  | Healthcare (Hospitals, clinics) |  | Close contact, direct care | High |  |
|  |  |  |  |  |  |  |  |  |  |
|  |  |  |  |  |  |  |  |  |  |
|  |  |  |  |  | Healthcare (Hospitals, clinics) |  | Face to face, direct physical contact, rode in same vehicle, was in same room | Not Reported |  |
|  |  |  |  |  |  |  |  |  |  |
|  |  |  |  |  | Other (workplace/flight contact/rideshare) |  | face to face, direct physical contact, rode in same vehicle, was in same room | Not Reported |  |
|  |  |  |  |  |  |  |  |  |  |
| USA | Currie 2021 | Surveillance | Fall 2020 | 3730 | Accommodations (University residence) | Mask usage at all times (except within students’ own rooms), physical distancing, when possible, self-monitoring for symptoms, and limited gatherings in accordance with local public health guidelines | Living with a roommate (Viral transmission) | Not reported |  |
| USA | D’Agostino 2021 | Retrospective cohort | March 2020-August 2020 | 5344 youth, 1486 staff | Sports and activities (Indoor, outdoor camp) | Screening, daily temperature checks, masks, hand hygiene, physical distancing, small cohorts, scheduled site cleanings, and staff COVID-19 education and workplace training | - | Low |  |
| USA | Denny 2021 | Surveillance | 2 August 2020 - 11 October 2020 | 10,265 | Education (University campus) | Facemask, social distancing, PPE, quarantine | Close contact (off-campus housing) | High |  |
| USA | Dimcheff 2021 | Serologic survey | 8 Jun 2020 – 8 Jul 2020 | 1476 | Healthcare (Tertiary-care referral facility within the VA healthcare system) | Facility-wide quality improvement and infection prevention initiative | Not Reported | High |  |
| USA | Drezner 2020 | Surveillance | 29 June 2020, - 9 August 2020 | Not Reported | Sports and activities (Youth Soccer club) | Mask, physical distancing, hand sanitizer, no group gatherings, quarantine | Close contact (other athletes, family members) | Not Reported |  |
| USA | Drogosz 2021 | Surveillance | 5 July 2020 to 13 September 2020 and 14 September 2021to 7 March 2021 | Not Reported | Education (Middle school and high school) | Quarantine measures for infected participants | Close contact while playing sports. | High |  |
|  |  |  |  |  |  |  |  |  |  |
|  |  |  |  |  | Sports and activities (Non school settings) |  | Not Reported | High |  |
|  |  |  |  |  |  |  |  |  |  |
|  |  |  |  |  |  |  |  |  |  |
| USA | Edward 2021 | Prospective cohort | January 2021 - March 2021 | 468 | Education (K-8 private schools) | Masking, distancing, testing, contact tracing, quarantine | In-school transmission | Low |  |
|  |  |  |  |  |  |  |  |  |  |
| USA | Falk 2021 | Surveillance | 31 August 2020 - 29 November 2020 | 17 schools (5530 students and staff members) | Education (K-12 schools) | Mask, social distancing, quarantine | Asymptomatic transmission within school settings | Low |  |
|  |  |  |  |  |  |  |  |  |  |
| USA | Gettings 2021 | Prospective cohort | December 2020-January 2021 | 86 index cases (1119 contacts, 59 positive included) | Education (Elementary, middle, high school) | Physical distancing, masking, handwashing, facility cleaning/disinfection, hybrid education, improved ventilation | Not Reported | Moderate |  |
| USA | Heinzerling 2020 | Cohort study | Feb-20 | 121 Cohort size (43 exposed) | Healthcare (Hospital) | Facemask, gloves, eye protection, gown | Close contact while confirmed COVID-19 patient, inadequate PPE use | High |  |
|  |  |  |  |  |  |  |  |  |  |
| USA | Lan 2021 | Retrospective cohort | Up to 3 June 2020 | 5,177 HCW (152 positive) | Healthcare (Community healthcare system) | Universal Masking | Front facing HCW, residential area’s cumulative attack rate, job risk | Low |  |
|  |  |  |  |  |  |  |  |  |  |
| USA | Lan 2020 | Cross-sectional | May-20 | 104 | Workplaces (a single grocery retail store) | Ability to practice social distancing, gloves, facemask | Direct exposure with customer, self-reported exposure SARS-cov-2 patients, inability to practice social distancing at store, commuting to work | High |  |
|  |  |  |  |  |  |  |  |  |  |
| USA | Maccannell 2021 | Surveillance | 18 March - 31 July 2020 | Not Reported | Specialized services (Long term care facilities) | Not reported | Close contact, direct care | High |  |
|  |  |  |  |  |  |  |  |  |  |
| USA | Paul 2021 | Cross-sectional | 1 September - 13 September 2020 | 159 | Sports and activities (Curling facilities) | NA | Curling tournaments with large attendance | High |  |
|  |  |  |  |  |  |  |  |  |  |
| USA | Porter 2021 | Surveillance | March 2020 - October 2020 | 677 | Workplaces (Workers in the seafood processing industry) | Social distancing, quarantine, post travel entry quarantine, midseason transfers, serial testing, daily symptom screening, notifying public health of a positive worker | Person-to-person transmission within an entry quarantine group | High |  |
|  |  |  |  |  |  |  |  |  |  |
| USA | Ramirez 2021 | Surveillance | 24 August 2020 - 19 March 2021 | 1154 (grades 1 to 12) | Transportation (School buses) | Mask, social distancing | School transportation and viral transmission | Low |  |
|  |  |  |  |  |  |  |  |  |  |
| USA | Thadhani 2021 | Case-control | February 2020 - June 2020 | 170,234 | Healthcare (Dialysis centre) | Screening, testing, isolation, wearing a mask, PPE | Shared chairs | Low |  |
|  |  |  |  |  |  |  |  |  |  |
| USA | Valesano 2021 | Surveillance | 16 August 2020 - 24 November 2020 | 1659 | Education (University) | NA | Transmission clusters | Unclear |  |
|  |  |  |  |  |  |  |  |  |  |
| USA | Vang 2021 | Cohort study | 21 August 2020 - 5 September 2020 | 965 cases (54 gatherings linked to the contacts) | Accommodations (On-campus and off-campus university congregate settings) | Mask, social distancing, hand hygiene | Social gatherings associated with fraternities or sororities | Not reported |  |
|  |  |  |  |  |  |  |  |  |  |
| USA | Whaley 2021 | Cross-sectional | 1 January 2020 8 November 2020 | 6 535 987 | Events and entertainment (Social gatherings (birthdays)) | Masking, physical distancing | Close contact corresponding with social gathering and celebrations in different households | High |  |
|  |  |  |  |  |  |  |  |  |  |
| USA | Zabarsky 2021 | Prospective cohort | 15 March 2020 - 15 July 2020 | 1534 personnel (with nasopharyngeal swab collection) | Healthcare (Medical center) | Facemasks | Higher exposure at work ((non-patient care settings like nursing stations, staff work and break rooms during meals), shorter duration contacts within 6 feet)), higher exposure to infected family members or other individuals in the community | High |  |
|  |  |  |  |  |  |  |  |  |  |
| USA | Zimmerman 2021 | Prospective cohort | October 2020-February 2021 | 185477 | Education (Primary & secondary schools, Elementary school, Middle school, High school) | Physical distancing, masking, handwashing, hybrid education | Not reported | Low |  |
|  |  |  |  |  |  |  |  |  |  |
|  |  |  |  |  |  |  |  |  |  |
|  |  |  |  |  |  |  |  |  |  |
|  |  |  |  |  |  |  |  |  |  |
| USA | Zimmerman 2021 | Prospective cohort | August 2020-October 2020 | >90 000 students and staff (773 cases) | Education | Masking, distancing, hand hygiene, symptom monitoring, temperature check | Not reported | Low |  |
| Vietnam | Le 2021 | Surveillance | December2019 - February 2020 | 12 patients (2 clusters) | Household | Not reported | Travel from the endemic region | Not Reported |  |
