# Supplementary material for: SARS-CoV-2 transmission risk for common group activities and settings: a living scoping review
Source: Eur J Public Health. 2023 Nov 23;34(1):196–201. doi: 10.1093/eurpub/ckad195 (PMC10843946; doi:10.1093/eurpub/ckad195)
Supplement: ckad195_Supplementary_Data [file ckad195_supplementary_data.zip › ckad195_Supplementary_Data/ejph-2023-07-om-0370-File009.docx]

# **Appendix 6. List of Excluded studies**

# **Excluded study designs:**

**Abbas, M.,Nunes, T. R.,Cori, A.,Cordey, S.,Laubscher, F.,Baggio, S.,Jombart, T.,Iten, A.,Vieux, L.,Teixeira, D.,Perez, M.,Pittet, D.,Frangos, E.,Graf, C. E.,Zingg, W.,Harbarth, S.**. Explosive nosocomial outbreak of SARS-CoV-2 in a rehabilitation clinic: the limits of genomics for outbreak reconstruction. *Journal of Hospital Infection.* 2021. 27:27

**Ahmadzadeh, M.,Farokhi, E.,Shams, M.**. Investigating the effect of air conditioning on the distribution and transmission of COVID-19 virus particles. *Journal of Cleaner Production.* 2021. 316:128147

**Alaidroos, A.,Almaimani, A.,Baik, A.,Al-Amodi, M.,Rahaman, K. R.**. Are Historical Buildings More Adaptive to Minimize the Risks of Airborne Transmission of Viruses and Public Health? A Study of the Hazzazi House in Jeddah (Saudi Arabia). *International Journal of Environmental Research & Public Health [Electronic Resource].* 2021. 18:30

**Alessandro, Zivelonghi, Massimo, Lai**. Minimizing cumulative risk to control airborne transmission of SARS-Cov-2 in schools. *#journal#.* 2020. #volume#:#pages#

**Ambatipudi, M.,Carrillo Gonzalez, P.,Tasnim, K.,Daigle, J. T.,Kulyk, T.,Jeffreys, N.,Sule, N.,Trevino, R.,He, E. M.,Mooney, D. J.,Koh, E.**. Risk quantification for SARS-CoV-2 infection through airborne transmission in university settings. *Journal of Occupational & Environmental Hygiene.* 2021. #volume#:1-19

**Arav, Y.,Klausner, Z.,Fattal, E.**. Theoretical investigation of pre-symptomatic SARS-CoV-2 person-to-person transmission in households. *Scientific Reports.* 2021. 11:14488

**Arinaminpathy, N.,Das, J.,McCormick, T. H.,Mukhopadhyay, P.,Sircar, N.**. Quantifying heterogeneity in SARS-CoV-2 transmission during the lockdown in India. *Epidemics.* 2021. 36 (no pagination):#pages#

**Arino, J.,Boelle, P. Y.,Milliken, E.,Portet, S.**. Risk of COVID-19 variant importation - How useful are travel control measures?. *Infectious Disease Modelling.* 2021. 6:875-897

**Ashcroft, P.,Lehtinen, S.,Angst, D. C.,Low, N.,Bonhoeffer, S.**. Quantifying the impact of quarantine duration on COVID-19 transmission. *eLife.* 2021. 10:05

**Augenbraun, B. L.,Lasner, Z. D.,Mitra, D.,Prabhu, S.,Raval, S.,Sawaoka, H.,Doyle, J. M.**. Assessment and mitigation of aerosol airborne SARS-CoV-2 transmission in laboratory and office environments. *Journal of Occupational & Environmental Hygiene.* 2020. 17:447-456

**Azimi, P.,Keshavarz, Z.,Cedeno Laurent, J. G.,Stephens, B.,Allen, J. G.**. Mechanistic transmission modeling of COVID-19 on the Diamond Princess cruise ship demonstrates the importance of aerosol transmission. *Proceedings of the National Academy of Sciences of the United States of America.* 2021. 118:23

**Abe, T., Watanabe, H., Iriyama, H., Komori, A., Kainoh, T., Inaba, K., Narahashi, K., Tokuda, Y.**. Three-pronged strategy for COVID-19 prevention in medical personnel and patients in a hospital setting during an outbreak. *Intensive Care Medicine Experimental. Conference: European Society of Intensive Care Medicine Annual Congress, ESICM.* 2021. 9:#pages#

**Adam, D. C.,Wu, P.,Wong, J. Y.,Lau, E. H. Y.,Tsang, T. K.,Cauchemez, S.,Leung, G. M.,Cowling, B. J.**. Clustering and superspreading potential of SARS-CoV-2 infections in Hong Kong. *Nature Medicine.* 2020. 26:1714-1719

**Adrizain, R.,Sari, N.**. Corona virus disease 2019 (Covid-19) incidence in pediatric oncology patient: Does routine screening affect the risk for the transmission in the hospital. *Journal of the Pediatric Infectious Diseases Society.* 2021. 10(SUPPL 2):S18

**Agne, Ulyte, Thomas, Radtke, Irene, Abela, Sarah, H. Haile, Julia, Braun, Ruedi, Jung, Christoph, Berger, Alexandra, Trkola, Jan, Fehr, Milo, A. Puhan, Susi, Kriemler**. Seroprevalence and immunity of SARS-CoV-2 infection in children and adolescents in schools in Switzerland: design for a longitudinal, school-based prospective cohort study. *#journal#.* 2020. #volume#:#pages#

**Agut, H.**. [Covid-19, september 2020: fall storm warning]. *Virologie (Montrouge).* 2020. 24:361-367

**Akinbami, L. J.,Vuong, N.,Petersen, L. R.,Sami, S.,Patel, A.,Lukacs, S. L.,Mackey, L.,Grohskopf, L. A.,Shehu, A.,Atas, J.**. SARS-CoV-2 seroprevalence among healthcare, first response, and public safety personnel, detroit metropolitan area, Michigan, USA, May-June 2020. *Emerging Infectious Diseases.* 2020. 26(12):2863-2871

**Amato, L.,Jurisic, L.,Puglia, I.,Di Lollo, V.,Curini, V.,Torzi, G.,Di Girolamo, A.,Mangone, I.,Mancinelli, A.,Decaro, N.,Calistri, P.,Di Giallonardo, F.,Lorusso, A.,D'Alterio, N.**. Multiple detection and spread of novel strains of the SARS-CoV-2 B.1.177 (B.1.177.75) lineage that test negative by a commercially available nucleocapsid gene real-time RT-PCR. *Emerging Microbes & Infections.* 2021. 10:1148-1155

**Anand, S.,Mayya, Y. S.**. Size distribution of virus laden droplets from expiratory ejecta of infected subjects. *Scientific Reports.* 2020. 10:21174

**Andrade, L. A.,Gomes, D. S.,Goes, M. A. O.,Souza, M. S. F.,Teixeira, D. C. P.,Ribeiro, C. J. N.,Alves, J. A. B.,Araujo, Kcgm,Santos, A. D. D.**. Surveillance of the first cases of COVID-19 in Sergipe using a prospective spatiotemporal analysis: the spatial dispersion and its public health implications. *Revista Da Sociedade Brasileira de Medicina Tropical.* 2020. 53:e20200287

**Anne-Mari Gjestvang, Moe, Mina, Eriksen, Tiril, Schjølberg, Fred, Haugen**. SARS-CoV-2 serological findings and exposure risk among employees in school and retail after first and second wave COVID-19 pandemic in Oslo, Norway: a cohort study. *#journal#.* 2021. #volume#:#pages#

**Anonymous,**. CORONAVIRUS: Two-way transmission on mink farms. *Science.* 2021. 371(6525):139B-139C

**Areekal, B.,Vijayan, S. M.,Suseela, M. S.,Andrews, M. A.,Ravi, R. K.,Sukumaran, S. T.,Jose, R.,Edappanatt, F. T. T.**. Risk factors, epidemiological and clinical outcome of close contacts of covid-19 cases in a tertiary hospital in southern india. *Journal of Clinical and Diagnostic Research.* 2021. 15(3):LC34-LC37

**Arshad, M.,Otero, S.,Mithal, L. B.**. Transmission of SARS-CoV-2 to household members of COVID positive healthcare workers. *Open Forum Infectious Diseases.* 2020. 7(SUPPL 1):S317-S318

**Assob-Nguedia, J. C.,Dongo, D.,Nguimkeu, P. E.**. Early dynamics of transmission and projections of COVID-19 in some West African countries. *Infectious Disease Modelling.* 2020. 5:839-847

**Atherstone, C.,Peterson, M. L.,Malone, M.,Honein, M. A.,MacNeil, A.,O'Neal, C. S.,Paul, S.,Harmon, K. G.,Goerl, K.,Wolfe, C. R.,Casani, J.,Barrios, L. C.,Covid- Collegiate Athlete Testing Group**. Time from Start of Quarantine to SARS-CoV-2 Positive Test Among Quarantined College and University Athletes - 17 States, June-October 2020. *MMWR - Morbidity & Mortality Weekly Report.* 2021. 70:7-11

**Avwioro, G.,Egwunyenga, A.,Adjekuko, C.,Mgbere, O.,Odibo, E.,Iyiola, S.,Enitan, S. S.,Essien, E. J.**. Commercial sex work during novel coronavirus (SARS-CoV-2) era in the niger delta region: Relationships between knowledge, preventive practice, and transmission potential. *International Journal of Women's Health.* 2021. 13:509-523

**Backer, J. A.,Mollema, L.,Vos, E. R.,Klinkenberg, D.,Van Der Klis, F. R.,De Melker, H. E.,Van Den Hof, S.,Wallinga, J.**. Impact of physical distancing measures against COVID-19 on contacts and mixing patterns: Repeated cross-sectional surveys, the Netherlands, 2016-17, April 2020 and June 2020. *Eurosurveillance.* 2021. 26(8) (no pagination):#pages#

**Bakamutumaho, B.,Cummings, M. J.,Owor, N.,Kayiwa, J.,Namulondo, J.,Byaruhanga, T.,Muwanga, M.,Nsereko, C.,Rwamutwe, E.,Mutonyi, R.,Achan, J.,Wanyenze, L.,Ndazarwe, A.,Nakanjako, R.,Natuhwera, R.,Nsangi, A.,Bosa, H. K.,Ocom, F.,O'Donnell, M. R.,Kikaire, B.,Lutwama, J. J.**. Severe COVID-19 in Uganda across Two Epidemic Phases: A Prospective Cohort Study. *The American journal of tropical medicine and hygiene..* 2021. 09:#pages#

**Baker, M. A., Fiumara, K., Rhee, C., Williams, S. A., Tucker, R., Wickner, P., Resnick, A., Klompas, M.**. Low Risk of Coronavirus Disease 2019 (COVID-19) Among Patients Exposed to Infected Healthcare Workers. *Clinical infectious diseases : an official publication of the Infectious Diseases Society of America.* 2021. 73(7):e1878-e1880

**Bellin, E., Elkin, A. H., Stiefel, L., Shteingart, L., Infield, M., Nemetski, S. M.**. Private High School Reopened-COVID Mitigation and Clinical Surveillance Using an Internet Application. *Journal of Public Health Management & Practice.* 2022. 28:36-42

**Berihun, G.,Walle, Z.,Teshome, D.,Berhanu, L.,Abebe, M.,Ademas, A.,Gizeyatu, A.,Keleb, A.,Malede, A.,Atikilt, G.,Teym, A.,Adane, M.**. Knowledge, Attitude, and Preventive Practices Towards COVID-19 Among Students of Ethiopian Higher Education Institutions. *Journal of multidisciplinary healthcare.* 2021. 14:2123-2136

**Bernard Stoecklin, S.,Rolland, P.,Silue, Y.,Mailles, A.,Campese, C.,Simondon, A.,Mechain, M.,Meurice, L.,Nguyen, M.,Bassi, C.,Yamani, E.,Behillil, S.,Ismael, S.,Nguyen, D.,Malvy, D.,Lescure, F. X.,Georges, S.,Lazarus, C.,Tabai, A.,Stempfelet, M.,Enouf, V.,Coignard, B.,Levy-Bruhl, D.,Investigation, Team**. First cases of coronavirus disease 2019 (COVID-19) in France: surveillance, investigations and control measures, January 2020. *Euro Surveillance: Bulletin Europeen sur les Maladies Transmissibles = European Communicable Disease Bulletin.* 2020. 25:02

**Bhatia, R.,Klausner, J.**. Estimating individual risks of COVID-19-associated hospitalization and death using publicly available data. *PLoS ONE [Electronic Resource].* 2020. 15:e0243026

**Bhattacharya, A., Collin, S. M., Stimson, J., Thelwall, S., Nsonwu, O., Gerver, S., Robotham, J., Wilcox, M., Hopkins, S., Hope, R.**. Healthcare-associated COVID-19 in England: A national data linkage study. *Journal of Infection.* 2021. 83(5):565-572

**Bielecki, M.,Zust, R.,Siegrist, D.,Meyerhofer, D.,Crameri, G. A. G.,Stanga, Z.,Stettbacher, A.,Buehrer, T. W.,Deuel, J. W.**. Social Distancing Alters the Clinical Course of COVID-19 in Young Adults: A Comparative Cohort Study. *Clinical Infectious Diseases.* 2021. 72(4):598-603

**Bird, P. W.,Sandhu, K.,Fletcher, O.,Ames, B.,Toovey, O.,Tang, J. W. T.**. Retrospective SARS-CoV-2 IgG screening during the first wave (March-June 2020) of the COVID-19 pandemic in the United Kingdom. *Journal of Medical Virology.* 2021. 93(10):6050-6053

**Borges, V.,Isidro, J.,Cortes-Martins, H.,Duarte, S.,Vieira, L.,Leite, R.,Gordo, I.,Caetano, C. P.,Nunes, B.,Sa, R.,Oliveira, A.,Guiomar, R.,Portuguese network for, Sars-CoV-genomics,Gomes, J. P.**. Massive dissemination of a SARS-CoV-2 Spike Y839 variant in Portugal. *Emerging Microbes & Infections.* 2020. 9:2488-2496

**Burke, R. M.,Midgley, C. M.,Dratch, A.,Fenstersheib, M.,Haupt, T.,Holshue, M.,Ghinai, I.,Jarashow, M. C.,Lo, J.,McPherson, T. D.,Rudman, S.,Scott, S.,Hall, A. J.,Fry, A. M.,Rolfes, M. A.**. Active Monitoring of Persons Exposed to Patients with Confirmed COVID-19 - United States, January-February 2020. *MMWR - Morbidity & Mortality Weekly Report.* 2020. 69:245-246

**Cao, Y.,Cheng, S.**. Impact of COVID-19 outbreak on multi-scale asymmetric spillovers between food and oil prices. *Resources Policy.* 2021. 74:102364

**Capon, A.,Ousta, D.,Ferson, M.,Ingleton, A.,Sheppeard, V.**. A multiple site community outbreak of COVID-19 in Sydney, Australia. *Australian & New Zealand Journal of Public Health.* 2021. 45:129-132

**Carrillo-Diaz, M.,Lacomba-Trejo, L.,Romero-Maroto, M.,Gonzalez-Olmo, M. J.**. Facial self-touching and the propagation of covid-19: The role of gloves in the dental practice. *International Journal of Environmental Research and Public Health.* 2021. 18(13) (no pagination):#pages#

**Carter, R. J.,Rose, D. A.,Sabo, R. T.,Clayton, J.,Steinberg, J.,Anderson, M.,Cdc Covid- Response Team**. Widespread Severe Acute Respiratory Syndrome Coronavirus 2 Transmission Among Attendees at a Large Motorcycle Rally and their Contacts, 30 US Jurisdictions, August-September, 2020. *Clinical Infectious Diseases.* 2021. 73:S106-S109

**Chan, C. H.,Wen, T. H.**. Revisiting the effects of high-speed railway transfers in the early covid-19 cross-province transmission in mainland china. *International Journal of Environmental Research and Public Health.* 2021. 18(12) (no pagination):#pages#

**Chappell, J. G.,Tsoleridis, T.,Clark, G.,Berry, L.,Holmes, N.,Moore, C.,Carlile, M.,Sang, F.,Debebe, B. J.,Wright, V.,Irving, W. L.,Thomson, B. J.,Boswell, T. C. J.,Willingham, I.,Joseph, A.,Smith, W.,Khakh, M.,Fleming, V. M.,Lister, M. M.,Howson-Wells, H. C.,Holmes, E. C.,Loose, M. W.,Ball, J. K.,McClure, C. P.,On Behalf Of The Cog-Uk, Consortium**. Retrospective screening of routine respiratory samples revealed undetected community transmission and missed intervention opportunities for SARS-CoV-2 in the United Kingdom. *Journal of General Virology.* 2021. 102:06

**Chen, C., Nadeau, S. A., Topolsky, I., Manceau, M., Huisman, J. S., Jablonski, K. P., Fuhrmann, L., Dreifuss, D., Jahn, K., Beckmann, C., Redondo, M., Noppen, C., Risch, L., Risch, M., Wohlwend, N., Kas, S., Bodmer, T., Roloff, T., Stange, M., Egli, A., Eckerle, I., Kaiser, L., Denes, R., Feldkamp, M., Nissen, I., Santacroce, N., Burcklen, E., Aquino, C., de Gouvea, A. C., Moccia, M. D., Gruter, S., Sykes, T., Opitz, L., White, G., Neff, L., Popovic, D., Patrignani, A., Tracy, J., Schlapbach, R., Dermitzakis, E. T., Harshman, K., Xenarios, I., Pegeot, H., Cerutti, L., Penet, D., Blin, A., Elies, M., Althaus, C. L., Beisel, C., Beerenwinkel, N., Ackermann, M., Stadler, T.**. Quantification of the spread of SARS-CoV-2 variant B.1.1.7 in Switzerland. *Epidemics.* 2021. 37 (no pagination):#pages#

**Chen, J.,Han, T.,Huang, M.,Yang, Y.,Shang, F.,Zheng, Y.,Zhao, W.,Luo, L.,Han, X.,Lin, A.,Zhao, H.,Gu, Q.,Shi, Y.,Li, J.,Xu, X.,Liu, K.,Deng, Y.,Jia, E.,Cao, Q.**. Clinical characteristics of asymptomatic carriers of novel coronavirus disease 2019: A multi-center study in Jiangsu Province. *Virulence.* 2020. 11:1557-1568

**Chen, Y. C.,Yang, H. P.,Li, H. C.,Huang, P. Y.,Chen, C. L.,Chiu, C. H.**. Features and transmission dynamics of SARS-CoV-2 superspreading events in Taiwan: Implications for effective and sustainable community-centered control. *Pediatrics & Neonatology.* 2021. 62:437-440

**Chen, Y., Keen, C., Sandys, V., Simpson, A.**. The role of environmental assessment in workplace COVID-19 outbreak investigation to understand SARS-COV-2 transmission. *Occupational and Environmental Medicine.* 2021. 78(SUPPL 1):A158

**Cheng, H. M.,Zhao, X.,Lim, W. S.,Tan, B. J. M.,Tey, H. L.**. Factors affecting duration of SARS-Cov-2 viral shedding in mildly symptomatic patients isolated in a community facility. *PLoS ONE.* 2021. 16(9 September) (no pagination):#pages#

**Chrysostomou, A. C.,Vrancken, B.,Koumbaris, G.,Themistokleous, G.,Aristokleous, A.,Masia, C.,Eleftheriou, C.,Ioannou, C.,Stylianou, D. C.,Ioannides, M.,Petrou, P.,Georgiou, V.,Hatziyianni, A.,Lemey, P.,Vandamme, A. M.,Patsalis, P. P.,Kostrikis, L. G.**. A comprehensive molecular epidemiological analysis of sars-cov-2 infection in cyprus from april 2020 to january 2021: Evidence of a highly polyphyletic and evolving epidemic. *Viruses.* 2021. 13(6) (no pagination):#pages#

**Chughtai, O. R.,Batool, H.,Khan, M. D.,Chughtai, A. S.**. Frequency of COVID-19 IgG Antibodies among Special Police Squad Lahore, Pakistan. *Jcpsp, Journal of the College of Physicians & Surgeons - Pakistan.* 2020. 30:735-739

**Chung, C.,Palomino, J.,Thammasitboon, S.**. Assessment of risk of transmission from a COVID-19 polymerase chain reaction (pcr) re-positive patient. *American Journal of Respiratory and Critical Care Medicine. Conference: American Thoracic Society International Conference, ATS.* 2021. 203:#pages#

**Conte, M., Feltracco, M., Chirizzi, D., Trabucco, S., Dinoi, A., Gregoris, E., Barbaro, E., La Bella, G., Ciccarese, G., Belosi, F., La Salandra, G., Gambaro, A., Contini, D.**. Airborne concentrations of SARS-CoV-2 in indoor community environments in Italy. *Environmental science and pollution research international.* 2022. 29(10):13905-13916

**Cook, J. D.,Grant, E. H. C.,Coleman, J. T. H.,Sleeman, J. M.,Runge, M. C.**. Risks posed by SARS-CoV-2 to North American bats during winter fieldwork. *Conservation Science & Practice.* 2021. 3:e410

**Corman, V. M.,Rabenau, H. F.,Adams, O.,Oberle, D.,Funk, M. B.,Keller-Stanislawski, B.,Timm, J.,Drosten, C.,Ciesek, S.**. SARS-CoV-2 asymptomatic and symptomatic patients and risk for transfusion transmission. *Transfusion.* 2020. 60:1119-1122

**Correa-Martinez, C. L.,Kampmeier, S.,Kumpers, P.,Schwierzeck, V.,Hennies, M.,Hafezi, W.,Kuhn, J.,Pavenstadt, H.,Ludwig, S.,Mellmann, A.**. A Pandemic in Times of Global Tourism: Superspreading and Exportation of COVID-19 Cases from a Ski Area in Austria. *Journal of Clinical Microbiology.* 2020. 58:26

**Costa, S. F.,Giavina-Bianchi, P.,Buss, L.,Mesquita Peres, C. H.,Rafael, M. M.,Dos Santos, L. G. N.,Bedin, A. A.,Francisco, M. C. P. B.,Satakie, F. M.,Jesus Menezes, M. A.,Dal Secco, L. M.,Rodrigues Caron, D. M.,de Oliveira, A. B.,de Faria, M. F. L.,de Aurelio Penteado, A. S.,de Souza, I. O. M.,de Fatima Pereira, G.,Pereira, R.,Matos Porto, A. P.,Sanchez Espinoza, E. P.,Mendes-Correa, M. C.,Dos Santos Lazari, C.,Kalil, J.,de Moliterno Perondi, M. B.,de Oliveira Bonfa, E. S. D.,Perreira, A. J.,Sabino, E.,da Silva Duarte, A. J.,Segurado, A. C.,Dos Santos, V. A.,Levin, A. S.**. SARS-CoV-2 seroprevalence and risk factors among oligo/asymptomatic healthcare workers(HCW): estimating the impact of community transmission. *Clinical infectious diseases : an official publication of the Infectious Diseases Society of America..* 2020. 13:#pages#

**Covid- National Incident Room Surveillance Team**. COVID-19, Australia: Epidemiology Report 16 (Reporting week to 23:59 AEST 17 May 2020). *Communicable Diseases Intelligence.* 2020. 44:22

**Covid-Field Response, Group,Laboratory Testing, Group,Ying, Zhang**. The Source of Infection of the 137<sup>th</sup> Confirmed Case of COVID-19 - Tianjin Municipality, China, June 17, 2020. *China CDC Weekly.* 2020. 2:507-510

**Cruz, C. J. P.,Ganly, R.,Li, Z.,Gietel-Basten, S.**. Exploring the young demographic profile of COVID-19 cases in Hong Kong: Evidence from migration and travel history data. *PLoS ONE.* 2020. 15(6 June) (no pagination):#pages#

**da Silva, F. A. F., de Brito, B. B., Santos, M. L. C., Marques, H. S., da Silva Junior, R. T., de Carvalho, L. S., de Sousa Cruz, S., Rocha, G. R., Correa Santos, G. L., de Souza, K. C., Maciel, R. G. A., Lopes, D. S., Silva, N. O. E., Oliveira, M. V., de Melo, F. F.**. Transmission of severe acute respiratory syndrome coronavirus 2 via fecal-oral: Current knowledge. *World Journal of Clinical Cases.* 2021. 9:8280-8294

**Danilo, Buonsenso, Cristina De, Rose, Rosanna, Moroni, Piero, Valentini**. SARS-CoV-2 infections in Italian schools: preliminary findings after one month of school opening during the second wave of the pandemic. *#journal#.* 2020. #volume#:#pages#

**Dargin, J. S.,Li, Q.,Jawer, G.,Xiao, X.,Mostafavi, A.**. Compound hazards: An examination of how hurricane protective actions could increase transmission risk of COVID-19. *International Journal of Disaster Risk Reduction.* 2021. 65:102560

**Dave, D.,McNichols, D.,Sabia, J. J.**. The contagion externality of a superspreading event: The Sturgis Motorcycle Rally and COVID-19. *Southern Economic Journal.* 2020. 02:02

**Davies, M. A. M., Spincer, M., Klein, A., Walters, S., Wright, P., Hurley, A., Stokes, K., Hill, J.**. "Pilot" spectator events in British horseracing during COVID-19: post-event SMS COVID-19 reporting. *Scandinavian journal of medicine & science in sports.* 2022. 32(2):372-380

**Daw, M. A.,El-Bouzedi, A. H.,Ahmed, M. O.,Alejenef, A. A.**. The epidemiological characteristics of covid-19 in libya during the ongoing-armed conflict. *Pan African Medical Journal.* 2020. 37:1-10

**de Barros, E. N. C.,Do Valle, A. P.,Braga, P. E.,Viscondi, J. Y. K.,da Fonseca, A. R. B.,Vanni, T.,da Silva, A.,Cardoso, M. R.,Villas Boas, P. J. F.,Precioso, A. R.**. COVID-19 in long-term care facilities in Brazil: Serological survey in a post-outbreak setting. *Revista do Instituto de Medicina Tropical de Sao Paulo.* 2021. 63:1-6

**de Laval, F.,Grosset-Janin, A.,Delon, F.,Allonneau, A.,Tong, C.,Letois, F.,Couderc, A.,Sanchez, M. A.,Destanque, C.,Biot, F.,Raynaud, F.,Bigaillon, C.,Ferraris, O.,Simon-Loriere, E.,Enouf, V.,Andriamanantena, D.,de Santi, V. P.,Javelle, E.,Merens, A.**. Lessons learned from the investigation of a COVID-19 cluster in Creil, France: effectiveness of targeting symptomatic cases and conducting contact tracing around them. *BMC Infectious Diseases.* 2021. 21:457

**de Souza Melo, A.,da Penha Sobral, A. I. G.,Marinho, M. L. M.,Duarte, G. B.,Vieira, A. A.,Sobral, M. F. F.**. The impact of social distancing on COVID-19 infections and deaths. *Tropical Diseases, Travel Medicine and Vaccines.* 2021. 7(1) (no pagination):#pages#

**Del Brutto, O. H.,Costa, A. F.,Mera, R. M.,Andrade-Molina, D.,Recalde, B. Y.,Garcia, H. H.,Fernandez-Cadena, J. C.**. SARS-CoV-2 RNA in Swabbed Samples from Latrines and Flushing Toilets: A Case-Control Study in a Rural Latin American Setting. *American Journal of Tropical Medicine & Hygiene.* 2021. 13:13

**Delgado-Sanchez, S., Serrano-Ortiz, A., Ruiz-Montero, R., Lorusso, N., Rumbao-Aguirre, J. M., Salcedo-Leal, I.**. Impact of the first superspreading outbreak of COVID-19 related to a nightlife establishment in Andalusia, Spain. *Journal of Healthcare Quality Research.* 2021. 29:29

**Deng, X., Garcia-Knight, M. A., Khalid, M. M., Servellita, V., Wang, C., Morris, M. K., Sotomayor-Gonzalez, A., Glasner, D. R., Reyes, K. R., Gliwa, A. S., Reddy, N. P., Sanchez San Martin, C., Federman, S., Cheng, J., Balcerek, J., Taylor, J., Streithorst, J. A., Miller, S., Sreekumar, B., Chen, P. Y., Schulze-Gahmen, U., Taha, T. Y., Hayashi, J. M., Simoneau, C. R., Kumar, G. R., McMahon, S., Lidsky, P. V., Xiao, Y., Hemarajata, P., Green, N. M., Espinosa, A., Kath, C., Haw, M., Bell, J., Hacker, J. K., Hanson, C., Wadford, D. A., Anaya, C., Ferguson, D., Frankino, P. A., Shivram, H., Lareau, L. F., Wyman, S. K., Ott, M., Andino, R., Chiu, C. Y.**. Transmission, infectivity, and neutralization of a spike L452R SARS-CoV-2 variant. *Cell.* 2021. 184(13):3426-3437.e8

**Deng, X.,Gu, W.,Federman, S.,du Plessis, L.,Pybus, O. G.,Faria, N.,Wang, C.,Yu, G.,Pan, C. Y.,Guevara, H.,Sotomayor-Gonzalez, A.,Zorn, K.,Gopez, A.,Servellita, V.,Hsu, E.,Miller, S.,Bedford, T.,Greninger, A. L.,Roychoudhury, P.,Starita, L. M.,Famulare, M.,Chu, H. Y.,Shendure, J.,Jerome, K. R.,Anderson, C.,Gangavarapu, K.,Zeller, M.,Spencer, E.,Andersen, K. G.,MacCannell, D.,Paden, C. R.,Li, Y.,Zhang, J.,Tong, S.,Armstrong, G.,Morrow, S.,Willis, M.,Matyas, B. T.,Mase, S.,Kasirye, O.,Park, M.,Chan, C.,Yu, A. T.,Chai, S. J.,Villarino, E.,Bonin, B.,Wadford, D. A.,Chiu, C. Y.**. A Genomic Survey of SARS-CoV-2 Reveals Multiple Introductions into Northern California without a Predominant Lineage. *MedRxiv : the Preprint Server for Health Sciences.* 2020. 30:30

**Denis, F.,Septans, A. L.,Le Goff, F.,Jeanneau, S.,Lescure, F. X.**. Analysis of COVID-19 transmission sources in France by self-assessment before and after the partial Lockdown: Observational study. *Journal of Medical Internet Research.* 2021. 23(5) (no pagination):#pages#

**Dhewantara, P. W.,Puspita, T.,Marina, R.,Lasut, D.,Riandi, M. U.,Wahono, T.,Ridwan, W.,Ruliansyah, A.**. Geo-clusters and socio-demographic profiles at village-level associated with COVID-19 incidence in the metropolitan city of Jakarta: An ecological study. *Transboundary & Emerging Diseases.* 2021. 05:05

**Di Carlo, P.,Chiacchiaretta, P.,Sinjari, B.,Aruffo, E.,Stuppia, L.,De Laurenzi, V.,Di Tomo, P.,Pelusi, L.,Potenza, F.,Veronese, A.,Vecchiet, J.,Falasca, K.,Ucciferri, C.**. Air and surface measurements of SARS-CoV-2 inside a bus during normal operation. *PLoS ONE.* 2020. 15(11) (no pagination):#pages#

**Di Carlo, P.,Falasca, K.,Ucciferri, C.,Sinjari, B.,Aruffo, E.,Antonucci, I.,Di Serafino, A.,Pompilio, A.,Damiani, V.,Mandatori, D.,De Fabritiis, S.,Dufrusine, B.,Capone, E.,Chiacchiaretta, P.,Brune, W. H.,Di Bonaventura, G.,Vecchiet, J.**. Normal breathing releases SARS-CoV-2 into the air. *Journal of Medical Microbiology.* 2021. 70(3) (no pagination):#pages#

**Diebig, M.,Gritzka, S.,Dragano, N.,Angerer, P.**. Presentation of a participatory approach to develop preventive measures to reduce COVID-19 transmission in child care. *Journal of Occupational Medicine and Toxicology.* 2021. 16(1) (no pagination):#pages#

**Diez-Fuertes, F.,Iglesias-Caballero, M.,Garcia-Perez, J.,Monzon, S.,Jimenez, P.,Varona, S.,Cuesta, I.,Zaballos, A.,Jimenez, M.,Checa, L.,Pozo, F.,Perez-Olmeda, M.,Thomson, M. M.,Alcami, J.,Casas, I.**. A Founder Effect Led Early SARS-CoV-2 Transmission in Spain. *Journal of Virology.* 2021. 95:13

**Ding, H.,Zhang, J.**. Dynamic associations between temporal behavior changes caused by the COVID-19 pandemic and subjective assessments of policymaking: A case study in Japan. *Transport Policy.* 2021. 110:58-70

**Dirgar, E.,Tosun, B.,Berse, S.,Tosun, N.**. Evaluating the novel coronavirus infection outbreak surveillance results in a state hospital: A retrospective study. *African Health Sciences.* 2021. 21(3):1107-1116

**Du, P.,Ding, N.,Li, J.,Zhang, F.,Wang, Q.,Chen, Z.,Song, C.,Han, K.,Xie, W.,Liu, J.,Wei, L.,Ma, S.,Hua, M.,Yu, F.,Wang, L.,Wang, W.,An, K.,Chen, J.,Liu, H.,Gao, G.,Wang, S.,Huang, Y.,Wu, A. R.,Wang, J.,Liu, D.,Zeng, H.,Chen, C.**. Genomic surveillance of COVID-19 cases in Beijing. *Nature Communications.* 2020. 11(1) (no pagination):#pages#

**Dzingirai, B.,Matyanga, C. M. J.,Mudzviti, T.,Siyawamwaya, M.,Tagwireyi, D.**. Risks to the community pharmacists and pharmacy personnel during COVID-19 pandemic: perspectives from a low-income country. *Journal of Pharmaceutical Policy & Practice.* 2020. 13:42

**Echternach, M.,Herrmann, L.,Gantner, S.,Tur, B.,Peters, G.,Westphalen, C.,Benthaus, T.,Koberlein, M.,Kuranova, L.,Dollinger, M.,Kniesburges, S.**. The Effect of Singers' Masks on the Impulse Dispersion of Aerosols During Singing. *Journal of Voice.* 2021. 02:02

**El Bouzidi, K.,Pirani, T.,Rosadas, C.,Ijaz, S.,Pearn, M.,Chaudhry, S.,Patel, S.,Sureda-Vives, M.,Fernandez, N.,Khan, M.,Cherepanov, P.,McClure, M. O.,Tedder, R. S.,Zuckerman, M.,Covid, Stoics**. Severe Acute Respiratory Syndrome Coronavirus-2 Infections in Critical Care Staff: Beware the Risks Beyond the Bedside. *Critical Care Medicine.* 2021. 49:428-436

**Elisabetta, Larosa, Olivera, Djuric, Mariateresa, Cassinadri, Silvia, Cilloni, Eufemia, Bisaccia, Massimo, Vicentini, Francesco, Venturelli, Paolo Giorgi, Rossi, Patrizio, Pezzotti, Emanuela, Bedeschi, Reggio Emilia Covid-19 Working, Group**. Secondary transmission of COVID-19 in preschool and school settings after their reopening in northern Italy: a population-based study. *#journal#.* 2020. #volume#:#pages#

**Ellingford, J. M.,George, R.,McDermott, J. H.,Ahmad, S.,Edgerley, J. J.,Gokhale, D.,Newman, W. G.,Ball, S.,Machin, N.,Black, G. C.**. Genomic and healthcare dynamics of nosocomial SARS-CoV-2 transmission. *eLife.* 2021. 10:17

**Eslami, H.,Jalili, M.**. The role of environmental factors to transmission of SARS-CoV-2 (COVID-19). *AMB Express.* 2020. 10(1) (no pagination):#pages#

**Fawad, M.,Mubarik, S.,Malik, S. S.,Hao, Y.,Yu, C.,Ren, J.**. Trend dynamics of severe acute respiratory syndrome coronavirus 2 (Sars-cov-2) transmission in 16 cities of Hubei Province, China. *Clinical Epidemiology.* 2020. 12:699-709

**Feehan, D. M.,Mahmud, A. S.**. Quantifying population contact patterns in the United States during the COVID-19 pandemic. *Nature Communications.* 2021. 12(1) (no pagination):#pages#

**Feng, M.,Ling, Q.,Xiong, J.,Manyande, A.,Xu, W.,Xiang, B.**. Geographical and Epidemiological Characteristics of Sporadic Coronavirus Disease 2019 Outbreaks From June to December 2020 in China: An Overview of Environment-To-Human Transmission Events. *Frontiers in Medicine.* 2021. 8:654422

**Firew, T.,Greene, C.,Salman, K.,Sano, E.,Flores, S.,Lee, J.,Lang, K.,Chang, B.**. 235 An Assessment of Health Care Worker Safety during COVID-19. *Annals of Emergency Medicine.* 2020. 76(4 Supplement):S91

**Fitzgerald, N.,Uny, I.,Brown, A.,Eadie, D.,Ford, A.,Lewsey, J.,Stead, M.**. Managing COVID-19 Transmission Risks in Bars: An Interview and Observation Study. *Journal of Studies on Alcohol & Drugs.* 2021. 82:42-54

**Fong, D.,Mair, M. J.,Lanthaler, F.,Alber, M.,Mitterer, M.**. Mobility as a driver of severe acute respiratory syndrome coronavirus 2 in cancer patients during the second coronavirus disease 2019 pandemic wave. *International Journal of Cancer..* 2021. #volume#:#pages#

**Fontanet, A., Tondeur, L., Grant, R., Temmam, S., Madec, Y., Bigot, T., Grzelak, L., Cailleau, I., Besombes, C., Ungeheuer, M. N., Renaudat, C., Perlaza, B. L., Arowas, L., Jolly, N., Pellerin, S. F., Kuhmel, L., Staropoli, I., Huon, C., Chen, K. Y., Crescenzo-Chaigne, B., Munier, S., Charneau, P., Demeret, C., Bruel, T., Eloit, M., Schwartz, O., Hoen, B.**. SARS-CoV-2 infection in schools in a northern French city: A retrospective serological cohort study in an area of high transmission, France, January to April 2020. *Eurosurveillance.* 2021. 26(15):1-12

**Froum, S. H.,Froum, S. J.**. Incidence of COVID-19 Virus Transmission in Three Dental Offices: A 6-Month Retrospective Study. *International Journal of Periodontics & Restorative Dentistry.* 2020. 40:853-859

**Fu, L.,Nielsen, P. V.,Wang, Y.,Liu, L.**. Measuring interpersonal transmission of expiratory droplet nuclei in close proximity. *Indoor and Built Environment..* 2021. #volume#:#pages#

**Fuentes, L. R.,Zhang, H.,Cherif, A.,Kotanko, P.**. Network analysis of in-center spread of COVID-19: A single dialysis center experience. *Journal of the American Society of Nephrology.* 2020. 31:269

**Furuse, Y.,Sando, E.,Tsuchiya, N.,Miyahara, R.,Yasuda, I.,Ko, Y. K.,Saito, M.,Morimoto, K.,Imamura, T.,Shobugawa, Y.,Nagata, S.,Jindai, K.,Imamura, T.,Sunagawa, T.,Suzuki, M.,Nishiura, H.,Oshitani, H.**. Clusters of Coronavirus Disease in Communities, Japan, January-April 2020. *Emerging Infectious Diseases.* 2020. 26:09

**Gault, G.,Monpierre, O.,Carion, G.,Rispal, P.,Royer, H.,Lafon, M. E.,Habold, D.,Filleul, L.**. Screening around a COVID-19 cluster: Exploring its impact on a local virus outbreak. *Infectious Diseases Now.* 2021. 51:391-394

**Ge, Y.,Martinez, L.,Sun, S.,Chen, Z.,Zhang, F.,Li, F.,Sun, W.,Chen, E.,Pan, J.,Li, C.,Sun, J.,Handel, A.,Ling, F.,Shen, Y.**. COVID-19 Transmission Dynamics among Close Contacts of Index Patients with COVID-19: A Population-Based Cohort Study in Zhejiang Province, China. *JAMA Internal Medicine..* 2021. #volume#:#pages#

**Gelbart, B.,Schapkaitz, E.,Kaftel, S.,Peretz, E.,Peretz, A.**. The relationship between serological testing, demographics, clinical presentation and RT-PCR testing for COVID-19. *Clinical Laboratory.* 2021. 67(5):1293-1298

**Germain, N.,Herwegh, S.,Hatzfeld, A. S.,Bocket, L.,Prevost, B.,Danze, P. M.,Marchetti, P.**. Retrospective study of COVID-19 seroprevalence among tissue donors at the onset of the outbreak before implementation of strict lockdown measures in France. *Cell & Tissue Banking.* 2021. 22:511-518

**Giachetto, G.,Mauvezin, J.,Babio, S.,Dabezies, C.,Calvo, L.,Mara, P.**. [SARS-CoV-2 infection in children: Clinical characteristics and dynamics of SARS-CoV-2 transmission in a private health center. Uruguay]. *Revista Chilena de Infectologia.* 2021. 38:500-505

**Gibson, G., Weitz, J. S., Shannon, M. P., Holton, B., Bryksin, A., Liu, B., Sieglinger, M., Coenen, A. R., Zhao, C., Beckett, S. J., Bramblett, S., Williamson, J., Farrell, M., Ortiz, A., Abdallah, C. T., Garcia, A. J.**. Surveillance-to-Diagnostic Testing Program for Asymptomatic SARS-CoV-2 Infections on a Large, Urban Campus in Fall 2020. *Epidemiology.* 2021. 01:01

**Giner, J.,Villanueva-saz, S.,Tobajas, A. P.,Perez, M. D.,Gonzalez, A.,Verde, M.,Yzuel, A.,Garcia-garcia, A.,Taleb, V.,Lira-navarrete, E.,Hurtado-guerrero, R.,Pardo, J.,Santiago, L.,Pano, J. R.,Ruiz, H.,Lacasta, D.,Fernandez, A.**. Sars-cov-2 seroprevalence in household domestic ferrets (Mustela putorius furo). *Animals.* 2021. 11(3):1-11

**Gomez-Carballa, A.,Bello, X.,Pardo-Seco, J.,Perez Del Molino, M. L.,Martinon-Torres, F.,Salas, A.**. Phylogeography of SARS-CoV-2 pandemic in Spain: a story of multiple introductions, micro-geographic stratification, founder effects, and super-spreaders. *Zoological Research.* 2020. 41:605-620

**Gong, X.,Xiao, W.,Cui, Y.,Wang, Y.,Kong, D.,Mao, S.,Zheng, Y.,Xiang, L.,Lu, L.,Jiang, C.,Yu, X.,Zhu, Y.,Fang, Q.,Pan, H.,Wu, H.**. Three infection clusters related with potential pre-symptomatic transmission of coronavirus disease (COVID-19), Shanghai, China, January to February 2020. *Euro Surveillance: Bulletin Europeen sur les Maladies Transmissibles = European Communicable Disease Bulletin.* 2020. 25:08

**Gonzalez-Reiche, A. S.,Hernandez, M. M.,Sullivan, M. J.,Ciferri, B.,Alshammary, H.,Obla, A.,Fabre, S.,Kleiner, G.,Polanco, J.,Khan, Z.,Alburquerque, B.,van de Guchte, A.,Dutta, J.,Francoeur, N.,Melo, B. S.,Oussenko, I.,Deikus, G.,Soto, J.,Sridhar, S. H.,Wang, Y. C.,Twyman, K.,Kasarskis, A.,Altman, D. R.,Smith, M.,Sebra, R.,Aberg, J.,Krammer, F.,Garcia-Sastre, A.,Luksza, M.,Patel, G.,Paniz-Mondolfi, A.,Gitman, M.,Sordillo, E. M.,Simon, V.,van Bakel, H.**. Introductions and early spread of SARS-CoV-2 in the New York City area. *Science.* 2020. 369:297-301

**Gorges, R. J.,Konetzka, R. T.**. Factors Associated with Racial Differences in Deaths among Nursing Home Residents with COVID-19 Infection in the US. *JAMA Network Open.* 2021. 4(2) (no pagination):#pages#

**Grimwood, K.,Lambert, S. B.,Ware, R. S.**. Endemic Non-SARS-CoV-2 Human Coronaviruses in a Community-Based Australian Birth Cohort. *Pediatrics.* 2020. 146:11

**Grosso, A.,Rigoli, R.,Formentini, S.,Di Perri, G.,Scotton, P.,Dapavo, G.,Fioretto, M.,Scarpa, G.**. Suppression of Covid-19 outbreak among healthcare workers at the Treviso Regional Hospital, Italy and lessons for ophthalmologists. *European Journal of Ophthalmology.* 2020. #volume#:1120672120982520

**Grover, S.,Sinha, P.,Sahoo, S.,Arumugham, S.,Baliga, S.,Chakrabarti, S.,Thirthalli, J.**. Electroconvulsive therapy during the COVID-19 pandemic. *Indian Journal of Psychiatry.* 2020. 62:582-584

**Guarino, M.,Cossiga, V.,Fiorentino, A.,Pontillo, G.,Morisco, F.**. Use of Telemedicine for Chronic Liver Disease at a Single Care Center During the COVID-19 Pandemic: Prospective Observational Study. *Journal of Medical Internet Research.* 2020. 22:e20874

**Guimaraes, R. M.,Monteiro Da Silva, J. H. C.,Brusse, G. P. L.,Martins, T. C. F.**. Effect of Physical Distancing on Covid-19 Incidence in Brazil: Does the Strictness of Mandatory Rules Matter?. *Health Policy & Planning.* 2021. 11:11

**Guo, H.,Li, W.,Huang, Y.,Li, X.,Li, Z.,Zhou, H.,Sun, E.,Li, L.,Li, J.**. Increased microbial loading in aerosols produced by non-contact air-puff tonometer and relative suggestions for the prevention of coronavirus disease 2019 (COVID-19). *PLoS ONE.* 2020. 15(10 October) (no pagination):#pages#

**Haas, E. J.,Angulo, F. J.,McLaughlin, J. M.,Anis, E.,Singer, S. R.,Khan, F.,Brooks, N.,Smaja, M.,Mircus, G.,Pan, K.,Southern, J.,Swerdlow, D. L.,Jodar, L.,Levy, Y.,Alroy-Preis, S.**. Impact and effectiveness of mRNA BNT162b2 vaccine against SARS-CoV-2 infections and COVID-19 cases, hospitalisations, and deaths following a nationwide vaccination campaign in Israel: an observational study using national surveillance data. *Lancet.* 2021. 397:1819-1829

**Hamad, A. I.,Elgaali, M.,Abuhelaiqa, E.,Alkadi, M. M.,Othman, M.,Elesnawi, M. A.,Ghonimi, T. A.,Al-Malki, H. A.**. Impact of undertaking safeguards to limit exposure and prevent COVID-19 infection in ambulatory dialysis: A single-center experience. *Journal of the American Society of Nephrology.* 2020. 31:268

**Han, J.,He, S.**. Urban flooding events pose risks of virus spread during the novel coronavirus (COVID-19) pandemic. *Science of the Total Environment.* 2021. 755:142491

**Han, Y.,Yang, L.,Jia, K.,Li, J.,Feng, S.,Chen, W.,Zhao, W.,Pereira, P.**. Spatial distribution characteristics of the COVID-19 pandemic in Beijing and its relationship with environmental factors. *Science of the Total Environment.* 2021. 761 (no pagination):#pages#

**Harith, A. A., Ab Gani, M. H., Griffiths, R., Abu Bakar, N. A., Hadi, A. A., Mahjom, M.**. COVID-19 infection among healthcare workers at Malaysia hospitals. *Occupational and Environmental Medicine.* 2021. 78(SUPPL 1):A14

**Hasan, S. M.,Das, S.,Hanifi, S. M. A.,Shafique, S.,Rasheed, S.,Reidpath, D. D.**. A place-based analysis of COVID-19 risk factors in Bangladesh urban slums: a secondary analysis of World Bank microdata. *BMC Public Health.* 2021. 21:502

**Hassan, M. M.,El Zowalaty, M. E.,Khan, S. A.,Islam, A.,Nayem, M. R. K.,Jarhult, J. D.**. Role of Environmental Temperature on the Attack rate and Case fatality rate of Coronavirus Disease 2019 (COVID-19) Pandemic. *Infection Ecology and Epidemiology.* 2020. 10(1) (no pagination):#pages#

**Health, Colorado School of Public, University, Colorado State, University of Colorado, Boulder, University, Colorado Mesa, University, Regis, Center, Denver Health Medical, University of Colorado, Colorado Springs, University of Colorado, Denver**. Asymptomatic and Pre-symptomatic Transmission of SARS-CoV-2 in a Community Congregant Setting. *#journal#.* 2021. #volume#:#pages#

**Health, Norwegian Institute of Public, Oslo, University of**. Antigen Rapid Test Screening to Prevent SARS-CoV-2 Transmission (COVID-19) at Mass Gathering Events. *#journal#.* 2021. #volume#:#pages#

**Heylen, L.,Van Kerrebroeck, M.,Oris, E.,Hendrickx, L.,Macken, E.,Metalidis, C.,Peeters, J.,Van Mieghem, A.,Steensels, D.**. Institutional transport as risk factor for covid-19 in hemodialysis patients. *Nephrology Dialysis Transplantation.* 2021. 36(SUPPL 1):i463-i465

**Hong, B.,Bonczak, B. J.,Gupta, A.,Thorpe, L. E.,Kontokosta, C. E.**. Exposure density and neighborhood disparities in COVID-19 infection risk. *Proceedings of the National Academy of Sciences of the United States of America.* 2021. 118:30

**Hospital, Oslo University, AS, Age Labs**. Risk Factors for Community- and Workplace Transmission of COVID-19. *#journal#.* 2020. #volume#:#pages#

**Hou, L.,Zhou, H.,Meng, N.,Yu, X.,Wang, X.,Wang, T.,Zhang, J.,Wang, Y.,Li, S.,Guo, S.,Yu, J.,Chen, M.,Shi, W.,Xiao, N.,Yang, C.,Liu, J.**. A COVID-19 Outbreak Emerging in a Food Processing Company - Harbin City, Heilongjiang Province, China, January-February 2021. *China CDC Weekly.* 2021. 3:681-687

**Hu, S.,Wang, W.,Wang, Y.,Litvinova, M.,Luo, K.,Ren, L.,Sun, Q.,Chen, X.,Zeng, G.,Li, J.,Liang, L.,Deng, Z.,Zheng, W.,Li, M.,Yang, H.,Guo, J.,Wang, K.,Chen, X.,Liu, Z.,Yan, H.,Shi, H.,Chen, Z.,Zhou, Y.,Sun, K.,Vespignani, A.,Viboud, C.,Gao, L.,Ajelli, M.,Yu, H.**. Infectivity, susceptibility, and risk factors associated with SARS-CoV-2 transmission under intensive contact tracing in Hunan, China. *Nature communications.* 2021. 12:1533

**Hu, X.,Ni, W.,Wang, Z.,Ma, G.,Pan, B.,Dong, L.,Gao, R.,Jiang, F.**. The distribution of SARS-CoV-2 contamination on the environmental surfaces during incubation period of COVID-19 patients. *Ecotoxicology and Environmental Safety.* 2021. 208 (no pagination):#pages#

**Huang, L.,Zhang, X.,Zhang, L.,Xu, J.,Wei, Z.,Xu, Y.,Zhang, C.,Xu, A.**. Swab and Sputum SARS-CoV-2 RNA-Negative, CT-Positive, Symptomatic Contacts of COVID-19 Cases: A Hypothesis-Generating Prospective Population-Based Cohort Study of Eight Clusters. *Frontiers in Medicine.* 2021. 8:685544

**Huang, X.,Shao, X.,Xing, L.,Hu, Y.,Sin, D. D.,Zhang, X.**. The impact of lockdown timing on COVID-19 transmission across US counties. *EClinicalMedicine.* 2021. (no pagination):#pages#

**Hwang, H., Lim, J. S., Song, S. A., Achangwa, C., Sim, W., Kim, G., Ryu, S.**. Transmission dynamics of the Delta variant of SARS-CoV-2 infections in South Korea. *Journal of Infectious Diseases.* 2021. 02:02

**Hwang, S. E.,Chang, J. H.,Oh, B.,Heo, J.**. Possible aerosol transmission of COVID-19 associated with an outbreak in an apartment in Seoul, South Korea, 2020. *International Journal of Infectious Diseases.* 2021. 104:73-76

**Imamura, T.,Saito, M.,Ko, Y. K.,Imamura, T.,Otani, K.,Akaba, H.,Ninomiya, K.,Furuse, Y.,Miyahara, R.,Sando, E.,Yasuda, I.,Tsuchiya, N.,National, Covid-Cluster Response Taskforce,Suzuki, M.,Oshitani, H.**. Roles of Children and Adolescents in COVID-19 Transmission in the Community: A Retrospective Analysis of Nationwide Data in Japan. *Frontiers in Pediatrics.* 2021. 9:705882

**Islamoglu, M. S., Cengiz, M., Borku Uysal, B., Ikitimur, H., Demirbilek, M., Dokur, M., Seyhan, S., Koc, S., Yavuzer, S.**. COVID-19 seroconversion in the aircrew from Turkey. *Travel Medicine and Infectious Disease.* 2021. 44 (no pagination):#pages#

**Janowski, A. B., Polgreen, P. M., Beekmann, S. E., Newland, J. G.**. Perceptions of risk of SARS-CoV-2 transmission in social and educational activities by infectious diseases and general pediatric healthcare providers, a pre-vaccine risk perception cross-sectional survey. *PLoS ONE [Electronic Resource].* 2022. 17:e0263767

**Jean-Paul, R. Soucy, Amir, Ghasemi, Shelby, L. Sturrock, Isha, Berry, Sarah, A. Buchan, Derek, R. MacFadden, Nick, Daneman, Nicholas, Gibb, Kevin, A. Brown**. Increased Interregional Travel to Shopping Malls and Restaurants in Response to Differential COVID-19 Restrictions in the Greater Toronto Area. *#journal#.* 2021. #volume#:#pages#

**Jindahra, P., Wongboonsin, K., Wongboonsin, P.**. Demographic and initial outbreak patterns of COVID-19 in Thailand. *Journal of Population Research.* 2021. #volume#:1-22

**Jordan, I., Fernandez de Sevilla, M., Fumado, V., Bassat, Q., Bonet-Carne, E., Fortuny, C., Garcia-Miquel, A., Jou, C., Adroher, C., Mele Casas, M., Girona-Alarcon, M., Hernandez Garcia, M., Pons Tomas, G., Ajanovic, S., Arias, S., Balanza, N., Baro, B., Millat-Martinez, P., Varo, R., Alonso, S., Alvarez-Lacalle, E., Lopez, D., Claverol, J., Cubells, M., Brotons, P., Codina, A., Cuadras, D., Bruijning-Verhagen, P., Faust, S., Munro, A., Munoz-Almagro, C., Catala, M., Prats, C., Garcia-Garcia, J. J., Gratacos, E.**. Transmission of Severe Acute Respiratory Syndrome Coronavirus 2 Infection Among Children in Summer Schools Applying Stringent Control Measures in Barcelona, Spain. *Clinical infectious diseases : an official publication of the Infectious Diseases Society of America.* 2022. 74(1):66-73

**Juscamayta-Lopez, E.,Carhuaricra, D.,Tarazona, D.,Valdivia, F.,Rojas, N.,Maturrano, L.,Gavilan, R.**. Phylogenomics reveals multiple introductions and early spread of SARS-CoV-2 into Peru. *Journal of Medical Virology.* 2021. 93:5961-5968

**Kan, Z.,Kwan, M. P.,Wong, M. S.,Huang, J.,Liu, D.**. Identifying the space-time patterns of COVID-19 risk and their associations with different built environment features in Hong Kong. *Science of the Total Environment.* 2021. 772:145379

**Kang, D.,Ellgen, C.,Kulstad, E.**. Possible effects of air temperature on COVID-19 disease severity and transmission rates. *Journal of Medical Virology.* 2021. 93(9):5358-5366

**Kang, Y.,You, Z.,Wang, K.,Dong, Z.,Zhang, J.,Qiu, Y.,Ge, G.**. A retrospective view of pediatric cases infected with SARS-CoV-2 of a middle-sized city in mainland China. *Medicine.* 2020. 99:e23797

**Kannian, P., Jayaraman, B. G., Alamelu, S., Lavanya, C., Kumarasamy, N., Rajan, G., Ranganathan, K., Mahanathi, P., Ashwini, V., Challacombe, S. J., Webster-Cyriaque, J., Johnson, N. W.**. Implications in the quantification of SARS-CoV2 copies in concurrent nasopharyngeal swabs, whole mouth fluid and respiratory droplets. *Virus Research.* 2021. 303 (no pagination):#pages#

**Karami, C.,Normohammadi, A.,Dargahi, A.,Vosoughi, M.,Zandian, H.,Jeddi, F.,Mokhtari, S. A.,Moradi-Asl, E.**. Investigation of SARS-CoV-2 virus on nozzle surfaces of fuel supply stations in North West of Iran. *Science of the Total Environment.* 2021. 780:146641

**Karmarkar, E. N., Blanco, I., Amornkul, P. N., Dubois, A., Deng, X., Moonan, P. K., Rubenstein, B. L., Miller, D. A., Kennedy, I., Yu, J., Dauterman, J. P., Ongpin, M., Hathaway, W., Hoo, L., Trammell, S., Dosunmu, E. F., Yu, G., Khwaja, Z., Lu, W., Talai, N. Z., Jain, S., Louie, J. K., Philip, S. S., Federman, S., Masinde, G., Wadford, D. A., Bobba, N., Stoltey, J., Smith, A., Epson, E., Chiu, C. Y., Bennett, A. S., Vasquez, A. M., Williams, T.**. Timely intervention and control of a novel coronavirus (COVID-19) outbreak at a large skilled nursing facility-San Francisco, California, 2020. *Infection Control and Hospital Epidemiology.* 2021. 42(10):1173-1180

**Kashuba, V. I.,Hryshchenko, N. V.,Gerashchenko, G. V.,Melnichuk, N. S.,Marchishak, T. V.,Chernushyn, S. Yu,Chernenko, L. M.,Liashko, V. K.,Tkachuk, Z. Yu,Tukalo, M. A.**. Identification and characterization of the sars-cov-2 lineage b.1.1.7 upon the new outbreak of the covid-19 in Ukraine in february 2021. *Biopolymers and Cell.* 2021. 37(2):117-124

**Kayaaslan, B.,Korukluoglu, G.,Hasanoglu, I.,Kalem, A. K.,Eser, F.,Akinci, E.,Guner, R.**. Investigation of SARS-CoV-2 in Semen of Patients in the Acute Stage of COVID-19 Infection. *Urologia Internationalis.* 2020. 104(9-10):678-683

**Kearney, A.,Searl, J.,Erickson-DiRenzo, E.,Doyle, P. C.**. The Impact of COVID-19 on Speech-Language Pathologists Engaged in Clinical Practices With Elevated Coronavirus Transmission Risk. *American Journal of Speech-Language Pathology.* 2021. 30:1673-1685

**Kemp, S. A., Cheng, M. T. K., Hamilton, W., Kamelian, K., Singh, S., Rakshit, P., Aggrawal, A., Illingworth, C., Gupta, R. K.**. Transmission of B.1.617.2 Delta variant between vaccinated healthcare workers. *medRxiv..* 2021. 21:#pages#

**Kennedy-Shaffer, L.,Baym, M.,Hanage, W. P.**. Perfect as the enemy of good: tracing transmissions with low-sensitivity tests to mitigate SARS-CoV-2 outbreaks. *The Lancet. Microbe.* 2021. 2:e219-e224

**Kennedy-Shaffer, L.,Kahn, R.,Lipsitch, M.**. Estimating vaccine efficacy against transmission via effect on viral load. *Epidemiology.* 2021. 30:#pages#

**Khan, M. S.,Haq, I.,Qurieshi, M. A.,Majid, S.,Bhat, A. A.,Qazi, T. B.,Chowdri, I. N.,Sabah, I.,Kawoosa, M. F.,Lone, A. A.,Nabi, S.,Sumji, I. A.,Obaid, M.,Kousar, R.**. SARS-CoV-2 Seroprevalence Among Healthcare Workers by Workplace Exposure Risk in Kashmir, India. *Journal of Hospital Medicine (Online).* 2021. 16:274-281

**Kim, I.,Lee, J.,Lee, J.,Shin, E.,Chu, C.,Lee, S. K.**. KCDC Risk Assessments on the Initial Phase of the COVID-19 Outbreak in Korea. *Osong Public Health & Research Perspectives.* 2020. 11:67-73

**Kim, J. M.,Park, S. Y.,Lee, D.,Kim, J. S.,Park, Y.,Gwack, J.,Kim, M. Y.,Song, D. H.,Jeong, S. T.,Chung, Y. S.,Yoo, C. K.,Lee, H. Y.,Han, M. G.**. Genomic investigation of the coronavirus disease-2019 outbreak in the Republic of Korea. *Scientific Reports.* 2021. 11:6009

**Kim, N. J.,Choe, P. G.,Park, S. J.,Lim, J.,Lee, W. J.,Kang, C. K.,Park, W. B.,Seong, M. W.,Oh, M. D.**. A cluster of tertiary transmissions of 2019 novel coronavirus (SARS-CoV-2) in the community from infectors with common cold symptoms. *Korean Journal of Internal Medicine.* 2020. 35:758-764

**Klompas, M., Baker, M. A., Griesbach, D., Tucker, R., Gallagher, G. R., Lang, A. S., Fink, T., Cumming, M., Smole, S., Madoff, L. C., Rhee, C.**. Transmission of severe acute respiratory syndrome coronavirus 2 (sars-cov-2) from asymptomatic and presymptomatic individuals in healthcare settings despite medical masks and eye protection. *Clinical Infectious Diseases.* 2021. 73(9):1693-1695

**Kniesburges, S.,Schlegel, P.,Peters, G.,Westphalen, C.,Jakubas, B.,Veltrup, R.,Kist, A. M.,Dollinger, M.,Gantner, S.,Kuranova, L.,Benthaus, T.,Semmler, M.,Echternach, M.**. Effects of surgical masks on aerosol dispersion in professional singing. *Journal of Exposure Science & Environmental Epidemiology.* 2021. 05:05

**Kohn, R., Brown, M., Hasson, C., Sheeran, T., Stanton, L., Nanda, A., Bayer, T.**. COVID-19 and Long-Term Care Healthcare Worker Mental Health in Rhode Island. *American Journal of Geriatric Psychiatry.* 2021. 29(4 Supplement):S101-S102

**Koizumi, N.,Siddique, A. B.,Andalibi, A.**. Assessment of SARS-CoV-2 transmission among attendees of live concert events in Japan using contact-tracing data. *Journal of Travel Medicine.* 2020. 27:20

**Kong, D.,Wu, H.,Pan, H.,Wagner, A. L.,Zheng, Y.,Gong, X.,Zhu, Y.,Jin, B.,Xiao, W.,Mao, S.,Lin, S.,Han, R.,Yu, X.,Cui, P.,Jiang, C.,Fang, Q.,Lu, Y.,Fu, C.**. Pre-symptomatic transmission of novel coronavirus in community settings. *Influenza and other Respiratory Viruses.* 2020. 14(6):610-614

**Kong, I.,Park, Y.,Woo, Y.,Cha, J.,Choi, J.,Yum, M.,Kim, T.,Jo, J.,Kim, M.,Park, S.,Bahk, H.,Yu, J.,Kwon, J.,Shin, N.,In, H.,Jung, J.,Jeon, B.,Kim, D.,Jin, G.,Kim, Y.,Kim, E.,Kim, B. I.,Lee, H.,Jin, Y.,Kim, H.,Ryu, B.,Shin, S.,Shin, E.,Jang, Y.,Choi, E.,Hyun, J.,Ko, D.,Seo, G.,Kwon, S.,Park, E.,Hwang, S.,Lee, S.,Kang, B.,Lee, D.,Jo, S.,Choi, S.,Lee, J.,Cho, S.,Park, J.,Lee, E.,Kim, J.,Kim, S.,Kim, U.**. Early epidemiological and clinical characteristics of 28 cases of coronavirus disease in South Korea. *Osong Public Health and Research Perspectives.* 2020. #volume#:8-14

**Korea Centers for Disease, Control,Prevention,**. Early Trend of Imported COVID-19 Cases in South Korea. *Osong Public Health & Research Perspectives.* 2020. 11:140-145

**Kostaki, E. G.,Pavlopoulos, G. A.,Verrou, K. M.,Ampatziadis-Michailidis, G.,Harokopos, V.,Hatzis, P.,Moulos, P.,Siafakas, N.,Pournaras, S.,Hadjichristodoulou, C.,Chatzopoulou, F.,Chatzidimitriou, D.,Panagopoulos, P.,Lourida, P.,Argyraki, A.,Lytras, T.,Sapounas, S.,Gerolymatos, G.,Panagiotakopoulos, G.,Prezerakos, P.,Tsiodras, S.,Sypsa, V.,Hatzakis, A.,Anastassopoulou, C.,Spanakis, N.,Tsakris, A.,Dimopoulos, M. A.,Kotanidou, A.,Sfikakis, P.,Kollias, G.,Magiorkinis, G.,Paraskevis, D.**. Molecular Epidemiology of SARS-CoV-2 in Greece Reveals Low Rates of Onward Virus Transmission after Lifting of Travel Restrictions Based on Risk Assessment during Summer 2020. *Msphere.* 2021. #volume#:e0018021

**Kreidl, P.,Schmid, D.,Maritschnik, S.,Richter, L.,Borena, W.,Genger, J. W.,Popa, A.,Penz, T.,Bock, C.,Bergthaler, A.,Allerberger, F.**. Emergence of coronavirus disease 2019 (COVID-19) in Austria. *Wiener Klinische Wochenschrift.* 2020. 132:645-652

**Krishna Kumar Nair, G.,Woo Yoo, J.,Vanella, G.,Angeletti, S.,Mauro, A.,Zingone, F.,Toma, A.,Pouillon, L.,Papanikolaou, I. S.,Boskoski, I.,Bronswijk, M. J.,Capurso, G.,Dilaghi, E.,Di Sabatino, A.,Savarino, E.,Fracasso, P.,Matteo, M. V.,Van Der Merwe, S.,Petrone, M.,Di Giulio, E.,Roelandt, P.,Arcidiacono, P. G.,Costamagna, G.,Farrell, J. J.**. Id: 3522469 Risk of Covid-19 Transmission and Outcomes in Healthcare Workers Present during Gastrointestinal Endoscopic Procedures: An International Multicenter Study. *Gastrointestinal Endoscopy.* 2021. 93(6 Supplement):AB45-AB46

**Kwok, C. Y. T.,Wong, M. S.,Chan, K. L.,Kwan, M. P.,Nichol, J. E.,Liu, C. H.,Wong, J. Y. H.,Wai, A. K. C.,Chan, L. W. C.,Xu, Y.,Li, H.,Huang, J.,Kan, Z.**. Spatial analysis of the impact of urban geometry and socio-demographic characteristics on COVID-19, a study in Hong Kong. *Science of the Total Environment.* 2021. 764:144455

**Kwon, K. S., Park, J. I., Park, Y. J., Jung, D. M., Ryu, K. W., Lee, J. H.**. Evidence of Long-Distance Droplet Transmission of SARS-CoV-2 by Direct Air Flow in a Restaurant in Korea. *J Korean Med Sci.* 2020. 35:e415

**La Hoz, R., Agarwal, A., Aslam, S., Dunn, K., Goldman, J., Levine, D., Marboe, C., Marklin, G. F., Pouch, S. M., Rana, M., Razonable, R., Stevenson, H. L., Te, H., Woolley, A. E., Michaels, M., Danziger-Isakov, L.**. COVID-19 potential donor derived transmission events investigations: Early dtac experience. *American Journal of Transplantation.* 2021. 21(SUPPL 4):351

**Lackermair, K.,William, F.,Grzanna, N.,Lehmann, E.,Fichtner, S.,Kucher, H. B.,Wilhelm, K.,Estner, H.**. Infection with SARS-CoV-2 in primary care health care workers assessed by antibody testing. *Family Practice.* 2021. 38:76-79

**Ladner, J. T.,Larsen, B. B.,Bowers, J. R.,Hepp, C. M.,Bolyen, E.,Folkerts, M.,Sheridan, K.,Pfeiffer, A.,Yaglom, H.,Lemmer, D.,Sahl, J. W.,Kaelin, E. A.,Maqsood, R.,Bokulich, N. A.,Quirk, G.,Watts, T. D.,Komatsu, K. K.,Waddell, V.,Lim, E. S.,Caporaso, J. G.,Engelthaler, D. M.,Worobey, M.,Keim, P.**. An Early Pandemic Analysis of SARS-CoV-2 Population Structure and Dynamics in Arizona. *mBio.* 2020. 11:04

**Ladoy, A.,Opota, O.,Carron, P. N.,Guessous, I.,Vuilleumier, S.,Joost, S.,Greub, G.**. Size and duration of COVID-19 clusters go along with a high SARS-CoV-2 viral load: A spatio-temporal investigation in Vaud state, Switzerland. *Science of the Total Environment.* 2021. 787:147483

**Lamarca, A. P., de Almeida, L. G. P., Francisco, R. S., Lima, L. F. A., Scortecci, K. C., Perez, V. P., Brustolini, O. J., Sousa, E. S. S., Secco, D. A., Santos, A. M. G., Albuquerque, G. R., Mariano, A. P. M., Maciel, B. M., Gerber, A. L., Guimaraes, A. P. C., Nascimento, P. R., Neto, F. P. F., Gadelha, S. R., Porto, L. C., Campana, E. H., Jeronimo, S. M. B., Vasconcelos, A. T. R.**. Genomic surveillance of sars-cov-2 tracks early interstate transmission of p.1 lineage and diversification within p.2 clade in brazil. *PLoS Neglected Tropical Diseases.* 2021. 15(10) (no pagination):#pages#

**Lastrucci, V.,Lorini, C.,Riccio, M. D.,Gori, E.,Chiesi, F.,Sartor, G.,Zanella, B.,Boccalini, S.,Bechini, A.,Puggelli, F.,Bonanni, P.,Bonaccorsi, G.**. Sars-cov-2 seroprevalence survey in people involved in different essential activities during the general lock-down phase in the province of prato (Tuscany, italy). *Vaccines.* 2020. 8(4):1-9

**Lauriane, Lenggenhager, Romain, Martischang, Julien, Sauser, Monica, Perez, Laure, Vieux, Christophe, Graf, Samuel, Cordey, Florian, Laubscher, Tomas Robalo, Nunes, Walter, Zingg, Anne, Cori, Stephan, Harbarth, Mohamed, Abbas**. Occupational versus community risk of SARS-CoV-2 infection among employees of a long-term care facility: an observational study. *#journal#.* 2021. #volume#:#pages#

**Lebel, G.,Fortin, E.,Lo, E.,Boivin, M. C.,Tandonnet, M.,Gravel, N.**. Detection of COVID-19 case clusters in Quebec, May-October 2020. *Canadian Journal of Public Health. Revue Canadienne de Sante Publique.* 2021. 09:09

**Lee, L. Y. W.,Rozmanowski, S.,Pang, M.,Charlett, A.,Anderson, C.,Hughes, G. J.,Barnard, M.,Peto, L.,Vipond, R.,Sienkiewicz, A.,Hopkins, S.,Bell, J.,Crook, D. W.,Gent, N.,Walker, A. S.,Peto, T. E. A.,Eyre, D. W.**. SARS-CoV-2 infectivity by viral load, S gene variants and demographic factors and the utility of lateral flow devices to prevent transmission. *Clinical infectious diseases : an official publication of the Infectious Diseases Society of America..* 2021. 11:#pages#

**Leitao, I. C., Calil, P. T., Galliez, R. M., Moreira, F. R. R., Mariani, D., Castineiras, A. C. P., da Silva, G. P. D., Maia, R. A., Correa, I. A., Monteiro, F. L. L., de Souza, M. R. M., Goncalves, C. C. A., Higa, L. M., de Jesus Ribeiro, L., Fonseca, V. W. P., Bastos, V. C., Voloch, C. M., Faffe, D. S., da Costa Ferreira, O., Jr., Tanuri, A., Castineiras, Tmpp, da Costa, L. J., on the behalf of the, Ufrj-Covid Workgroup**. Prolonged SARS-CoV-2 Positivity in Immunocompetent Patients: Virus Isolation, Genomic Integrity, and Transmission Risk. *Microbiology Spectrum.* 2021. #volume#:e0085521

**Li, C.,Ji, F.,Wang, L.,Wang, L.,Hao, J.,Dai, M.,Liu, Y.,Pan, X.,Fu, J.,Li, L.,Yang, G.,Yang, J.,Yan, X.,Gu, B.**. Asymptomatic and Human-to-Human Transmission of SARS-CoV-2 in a 2-Family Cluster, Xuzhou, China. *Emerging Infectious Diseases.* 2020. 26:1626-1628

**Li, J.,Gong, X.,Wang, Z.,Chen, R.,Li, T.,Zeng, D.,Li, M.**. Clinical features of familial clustering in patients infected with 2019 novel coronavirus in Wuhan, China. *Virus Research.* 2020. 286:198043

**Lim, J. S.,Noh, E.,Shim, E.,Ryu, S.**. Temporal Changes in the Risk of Superspreading Events of Coronavirus Disease 2019. *Open Forum Infectious Diseases.* 2021. 8:ofab350

**Lin, S.,Pan, H.,Wu, H.,Yu, X.,Cui, P.,Han, R.,Jiang, C.,Kong, D.,Zheng, Y.,Gong, X.,Xiao, W.,Mao, S.,Jin, B.,Zhu, Y.,Sun, X.**. Epidemiological and clinical characteristics of 161 discharged cases with coronavirus disease 2019 in Shanghai, China. *BMC Infectious Diseases.* 2020. 20(1) (no pagination):#pages#

**Liu, L.**. Emerging study on the transmission of the Novel Coronavirus (COVID-19) from urban perspective: Evidence from China. *Cities.* 2020. 103:102759

**Liu, S.,Qin, Y.,Xie, Z.,Zhang, J.**. The Spatio-Temporal Characteristics and Influencing Factors of Covid-19 Spread in Shenzhen, China-An Analysis Based on 417 Cases. *International Journal of Environmental Research & Public Health [Electronic Resource].* 2020. 17:13

**Liu, Y., Liu, J., Plante, K. S., Plante, J. A., Xie, X., Zhang, X., Ku, Z., An, Z., Scharton, D., Schindewolf, C., Widen, S. G., Menachery, V. D., Shi, P. Y., Weaver, S. C.**. The N501Y spike substitution enhances SARS-CoV-2 infection and transmission. *Nature..* 2021. #volume#:#pages#

**Llueca, A., Barneo-Munoz, M., Escrig, J., de Llanos, R.**. Sars-cov-2 prevalence in laparoscopic surgery filters. Analysis in patients with negative oropharyngeal rt-qpcr in a pandemic context: A cross-sectional study<sup>+</sup>. *Journal of Personalized Medicine.* 2021. 11(11) (no pagination):#pages#

**Loth, A. G.,Guderian, D. B.,Haake, B.,Zacharowski, K.,Stover, T.,Leinung, M.**. Aerosol Exposure During Surgical Tracheotomy in SARS-CoV-2 Positive Patients. *Shock.* 2021. 55:472-478

**Lunn, P. D.,Timmons, S.,Belton, C. A.,Barjakova, M.,Julienne, H.,Lavin, C.**. Motivating social distancing during the COVID-19 pandemic: An online experiment. *Social Science & Medicine.* 2020. 265:113478

**Ma, H.,Wang, Z.,Zhao, X.,Han, J.,Zhang, Y.,Wang, H.,Chen, C.,Wang, J.,Jiang, F.,Lei, J.,Song, J.,Jiang, S.,Zhu, S.,Liu, H.,Wang, D.,Meng, Y.,Mao, N.,Wang, Y.,Zhu, Z.,Chen, Z.,Wang, B.,Song, Q.,Du, H.,Yuan, Q.,Xia, D.,Xia, Z.,Liu, P.,Wu, Y.,Feng, Z.,Gao, R.,Gao, G. F.,Xu, W.**. Long Distance Transmission of SARS-CoV-2 from Contaminated Cold Chain Products to Humans - Qingdao City, Shandong Province, China, September 2020. *China CDC Weekly.* 2021. 3:637-644

**Malheiro, R.,Figueiredo, A. L.,Magalhaes, J. P.,Teixeira, P.,Moita, I.,Moutinho, M. C.,Mansilha, R. B.,Goncalves, L. M.,Ferreira, E.**. Effectiveness of contact tracing and quarantine on reducing COVID-19 transmission: a retrospective cohort study. *Public Health.* 2020. 189:54-59

**Manouana, G. P., Nzamba Maloum, M., Bikangui, R., Oye Bingono, S. O., Ondo Nguema, G., Honkpehedji, J. Y., Rossatanga, E. G., Zoa-Assoumou, S., Pallerla, S. R., Rachakonda, S., Ndong Mintsa, A., Lekana-Douki, J. B., Djoba Siawaya, J. F., Borrmann, S., Kremsner, P. G., Lell, B., Velavan, T. P., Adegnika, A. A.**. Emergence of B.1.1.318 SARS-CoV-2 viral lineage and high incidence of alpha B.1.1.7 variant of concern in the Republic of Gabon. *International Journal of Infectious Diseases.* 2022. 114:151-154

**Margiotti, K.,Fabiani, M.,Cupellaro, M.,Mesoraca, A.,Giorlandino, C.**. Retrospective observational study of the environmental impact in SARS-CoV-2 virus transmission based on exclusion Criteria. *American Journal of Infectious Diseases.* 2021. 17(1):1-4

**Mashrur, F. R.,Roy, A. D.,Chhoan, A. P.,Sarker, S.,Saha, A.,Hasan, S. M. N.,Saha, S.**. Impact of demographic, environmental, socioeconomic, and government intervention on the spreading of COVID-19. *Clinical Epidemiology and Global Health.* 2021. 12 (no pagination):#pages#

**Mendis, D. M.,Perera, T. P. W.,Manoj, S. S.,Iresha, G. K.,Buddhadasa, P. C. L. S.,Hansani, H. C. L.,Jayatilleke, K.**. Assessing risk factors for health care associated transmission of SARS-Cov-2 virus infection among health care personnel in a tertiary care hospital, Sri Lanka; a case-control study. *International Journal of Antimicrobial Agents.* 2021. 58(Supplement 1) (no pagination):#pages#

**Meng, T.**. Clusters in the Spread of the COVID-19 Pandemic: Evidence From the G20 Countries. *Frontiers in Public Health.* 2020. 8:628789

**Meyers, K. J.,Dillman, B.,Williams, C.,Jiang, J.,Clifford, N.,Miller, J. L.,Jones, M. E.,Goetz, I. A.,Botros, F. T.,Knorr, J.,Manner, D. H.,Woodward, B.**. Follow-up of SARS-CoV-2 positive subgroup from the Asymptomatic novel CORonavirus iNFection study. *Journal of Medical Virology.* 2021. 93:2925-2931

**Micheli, V.,Rimoldi, S. G.,Romeri, F.,Comandatore, F.,Mancon, A.,Gigantiello, A.,Perini, M.,Mileto, D.,Pagani, C.,Lombardi, A.,Gismondo, M. R.**. Geographical reconstruction of the SARS-CoV-2 outbreak in Lombardy (Italy) during the early phase. *Journal of Medical Virology.* 2021. 93:1752-1757

**Migisha, R.,Kwesiga, B.,Mirembe, B. B.,Amanya, G.,Kabwama, S. N.,Kadobera, D.,Bulage, L.,Nsereko, G.,Wadunde, I.,Tindyebwa, T.,Lubwama, B.,Kagirita, A. A.,Kayiwa, J. T.,Lutwama, J. J.,Boore, A. L.,Harris, J. R.,Bosa, H. K.,Ario, A. R.**. Early cases of SARS-CoV-2 infection in Uganda: epidemiology and lessons learned from risk-based testing approaches - March-April 2020. *Global Health.* 2020. 16:114

**Mohindra, R.,Ghai, A.,Brar, R.,Khandelwal, N.,Biswal, M.,Suri, V.,Goyal, K.,Singh, M. P.,Bhalla, A.,Rana, K.,Lakshmi, P. V. M.**. Superspreaders: A Lurking Danger in the Community. *Journal of Primary Care & Community Health.* 2021. 12:2150132720987432

**Molaeb, B. S.,Saad, M.,Almoosa, Z.**. Clustering of COVID-19 infections among healthcare workers: Experience from a tertiary care center in Saudi Arabia. *Antimicrobial Resistance and Infection Control. Conference: 6th International Conference on Prevention and Infection Control, ICPIC.* 2021. 10:#pages#

**Monge, M., Abdel-Hady, A., Aslett, L. D., Calfee, M. W., Williams, B., Ratliff, K., Ryan, S., Oudejans, L., Touati, A.**. Inactivation of MS2 bacteriophage on copper film deployed in high touch areas of a public transport system. *Letters in Applied Microbiology.* 2021. 04:04

**Moniz, M.,Soares, P.,Nunes, C.**. COVID-19 Transmission Dynamics: A Space-and-Time Approach. *Portuguese Journal of Public Health.* 2021. 38(1):4-10

**Moreno, G. K.,Braun, K. M.,Riemersma, K. K.,Martin, M. A.,Halfmann, P. J.,Crooks, C. M.,Prall, T.,Baker, D.,Baczenas, J. J.,Heffron, A. S.,Ramuta, M.,Khubbar, M.,Weiler, A. M.,Accola, M. A.,Rehrauer, W. M.,O'Connor, S. L.,Safdar, N.,Pepperell, C. S.,Dasu, T.,Bhattacharyya, S.,Kawaoka, Y.,Koelle, K.,O'Connor, D. H.,Friedrich, T. C.**. Distinct patterns of SARS-CoV-2 transmission in two nearby communities in Wisconsin, USA. *MedRxiv : the Preprint Server for Health Sciences.* 2020. 10:10

**Mortezagholi, S.,Rostamzadeh, D.,Alinejad, M.,Younesi, V.,Tabarsi, P.,Shabani, M.**. Prevalence of Anti-SARS-CoV-2 Specific Antibodies in Health-Care Workers Compared to General Population during an Early Phase of the Pandemic, Tehran-Iran. *Iranian Journal Of Immunology: IJI.* 2021. 18:82-92

**Mouliou, D. S.,Kotsiou, O. S.,Gourgoulianis, K. I.**. Estimates of COVID-19 Risk Factors among Social Strata and Predictors for a Vulnerability to the Infection. *International Journal of Environmental Research & Public Health [Electronic Resource].* 2021. 18:18

**Mun, E.,Kim, Y. M.,Han, B.,Jeong, J.,Kim, W.,Lee, C.**. A case series of flight attendants at risk of COVID-19 in South Korea in 2020. *Annals of Occupational and Environmental Medicine.* 2021. 33 (no pagination):#pages#

**Murewanhema, G.**. COVID-19 control pitfalls and challenges and drivers of SARS-CoV-2 transmission in Zimbabwe. *The Pan African medical journal.* 2021. 38:28

**Murr, A. T.,Lenze, N. R.,Gelpi, M. W.,Brown, W. C.,Ebert, C. S., Jr.,Senior, B. A.,Thorp, B. D.,Kimple, A. J.,Zanation, A. M.**. Quantification of Aerosol Concentrations During Endonasal Instrumentation in the Clinic Setting. *Laryngoscope.* 2021. 131:E1415-E1421

**Nanda, M., Aashima, Sharma, R.**. Review of COVID-19 epidemiology and public health response in Europe in 2020. *Clinical Epidemiology and Global Health.* 2021. 12 (no pagination):#pages#

**Nashed, L., Mani, J., Hazrati, S., Richards, T., Nerikar, N., Ravi, S., Mattei, L., Levy, S., Maxwell, G., Hourigan, S.**. SARS-CoV-2 prevalence in feces of very young children, a longitudinal study. *Journal of Pediatric Gastroenterology and Nutrition.* 2021. 73(1 SUPPL 1):S140-S141

**Ngwira, A.,Kumwenda, F.,Munthali, E. C. S.,Nkolokosa, D.**. Spatial temporal distribution of COVID-19 risk during the early phase of the pandemic in Malawi. *PeerJ.* 2021. 9:e11003

**Nixon, E., Thomas, A., Stocks, D., Barreaux, A. M. G., Hemani, G., Trickey, A., Kwiatkowska, R., Walker, J., Ellis, D., Danon, L., Relton, C., Christensen, H., Brooks-Pollock, E.**. Impacts of vaccination and asymptomatic testing on SARS-CoV-2 transmission dynamics in a university setting. *medRxiv..* 2021. 24:#pages#

**Nordstrom, P.,Ballin, M.,Nordstrom, A.**. Association between Risk of COVID-19 Infection in Nonimmune Individuals and COVID-19 Immunity in Their Family Members. *JAMA Internal Medicine..* 2021. #volume#:#pages#

**Oliva, C.,Favato, G.**. Risk of Exposure to COVID-19: Visit Duration Data Can Inform Our Daily Activities Choices: An Epidemiological Investigation Using Community Mobility Data from the Metropolitan Area of Genoa, Italy. *International Journal of Environmental Research & Public Health [Electronic Resource].* 2021. 18:27

**Oster, A. M.,Kang, G. J.,Cha, A. E.,Beresovsky, V.,Rose, C. E.,Rainisch, G.,Porter, L.,Valverde, E. E.,Peterson, E. B.,Driscoll, A. K.,Norris, T.,Wilson, N.,Ritchey, M.,Walke, H. T.,Rose, D. A.,Oussayef, N. L.,Parise, M. E.,Moore, Z. S.,Fleischauer, A. T.,Honein, M. A.,Dirlikov, E.,Villanueva, J.**. Trends in Number and Distribution of COVID-19 Hotspot Counties - United States, March 8-July 15, 2020. *MMWR - Morbidity & Mortality Weekly Report.* 2020. 69:1127-1132

**Ozyigit, A.**. Understanding Covid-19 transmission: The effect of temperature and health behavior on transmission rates. *Infection, Disease and Health.* 2020. 25(4):233-238

**Paris, Assistance Publique - Hôpitaux de, PRODISS, WEEZEVENT**. Study on Prevention of SARS-CoV-2 Transmission During a Large Indoor Gathering Event. *#journal#.* 2021. #volume#:#pages#

**Paskarini, I.,Haqi, D. N.,Arini, S. Y.,Dwiyanti, E.,Alayyannur, P. A.**. Analysis of risk of exposure to COVID-19 in fishermen in Kenjeran. *Systematic Reviews in Pharmacy.* 2020. 11(11):1643-1648

**Patel, R. K.,Shackelford, I. A.,Priddy, M. C.,Kopechek, J. A.**. Effect of speech volume on respiratory emission of oral bacteria as a potential indicator of pathogen transmissibility risk. *Journal of the Acoustical Society of America.* 2020. 148:2322

**Peirlinck, M.,Linka, K.,Sahli Costabal, F.,Kuhl, E.**. Outbreak dynamics of COVID-19 in China and the United States. *Biomechanics and Modeling in Mechanobiology.* 2020. 19(6):2179-2193

**Piana, A.,Colucci, M. E.,Valeriani, F.,Marcolongo, A.,Sotgiu, G.,Pasquarella, C.,Margarucci, L. M.,Petrucca, A.,Gianfranceschi, G.,Babudieri, S.,Vitali, P.,D'Ermo, G.,Bizzarro, A.,De Maio, F.,Vitali, M.,Azara, A.,Romano, F.,Simmaco, M.,Romano Spica, V.**. Monitoring COVID-19 Transmission Risks by Quantitative Real-Time PCR Tracing of Droplets in Hospital and Living Environments. *Msphere.* 2021. 6:06

**Poletti, P.,Tirani, M.,Cereda, D.,Trentini, F.,Guzzetta, G.,Sabatino, G.,Marziano, V.,Castrofino, A.,Grosso, F.,Del Castillo, G.,Piccarreta, R.,Andreassi, A.,Melegaro, A.,Gramegna, M.,Ajelli, M.,Merler, S.,A. T. S. Lombardy COVID-19 Task Force**. Association of Age With Likelihood of Developing Symptoms and Critical Disease Among Close Contacts Exposed to Patients With Confirmed SARS-CoV-2 Infection in Italy. *JAMA Network Open.* 2021. 4:e211085

**Pomara, C.,Salerno, M.,Sessa, F.,Esposito, M.,Barchitta, M.,Ledda, C.,Grassi, P.,Liberto, A.,Mattaliano, A. R.,Rapisarda, V.,Ferrante, M.,Agodi, A.**. Safe management strategies in clinical forensic autopsies of confirmed COVID-19 cases. *Diagnostics.* 2021. 11(3) (no pagination):#pages#

**Pramanik, M.,Udmale, P.,Bisht, P.,Chowdhury, K.,Szabo, S.,Pal, I.**. Climatic factors influence the spread of COVID-19 in Russia. *International Journal of Environmental Health Research.* 2020. #volume#:1-16

**Pranata, A.,Zulkifli, B.,Santosa, S. F.,Oktiviyari, A.,Zulfitri, Z.,Hayati, Z.,Mudatsir, M.,Ichsan, I.,Harapan, H.**. Discordant results of SARS-CoV-2 PCR-based tests in the early phase of pandemic in Indonesia: Infection control consequences. *Vacunas..* 2021. #volume#:#pages#

**Pribadi, D. O.,Saifullah, K.,Putra, A. S.,Nurdin, M.,Iman, L. O. S.,Rustiadi, E.**. Spatial analysis of COVID-19 outbreak to assess the effectiveness of social restriction policy in dealing with the pandemic in Jakarta. *Spatial and Spatio-temporal Epidemiology.* 2021. 39 (no pagination):#pages#

**Qin, W.,Sun, J.,Xu, P.,Gong, T.,Li, X.,Liu, L.,Hu, J.,Wang, Y.,Xie, S.,Li, K.,Chang, H.,Lyu, Y.**. The descriptive epidemiology of coronavirus disease 2019 during the epidemic period in Lu'an, China: achieving limited community transmission using proactive response strategies. *Epidemiology & Infection.* 2020. 148:e132

**Qiu, C.,Deng, Z.,Xiao, Q.,Shu, Y.,Deng, Y.,Wang, H.,Liao, X.,Liu, H.,Zhou, D.,Zhao, X.,Zhou, J.,Wang, J.,Shi, Z.,Long, D.**. Transmission and clinical characteristics of coronavirus disease 2019 in 104 outside-Wuhan patients, China. *Journal of Medical Virology.* 2020. 92:2027-2035

**Quach, H. L.,Hoang, N. T.,Nguyen, C. K.,Pham, Q. T.,Phung, C. D.,Tran, N. D.,Le, Q. M. T.,Ngu, D. N.,Tran, A. T.,La, N. Q.,Tran, D. Q.,Nguyen, T. T.,Vogt, F.,Dang, D. A.**. Successful containment of a flight-imported COVID-19 outbreak through extensive contact tracing, systematic testing and mandatory quarantine: Lessons from Vietnam. *Travel Medicine & Infectious Disease.* 2021. 42:102084

**Queromes, G.,Destras, G.,Bal, A.,Regue, H.,Burfin, G.,Brun, S.,Fanget, R.,Morfin, F.,Valette, M.,Trouillet-Assant, S.,Lina, B.,Frobert, E.,Josset, L.**. Characterization of SARS-CoV-2 ORF6 deletion variants detected in a nosocomial cluster during routine genomic surveillance, Lyon, France. *Emerging Microbes & Infections.* 2021. 10:167-177

**Rahi, M.,Le Pluart, D.,Beaudet, A.,Ismael, S.,Parisey, M.,Poey, N.,Tarhini, H.,Lescure, F. X.,Yazdanpanah, Y.,Deconinck, L.**. Sociodemographic characteristics and transmission risk factors in patients hospitalized for COVID-19 before and during the lockdown in France. *BMC Infectious Diseases.* 2021. 21:812

**Ranzani, O. T.,Hitchings, M. D. T.,Dorion, M.,D'Agostini, T. L.,de Paula, R. C.,de Paula, O. F. P.,Villela, E. F. M.,Torres, M. S. S.,de Oliveira, S. B.,Schulz, W.,Almiron, M.,Said, R.,de Oliveira, R. D.,Vieira da Silva, P.,de Araujo, W. N.,Gorinchteyn, J. C.,Andrews, J. R.,Cummings, D. A. T.,Ko, A. I.,Croda, J.**. Effectiveness of the CoronaVac vaccine in older adults during a gamma variant associated epidemic of covid-19 in Brazil: test negative case-control study. *BMJ.* 2021. 374:n2015

**Rashed, E. A.,Kodera, S.,Gomez-Tames, J.,Hirata, A.**. Influence of Absolute Humidity, Temperature and Population Density on COVID-19 Spread and Decay Durations: Multi-Prefecture Study in Japan. *International Journal of Environmental Research & Public Health [Electronic Resource].* 2020. 17:24

**Redditt, V.,Wright, V.,Rashid, M.,Male, R.,Bogoch, I.**. Outbreak of SARS-CoV-2 infection at a large refugee shelter in Toronto, April 2020: a clinical and epidemiologic descriptive analysis. *CMAJ open.* 2020. 8:E819-E824

**Riou, J.,Althaus, C. L.**. Pattern of early human-to-human transmission of Wuhan 2019 novel coronavirus (2019-nCoV), December 2019 to January 2020. *Eurosurveillance.* 2020. 25(4) (no pagination):#pages#

**Rodriguez-Diaz, C. E.,Guilamo-Ramos, V.,Mena, L.,Hall, E.,Honermann, B.,Crowley, J. S.,Baral, S.,Prado, G. J.,Marzan-Rodriguez, M.,Beyrer, C.,Sullivan, P. S.,Millett, G. A.**. Risk for COVID-19 infection and death among Latinos in the United States: examining heterogeneity in transmission dynamics. *Annals of Epidemiology.* 2020. 52:46-53.e2

**Romano-Bertrand, S.,Aho Glele, L. S.,Grandbastien, B.,Lepelletier, D.**. Preventing SARS-CoV-2 transmission in rehabilitation pools and therapeutic water environments. *Journal of Hospital Infection.* 2020. 105(4):625-627

**Ruthberg, J. S.,Quereshy, H. A.,Jella, T. K.,Kocharyan, A.,D'Anza, B.,Maronian, N.,Otteson, T. D.**. Geospatial analysis of COVID-19 and otolaryngologists above age 60. *American Journal of Otolaryngology - Head and Neck Medicine and Surgery.* 2020. 41(4) (no pagination):#pages#

**S. ARS-CoV-2 variant with lineage B.1.351 clusters investigation team,Members of the, Sars-CoV-variant with lineage B. clusters investigation team**. Linked transmission chains of imported SARS-CoV-2 variant B.1.351 across mainland France, January 2021. *Euro Surveillance: Bulletin Europeen sur les Maladies Transmissibles = European Communicable Disease Bulletin.* 2021. 26:04

**Saadatian-Elahi, M.,Picot, V.,Henaff, L.,Pradel, F. K.,Escuret, V.,Dananche, C.,Elias, C.,Endtz, H. P.,Vanhems, P.**. Protocol for a prospective, observational, hospital-based multicentre study of nosocomial SARS-CoV-2 transmission: NOSO-COR Project. *BMJ Open.* 2020. 10:e039088

**Sagara, I.,Woodford, J.,Kone, M.,Assadou, M. H.,Katile, A.,Attaher, O.,Zeguime, A.,Doucoure, M.,Higbee, E.,Lane, J.,Mohan, R.,Doritchamou, J.,Zaidi, I.,Esposito, D.,Kwan, J.,Sadtler, K.,Dicko, A.,Duffy, P. E.**. Rapidly increasing SARS-CoV-2 seroprevalence and limited clinical disease in three Malian communities: a prospective cohort study. *Clinical Infectious Diseases.* 2021. 29:29

**San, J. E.,Ngcapu, S.,Kanzi, A. M.,Tegally, H.,Fonseca, V.,Giandhari, J.,Wilkinson, E.,Nelson, C. W.,Smidt, W.,Kiran, A. M.,Chimukangara, B.,Pillay, S.,Singh, L.,Fish, M.,Gazy, I.,Martin, D. P.,Khanyile, K.,Lessells, R.,de Oliveira, T.**. Transmission dynamics of SARS-CoV-2 within-host diversity in two major hospital outbreaks in South Africa. *Virus Evolution.* 2021. 7:veab041

**Sarkar, B.,Sinha, R. N.,Sarkar, K.**. Initial Viral Load of a COVID-19-Infected Case Indicated by its Cycle Threshold Value of Polymerase Chain Reaction Could be used as a Predictor of its Transmissibility - An Experience from Gujarat, India. *Indian Journal of Community Medicine.* 2020. 45:278-282

**Saskatchewan, University of, Foundation, Saskatchewan Health Research**. The Effects of Wearing a Face Mask During Exercise in Youth Hockey Players During COVID-19. *#journal#.* 2021. #volume#:#pages#

**Schnake-Mahl, Alina S., O’Leary, Gabriella, Mullachery, Pricila H., Vaidya, Vaishnavi, Connor, Gabrielle, Rollins, Heather, Kolker, Jennifer, Diez Roux, Ana V., Bilal, Usama**. Evaluating the impact of keeping indoor dining closed on COVID-19 rates among large US cities: a quasi-experimental design. *medRxiv.* 2021. #volume#:2021.04.12.21251656

**Schreiber, S., Faude, O., Gartner, B., Meyer, T., Egger, F.**. Risk of SARS-CoV-2 transmission from on-field player contacts in amateur, youth and professional football (soccer). *British journal of sports medicine.* 2022. 56(3):158-164

**Schrock, J. M.,Ryan, D. T.,Saber, R.,Benbow, N.,Vaught, L. A.,Reiser, N.,Velez, M. P.,Hsieh, R.,Newcomb, M.,Demonbreun, A. R.,Mustanski, B.,McNally, E. M.,D'Aquila, R.,McDade, T. W.**. Cohabitation with a Known Coronavirus Disease 2019 Case Is Associated with Greater Antibody Concentration and Symptom Severity in a Community-Based Sample of Seropositive Adults. *Open Forum Infectious Diseases.* 2021. 8(7) (no pagination):#pages#

**Setti, L.,Passarini, F.,De Gennaro, G.,Barbieri, P.,Licen, S.,Perrone, M. G.,Piazzalunga, A.,Borelli, M.,Palmisani, J.,D. I. Gilio A,Rizzo, E.,Colao, A.,Piscitelli, P.,Miani, A.**. Potential role of particulate matter in the spreading of COVID-19 in Northern Italy: First observational study based on initial epidemic diffusion. *BMJ Open.* 2020. 10(9) (no pagination):#pages#

**Shah, A. S. V., Gribben, C., Bishop, J., Hanlon, P., Caldwell, D., Wood, R., Reid, M., McMenamin, J., Goldberg, D., Stockton, D., Hutchinson, S., Robertson, C., McKeigue, P. M., Colhoun, H. M., McAllister, D. A.**. Effect of vaccination on transmission of SARS-CoV-2. *New England Journal of Medicine.* 2021. 385(18):1718-1720

**Shah, M. R.,Jan, I.,Johns, J.,Singh, K.,Kumar, P.,Belarmino, N.,Saggiomo, K.,Hayes, C.,Toppmeyer, D.,Washington, K.,Haffty, B.,Libutti, S.,Evens, A. M.**. Environmental Sars-Cov-2 Surface Testing: Low Incidence of Virus Positivity in Outpatient and Inpatient Hematology/Oncology Settings. *Blood.* 2020. 136(Supplement 1):24-25

**Shakiba, M.,Nazemipour, M.,Heidarzadeh, A.,Mansournia, M. A.**. Prevalence of asymptomatic COVID-19 infection using a seroepidemiological survey. *Epidemiology & Infection.* 2020. 148:e300

**Shapiro Ben David, S.,Rahamim-Cohen, D.,Tasher, D.,Geva, A.,Azuri, J.,Ash, N.**. COVID-19 in children and the effect of schools reopening on potential transmission to household members. *Acta Paediatrica, International Journal of Paediatrics.* 2021. 110(9):2567-2573

**Sharif, N., Alzahrani, K. J., Ahmed, S. N., Opu, R. R., Ahmed, N., Talukder, A., Nunia, R., Chowdhury, M. S., Nodi, I. J., Saha, T., Zhang, M., Dey, S. K.**. Protective measures are associated with the reduction of transmission of COVID-19 in Bangladesh: A nationwide cross-sectional study. *PLoS ONE.* 2021. 16(11 November) (no pagination):#pages#

**Shehatta, A. L.,Racela, B.,Howard, I.,Alinier, G.,Jaouni, H.,Hassan, I.**. Safety of healthcare workers undertaking transport and retrieval of patients on extracorporeal membrane oxygenation during the peak of COVID-19 pandemic in the State of Qatar. *Journal of Emergency Medicine, Trauma and Acute Care.* 2021. 2021(2) (no pagination):#pages#

**Shi, F.,Wen, H.,Liu, R.,Bai, J.,Wang, F.,Mubarik, S.,Liu, X.,Yu, Y.,Hong, Q.,Cao, J.,Yu, C.**. The comparison of epidemiological characteristics between confirmed and clinically diagnosed cases with COVID-19 during the early epidemic in Wuhan, China. *Global Health Research and Policy.* 2021. 6:18

**Shi, J.,Wen, Z.,Zhong, G.,Yang, H.,Wang, C.,Huang, B.,Liu, R.,He, X.,Shuai, L.,Sun, Z.,Zhao, Y.,Liu, P.,Liang, L.,Cui, P.,Wang, J.,Zhang, X.,Guan, Y.,Tan, W.,Wu, G.,Chen, H.,Bu, Z.**. Susceptibility of ferrets, cats, dogs, and other domesticated animals to SARS-coronavirus 2. *Science.* 2020. 368(6494):1016-1020

**Shirreff, G.,Zahar, J. R.,Temime, L.,Opatowski, L.**. Estimating SARS-COV-2 transmission and efficacy of preventive measures in a long-term care facility. *Antimicrobial Resistance and Infection Control. Conference: 6th International Conference on Prevention and Infection Control, ICPIC.* 2021. 10:#pages#

**Simon, Craxford, Jessica, Nightingale, Adeel, Ikram, Ben Arthur, Marson, Anthony, Kelly, Alan, Norrish, Amrita, Vijay, Stuart, Astbury, Lola, Cusin, Waheed, Ashraf, Jayne, Newham, Guruprasad, Aithal, Patrick, Tighe, Jonathan, Ball, Alexander, W. Tarr, Richard, A. Urbanowicz, Ana, Valdes, Benjamin, Ollivere**. SARS-CoV-2 transmission from the healthcare setting into the home: a prospective longitudinal cohort study. *#journal#.* 2021. #volume#:#pages#

**Singhal, S., Warren, C., Hobin, E., Smith, B.**. How Often Are Dental Care Workers Exposed to Occupational Characteristics that Put Them at Higher Risk of Exposure and Transmission of COVID-19? A Comparative Analysis. *Journal (Canadian Dental Association).* 2021. 87:l16

**Sitorus, R. J.,Wathan, I.,Ridwan, H.,Wibisono, H.,Nuraini, L.,Yusri,,Kosim, G.,Nurdin, N.,Mamat, H.,Andrayani, I.,Antara, N. Y.,Natalia, M.**. Transmission dynamics of novel Coronavirus-SARS-CoV-2 in South Sumatera, Indonesia. *Clinical Epidemiology and Global Health.* 2021. 11 (no pagination):#pages#

**Sjaarda, C. P.,Rustom, N.,Evans, G. A.,Huang, D.,Perez-Patrigeon, S.,Hudson, M. L.,Wong, H.,Sun, Z.,Guan, T. H.,Ayub, M.,Soares, C. N.,Colautti, R. I.,Sheth, P. M.**. Phylogenomics reveals viral sources, transmission, and potential superinfection in early-stage COVID-19 patients in Ontario, Canada. *Scientific Reports.* 2021. 11:3697

**Sockrider, M.,Krishnan, V.**. Socializing during the COVID-19 Pandemic. *American Journal of Respiratory and Critical Care Medicine.* 2021. 203(1):P3-P4

**Sohail, M. A., Hanane, T., Lane, J. E., Vachharajani, T. J.**. Safety and efficacy of bedside insertion of tunneled hemodialysis catheters in critically ill patients with COVID-19. *Journal of the American Society of Nephrology.* 2021. 32:767

**Song, H.,McKenna, R.,Chen, A. T.,David, G.,Smith-McLallen, A.**. The impact of the non-essential business closure policy on Covid-19 infection rates. *International Journal of Health Economics and Management.* 2021. 01:01

**Song, Y.,Ge, Z.,Cui, S.,Tian, D.,Wan, G.,Zhu, S.,Wang, X.,Wang, Y.,Zhao, X.,Xiang, P.,Xu, Y.,Zhang, T.,Liu, L.,Liu, G.,Wang, Y.,Tan, J.,Zhang, W.,Xu, W.,Chen, Z.**. COVID-19 Cases from the First Local Outbreak of the SARS-CoV-2 B.1.1.7 Variant in China May Present More Serious Clinical Features: A Prospective, Comparative Cohort Study. *Microbiology Spectrum.* 2021. 9:e0027321

**Sousa, P.,Da Costa, N. M.,Da Costa, E. M.,Rocha, J.,Ricoca Peixoto, V.,Campos Fernandes, A.,Gaspar, R.,Duarte-Ramos, F.,Abrantes, P.,Leite, A.**. COMPRIME - COnhecer Mais PaRa Intervir MElhor: Preliminary Mapping of Municipal Level Determinants of COVID-19 Transmission in Portugal at Different Moments of the 1st Epidemic Wave. *Portuguese Journal of Public Health.* 2021. 38(1):18-25

**Southall, E.,Holmes, A.,Hill, E. M.,Atkins, B. D.,Leng, T.,Thompson, R. N.,Dyson, L.,Keeling, M. J.,Tildesley, M. J.**. An analysis of school absences in England during the COVID-19 pandemic. *BMC Medicine.* 2021. 19(1) (no pagination):#pages#

**Spaccaferri, G.,Calba, C.,Vilain, P.,Garras, L.,Durand, C.,Pilorget, C.,Atiki, N.,Bernillon, P.,Bosc, L.,Fougere, E.,Hanon, J. B.,Henry, V.,Huchet-Kervella, C.,Martel, M.,Ponties, V.,Mouly, D.,Rolland du Roscoat, E.,Le Vu, S.,Desenclos, J. C.,Laporte, A.,Regional, Monic group,Rolland, P.**. COVID-19 hotspots through clusters analysis in France (may-October 2020): where should we track the virus to mitigate the spread?. *BMC Public Health.* 2021. 21:1834

**Spoelder, E. J.,Tacken, M. C. T.,van Geffen, G. J.,Slagt, C.**. Helicopter transport of critical care COVID-19 patients in the Netherlands: protection against COVID-19 exposure-a challenge to critical care retrieval personnel in a novel operation. *Scandinavian Journal of Trauma, Resuscitation & Emergency Medicine.* 2021. 29:41

**Steven, J. Krieg, Jennifer, J. Schnur, Marie, L. Miranda, Michael, E. Pfrender, Nitesh, V. Chawla**. Symptomatic, Presymptomatic, and Asymptomatic Transmission of SARS-CoV-2. *#journal#.* 2021. #volume#:#pages#

**Straif, K., Espinosa, A., Vinyals, G. C., Karachaliou, M., Alba Hidalgo, M. A., Papantoniou, K., Garcia-Aymerich, J., De Cid, R., Goldberg, X., Kogevinas, M.**. Occupational risk factors for SARS-COV-2 infection and COVID-19: Results from the covicat cohort study in Catalonia, Spain. *Occupational and Environmental Medicine.* 2021. 78(SUPPL 1):A12

**Sudheer, P.,Vibha, D.,Padma Srivastava, M.,Tripathi, M.,Kumar Srivastava, A.,Bhatia, R.,Singh, M.,Vishnu, V. Y.,Rajan, R.,Pandit, A.,Singh, R.,Das, A.,Gupta, A.,Elavarasi, A.,Mr, D.,Aggarwal, A.,Ramanujam, B.**. Impact of COVID-19 pandemic on incidence of health-care associated infections in neurology intensive care unit (ICU) and its associated mortality - A retrospective cohort study. *Journal of the Neurological Sciences.* 2021. Conference: World Congress of Neurology:#pages#

**Sun, Y.,Yang, P.,Wang, Q.,Zhang, L.,Duan, W.,Pan, Y.,Wu, S.,Wang, H.**. Influenza vaccination and non-pharmaceutical measure effectiveness for preventing influenza outbreaks in schools: A surveillance-based evaluation in Beijing. *Vaccines.* 2020. 8(4):1-10

**Syed, A. A. O.,Jahan, S.,Aldahlawi, A. A.,Alghazzawi, E. A.**. Preventive Practices of Ophthalmologists During COVID-19 Pandemic. *Clinical Ophthalmology.* 2021. 15:1267-1275

**Tan, F.,Wang, K.,Liu, J.,Liu, D.,Luo, J.,Zhou, R.**. Viral Transmission and Clinical Features in Asymptomatic Carriers of SARS-CoV-2 in Wuhan, China. *Frontiers in Medicine.* 2020. 7 (no pagination):#pages#

**Tan, Y. P.,Tan, B. Y.,Pan, J.,Wu, J.,Zeng, S. Z.,Wei, H. Y.**. Epidemiologic and clinical characteristics of 10 children with coronavirus disease 2019 in Changsha, China. *Journal of Clinical Virology.* 2020. 127:104353

**Tanaka, H.,Hirayama, A.,Nagai, H.,Shirai, C.,Takahashi, Y.,Shinomiya, H.,Taniguchi, C.,Ogata, T.**. Increased Transmissibility of the SARS-CoV-2 Alpha Variant in a Japanese Population. *International Journal of Environmental Research & Public Health [Electronic Resource].* 2021. 18:22

**Tarabichi, Y.,Watts, B.,Collins, T.,Margolius, D.,Avery, A.,Gunzler, D.,Perzynski, A.**. SARS-CoV-2 Infection among Serially Tested Emergency Medical Services Workers. *Prehospital Emergency Care.* 2021. 25:39-45

**Tasakis, R. N.,Samaras, G.,Jamison, A.,Lee, M.,Paulus, A.,Whitehouse, G.,Verkoczy, L.,Papavasiliou, F. N.,Diaz, M.**. SARS-CoV-2 variant evolution in the United States: High accumulation of viral mutations over time likely through serial Founder Events and mutational bursts. *PLoS ONE [Electronic Resource].* 2021. 16:e0255169

**Tavares, A. S. R.,Bellem, F.,Abreu, R.,Leitao, C.,Medeiros, N.,Alves, P.,Calmeiro, L.**. Assessment and impact of the risk of exposure of portuguese biomedical scientists in the context of covid-19<sup>+</sup>. *International Journal of Environmental Research and Public Health.* 2021. 18(13) (no pagination):#pages#

**Teheran, A. A.,Camero Ramos, G.,Prado de la Guardia, R.,Hernandez, C.,Herrera, G.,Pombo, L. M.,Avila, A. A.,Florez, C.,Barros, E. C.,Perez-Garcia, L.,Paniz-Mondolfi, A.,Ramirez, J. D.**. Epidemiological characterisation of asymptomatic carriers of COVID-19 in Colombia: a cross-sectional study. *BMJ Open.* 2020. 10:e042122

**Tenforde, M. W.,Billig Rose, E.,Lindsell, C. J.,Shapiro, N. I.,Files, D. C.,Gibbs, K. W.,Prekker, M. E.,Steingrub, J. S.,Smithline, H. A.,Gong, M. N.,Aboodi, M. S.,Exline, M. C.,Henning, D. J.,Wilson, J. G.,Khan, A.,Qadir, N.,Stubblefield, W. B.,Patel, M. M.,Self, W. H.,Feldstein, L. R.,Cdc Covid- Response Team**. Characteristics of Adult Outpatients and Inpatients with COVID-19 - 11 Academic Medical Centers, United States, March-May 2020. *MMWR - Morbidity & Mortality Weekly Report.* 2020. 69:841-846

**Thippareddi, H.,Balamurugan, S.,Patel, J.,Singh, M.,Brassard, J.**. Coronaviruses - Potential human threat from foodborne transmission?. *Food Science & Technology-Lebensmittel-Wissenschaft & Technologie.* 2020. #volume#:110147

**Timmons Sund, L.,Bhatt, N. K.,Ference, E. H.,Kim, W.,Johns, M. M., 3rd**. Respiratory Particle Emission During Voice Assessment and Therapy Tasks in a Single Subject. *Journal of Voice.* 2020. 22:22

**Tiwari, A.,So, M. K. P.,Chong, A. C. Y.,Chan, J. N. L.,Chu, A. M. Y.**. Pandemic risk of COVID-19 outbreak in the United States: An analysis of network connectedness with air travel data. *International Journal of Infectious Diseases.* 2021. 103:97-101

**Tokareva, Y.,Englund, J. A.,Dickerson, J. A.,Brown, J. C.,Zerr, D. M.,Walter, E.,Tsogoo, A.,Cappetto, K.,Valdez Gonzalez, J.,Strelitz, B.,Klein, E. J.**. Prevalence of Health Care and Hospital Worker SARS-CoV-2 IgG Antibody in a Pediatric Hospital. *Hospital Pediatrics.* 2021. 11:e48-e53

**Vannoni, M.,McKee, M.,Semenza, J. C.,Bonell, C.,Stuckler, D.**. Using volunteered geographic information to assess mobility in the early phases of the COVID-19 pandemic: A cross-city time series analysis of 41 cities in 22 countries from March 2nd to 26th 2020. *Globalization and Health.* 2020. 16(1) (no pagination):#pages#

**Vermeer, Paul, Kregting, Joris**. Religion and the Transmission of COVID-19 in The Netherlands. *Religions.* 2020. 11:393

**Viegas, M.**. Argentine epidemiological surveillance of SARS-CoV2 in the NGS era. *Biocell.* 2021. 45(SUPPL 1):32-33

**Villani, A.,Coltella, L.,Ranno, S.,Bianchi di Castelbianco, F.,Murru, P. M.,Sonnino, R.,Mazzone, T.,Piccioni, L.,Linardos, G.,Chiavelli, S.,Pontarelli, F.,Corsello, G.,Raponi, M.,Perno, C. F.,Concato, C.**. School in Italy: a safe place for children and adolescents. *Italian Journal of Pediatrics.* 2021. 47(1) (no pagination):#pages#

**Wake, R. M.,Morgan, M.,Choi, J.,Winn, S.**. Reducing nosocomial transmission of COVID-19: implementation of a COVID-19 triage system. *Clinical Medicine.* 2020. 20:e141-e145

**Waltenburg, M. A., Victoroff, T., Rose, C. E., Butterfield, M., Jervis, R. H., Fedak, K. M., Gabel, J. A., Feldpausch, A., Dunne, E. M., Austin, C., Ahmed, F. S., Tubach, S., Rhea, C., Krueger, A., Crum, D. A., Vostok, J., Moore, M. J., Turabelidze, G., Stover, D., Donahue, M., Edge, K., Gutierrez, B., Kline, K. E., Martz, N., Rajotte, J. C., Julian, E., Diedhiou, A., Radcliffe, R., Clayton, J. L., Ortbahn, D., Cummins, J., Barbeau, B., Murphy, J., Darby, B., Graff, N. R., Dostal, T. K. H., Pray, I. W., Tillman, C., Dittrich, M. M., Burns-Grant, G., Lee, S., Spieckerman, A., Iqbal, K., Griffing, S. M., Lawson, A., Mainzer, H. M., Bealle, A. E., Edding, E., Arnold, K. E., Rodriguez, T., Merkle, S., Pettrone, K., Schlanger, K., LaBar, K., Hendricks, K., Lasry, A., Krishnasamy, V., Walke, H. T., Rose, D. A., Honein, M. A.**. Update: COVID-19 Among Workers in Meat and Poultry Processing Facilities - United States, April-May 2020. *MMWR Morb Mortal Wkly Rep.* 2020. 69:887-892

**Wang, F.,Tan, Z.,Yu, Z.,Yao, S.,Guo, C.**. Transmission and control pressure analysis of the COVID-19 epidemic situation using multisource spatio-temporal big data. *PLoS ONE [Electronic Resource].* 2021. 16:e0249145

**Wang, Y.,Teunis, P.**. Strongly Heterogeneous Transmission of COVID-19 in Mainland China: Local and Regional Variation. *Frontiers in Medicine.* 2020. 7:329

**Wee, L. E. I.,Thien, S. Y.,Singh, S. R.,Ling, M. L.,Venkatachalam, I.**. Containing COVID-19 in a specialised neurology centre: the risks of presymptomatic transmission. *Neurological Sciences.* 2020. 41(8):2013-2015

**Wee, L. E.,Venkatachalam, I.,Sim, X. Y. J.,Tan, K. B. K.,Wen, R.,Tham, C. K.,Gan, W. H.,Ko, K. K. K.,Ho, W. Q.,Kwek, G. T. C.,Conceicao, E. P.,Sng, C. Y. E.,Ng, X. H. J.,Ong, J. Y.,Chiang, J. L.,Chua, Y. Y.,Ling, M. L.,Tan, T. T.,Wijaya, L.**. Containment of COVID-19 and reduction in healthcare-associated respiratory viral infections through a multi-tiered infection control strategy. *Infection, Disease and Health.* 2021. 26(2):123-131

**Weil, A. A.,Newman, K. L.,Ong, T. D.,Davidson, G. H.,Logue, J.,Brandstetter, E.,Magedson, A.,McDonald, D.,McCulloch, D. J.,Neme, S.,Lewis, J.,Duchin, J. S.,Zhong, W.,Starita, L. M.,Bedford, T.,Roxby, A. C.,Chu, H. Y.**. Cross-Sectional Prevalence of SARS-CoV-2 Among Skilled Nursing Facility Employees and Residents Across Facilities in Seattle. *Journal of General Internal Medicine.* 2020. 35:3302-3307

**Williams, G. H.,Llewelyn, A.,Brandao, R.,Chowdhary, K.,Hardisty, K. M.,Loddo, M.**. SARS-CoV-2 testing and sequencing for international arrivals reveals significant cross border transmission of high risk variants into the United Kingdom. *EClinicalMedicine.* 2021. 38:101021

**Wu, C. H.,Chou, Y. C.,Lin, F. H.,Hsieh, C. J.,Wu, D. C.,Peng, C. K.,Yu, C. P.**. Epidemiological features of domestic and imported cases with COVID-19 between January 2020 and March 2021 in Taiwan. *Medicine.* 2021. 100:e27360

**Wu, H.,Wu, C.,Lu, Q.,Ding, Z.,Xue, M.,Lin, J.**. Spatiotemporal analysis and the characteristics of the case transmission network of 2019 novel coronavirus disease (COVID-19) in Zhejiang Province, China. *PLoS ONE [Electronic Resource].* 2021. 16:e0257587

**Xiao, Z.,Xie, X.,Guo, W.,Luo, Z.,Liao, J.,Wen, F.,Zhou, Q.,Han, L.,Zheng, T.**. Examining the incubation period distributions of COVID-19 on Chinese patients with different travel histories. *Journal of Infection in Developing Countries.* 2020. 14(4):323-327

**Xu, X. K.,Liu, X. F.,Wu, Y.,Ali, S. T.,Du, Z.,Bosetti, P.,Lau, E. H. Y.,Cowling, B. J.,Wang, L.**. Reconstruction of Transmission Pairs for Novel Coronavirus Disease 2019 (COVID-19) in Mainland China: Estimation of Superspreading Events, Serial Interval, and Hazard of Infection. *Clinical Infectious Diseases.* 2020. 71:3163-3167

**Yadav, D. K.,Shah, P. K.,Enyoh, C. E.,Setiawan, B.**. Paper-based record file of patients could be a fomite for SARS-CoV-2 transmission in hospital setting of low and middle income countries (LMICs). *Pakistan Journal of Medical and Health Sciences.* 2020. 14(3):993-995

**Yamagishi, T.,Ohnishi, M.,Matsunaga, N.,Kakimoto, K.,Kamiya, H.,Okamoto, K.,Suzuki, M.,Gu, Y.,Sakaguchi, M.,Tajima, T.,Takaya, S.,Ohmagari, N.,Takeda, M.,Matsuyama, S.,Shirato, K.,Nao, N.,Hasegawa, H.,Kageyama, T.,Takayama, I.,Saito, S.,Wada, K.,Fujita, R.,Saito, H.,Okinaka, K.,Griffith, M.,Parry, A. E.,Barnetson, B.,Leonard, J.,Wakita, T.**. Environmental Sampling for Severe Acute Respiratory Syndrome Coronavirus 2 During a COVID-19 Outbreak on the Diamond Princess Cruise Ship. *Journal of Infectious Diseases.* 2020. 222:1098-1102

**Yamamoto, K.,Takazono, T.,Okamoto, R.,Morimoto, S.,Hosogaya, N.,Tashiro, M.,Miyazaki, T.,Yanagihara, K.,Izumikawa, K.,Mukae, H.**. Evaluation of droplet and aerosol dispersion under high flow nasal cannula with or without surgical mask. *American Journal of Respiratory and Critical Care Medicine. Conference: American Thoracic Society International Conference, ATS.* 2021. 203:#pages#

**Yameen, M.,Sattar, S.,Khalid, A.,Aslam, M. A.,Zafar, N.,Saeed, M. H.,Arif, M. H.,Jahangeer, M.,Qadeer, A.,Hussain, S.,Aamir, M.,Mukhtar, S.,Nasir, H.,Shahzad, A.**. Novel SARS-COV-2 pandemic transmission with ongoing antiviral therapies and Vaccine design. *Postepy Mikrobiologii.* 2021. 60(1):13-20

**Yang, B., Tsang, T. K., Gao, H., Lau, E. H. Y., Lin, Y., Ho, F., Xiao, J., Wong, J. Y., Adam, D. C., Liao, Q., Wu, P., Cowling, B. J., Leung, G. M.**. Universal community nucleic acid testing for COVID-19 in Hong Kong reveals insights into transmission dynamics: a cross-sectional and modelling study. *Clinical Infectious Diseases.* 2021. 28:28

**Yang, C. H.,Jung, H.**. Topological dynamics of the 2015 South Korea MERS-CoV spread-on-contact networks. *Scientific Reports.* 2020. 10:4327

**Yanuar Safri, A.,Estiasari, R.,Maharani, K.,Budikayanti, A.,Pradiptaloka, E.,Stephanie, J.,Imran, D.,Kumalati, J.**. The risk of coronavirus disease (COVID-19) transmission to health workers in the neurology medical service at Dr. Cipto Mangunkusumo National Hospital, Jakarta. *Journal of the Neurological Sciences.* 2021. Conference: World Congress of Neurology:#pages#

**Yatsyshina, S.,Mamoshina, M.,Elkina, M.,Akimkin, V.**. Prevalence of COVID-19 in individuals without respiratory symptoms. *Antimicrobial Resistance and Infection Control. Conference: 6th International Conference on Prevention and Infection Control, ICPIC.* 2021. 10:#pages#

**Ye, M. J.,Sharma, D.,Campiti, V. J.,Rubel, K. E.,Burgin, S. J.,Illing, E. A.,Ting, J. Y.,Park, J. H.,Johnson, J. D.,Vernon, D. J.,Lee, H. B.,Nesemeier, B. R.,Shipchandler, T. Z.**. Aerosol and droplet generation from mandible and midface fixation: Surgical risk in the pandemic era. *American Journal of Otolaryngology.* 2021. 42:102829

**Yeh, T. Y.,Contreras, G. P.**. Viral transmission and evolution dynamics of sars-cov-2 in shipboard quarantine. *Bulletin of the World Health Organization.* 2021. 99(7):486-495

**Yoon, Y.,Choi, G. J.,Kim, J. Y.,Kim, K. R.,Park, H.,Chun, J. K.,Kim, Y. J.**. Childcare Exposure to Severe Acute Respiratory Syndrome Coronavirus 2 for 4-Year-Old Presymptomatic Child, South Korea. *Emerging Infectious Diseases.* 2021. 27:341-347

**Zamberg, I.,Mavrakanas, T.,Ernandez, T.,Bourquin, V.,Zellweger, M.,Marangon, N.,Raimbault, F.,Winzeler, R.,Iten, A.,Martin, P. Y.,Saudan, P.**. Management of patients on maintenance dialysis during the SARS CoV-2 pandemic: A perspective from Geneva, Switzerland. *Swiss Medical Weekly.* 2020. 150(SUPPL 248):12S

**Zeller, M.,Gangavarapu, K.,Anderson, C.,Smither, A. R.,Vanchiere, J. A.,Rose, R.,Snyder, D. J.,Dudas, G.,Watts, A.,Matteson, N. L.,Robles-Sikisaka, R.,Marshall, M.,Feehan, A. K.,Sabino-Santos, G., Jr.,Bell-Kareem, A. R.,Hughes, L. D.,Alkuzweny, M.,Snarski, P.,Garcia-Diaz, J.,Scott, R. S.,Melnik, L. I.,Klitting, R.,McGraw, M.,Belda-Ferre, P.,DeHoff, P.,Sathe, S.,Marotz, C.,Grubaugh, N. D.,Nolan, D. J.,Drouin, A. C.,Genemaras, K. J.,Chao, K.,Topol, S.,Spencer, E.,Nicholson, L.,Aigner, S.,Yeo, G. W.,Farnaes, L.,Hobbs, C. A.,Laurent, L. C.,Knight, R.,Hodcroft, E. B.,Khan, K.,Fusco, D. N.,Cooper, V. S.,Lemey, P.,Gardner, L.,Lamers, S. L.,Kamil, J. P.,Garry, R. F.,Suchard, M. A.,Andersen, K. G.**. Emergence of an early SARS-CoV-2 epidemic in the United States. *Cell.* 2021. 184:4939-4952.e15

**Zhang, K.,Tong, W.,Wang, X.,Lau, J. Y. N.**. Estimated prevalence and viral transmissibility in subjects with asymptomatic SARS-CoV-2 infections in Wuhan, China. *Precision Clinical Medicine.* 2020. 3(4):301-305

**Zhang, L.,Zhu, F.,Xie, L.,Wang, C.,Wang, J.,Chen, R.,Jia, P.,Guan, H. Q.,Peng, L.,Peng, P.,Zhang, P.,Chu, Q.,Shen, Q.,Wang, Y.,Xu, S. Y.,Zhao, J. P.,Zhou, M.,Chen, Y.**. The experience of treating patients with cancer during the COVID-19 pandemic in China. *Cancer Research. Conference: American Association for Cancer Research Annual Meeting, AACR.* 2020. 80:#pages#

**Zhang, R.,Li, Y.,Zhang, A. L.,Wang, Y.,Molina, M. J.**. Identifying airborne transmission as the dominant route for the spread of COVID-19. *Proceedings of the National Academy of Sciences of the United States of America.* 2020. 117(26):14857-14863

**Zhang, W.,Govindavari, J. P.,Davis, B. D.,Chen, S. S.,Kim, J. T.,Song, J.,Lopategui, J.,Plummer, J. T.,Vail, E.**. Analysis of Genomic Characteristics and Transmission Routes of Patients With Confirmed SARS-CoV-2 in Southern California During the Early Stage of the US COVID-19 Pandemic. *JAMA Network Open.* 2020. 3:e2024191

**Zhao, J.,Li, X.,Huang, W.,Zheng, J.**. Potential risk factors for case fatality rate of novel coronavirus (COVID-19) in China: A pooled analysis of individual patient data. *American Journal of Emergency Medicine.* 2020. 38(11):2374-2380

**Zhu, S.,Tao, J.,Gao, H.,He, D.**. Age, source, and future risk of COVID-19 infections in two settings of Hong Kong and Singapore. *BMC Research Notes.* 2020. 13:336

**Zulli, A.,Bakker, A.,Racharaks, R.,Nieto-Caballero, M.,Hernandez, M.,Shaughnessy, R.,Haverinen-Shaughnessy, U.,Ijaz, M. K.,Rubino, J.,Peccia, J.**. Occurrence of respiratory viruses on school desks. *American Journal of Infection Control.* 2021. 49:464-468

**Abtahi-Naeini, B.,Saffaei, A.**. COVID-19 and elective cosmetic procedures: Asymptomatic transmission in epidemic area and subsequent burdens. *Journal of Cosmetic Dermatology.* 2020. 19(8):1838-1839

**Ahmed, M. A. M.,Colebunders, R.,Siewe Fodjo, J. N.**. Evidence for significant COVID-19 community transmission in Somalia using a clinical case definition. *International Journal of Infectious Diseases.* 2020. 98:206-207

**Ali Hassan, S. M.,Palacios, C. M.,Ethier, T.,Bisleri, G.**. Improved Safety of Endoscopic Vessel Harvesting During the COVID-19 Pandemic. *Annals of Thoracic Surgery.* 2020. 110:e449-e450

**Alkalamouni, H.,Hitti, E.,Zaraket, H.**. Adopting fresh air ventilation may reduce the risk of airborne transmission of SARS-CoV-2 in COVID-19 unit. *Journal of Infection.* 2021. 01:01

**Allen, J. G.,Ibrahim, A. M.**. Indoor Air Changes and Potential Implications for SARS-CoV-2 Transmission. *JAMA - Journal of the American Medical Association.* 2021. 325(20):2112-2113

**Allen, J.,Scully, T.**. The cumulative risk of acquiring COVID-19 in outpatient pediatric practice. *Pediatric Pulmonology.* 2021. 56(1):19-20

**Altmann, D. M.**. Children and the return to school: How much should we worry about covid-19 and long covid?. *The BMJ.* 2021. 372 (no pagination):#pages#

**Anastassopoulou, C.,Spanakis, N.,Tsakris, A.**. SARS-CoV-2 transmission, the ambiguous role of children and considerations for the reopening of schools in the fall. *Future Microbiology.* 2020. 15(13):1201-1206

**Anonymous,**. Coronavirus: Covid-19 risks for children. *Science.* 2020. 370(6514):307B

**Anonymous,**. Updated rapid risk assessment from ECDC on the novel coronavirus disease 2019 (COVID-19) pandemic: Increased transmission in the EU/EEA and the UK. *Eurosurveillance.* 2020. 25:#pages#

**Ash, C.**. Time and intimacy drive transmission. *Science.* 2021. 371(6526):250B

**Atayik, E.**. Low percentages of eosinophils after the 4th dose of mepolizumab may be an independent risk factor for SARS-CoV-2 transmission in patients with severe asthma. *Allergy: European Journal of Allergy and Clinical Immunology.* 2021. 76(SUPPL 110):477

**Abulhassan, Y.,Davis, G. A.**. Considerations for the transportation of school aged children amid the Coronavirus pandemic. *Transportation Research Interdisciplinary Perspectives.* 2021. 9:100290

**Ahmad, Z.,Rahim, S.,Ud Din, N.,Ahmed, A.**. Practice of Academic Surgical Pathology During the COVID-19 Pandemic. *American Journal of Clinical Pathology.* 2020. 154:724-730

**Ahmed, N.,Maqsood, A.,Abduljabbar, T.,Vohra, F.**. Tobacco Smoking a Potential Risk Factor in Transmission of COVID-19 Infection. *Pakistan Journal of Medical Sciences.* 2020. 36:S104-S107

**Ak, G.,Gunay, A. Y.,Olley, R. C.,Sen, N.**. Managing emerging challenges of Coronavirus disease 2019 (COVID-19) in dentistry. *European Oral Research.* 2020. 54:101-107

**Al-Mayyahi, R. S.,Al-Tumah, W. A. G.**. Genetic Structure, Transmission, Clinical Characteristics, Diagnosis, Treatment and Prevention of Coronavirus Disease 2019 (COVID-19): A Review. *Iraqi Journal of Pharmaceutical Sciences.* 2021. 30(1):56-65

**Al-Tawfiq, J. A.,Auwaerter, P. G.**. Healthcare-associated infections: the hallmark of Middle East respiratory syndrome coronavirus with review of the literature. *Journal of Hospital Infection.* 2019. 101(1):20-29

**Aldahlawi, S. A.,Afifi, I. K.**. COVID-19 in dental practice: Transmission risk, infection control challenge, and clinical implications. *Open Dentistry Journal.* 2020. 14(1):348-354

**Althouse, B. M.,Wenger, E. A.,Miller, J. C.,Scarpino, S. V.,Allard, A.,Hebert-Dufresne, L.,Hu, H.**. Superspreading events in the transmission dynamics of SARS-CoV-2: Opportunities for interventions and control. *Plos Biology.* 2020. 18:e3000897

**Amdaoud, M.,Arcuri, G.,Levratto, N.**. Are regions equal in adversity? A spatial analysis of spread and dynamics of COVID-19 in Europe. *European Journal of Health Economics.* 2021. 22:629-642

**Amiri, A.,Qi, F.,Alonso, M. B. C. C.,Nunez, N. S.,Kozyk, O.**. Evaluation of the droplets and aerosols, posing potential risks of covid-19 disease infection transmission in dentistry: A systematic review and meta-analysis of observational studies. *Pesquisa Brasileira em Odontopediatria e Clinica Integrada.* 2021. 21 (no pagination):#pages#

**Anand, P.**. Covid-19: broadening research expertise to understand workplace transmission. *The BMJ.* 2020. 370 (no pagination):#pages#

**Ari, A.,Fink, J. B.**. Aerosol drug delivery to tracheotomized patients with COVID-19: Pragmatic suggestions for clinicians. *Canadian Journal of Respiratory Therapy.* 2021. 57:49-52

**Auerbach, A. M.,Thachil, T.**. How does Covid-19 affect urban slums? Evidence from settlement leaders in India. *World Development.* 2021. 140:105304

**Azuma, K.,Yanagi, U.,Kagi, N.,Kim, H.,Ogata, M.,Hayashi, M.**. Environmental factors involved in SARS-CoV-2 transmission: effect and role of indoor environmental quality in the strategy for COVID-19 infection control. *Environmental Health and Preventive Medicine.* 2020. 25(1) (no pagination):#pages#

**Aherfi, S.,Gautret, P.,Chaudet, H.,Raoult, D.,La Scola, B.**. Clusters of COVID-19 associated with Purim celebration in the Jewish community in Marseille, France, March 2020. *International Journal of Infectious Diseases.* 2020. 100:88-94

**Aizawa, Y.,Shobugawa, Y.,Tomiyama, N.,Nakayama, H.,Takahashi, M.,Yanagiya, J.,Kaji, N.,Ikuse, T.,Izumita, R.,Yamanaka, T.,Hasegawa, S.,Tamura, T.,Saito, R.,Saitoh, A.**. Coronavirus Disease 2019 Cluster Originating in a Primary School Teachers' Room in Japan. *Pediatric Infectious Disease Journal.* 2021. 23:23

**Al Lawati, A.,Khamis, F.,Al Habsi, S.,Al Dalhami, K.**. Risk of COVID-19 Infection in Healthcare Workers Exposed During Use of Non-invasive Ventilation in a Tertiary Care Hospital in Oman. *Oman Medical Journal.* 2021. 36:e236

**AlHarmi, R. A. R.,Fateel, T.,Sayed Adnan, J.,AlAwadhi, K.**. Acute pancreatitis in a patient with COVID-19. *BMJ Case Reports.* 2021. 14:11

**Asad, H.,Johnston, C.,Blyth, I.,Holborow, A.,Bone, A.,Porter, L.,Tidswell, P.,Healy, B.**. Health Care Workers and Patients as Trojan Horses: a COVID19 ward outbreak. *#journal#.* 2020. #volume#:#pages#

**Bassi, M.,Anile, M.,Pecoraro, Y.,Ruberto, F.,Martelli, S.,Piazzolla, M.,Pugliese, F.,Venuta, F.,De Giacomo, T.**. Bedside Transcervical-Transtracheal Postintubation Injury Repair in a COVID-19 Patient. *Annals of Thoracic Surgery.* 2020. 110:e417-e419

**Bender, J. K.,Brandl, M.,Hohle, M.,Buchholz, U.,Zeitlmann, N.**. Analysis of Asymptomatic and Presymptomatic Transmission in SARS-CoV-2 Outbreak, Germany, 2020. *Emerging Infectious Diseases.* 2021. 27:04

**Bernadou, A.,Bouges, S.,Catroux, M.,Rigaux, J. C.,Laland, C.,Leveque, N.,Noury, U.,Larrieu, S.,Acef, S.,Habold, D.,Cazenave-Roblot, F.,Filleul, L.**. High impact of COVID-19 outbreak in a nursing home in the Nouvelle-Aquitaine region, France, March to April 2020. *BMC Infectious Diseases.* 2021. 21(1) (no pagination):#pages#

**Bhutaka, N.,Shah, M.**. Outcome and safety of percutaneous tracheostomy in COVID-19 patients: A single-center experience. *Indian Journal of Critical Care Medicine.* 2021. 25(SUPPL 1):S9-S10

**Boender, T. S.,Bender, J. K.,Kruger, A.,Michaelis, K.,Buchholz, U.**. Factors preventing SARS-CoV-2 transmission during unintentional exposure in a GP practice: A cohort study of patient contacts; Germany, 2020. *Epidemiology and Infection..* 2021. #volume#:#pages#

**Bohmer, M. M.,Buchholz, U.,Corman, V. M.,Hoch, M.,Katz, K.,Marosevic, D. V.,Bohm, S.,Woudenberg, T.,Ackermann, N.,Konrad, R.,Eberle, U.,Treis, B.,Dangel, A.,Bengs, K.,Fingerle, V.,Berger, A.,Hormansdorfer, S.,Ippisch, S.,Wicklein, B.,Grahl, A.,Portner, K.,Muller, N.,Zeitlmann, N.,Boender, T. S.,Cai, W.,Reich, A.,An der Heiden, M.,Rexroth, U.,Hamouda, O.,Schneider, J.,Veith, T.,Muhlemann, B.,Wolfel, R.,Antwerpen, M.,Walter, M.,Protzer, U.,Liebl, B.,Haas, W.,Sing, A.,Drosten, C.,Zapf, A.**. Investigation of a COVID-19 outbreak in Germany resulting from a single travel-associated primary case: a case series. *The Lancet Infectious Diseases.* 2020. 20:920-928

**Boogaard, L. H.,Sikkema, R. S.,van Beek, Jhgm,Brockhoff, H. J.,Dalebout, E.,de Heus, B.,Niemansburg, S. L.,Nieuwenhuijse, D. F.,Stougje, D.,Verspui, E.,Oude Munnink, B. B.,Koopmans, M. P. G.,Fanoy, E. B.**. A mixed-methods approach to elucidate SARS-CoV-2 transmission routes and clustering in outbreaks in native workers and labour migrants in the fruit and vegetable packaging industry in South Holland, the Netherlands, May to July 2020. *International Journal of Infectious Diseases.* 2021. 109:24-32

**Brito, C. A. A.,Brito, M. C. M.,Martins, T. H. F.,Brito, C. C. M.,Albuquerque, M. F. M.,Brito, Rccm**. Clinical laboratory and dispersion pattern of COVID-19 in a family cluster in the social-distancing period. *Journal of Infection in Developing Countries.* 2020. 14:987-993

**Burak, K. W.,Law, S.,Rice, C.,Hu, J.,Fung, C. I.,Woo, A. K. H.,Fonseca, K.,Lang, A. L. S.,Kanji, J. N.,Meatherall, B. L.**. COVID-19 outbreak among physicians at a Canadian curling bonspiel: a descriptive observational study. *CMAJ open.* 2021. 9:E87-E95

**Capon, A.,Houston, J.,Rockett, R.,Sheppeard, V.,Chaverot, S.,Arnott, A.,Parashko, T.,Ferson, M.**. Risk factors leading to COVID-19 cases in a Sydney restaurant. *Australian and New Zealand journal of public health..* 2021. 28:#pages#

**Chang, Y. S., Mayer, S., Davis, E. S., Figueroa, E., Leo, P., Finn, P. W., Perkins, D. L.**. Transmission Dynamics of Large Coronavirus Disease Outbreak in Homeless Shelter, Chicago, Illinois, USA, 2020. *Emerging Infectious Diseases.* 2021. 28:02

**Charlotte, N.**. High Rate of SARS-CoV-2 Transmission Due to Choir Practice in France at the Beginning of the COVID-19 Pandemic. *Journal of Voice.* 2020. 23:23

**Chaw, L.,Koh, W. C.,Jamaludin, S. A.,Naing, L.,Alikhan, M. F.,Wong, J.**. Analysis of SARS-CoV-2 Transmission in Different Settings, Brunei. *Emerging Infectious Diseases.* 2020. 26:2598-2606

**Chen, J.,He, H.,Cheng, W.,Liu, Y.,Sun, Z.,Chai, C.,Kong, Q.,Sun, W.,Zhang, J.,Guo, S.,Shi, X.,Wang, J.,Chen, E.,Chen, Z.**. Potential transmission of SARS-CoV-2 on a flight from Singapore to Hangzhou, China: An epidemiological investigation. *Travel Medicine & Infectious Disease.* 2020. 36:101816

**Chen, S.,Yin, Q.,Shi, H.,Du, D.,Chang, S.,Ni, L.,Qiu, H.,Chen, Z.,Zhang, J.,Zhang, W.**. A familial cluster, including a kidney transplant recipient, of Coronavirus Disease 2019 (COVID-19) in Wuhan, China. *American Journal of Transplantation.* 2020. 20:1869-1874

**Cheng, V. C. C.,Fung, K. S. C.,Siu, G. K. H.,Wong, S. C.,Cheng, L. S. K.,Wong, M. S.,Lee, L. K.,Chan, W. M.,Chau, K. Y.,Leung, J. S. L.,Chu, A. W. H.,Chan, W. S.,Lu, K. K.,Tam, K. K. G.,Ip, J. D.,Leung, K. S. S.,Lung, D. C.,Tse, H.,To, K. K. W.,Yuen, K. Y.**. Nosocomial outbreak of COVID-19 by possible airborne transmission leading to a superspreading event. *Clinical infectious diseases : an official publication of the Infectious Diseases Society of America..* 2021. 14:#pages#

**Cheng, V. C.,Fung, K. S.,Siu, G. K.,Wong, S. C.,Cheng, L. S.,Wong, M. S.,Lee, L. K.,Chan, W. M.,Chau, K. Y.,Leung, J. S.,Chu, A. W.,Chan, W. S.,Lu, K. K.,Tam, K. K.,Ip, J. D.,Leung, K. S.,Lung, D. C.,Tse, H.,To, K. K.,Yuen, K. Y.**. Nosocomial Outbreak of Coronavirus Disease 2019 by Possible Airborne Transmission Leading to a Superspreading Event. *Clinical Infectious Diseases.* 2021. 73:e1356-e1364

**Cheng, W.,Zhao, B.,Chen, E.,Li, G.,Ma, J.,Cui, Y.,Xu, C.,Cui, Y.,Shen, B.,Luo, M.,Yin, D.,Yao, L.**. Index and First-Generation Cases in a COVID-19 Outbreak - Jilin Province, China, 2021. *China CDC Weekly.* 2021. 3:405-408

**Cho, H. J.,Koo, J. W.,Roh, S. K.,Kim, Y. K.,Suh, J. S.,Moon, J. H.,Sohn, S. K.,Baek, D. W.**. COVID-19 transmission and blood transfusion: A case report. *Journal of Infection and Public Health.* 2020. 13(11):1678-1679

**Covid-Epidemiology Investigation, Team,Laboratory Testing, Team,Zhang, W.**. Local Outbreak of COVID-19 in Shunyi District Attributed to an Asymptomatic Carrier with a History of Stay in Indonesia - Beijing Municipality, China, December 23, 2020. *China CDC Weekly.* 2021. 3:214-217

**Cruzado, M. A. A.,Ferrer, F.**. Outbreak in the dialysis unit: Contact tracing in a hospital based dialysis centre in the Philippines during the covid-19 pandemic. *Nephrology.* 2020. 25(SUPPL 1):3

**de Siqueira, I. C.,Camelier, A. A.,Maciel, E. A. P.,Nonaka, C. K. V.,Neves, Mclc,Macedo, Y. S. F.,de Sousa, K. A. F.,Araujo, V. C.,Paste, A. A.,Souza, B. S. F.,Graf, T.**. Early detection of P.1 variant of SARS-CoV-2 in a cluster of cases in Salvador, Brazil. *International Journal of Infectious Diseases.* 2021. 108:252-255

**Dhanasekaran, V.,Edwards, K. M.,Xie, R.,Gu, H.,Adam, D. C.,Chang, L. D. J.,Cheuk, S. S. Y.,Gurung, S.,Krishnan, P.,Ng, D. Y. M.,Liu, G. Y. Z.,Wan, C. K. C.,Cheng, S. S. M.,Tsang, D. N. C.,Cowling, B. J.,Peiris, M.,Poon, L. L. M.**. Air travel-related outbreak of multiple SARS-CoV-2 variants. *Journal of Travel Medicine.* 2021. 20:20

**Egger, F., Faude, O., Schreiber, S., Gartner, B. C., Meyer, T.**. Does playing football (soccer) lead to SARS-CoV-2 transmission? - A case study of 3 matches with 18 infected football players. *Science & Medicine in Football.* 2021. 5:2-7

**England, R.,Peirce, N.,Wedatilake, T.,Torresi, J.,Kemp, S.,Cook, M.,Mitchell, S.,Harland, A.**. The Potential for Airborne Transmission of SARS-CoV-2 in Sport: A Cricket Case Study. *International Journal of Sports Medicine.* 2021. 42:407-418

**Farinholt, T.,Doddapaneni, H.,Qin, X.,Menon, V.,Meng, Q.,Metcalf, G.,Chao, H.,Gingras, M. C.,Farinholt, P.,Agrawal, C.,Muzny, D. M.,Piedra, P. A.,Gibbs, R. A.,Petrosino, J.**. Transmission event of SARS-CoV-2 Delta variant reveals multiple vaccine breakthrough infections. *MedRxiv : the Preprint Server for Health Sciences.* 2021. 12:12

**Fisher, K. A.,Tenforde, M. W.,Feldstein, L. R.,Lindsell, C. J.,Shapiro, N. I.,Files, D. C.,Gibbs, K. W.,Erickson, H. L.,Prekker, M. E.,Steingrub, J. S.,Exline, M. C.,Henning, D. J.,Wilson, J. G.,Brown, S. M.,Peltan, I. D.,Rice, T. W.,Hager, D. N.,Ginde, A. A.,Talbot, H. K.,Casey, J. D.,Grijalva, C. G.,Flannery, B.,Patel, M. M.,Self, W. H.,I. V. Y. Network Investigators,Cdc Covid- Response Team**. Community and Close Contact Exposures Associated with COVID-19 Among Symptomatic Adults >=18 Years in 11 Outpatient Health Care Facilities - United States, July 2020. *MMWR - Morbidity & Mortality Weekly Report.* 2020. 69:1258-1264

**Gao, X.,Yuan, Z.,Yang, D.,Li, H.,Zhang, Y.,Gao, P.,Liu, X.,Zhao, W.,Xiao, T.,Duan, X.**. A family cluster of severe acute respiratory syndrome coronavirus 2 infections. *European Journal of Clinical Microbiology & Infectious Diseases.* 2020. 39:1611-1615

**Ghinai, I.,Woods, S.,Ritger, K. A.,McPherson, T. D.,Black, S. R.,Sparrow, L.,Fricchione, M. J.,Kerins, J. L.,Pacilli, M.,Ruestow, P. S.,Arwady, M. A.,Beavers, S. F.,Payne, D. C.,Kirking, H. L.,Layden, J. E.**. Community Transmission of SARS-CoV-2 at Two Family Gatherings - Chicago, Illinois, February-March 2020. *MMWR - Morbidity & Mortality Weekly Report.* 2020. 69:446-450

**Giachino, M.,Valera, C. B. G.,Rodriguez Velasquez, S.,Dohrendorf-Wyss, M. A.,Rozanova, L.,Flahault, A.**. Understanding the Dynamics of the COVID-19 Pandemic: A Real-Time Analysis of Switzerland's First Wave. *International Journal of Environmental Research & Public Health [Electronic Resource].* 2020. 17:27

**Giuliani, R.,Cairone, C.,Tavoschi, L.,Ciaffi, L.,Sebastiani, T.,Bartolotti, R.,Mancini, S.,Cremonini, L.,Ranieri, R.**. COVID-19 outbreak investigation and response in a penitentiary setting: the experience of a prison in Italy, February to April 2020. *Euro Surveillance: Bulletin Europeen sur les Maladies Transmissibles = European Communicable Disease Bulletin.* 2021. 26:09

**Gutierrez-Hernandez, O.,Garcia, L. V.**. Increased risk of COVID-19 from walking dogs? Most likely, a spurious finding. *Environmental Research.* 2021. 201 (no pagination):#pages#

**Hamner, L.,Dubbel, P.,Capron, I.,Ross, A.,Jordan, A.,Lee, J.,Lynn, J.,Ball, A.,Narwal, S.,Russell, S.,Patrick, D.,Leibrand, H.**. High SARS-CoV-2 Attack Rate Following Exposure at a Choir Practice - Skagit County, Washington, March 2020. *MMWR - Morbidity & Mortality Weekly Report.* 2020. 69:606-610

**Han, T.**. Outbreak investigation: transmission of COVID-19 started from a spa facility in a local community in Korea. *Epidemiology and health.* 2020. 42:e2020056

**Harada, S.,Uno, S.,Ando, T.,Iida, M.,Takano, Y.,Ishibashi, Y.,Uwamino, Y.,Nishimura, T.,Takeda, A.,Uchida, S.,Hirata, A.,Sata, M.,Matsumoto, M.,Takeuchi, A.,Obara, H.,Yokoyama, H.,Fukunaga, K.,Amagai, M.,Kitagawa, Y.,Takebayashi, T.,Hasegawa, N.**. Control of a Nosocomial Outbreak of COVID-19 in a University Hospital. *Open Forum Infectious Diseases.* 2020. 7:ofaa512

**Hendrix, M. J.,Walde, C.,Findley, K.,Trotman, R.**. Absence of Apparent Transmission of SARS-CoV-2 from Two Stylists After Exposure at a Hair Salon with a Universal Face Covering Policy - Springfield, Missouri, May 2020. *MMWR - Morbidity & Mortality Weekly Report.* 2020. 69:930-932

**Hernandez, A.,Munoz, P.,Rojas, J. C.,Eskildsen, G. A.,Sandoval, J.,Rao, K. S.,Gittens, R. A.,Loaiza, J. R.**. Epidemiological Chronicle of the First Recovered Coronavirus Disease Patient From Panama: Evidence of Early Cluster Transmission in a High School of Panama City. *Frontiers in Public Health.* 2020. 8:553730

**Herzog, M.,Beule, A. G.,Luers, J. C.,Guntinas-Lichius, O.,Sowerby, L. J.,Grafmans, D.**. Results of a national web-based survey on the SARS-CoV-2 infectious state of otorhinolaryngologists in Germany. *European Archives of Oto-Rhino-Laryngology.* 2021. 278:1247-1255

**Hijnen, D.,GeurtsvanKessel, C.,Marzano, A. V.,Eyerich, K.,Gim nez-Arnau, A. M.,Joly, P.,Vestergaard, C.,Sticherling, M.,Schmidt, E.,Lee, V. J.**. SARS-CoV-2 transmission from presymptomatic meeting attendee, Germany. *Emerging Infectious Diseases.* 2020. 26(8):1935-1937

**Hodcroft, E. B.**. Preliminary case report on the SARS-CoV-2 cluster in the UK, France, and Spain. *Swiss Medical Weekly.* 2020. 150:24

**Huang, R.,Xia, J.,Chen, Y.,Shan, C.,Wu, C.**. A family cluster of SARS-CoV-2 infection involving 11 patients in Nanjing, China. *The Lancet Infectious Diseases.* 2020. 20:534-535

**Jashari, R.,Van Esbroeck, M.,Vanhaebost, J.,Micalessi, I.,Kerschen, A.,Mastrobuoni, S.**. The risk of transmission of the novel coronavirus (SARS-CoV-2) with human heart valve transplantation: evaluation of cardio-vascular tissues from two consecutive heart donors with asymptomatic COVID-19. *Cell & Tissue Banking.* 2021. 09:09

**Jiang, G.,Wang, C.,Song, L.,Wang, X.,Zhou, Y.,Fei, C.,Liu, H.**. Aerosol transmission, an indispensable route of COVID-19 spread: case study of a department-store cluster. *Frontiers of Environmental Science & Engineering.* 2021. 15:46

**Jiang, X. L.,Zhang, X. L.,Zhao, X. N.,Li, C. B.,Lei, J.,Kou, Z. Q.,Sun, W. K.,Hang, Y.,Gao, F.,Ji, S. X.,Lin, C. F.,Pang, B.,Yao, M. X.,Anderson, B. D.,Wang, G. L.,Yao, L.,Duan, L. J.,Kang, D. M.,Ma, M. J.**. Transmission Potential of Asymptomatic and Paucisymptomatic Severe Acute Respiratory Syndrome Coronavirus 2 Infections: A 3-Family Cluster Study in China. *Journal of Infectious Diseases.* 2020. 221:1948-1952

**Karki, B. B., Mohammad, S., Chung, W., Eltweri, A., Sauodi, T., Dennison, A., Garcea, G.**. Multiple outbreak of SARS-COVID-19 in surgical wards at The Tertiary Hospital. *British Journal of Surgery.* 2021. 108(SUPPL 6):vi160

**Kasper, M. R.,Geibe, J. R.,Sears, C. L.,Riegodedios, A. J.,Luse, T.,von Thun, A. M.,McGinnis, M. B.,Olson, N.,Houskamp, D.,Fenequito, R.,Burgess, T. H.,Armstrong, A. W.,DeLong, G.,Hawkins, R. J.,Gillingham, B. L.**. An outbreak of covid-19 on an aircraft carrier. *New England Journal of Medicine.* 2020. 383(25):2417-2426

**Khanh, N. C.,Thai, P. Q.,Quach, H. L.,Thi, N. H.,Dinh, P. C.,Duong, T. N.,Mai, L. T. Q.,Nghia, N. D.,Tu, T. A.,Quang, N.,Quang, T. D.,Nguyen, T. T.,Vogt, F.,Anh, D. D.**. Transmission of SARS-CoV 2 During Long-Haul Flight. *Emerging Infectious Diseases.* 2020. 26:2617-2624

**Kobayashi, T., Yoshii, K., Linton, N. M., Suzuki, M., Nishiura, H.**. Age dependence of the natural history of infection with severe acute respiratory syndrome coronavirus 2 (SARS-CoV-2): An analysis of Diamond Princess data. *International Journal of Infectious Diseases.* 2021. 10:10

**Kong, D.,Wang, Y.,Lu, L.,Wu, H.,Ye, C.,Wagner, A. L.,Yang, J.,Zheng, Y.,Gong, X.,Zhu, Y.,Jin, B.,Xiao, W.,Mao, S.,Jiang, C.,Lin, S.,Han, R.,Yu, X.,Cui, P.,Fang, Q.,Lu, Y.,Pan, H.**. Clusters of 2019 coronavirus disease (COVID-19) cases in Chinese tour groups. *Transboundary & Emerging Diseases.* 2021. 68:684-691

**Koureas, M.,Speletas, M.,Bogogiannidou, Z.,Babalis, D.,Pinakas, V.,Pinaka, O.,Komnos, A.,Tsoutsa, S.,Papadamou, G.,Kyritsi, M. A.,Vontas, A.,Nakoulas, V.,Sapoynas, S.,Kanellopoulos, N.,Kalompatsios, D.,Papadouli, V.,Dadouli, K.,Soteriades, S.,Mina, P.,Mouchtouri, V. A.,Anagnostopoulos, L.,Stamoulis, K. E.,Agorastos, K.,Petinaki, E. A.,Prezerakos, P.,Tsiodras, S.,Hadjichristodoulou, C.**. Transmission Dynamics of SARS-CoV-2 during an Outbreak in a Roma Community in Thessaly, Greece-Control Measures and Lessons Learned. *International Journal of Environmental Research & Public Health [Electronic Resource].* 2021. 18:11

**Krass, P.,Zimbrick-Rogers, C.,Iheagwara, C.,Ford, C. A.,Calderoni, M.**. COVID-19 Outbreak Among Adolescents at an Inpatient Behavioral Health Hospital. *Journal of Adolescent Health.* 2020. 67(4):612-614

**Lam, H. Y.,Lam, T. S.,Wong, C. H.,Lam, W. H.,Mei, E. L. C.,Kuen, Y. L. C.,Wai, W. L. T.,Hin, B. H. C.,Wong, K. H.,Chuang, S. K.**. A superspreading event involving a cluster of 14 coronavirus disease 2019 (COVID-19) infections from a family gathering in Hong Kong Special Administrative Region SAR (China). *Western Pacific Surveillance Response Journal.* 2020. 11:36-40

**Lamichhane, S.,Gupta, S.,Akinjobi, G.,Ndubuka, N.**. Familial cluster of asymptomatic COVID-19 cases in a First Nation community in Northern Saskatchewan, Canada. *Canada Communicable Disease Report.* 2021. 47:94-96

**Leeman, D., Ma, T., Pathiraja, M., Taylor, J., Adnan, T., Baltas, I., Ioannou, A., Iyengar, S., Mearkle, R., Stockdale, T., Van Den Abbeele, K., Balasegaram, S.**. COVID-19 Nosocomial Transmission Dynamics, a Retrospective Cohort Study of Two Healthcare Associated Clusters in a District Hospital in England during March and April 2020. *Infection Control and Hospital Epidemiology..* 2021. #volume#:#pages#

**Leung, W. S., Chan, J. M. C., Chik, T. S. H., Lau, D. P. L., Choi, C. Y. C., Lau, A. W. T., Tsang, O. T. Y.**. Presumed COVID-19 index case on diamond princess cruise ship and evacuees to Hong Kong. *J Travel Med.* 2020. 27:#pages#

**Li, X.,Wang, Q.,Ding, P.,Cha, Y.,Mao, Y.,Ding, C.,Gu, W.,Wang, Y.,Ying, B.,Zhao, X.,Pan, L.,Li, Y.,Chang, J.,Meng, C.,Zhou, J.,Tang, Z.,Sun, R.,Deng, F.,Wang, C.,Li, L.,Wang, J.,MacIntyre, C. R.,Wu, Z.,Feng, Z.,Tang, S.,Xu, D.**. Risk factors and on-site simulation of environmental transmission of SARS-CoV-2 in the largest wholesale market of Beijing, China. *Science of the Total Environment.* 2021. 778:146040

**Lin, C. Y.,Wang, W. H.,Urbina, A. N.,Tseng, S. P.,Lu, P. L.,Chen, Y. H.,Yu, M. L.,Wang, S. F.**. Importation of SARS-CoV-2 infection leads to major COVID-19 epidemic in Taiwan. *International Journal of Infectious Diseases.* 2020. 97:240-244

**Lin, G. T.,Zhang, Y. H.,Xiao, M. F.,Wei, Y.,Chen, J. N.,Lin, D. J.,Wang, J. C.,Lin, Q. Y.,Lei, Z. X.,Zeng, Z. Q.,Li, L.,Li, H. A.,Zheng, Y.,Li, Q. Q.,Zhen, H. Z.,Jin, Y. M.,Wu, Q. X.,Zhang, F.,Xiang, W.**. Epidemiological investigation of a COVID-19 family cluster outbreak transmitted by a 3-month-old infant. *Health Information Science and Systems [Electronic Resource].* 2021. 9:6

**Lin, G.,Zhang, S.,Zhong, Y.,Zhang, L.,Ai, S.,Li, K.,Su, W.,Cao, L.,Zhao, Y.,Tian, F.,Li, J.,Wu, Y.,Guo, C.,Peng, R.,Wu, X.,Gan, P.,Zhu, W.,Lin, H.,Zhang, Z.**. Community evidence of severe acute respiratory syndrome coronavirus 2 (SARS-CoV-2) transmission through air. *Atmospheric Environment.* 2021. 246 (no pagination):#pages#

**Lin, J.,Yan, K.,Zhang, J.,Cai, T.,Zheng, J.**. A super-spreader of COVID-19 in Ningbo city in China. *Journal of Infection and Public Health.* 2020. 13:935-937

**Luo, K.,Lei, Z.,Hai, Z.,Xiao, S.,Rui, J.,Yang, H.,Jing, X.,Wang, H.,Xie, Z.,Luo, P.,Li, W.,Li, Q.,Tan, H.,Xu, Z.,Yang, Y.,Hu, S.,Chen, T.**. Transmission of SARS-CoV-2 in Public Transportation Vehicles: A Case Study in Hunan Province, China. *Open Forum Infectious Diseases.* 2020. 7:ofaa430

**Ma, H.,Zhang, J.,Wang, J.,Qin, Y.,Chen, C.,Song, Y.,Wang, L.,Meng, J.,Mao, L.,Li, F.,Li, N.,Cai, J.,Zhang, Y.,Wang, D.,Xia, Y.,Wang, H.,Jiang, S.,Zhao, X.,Niu, P.,Tan, W.,Ma, T.,Yao, Y.,Mao, N.,Zhu, Z.,Ji, T.,Yang, Q.,Huang, B.,Zhao, L.,Yu, J.,Bai, L.,Zhu, S.,Wang, D.,Zhang, Y.,Sun, Y.,Luan, M.,Wang, Y.,Sun, H.,Yang, S.,Bo, Z.,Ren, X.,Li, Z.,Gao, G. F.,Yao, W.,Yao, W.,Feng, Z.,Xu, W.**. COVID-19 Outbreak Caused by Contaminated Packaging of Imported Cold-Chain Products - Liaoning Province, China, July 2020. *China CDC Weekly.* 2021. 3:441-447

**Matthias, J.,Patrick, S.,Wiringa, A.,Pullman, A.,Hinton, S.,Campos, J.,Belville, T.,Sinner Mph, M.,Buchanan, T. T.,Sim, B.,Goldesberry, K. E.**. Epidemiologically Linked COVID-19 Outbreaks at a Youth Camp and Men's Conference - Illinois, June-July 2021. *MMWR - Morbidity & Mortality Weekly Report.* 2021. 70:1223-1227

**Micheli, V.,Mancon, A.,Malara, A.,Mileto, D.,Villani, P. G.,Rizzo, A.,Pagani, C.,Alquati, O.,Gismondo, M. R.**. What was behind the first recognition and characterization of autochthonous SARS-CoV-2 transmission in Italy: The impact on European scenario. *Clinical Case Reports.* 2021. 9(6) (no pagination):#pages#

**Miller, S. L.,Nazaroff, W. W.,Jimenez, J. L.,Boerstra, A.,Buonanno, G.,Dancer, S. J.,Kurnitski, J.,Marr, L. C.,Morawska, L.,Noakes, C.**. Transmission of SARS-CoV-2 by inhalation of respiratory aerosol in the Skagit Valley Chorale superspreading event. *Indoor Air.* 2021. 31:314-323

**Moreno, G. K.,Braun, K. M.,Pray, I. W.,Segaloff, H. E.,Lim, A.,Poulsen, K.,Meiman, J.,Borcher, J.,Westergaard, R. P.,Moll, M. K.,Friedrich, T. C.,O'Connor, D. H.**. Severe Acute Respiratory Syndrome Coronavirus 2 Transmission in Intercollegiate Athletics Not Fully Mitigated With Daily Antigen Testing. *Clinical Infectious Diseases.* 2021. 73:S45-S53

**Moreno, T., Gibbons, W.**. Carbon dioxide, COVID-19 and the importance of restaurant ventilation: a case study from Spain approaching Christmas 2021. *medRxiv..* 2021. 19:#pages#

**Murphy, N.,Boland, M.,Bambury, N.,Fitzgerald, M.,Comerford, L.,Dever, N.,O'Sullivan, M. B.,Petty-Saphon, N.,Kiernan, R.,Jensen, M.,O'Connor, L.**. A large national outbreak of COVID-19 linked to air travel, Ireland, summer 2020. *Euro Surveillance: Bulletin Europeen sur les Maladies Transmissibles = European Communicable Disease Bulletin.* 2020. 25:10

**Nakstad, B., Kaang, T., Gezmu, A. M., Strysko, J.**. Nosocomial SARS-CoV-2 transmission in a neonatal unit in Botswana: Chronic overcrowding meets a novel pathogen. *BMJ Case Reports.* 2021. 14(6) (no pagination):#pages#

**Nannu Shankar, S.,Witanachchi, C. T.,Morea, A. F.,Lednicky, J. A.,Loeb, J. C.,Alam, M. M.,Fan, Z. H.,Eiguren-Fernandez, A.,Wu, C. Y.**. SARS-CoV-2 in residential rooms of two self-isolating persons with COVID-19. *Journal of Aerosol Science.* 2022. 159:105870

**Okarska-Napierala, M.,Mandziuk, J.,Kuchar, E.**. SARS-CoV-2 Cluster in Nursery, Poland. *Emerging Infectious Diseases.* 2021. 27:#pages#

**Pantic, N.,Mitrovic, M.,Sabljic, N.,Vucic, M.,Bukumiric, Z.,Virijevic, M.,Pravdic, Z.,Rajic, J.,Balint, M. T.,Vidovic, A.,Tomin, D.,Vukovic, N. S.**. Acute leukemia and sars-cov-2 infection: Single centre experience. *HemaSphere.* 2021. 5(SUPPL 2):385

**Park, S. Y.,Kim, Y. M.,Yi, S.,Lee, S.,Na, B. J.,Kim, C. B.,Kim, J. I.,Kim, H. S.,Kim, Y. B.,Park, Y.,Huh, I. S.,Kim, H. K.,Yoon, H. J.,Jang, H.,Kim, K.,Chang, Y.,Kim, I.,Lee, H.,Gwack, J.,Kim, S. S.,Kim, M.,Kweon, S.,Choe, Y. J.,Park, O.,Park, Y. J.,Jeong, E. K.**. Coronavirus Disease Outbreak in Call Center, South Korea. *Emerging Infectious Diseases.* 2020. 26:1666-1670

**Parkulo, M. A.,Brinker, T. M.,Bosch, W.,Palaj, A.,DeRuyter, M. L.**. Risk of SARS-CoV-2 Transmission Among Coworkers in a Surgical Environment. *Mayo Clinic Proceedings.* 2021. 96:152-155

**Pavli, A.,Smeti, P.,Papadima, K.,Andreopoulou, A.,Hadjianastasiou, S.,Triantafillou, E.,Vakali, A.,Kefaloudi, C.,Pervanidou, D.,Gogos, C.,Maltezou, H. C.**. A cluster of COVID-19 in pilgrims to Israel. *Journal of Travel Medicine.* 2020. 27:20

**Pedersen, S. H.**. Covid-19 outbreak in a geriatric department. An observational Study. *European Geriatric Medicine.* 2020. 11(SUPPL 1):S82

**Perez-Lago, L.,Martinez-Lozano, H.,Pajares-Diaz, J. A.,Diaz-Gomez, A.,Machado, M.,Sola-Campoy, P. J.,Herranz, M.,Valerio, M.,Olmedo, M.,Suarez-Gonzalez, J.,Quesada-Cubo, V.,Gomez-Ruiz, M. D. M.,Lopez-Fresnena, N.,Sanchez-Arcilla, I.,Comas, I.,Gonzalez-Candelas, F.,Garcia de San Jose, S.,Banares, R.,Catalan, P.,Munoz, P.,Garcia de Viedma, D.,Gregorio Maranon Microbiology, I. D. Covid-Study Group**. Overlapping of Independent SARS-CoV-2 Nosocomial Transmissions in a Complex Outbreak. *Msphere.* 2021. 6:e0038921

**Periyasamy, P.,Ng, B. H.,Ali, U. K.,Rashid, Z. Z.,Kori, N.**. Aerosolized SARS-CoV-2 transmission risk: Surgical or N95 masks?. *Infection Control & Hospital Epidemiology.* 2021. 42:1150-1152

**Putallaz, P.,Bosshard, W.,Papadimitriou, M.,Senn, L.,Bula, C.**. Nosocomial spread of SARS-CoV-2 in a rehabilitation facility: The silent threat of asymptomatic patients with high viral load?. *European Geriatric Medicine.* 2020. 11(SUPPL 1):S10

**Ralli, M.,Morrone, A.,Arcangeli, A.,Ercoli, L.**. Asymptomatic patients as a source of transmission of COVID-19 in homeless shelters. *International Journal of Infectious Diseases.* 2021. 103:243-245

**Razavi, M.,Butt, Z. A.,Chen, H.,Tan, Z.**. In situ measurement of airborne particle concentration in a real dental office: implications for disease transmission. *International Journal of Environmental Research and Public Health.* 2021. 18(17) (no pagination):#pages#

**Roberts, R. J., Brooker, A., Lakshman, R.**. Outbreak of SARS-CoV-2 in a Children's Nursery in the United Kingdom. *Pediatric Infectious Disease Journal.* 2021. 40:e455-e458

**Rocklov, J.,Sjodin, H.,Wilder-Smith, A.**. COVID-19 outbreak on the diamond princess cruise ship: Estimating the epidemic potential and effectiveness of public health countermeasures. *Journal of Travel Medicine.* 2021. 27(3):1-7

**Saban, O.,Levy, J.,Chowers, I.**. Risk of SARS-CoV-2 transmission to medical staff and patients from an exposure to a COVID-19-positive ophthalmologist. *Graefes Archive for Clinical & Experimental Ophthalmology.* 2020. 258:2271-2274

**Sami, S., Horter, L., Valencia, D., Thomas, I., Pomeroy, M., Walker, B., Smith-Jeffcoat, S. E., Tate, J. E., Kirking, H. L., Kyaw, N. T. T., Burns, R., Blaney, K., Dorabawila, V., Hoen, R., Zirnhelt, Z., Schardin, C., Uehara, A., Retchless, A. C., Brown, V. R., Gebru, Y., Powell, C., Bart, S. M., Vostok, J., Lund, H., Kaess, J., Gumke, M., Propper, R., Thomas, D., Ojo, M., Green, A., Wieck, M., Wilson, E., Hollingshead, R. J., Nunez, S. V., Saady, D. M., Porse, C. C., Gardner, K., Drociuk, D., Scott, J., Perez, T., Collins, J., Shaffner, J., Pray, I., Rust, L. T., Brady, S., Kerins, J. L., Teran, R. A., Hughes, V., Sepcic, V., Low, E. W., Kemble, S. K., Berkley, A., Cleavinger, K., Safi, H., Webb, L. M., Hutton, S., Dewart, C., Dickerson, K., Hawkins, E., Zafar, J., Krueger, A., Bushman, D., Ethridge, B., Hansen, K., Tant, J., Reed, C., Boutwell, C., Hanson, J., Gillespie, M., Donahue, M., Lane, P., Serrano, R., Hernandez, L., Dethloff, M. A., Lynfield, R., Como-Sabetti, K., Lutterloh, E., Ackelsberg, J., Ricaldi, J. N.**. Investigation of SARS-CoV-2 Transmission Associated With a Large Indoor Convention - New York City, November-December 2021. *MMWR - Morbidity & Mortality Weekly Report.* 2022. 71:243-248

**Sami, S.,Turbyfill, C. R.,Daniel-Wayman, S.,Shonkwiler, S.,Fisher, K. A.,Kuhring, M.,Patrick, A. M.,Hinton, S.,Minor, A. S.,Ricaldi, J. N.,Ezike, N.,Kauerauf, J.,Duffus, W. A.**. Community Transmission of SARS-CoV-2 Associated with a Local Bar Opening Event - Illinois, February 2021. *MMWR - Morbidity & Mortality Weekly Report.* 2021. 70:528-532

**Sanville, B.,Corbett, R.,Pidcock, W.,Hardin, K.,Sebat, C.,Nguyen, M. V.,Thompson, G. R.,Haczku, A.,Schivo, M.,Cohen, S.**. A Community-transmitted Case of Severe Acute Respiratory Distress Syndrome (SARS) Due to SARS-CoV-2 in the United States. *Clinical Infectious Diseases.* 2020. 71:2222-2226

**Sarti, D.,Campanelli, T.,Rondina, T.,Gasperini, B.**. COVID-19 in Workplaces: Secondary Transmission. *Annals of Work Exposures And Health.* 2021. 10:10

**Schwartz, N. G.,Moorman, A. C.,Makaretz, A.,Chang, K. T.,Chu, V. T.,Szablewski, C. M.,Yousaf, A. R.,Brown, M. M.,Clyne, A.,DellaGrotta, A.,Drobeniuc, J.,Korpics, J.,Muir, A.,Drenzek, C.,Bandy, U.,Kirking, H. L.,Tate, J. E.,Hall, A. J.,Lanzieri, T. M.,Stewart, R. J.**. Adolescent with COVID-19 as the Source of an Outbreak at a 3-Week Family Gathering - Four States, June-July 2020. *MMWR - Morbidity & Mortality Weekly Report.* 2020. 69:1457-1459

**Schwierzeck, V.,Konig, J. C.,Kuhn, J.,Mellmann, A.,Correa-Martinez, C. L.,Omran, H.,Konrad, M.,Kaiser, T.,Kampmeier, S.**. First Reported Nosocomial Outbreak of Severe Acute Respiratory Syndrome Coronavirus 2 in a Pediatric Dialysis Unit. *Clinical Infectious Diseases.* 2021. 72:265-270

**Selhorst, P.,Van Ierssel, S.,Michiels, J.,Marien, J.,Bartholomeeusen, K.,Dirinck, E.,Vandamme, S.,Jansens, H.,Arien, K. K.**. Symptomatic SARS-CoV-2 reinfection of a health care worker in a Belgian nosocomial outbreak despite primary neutralizing antibody response. *Clinical Infectious Diseases.* 2020. 14:14

**Shen, Y.,Li, C.,Dong, H.,Wang, Z.,Martinez, L.,Sun, Z.,Handel, A.,Chen, Z.,Chen, E.,Ebell, M. H.,Wang, F.,Yi, B.,Wang, H.,Wang, X.,Wang, A.,Chen, B.,Qi, Y.,Liang, L.,Li, Y.,Ling, F.,Chen, J.,Xu, G.**. Community Outbreak Investigation of SARS-CoV-2 Transmission among Bus Riders in Eastern China. *JAMA Internal Medicine.* 2020. 180(12):1665-1671

**Shen, Y.,Xu, W.,Li, C.,Handel, A.,Martinez, L.,Ling, F.,Ebell, M.,Fu, X.,Pan, J.,Ren, J.,Gu, W.,Chen, E.**. A Cluster of Novel Coronavirus Disease 2019 Infections Indicating Person-to-Person Transmission Among Casual Contacts From Social Gatherings: An Outbreak Case-Contact Investigation. *Open Forum Infectious Diseases.* 2020. 7:ofaa231

**Shuklaa, S.,Khana, R.,Ahmedb, Y.,Memishc, Z. A.**. Conducting mass gathering events during the COVID-19 pandemic: A case study of Kumbh Mela 2021 as a potential 'super spreader event'. *Journal of Travel Medicine.* 2021. 05:05

**Smith-Jeffcoat, S. E., Pomeroy, M. A., Sleweon, S., Sami, S., Ricaldi, J. N., Gebru, Y., Walker, B., Brady, S., Christenberry, M., Bart, S., Vostok, J., Meyer, S., Seys, S., Markelz, A., Ditto, N., Newbern, V., Thomas, F. J., Thomas, D., Cabredo, E., Kellner, S., Brown, V. R., Tate, J. E., Kirking, H. L.**. Multistate Outbreak of SARS-CoV-2 B.1.1.529 (Omicron) Variant Infections Among Persons in a Social Network Attending a Convention - New York City, November 18-December 20, 2021. *MMWR - Morbidity & Mortality Weekly Report.* 2022. 71:238-242

**Streeck, H.,Schulte, B.,Kummerer, B. M.,Richter, E.,Holler, T.,Fuhrmann, C.,Bartok, E.,Dolscheid-Pommerich, R.,Berger, M.,Wessendorf, L.,Eschbach-Bludau, M.,Kellings, A.,Schwaiger, A.,Coenen, M.,Hoffmann, P.,Stoffel-Wagner, B.,Nothen, M. M.,Eis-Hubinger, A. M.,Exner, M.,Schmithausen, R. M.,Schmid, M.,Hartmann, G.**. Infection fatality rate of SARS-CoV2 in a super-spreading event in Germany. *Nature communications.* 2020. 11:5829

**Sugano, N.,Ando, W.,Fukushima, W.**. Cluster of Severe Acute Respiratory Syndrome Coronavirus 2 Infections Linked to Music Clubs in Osaka, Japan. *Journal of Infectious Diseases.* 2020. 222:1635-1640

**Suhs, T.,Gerlach, D.,Garfin, J.,Lorentz, A.,Firestone, M.,Sherden, M.,Hackman, K.,Gray, T.,Siebman, S.,Wienkes, H.,Vilen, K.,Wang, X.,Como-Sabetti, K.,Danila, R.,Smith, K.,Medus, C.**. COVID-19 Outbreak Associated with a Fitness Center - Minnesota, September-November 2020. *Clinical Infectious Diseases.* 2021. 23:23

**Suminski, R. R.,Dominick, G. M.,Wagner, N. J.**. A direct observation video method for describing covid-19 transmission factors on a micro-geographical scale: Viral transmission (vt)-scan. *International Journal of Environmental Research and Public Health.* 2021. 18(17) (no pagination):#pages#

**Sun, D.,Zhu, F.,Wang, C.,Wu, J.,Liu, J.,Chen, X.,Liu, Z.,Wu, Z.,Lu, X.,Ma, J.,Peng, H.,Xiao, H.**. Children Infected With SARS-CoV-2 From Family Clusters. *Frontiers in Pediatrics.* 2020. 8:386

**Szablewski, C. M.,Chang, K. T.,McDaniel, C. J.,Chu, V. T.,Yousaf, A. R.,Schwartz, N. G.,Brown, M.,Winglee, K.,Paul, P.,Cui, Z.,Slayton, R. B.,Tong, S.,Li, Y.,Uehara, A.,Zhang, J.,Sharkey, S. M.,Kirking, H. L.,Tate, J. E.,Dirlikov, E.,Fry, A. M.,Hall, A. J.,Rose, D. A.,Villanueva, J.,Drenzek, C.,Stewart, R. J.,Lanzieri, T. M.,Adebayo, A.,Aholou, T. M.,Amin, M. M.,Aryee, P.,Castaneda, C.,Chambers, T. V.,Fleshman, A. C.,Goodman, C.,Holmes, T.,Ivey-Stephenson, A.,Kamitani, E.,Katz, S.,Knapp, J. K.,Kolasa, M.,Lumsden, M. F.,Mayweather, E.,Mohammed, A.,Moorman, A. C.,PatelLarson, A.,Perinet, L. C.,Pilgard, M.,Pratt, D. D.,Railey, S.,Shah, J.,Tuckey, D.**. SARS-CoV-2 transmission dynamics in a sleep-away camp. *Pediatrics.* 2021. 147(4) (no pagination):#pages#

**Temte, J. L., Barlow, S., Temte, E., Goss, M., Florek, K., Braun, K. M., Friedrich, T. C., Reisdorf, E., Bateman, A. C., Uzicanin, A.**. Evidence of Early Household Transmission of SARS-CoV-2 Involving a School-aged Child. *WMJ.* 2021. 120:233-236

**Teo, W. Y.**. Implications for border containment strategies when COVID-19 presents atypically. *Public Health.* 2020. 186:193-196

**Teran, R. A.,Ghinai, I.,Gretsch, S.,Cable, T.,Black, S. R.,Green, S. J.,Perez, O.,Chlipala, G. E.,Maienschein-Cline, M.,Kunstman, K. J.,Bleasdale, S. C.,Fricchione, M. J.**. COVID-19 Outbreak Among a University's Men's and Women's Soccer Teams - Chicago, Illinois, July-August 2020. *MMWR - Morbidity & Mortality Weekly Report.* 2020. 69:1591-1594

**Tian, S.,Wu, M.,Chang, Z.,Wang, Y.,Zhou, G.,Zhang, W.,Xing, J.,Tian, H.,Zhang, X.,Zou, X.,Zhang, L.,Liu, M.,Chen, J.,Han, J.,Ning, K.,Chen, S.,Wu, T.**. Epidemiological investigation and intergenerational clinical characteristics of 24 coronavirus disease patients associated with a supermarket cluster: a retrospective study. *BMC Public Health.* 2021. 21:647

**Toyokawa, T., Shimada, T., Hayamizu, T., Sekizuka, T., Zukeyama, Y., Yasuda, M., Nakamura, Y., Okano, S., Kudaka, J., Kakita, T., Kuroda, M., Nakasone, T.**. Transmission of SARS-CoV-2 during a 2-h domestic flight to Okinawa, Japan, March 2020. *Influenza and other Respiratory Viruses.* 2022. 16(1):63-71

**Vivian Thangaraj, J. W.,Murhekar, M.,Mehta, Y.,Kataria, S.,Brijwal, M.,Gupta, N.,Choudhary, A.,Malhotra, B.,Vyas, M.,Sharma, H.,Yadav, N.,Bhatnagar, T.,Gupta, N.,Dar, L.,Gangakhedkar, R. R.,Bhargava, B.**. A cluster of SARS-CoV-2 infection among Italian tourists visiting India, March 2020. *Indian Journal of Medical Research.* 2020. 151:438-443

**Vlacha, V.,Feketea, G.,Petropoulou, A.,Tranca, S. D.**. The Significance of Duration of Exposure and Circulation of Fresh Air in SARS-CoV-2 Transmission Among Healthcare Workers. *Frontiers in Medicine.* 2021. 8 (no pagination):#pages#

**Voeten, Hacm,Sikkema, R. S.,Damen, M.,Oude Munnink, B. B.,Arends, C.,Stobberingh, E.,Hoogervorst, E.,Koopmans, M. P. G.,Fanoy, E.**. Unraveling the Modes of Transmission of Severe Acute Respiratory Syndrome Coronavirus 2 (SARS-CoV-2) During a Nursing Home Outbreak: Looking Beyond the Church Superspreading Event. *Clinical Infectious Diseases.* 2021. 73:S163-S169

**Wallace, M.,James, A. E.,Silver, R.,Koh, M.,Tobolowsky, F. A.,Simonson, S.,Gold, J. A. W.,Fukunaga, R.,Njuguna, H.,Bordelon, K.,Wortham, J.,Coughlin, M.,Harcourt, J. L.,Tamin, A.,Whitaker, B.,Thornburg, N. J.,Tao, Y.,Queen, K.,Uehara, A.,Paden, C. R.,Zhang, J.,Tong, S.,Haydel, D.,Tran, H.,Kim, K.,Fisher, K. A.,Marlow, M.,Tate, J. E.,Doshi, R. H.,Sokol, T.,Curran, K. G.**. Rapid transmission of severe acute respiratory syndrome coronavirus 2 in detention facility, Louisiana, USA, May-June, 2020. *Emerging Infectious Diseases.* 2021. 27(2):421-429

**Walshe, N., Fennelly, M., Hellebust, S., Wenger, J., Sodeau, J., Prentice, M., Grice, C., Jordan, V., Comerford, J., Downey, V., Perrotta, C., Mulcahy, G., Sammin, D.**. Assessment of Environmental and Occupational Risk Factors for the Mitigation and Containment of a COVID-19 Outbreak in a Meat Processing Plant. *Frontiers in Public Health.* 2021. 9:769238

**Wang, X.,Jiang, X.,Huang, Q.,Wang, H.,Gurarie, D.,Ndeffo-Mbah, M.,Fan, F.,Fu, P.,Horn, M. A.,Mondal, A.,King, C.,Xu, S.,Zhao, H.,Bai, Y.**. Risk factors of SARS-CoV-2 infection in healthcare workers: a retrospective study of a nosocomial outbreak. *Sleep Medicine: X.* 2020. 2:100028

**Wei, X. S.,Wang, X. R.,Zhang, J. C.,Yang, W. B.,Ma, W. L.,Yang, B. H.,Jiang, N. C.,Gao, Z. C.,Shi, H. Z.,Zhou, Q.**. A cluster of health care workers with COVID-19 pneumonia caused by SARS-CoV-2. *Journal of Microbiology, Immunology & Infection.* 2021. 54:54-60

**Weissberg, D.,Boni, J.,Rampini, S. K.,Kufner, V.,Zaheri, M.,Schreiber, P. W.,Abela, I. A.,Huber, M.,Sax, H.,Wolfensberger, A.**. Does respiratory co-infection facilitate dispersal of SARS-CoV-2? investigation of a super-spreading event in an open-space office. *Antimicrobial Resistance & Infection Control.* 2020. 9:191

**Wendt, R.,Nagel, S.,Nickel, O.,Wolf, J.,Kalbitz, S.,Kaiser, T.,Borte, S.,Lubbert, C.**. Comprehensive investigation of an in-hospital transmission cluster of a symptomatic SARS-CoV-2-positive physician among patients and healthcare workers in Germany. *Infection Control & Hospital Epidemiology.* 2020. 41:1209-1211

**Wenlock, R. D.,Tausan, M.,Mann, R.,Garr, W.,Preston, R.,Arnold, A.,Hoban, J.,Webb, L.,Quick, C.,Beckett, A.,Loveson, K.,Glaysher, S.,Elliott, S.,Malone, C.,Cogger, B.,Easton, L.,Covid- Genomics UK consortium,Robson, S. C.,Hassan-Ibrahim, M. O.,Sargent, C.**. Nosocomial or not? A combined epidemiological and genomic investigation to understand hospital-acquired COVID-19 infection on an elderly care ward. *Infection Prevention in Practice.* 2021. 3:100165

**Wilburn, J.,Blakey, E.,Trindall, A.,Burr, H.,Tanti, V.,Doolan, S.,Palmer, I.,Jewell, T.,Balakrishnan, R.**. COVID-19 within a large UK prison with a high number of vulnerable adults, march to june 2020: An outbreak investigation and screening event. *International Journal of Infectious Diseases.* 2021. 104:349-353

**Wong, J.,Abdul Aziz, A. B. Z.,Chaw, L.,Mahamud, A.,Griffith, M. M.,Lo, Y. R.,Naing, L.**. High proportion of asymptomatic and presymptomatic COVID-19 infections in air passengers to Brunei. *Journal of Travel Medicine.* 2020. 27:20

**Yamanoglu, A.,Akyol, P. Y.,Acar, H.,Celebi Yamanoglu, N. G.,Topal, F. E.,Kayali, A.**. How would you like your COVID-19? From a host with mild course disease, or from a severe one?. *American Journal of Emergency Medicine.* 2020. 38(11):2487.e7-2487.e12

**Yang, N.,Shen, Y.,Shi, C.,Ma, A. H. Y.,Zhang, X.,Jian, X.,Wang, L.,Shi, J.,Wu, C.,Li, G.,Fu, Y.,Wang, K.,Lu, M.,Qian, G.**. In-flight transmission cluster of COVID-19: a retrospective case series. *Infectious Diseases.* 2020. 52:891-901

**Yao, L.,Luo, M.,Jia, T.,Zhang, X.,Hou, Z.,Gao, F.,Wang, X.,Wu, X.,Cheng, W.,Li, G.,Lu, J.,Zhao, B.,Li, T.,Chen, E.,Yin, D.,Huang, B.**. COVID-19 Super Spreading Event Amongst Elderly Individuals - Jilin Province, China, January 2021. *China CDC Weekly.* 2021. 3:211-213

**Yu, P.,Zhu, J.,Zhang, Z.,Han, Y.**. A Familial Cluster of Infection Associated With the 2019 Novel Coronavirus Indicating Possible Person-to-Person Transmission During the Incubation Period. *Journal of Infectious Diseases.* 2020. 221:1757-1761

**Yue, Y.,Chen, Y.,Du, X.,Jin, Y.,Hu, M.,Jiang, X.,Wang, C.,Chen, Z.,Su, L.,Chen, C.,Jiang, S.,Tuo, X.**. A survey of a COVID-19 cluster of charter flight importation. *Public Health.* 2021. 199:107-109

**Zhang, H.,Chen, R.,Chen, J.,Chen, B.**. COVID-19 Transmission Within a Family Cluster in Yancheng, China. *Frontiers in Medicine.* 2020. 7:387

**Zhang, J.,Tian, S.,Lou, J.,Chen, Y.**. Familial cluster of COVID-19 infection from an asymptomatic. *Critical Care (London, England).* 2020. 24:119

**Zhang, J.,Wu, S.,Xu, L.**. Asymptomatic carriers of COVID-19 as a concern for disease prevention and control: more testing, more follow-up. *Bioscience Trends.* 2020. 14:206-208

**Zhang, N.,Chen, X.,Jia, W.,Jin, T.,Xiao, S.,Chen, W.,Hang, J.,Ou, C.,Lei, H.,Qian, H.,Su, B.,Li, J.,Liu, D.,Zhang, W.,Xue, P.,Liu, J.,Weschler, L. B.,Xie, J.,Li, Y.,Kang, M.**. Evidence for lack of transmission by close contact and surface touch in a restaurant outbreak of COVID-19. *Journal of Infection.* 2021. 83(2):207-216

**Zhang, Y.,Cao, X.,Ma, J.,Zhu, L.,Chen, J.**. A case describing patients with COVID-19 that secondarily transmitted. *Annals of Palliative Medicine.* 2021. 10:8972-8981

**Zhou, Y.,Teng, Z.,Chen, H.,Cui, X.,Fang, F.,Mou, J.,Jiang, H.,Zhang, X.**. Virology features of a family cluster of SARS-CoV-2 infections in Shanghai, China. *Biosafety and Health.* 2021. 3:187-189

**Bagdasarian, N.,Fisher, D.**. Heterogenous COVID-19 transmission dynamics within Singapore: A clearer picture of future national responses. *BMC Medicine.* 2020. 18:#pages#

**Bai, Y.,Yao, L.,Wei, T.,Tian, F.,Jin, D. Y.,Chen, L.,Wang, M.**. Presumed Asymptomatic Carrier Transmission of COVID-19. *JAMA.* 2020. 323:1406-1407

**Banerjee, I., Robinson, J.**. COVID-19 airborne transmission: a new frontier of infection. *Nepal Journal of Epidemiology.* 2021. 11(3):1076-1078

**Banik, R. K.,Ulrich, A.**. Evidence of Short-Range Aerosol Transmission of SARS-CoV-2 and Call for Universal Airborne Precautions for Anesthesiologists During the COVID-19 Pandemic. *Anesthesia & Analgesia.* 2020. 131:e102-e104

**Bax, A.,Bax, C. E.,Stadnytskyi, V.,Anfinrud, P.**. SARS-CoV-2 transmission via speech-generated respiratory droplets. *The Lancet Infectious Diseases.* 2021. 21(3):318

**Becker, E.,Vipond, R.,Mansell, C.**. Keeping up with COVID: identification of New Zealand's earliest known cluster of COVID-19 cases. *New Zealand Medical Journal.* 2021. 134:83-85

**Behjati, M.**. A simple complementary approach to reduce risk of contamination with COVID-19 during transesophageal echocardiography. *Journal of Echocardiography.* 2021. 19(4):269-270

**Berkow, L.,Kanowitz, A.**. COVID-19 Putting Patients at Risk of Unplanned Extubation and Airway Providers at Increased Risk of Contamination. *Anesthesia & Analgesia.* 2020. 131:e41-e43

**Bhuvan, K. C.,Shrestha, R.,Leggat, P. A.,Ravi Shankar, P.,Shrestha, S.**. Safety of air travel during the ongoing COVID-19 pandemic. *Travel Medicine and Infectious Disease.* 2021. 43 (no pagination):#pages#

**Bistoquet, M.,Galtier, F.,Marin, G.,Villard, O.,Ferreira, R.,Hermabessiere, S.,Montoya, A.,Jumas-Bilak, E.,Pageaux, G. P.,Dereure, O.,Chanques, G.,Klouche, K.,Morquin, D.,Reynes, J.,Le Moing, V.,Picot, M. C.,Tuaillon, E.,Makinson, A.**. Increased risks of SARS-CoV-2 nosocomial acquisition in high-risk COVID-19 units justify personal protective equipment: a cross-sectional study. *Journal of Hospital Infection.* 2021. 107:108-110

**Bo, M.,Brunetti, E.,Presta, R.,Rota, M.,Dutto, F.,Cortese, A.,Isaia, G.**. To keep a COVID-19-free hospital ward: mission possible?. *Aging-Clinical & Experimental Research.* 2020. 32:1627-1628

**Bouza, E.,Perez-Granda, M. J.,Escribano, P.,Fernandez-del-Rey, R.,Pastor, I.,Moure, Z.,Catalan, P.,Alonso, R.,Munoz, P.,Guinea, J.**. Outbreak of COVID-19 in a nursing home in Madrid. *Journal of Infection.* 2020. 81(4):647-679

**Braithwaite, I., Edge, C., Lewer, D., Hard, J.**. High COVID-19 death rates in prisons in England and Wales, and the need for early vaccination. *The Lancet Respiratory Medicine.* 2021. 9(6):569-570

**Brooks, J. T.,Butler, J. C.**. Effectiveness of Mask Wearing to Control Community Spread of SARS-CoV-2. *JAMA - Journal of the American Medical Association.* 2021. 325(10):998-999

**Buonsenso, D.,Graglia, B.**. High rates of SARS-CoV-2 transmission in a high-school class. *Journal of Paediatrics and Child Health.* 2021. 57(2):299-300

**Bahl, P.,de Silva, C.,Bhattacharjee, S.,Stone, H.,Doolan, C.,Chughtai, A. A.,MacIntyre, C. R.**. Droplets and Aerosols Generated by Singing and the Risk of Coronavirus Disease 2019 for Choirs. *Clinical Infectious Diseases.* 2021. 72:e639-e641

**Baker, M. G.,Peckham, T. K.,Seixas, N. S.**. Estimating the burden of United States workers exposed to infection or disease: A key factor in containing risk of COVID-19 infection. *PLoS ONE [Electronic Resource].* 2020. 15:e0232452

**Baniasad, M.,Mofrad, M. G.,Bahmanabadi, B.,Jamshidi, S.**. COVID-19 in Asia: Transmission factors, re-opening policies, and vaccination simulation. *Environmental Research.* 2021. 202 (no pagination):#pages#

**Beaussier, M.,Vanoli, E.,Zadegan, F.,Peray, H.,Bezian, E.,Jilesen, J.,Gandveau, G.,Gayraud, J. M.**. Aerodynamic analysis of hospital ventilation according to seasonal variations. A simulation approach to prevent airborne viral transmission pathway during Covid-19 pandemic. *Environment International.* 2022. 158 (no pagination):#pages#

**Belosi, F.,Conte, M.,Gianelle, V.,Santachiara, G.,Contini, D.**. On the concentration of SARS-CoV-2 in outdoor air and the interaction with pre-existing atmospheric particles. *Environmental Research.* 2021. 193 (no pagination):#pages#

**Benzell, S. G.,Collis, A.,Nicolaides, C.**. The Efficiency of U.S. Public Space Utilization During the COVID-19 Pandemic. *Risk Analysis.* 2021. 22:22

**Benzell, S. G.,Collis, A.,Nicolaides, C.**. Rationing social contact during the COVID-19 pandemic: Transmission risk and social benefits of US locations. *Proceedings of the National Academy of Sciences of the United States of America.* 2020. 117:14642-14644

**Bhardwaj, R.,Bangia, A.**. Data driven estimation of novel COVID-19 transmission risks through hybrid soft-computing techniques. *Chaos Solitons & Fractals.* 2020. 140:110152

**Bi, Q.,Lessler, J.,Eckerle, I.,Lauer, S. A.,Kaiser, L.,Vuilleumier, N.,Cummings, D. A. T.,Flahault, A.,Petrovic, D.,Guessous, I.,Stringhini, S.,Azman, A. S.,S. EROCoV-POP Study Group**. Insights into household transmission of SARS-CoV-2 from a population-based serological survey. *Nature communications.* 2021. 12:3643

**Bilinski, A.,Salomon, J. A.,Giardina, J.,Ciaranello, A.,Fitzpatrick, M. C.**. Passing the Test: A Model-Based Analysis of Safe School-Reopening Strategies. *Annals of Internal Medicine.* 2021. 174:1090-1100

**Boccuni, F., Buresti, G., Gagliardi, D., Rondinone, B. M., Persechino, B., Petyx, M., Valenti, A., Iavicoli, S.**. Risk analysis of Covid-19 in the workplace: practical approaches by activity sector in Italy. *Safety and Health at Work.* 2022. 13(Supplement):S77

**Bracis, C.,Burns, E.,Moore, M.,Swan, D.,Reeves, D. B.,Schiffer, J. T.,Dimitrov, D.**. Widespread testing, case isolation and contact tracing may allow safe school reopening with continued moderate physical distancing: A modeling analysis of King County, WA data. *Infectious Disease Modelling.* 2021. 6:24-35

**Brainard, J.,Rushton, S.,Winters, T.,Hunter, P. R.**. Introduction to and spread of COVID-19-like illness in care homes in Norfolk, UK. *Journal of Public Health.* 2021. 43:228-235

**Brooks-Pollock, E., Christensen, H., Trickey, A., Hemani, G., Nixon, E., Thomas, A. C., Turner, K., Finn, A., Hickman, M., Relton, C., Danon, L.**. High COVID-19 transmission potential associated with re-opening universities can be mitigated with layered interventions. *Nature Communications.* 2021. 12(1) (no pagination):#pages#

**Brosseau, L. M.,Rosen, J.,Harrison, R.**. Selecting Controls for Minimizing SARS-CoV-2 Aerosol Transmission in Workplaces and Conserving Respiratory Protective Equipment Supplies. *Annals of Work Exposures And Health.* 2021. 65:53-62

**Buonanno, G.,Stabile, L.,Morawska, L.**. Estimation of airborne viral emission: Quanta emission rate of SARS-CoV-2 for infection risk assessment. *Environment International.* 2020. 141 (no pagination):#pages#

**Baskaran, T. P.,Raghav, P.,K, H. N.,Saurabh, S.**. Enabling Readiness of a School to Reopen during a Pandemic - A Field Experience. *Disaster medicine and public health preparedness.* 2021. #volume#:1-14

**Baylis, P., Beauregard, P. L., Connolly, M., Fortin, N. M., Green, D. A., Gutierrez-Cubillos, P., Gyetvay, S., Haeck, C., Molnar, T. L., Simard-Duplain, G., Siu, H. E., teNyenhuis, M., Warman, C.**. The distribution of COVID-19-related risks. *Canadian Journal of Economics.* 2021. 16:16

**Beggs, C. B.,Avital, E. J.**. Upper-room ultraviolet air disinfection might help to reduce COVID-19 transmission in buildings: A feasibility study. *PeerJ.* 2020. 8 (no pagination):#pages#

**Biswas Mukherjee, S.,Gorohovski, A.,Merzon, E.,Levy, E.,Mukherjee, S.,Frenkel-Morgenstern, M.**. Seasonal UV exposure and vitamin D: Association with the dynamics of COVID-19 transmission in Europe. *FEBS Open Bio.* 2021. 05:05

**Blocken, B., van Druenen, T., van Hooff, T., Verstappen, P. A., Marchal, T., Marr, L. C.**. Can indoor sports centers be allowed to re-open during the COVID-19 pandemic based on a certificate of equivalence?. *Build Environ.* 2020. 180:107022

**Bonell, C.,Melendez-Torres, G. J.,Viner, R. M.,Rogers, M. B.,Whitworth, M.,Rutter, H.,Rubin, G. J.,Patton, G.**. An evidence-based theory of change for reducing SARS-CoV-2 transmission in reopened schools. *Health and Place.* 2020. 64 (no pagination):#pages#

**Bulfone, T. C.,Malekinejad, M.,Rutherford, G. W.,Razani, N.**. Outdoor Transmission of SARS-CoV-2 and Other Respiratory Viruses: A Systematic Review. *Journal of Infectious Diseases.* 2021. 223:550-561

**Cackett, P.,Bennett, H.**. Phacoemulsification and pars plana vitrectomy: no evidence of an increased risk of aerosol transmission. *Eye (Basingstoke).* 2021. 35(4):1274

**Cai, J.,Sun, W.,Huang, J.,Gamber, M.,Wu, J.,He, G.**. Indirect Virus Transmission in Cluster of COVID-19 Cases, Wenzhou, China, 2020. *Emerging Infectious Diseases.* 2020. 26:1343-1345

**Cao, Q.,Chen, Y. C.,Chen, C. L.,Chiu, C. H.**. SARS-CoV-2 infection in children: Transmission dynamics and clinical characteristics. *Journal of the Formosan Medical Association.* 2020. 119(3):670-673

**Cao, W.,Chen, C.,Li, M.,Nie, R.,Lu, Q.,Song, D.,Li, S.,Yang, T.,Liu, Y.,Du, B.,Wang, X.**. Important factors affecting COVID-19 transmission and fatality in metropolises. *Public Health.* 2021. 190:e21-e23

**Cappy, P.,Candotti, D.,Sauvage, V.,Lucas, Q.,Boizeau, L.,Gomez, J.,Enouf, V.,Chabli, L.,Pillonel, J.,Tiberghien, P.,Morel, P.,Laperche, S.**. No evidence of SARS-CoV-2 transfusion transmission despite RNA detection in blood donors showing symptoms after donation. *Blood.* 2020. 136(16):1888-1891

**Caruso, B. A.,Freeman, M. C.**. Shared sanitation and the spread of COVID-19: risks and next steps. *The Lancet Planetary Health.* 2020. 4(5):e173

**Chau, N. V. V.,Hong, N. T. T.,Ngoc, N. M.,Thanh, T. T.,Khanh, P. N. Q.,Nguyet, L. A.,Nhu, L. N. T.,Ny, N. T. H.,Man, D. N. H.,Hang, V. T. T.,Phong, N. T.,Que, N. T. H.,Tuyen, P. T.,Tu, T. N. H.,Hien, T. T.,Minh, N. N. Q.,Hung, L. M.,Truong, N. T.,Yen, L. M.,Rogier van Doorn, H.,Dung, N. T.,Thwaites, G.,Dung, N. T.,Van Tan, L.,Oucru Covid- research group**. Superspreading Event of SARS-CoV-2 Infection at a Bar, Ho Chi Minh City, Vietnam. *Emerging Infectious Diseases.* 2021. 27:01

**Chen, P. Z.,Koopmans, M.,Fisman, D. N.,Gu, F. X.**. Understanding why superspreading drives the COVID-19 pandemic but not the H1N1 pandemic. *The Lancet Infectious Diseases.* 2021. 21:1203-1204

**Chen, W.,Wang, J.,Cheng, W.,Li, D.,Zheng, Y.,Zhang, J.,Liu, Y.**. Hidden risk of nosocomial transmission: a presymptomatic novel coronavirus disease-19 (COVID-19) case with ischemic stroke. *Journal of Thoracic Disease.* 2020. 12:3442-3444

**Cheng, V. C. C.,Wong, S. C.,To, K. K. W.,Ho, P. L.,Yuen, K. Y.**. Preparedness and proactive infection control measures against the emerging novel coronavirus in China. *Journal of Hospital Infection.* 2020. 104(3):254-255

**Cho, H. J.,Feldman, L. S.,Keller, S.,Hoffman, A.,Pahwa, A. K.,Krouss, M.**. Choosing Wisely in the COVID-19 Era: Preventing Harm to Healthcare Workers. *Journal of Hospital Medicine (Online).* 2020. 15:360-362

**Chrimes, N.,Cook, T. M.,Harrop-Griffiths, W.**. Opening operating theatre doors after aerosol-generating procedures is not a high-risk action. *Anaesthesia.* 2021. 76(S3):12-13

**Chu, D. K. W.,Gu, H.,Chang, L. D. J.,Cheuk, S. S. Y.,Gurung, S.,Krishnan, P.,Ng, D. Y. M.,Liu, G. Y. Z.,Wan, C. K. C.,Tsang, D. N. C.,Peiris, M.,Poon, L. L. M.**. SARS-CoV-2 Superspread in Fitness Center, Hong Kong, China, March 2021. *Emerging Infectious Diseases.* 2021. 27:2230-2232

**Chu, V. T.,Yousaf, A. R.,Chang, K.,Schwartz, N. G.,McDaniel, C. J.,Lee, S. H.,Szablewski, C. M.,Brown, M.,Drenzek, C. L.,Dirlikov, E.,Rose, D. A.,Villanueva, J.,Fry, A. M.,Hall, A. J.,Kirking, H. L.,Tate, J. E.,Lanzieri, T. M.,Stewart, R. J.,Georgia Camp Investigation, Team**. Household Transmission of SARS-CoV-2 from Children and Adolescents. *New England Journal of Medicine.* 2021. 385:954-956

**Chung, H.,Kim, M. N.,Kim, W. Y.,Park, M. S.,Jung, J.,Kim, S. H.**. Risk of coronavirus disease 2019 transmission in an emergency department with multiple open beds. *Clinical Microbiology and Infection.* 2021. 27(10):1531-1533

**Cipriano, M.,Giacalone, A.,Ruberti, E.**. Sexual Behaviors During COVID-19: The Potential Risk of Transmission. *Archives of Sexual Behavior.* 2020. 49:1431-1432

**Coe, N. B.,Van Houtven, C. H.**. Living Arrangements of Older Adults and COVID-19 Risk: It Is Not Just Nursing Homes. *Journal of the American Geriatrics Society.* 2020. 68(7):1398-1399

**Conover, C. S.**. Transmission of Severe Acute Respiratory Syndrome Coronavirus 2 via Contaminated Surfaces: What Is to Be Done?. *Clinical Infectious Diseases.* 2021. 72(11):2062-2064

**Cahill, N.,Morris, D.**. Recreational waters - A potential transmission route for SARS-CoV-2 to humans?. *Science of the Total Environment.* 2020. 740 (no pagination):#pages#

**Calum, H. P.,Sode, L. P.,Pedersen, M.**. Status: nosocomial transmission and prevention of SARS-CoV-2 in a Danish context. *Apmis.* 2021. 129(7):340-351

**Carraturo, F.,Del Giudice, C.,Morelli, M.,Cerullo, V.,Libralato, G.,Galdiero, E.,Guida, M.**. Persistence of SARS-CoV-2 in the environment and COVID-19 transmission risk from environmental matrices and surfaces. *Environmental Pollution.* 2020. 265:115010

**Centers for Disease, Control, Prevention**. Considerations for Restaurant and Bar Operators. *#journal#.* 2021. #volume#:#pages#

**Cevik, M.,Marcus, J. L.,Buckee, C.,Smith, T. C.**. Severe Acute Respiratory Syndrome Coronavirus 2 (SARS-CoV-2) Transmission Dynamics Should Inform Policy. *Clinical Infectious Diseases.* 2021. 73:S170-S176

**Cevik, M.,Marcus, J. L.,Buckee, C.,Smith, T. C.**. SARS-CoV-2 transmission dynamics should inform policy. *Clinical infectious diseases : an official publication of the Infectious Diseases Society of America..* 2020. 23:#pages#

**Chen, D. A.,Lee, M.,Lelli, G. J.,Kacker, A.**. Evaluation of the aerosol generating potential of endoscopic dacryocystorhinostomy. *Laryngoscope Investigative Otolaryngology..* 2021. #volume#:#pages#

**Chiampas, G. T.,Ibiebele, A. L.**. A Sports Practitioner's Perspective on the Return to Play During the Early Months of the COVID-19 Pandemic: Lessons Learned and Next Steps. *Sports Medicine.* 2021. 13:13

**Chirico, F.,Sacco, A.,Bragazzi, N. L.,Magnavita, N.**. Can air-conditioning systems contribute to the spread of SARS/MERS/COVID-19 infection? Insights from a rapid review of the literature. *International Journal of Environmental Research and Public Health.* 2020. 17(17):1-11

**Correia, G.,Rodrigues, L.,Gameiro da Silva, M.,Goncalves, T.**. Airborne route and bad use of ventilation systems as non-negligible factors in SARS-CoV-2 transmission. *Medical Hypotheses.* 2020. 141 (no pagination):#pages#

**Czajkowska, S.,Potempa, N.,Rupa-Matysek, J.,Surdacka, A.**. Preventing the suspension of dental clinics by minimizing the risk of SARS-CoV-2 transmission during dental treatment. *Dental & Medical Problems.* 2021. 58:397-403

**Candido, D. S.,Claro, I. M.,de Jesus, J. G.,Souza, W. M.,Moreira, F. R. R.,Dellicour, S.,Mellan, T. A.,du Plessis, L.,Pereira, R. H. M.,Sales, F. C. S.,Manuli, E. R.,Theze, J.,Almeida, L.,Menezes, M. T.,Voloch, C. M.,Fumagalli, M. J.,Coletti, T. M.,da Silva, C. A. M.,Ramundo, M. S.,Amorim, M. R.,Hoeltgebaum, H. H.,Mishra, S.,Gill, M. S.,Carvalho, L. M.,Buss, L. F.,Prete, C. A.,Ashworth, J.,Nakaya, H. I.,Peixoto, P. S.,Brady, O. J.,Nicholls, S. M.,Tanuri, A.,Rossi, A. D.,Braga, C. K. V.,Gerber, A. L.,de Guimaraes, A. P. C.,Gaburo, N.,Alencar, C. S.,Ferreira, A. C. S.,Lima, C. X.,Levi, J. E.,Granato, C.,Ferreira, G. M.,Francisco, R. S.,Granja, F.,Garcia, M. T.,Moretti, M. L.,Perroud, M. W.,Castineiras, T. M. P. P.,Lazari, C. S.,Hill, S. C.,de Souza Santos, A. A.,Simeoni, C. L.,Forato, J.,Sposito, A. C.,Schreiber, A. Z.,Santos, M. N. N.,de Sa, C. Z.,Souza, R. P.,Resende-Moreira, L. C.,Teixeira, M. M.,Hubner, J.,Leme, P. A. F.,Moreira, R. G.,Nogueira, M. L.,Ferguson, N. M.,Costa, S. F.,Proenca-Modena, J. L.,Vasconcelos, A. T. R.,Bhatt, S.,Lemey, P.,Wu, C. H.,Rambaut, A.,Loman, N. J.,Aguiar, R. S.,Pybus, O. G.,Sabino, E. C.,Faria, N. R.**. Evolution and epidemic spread of SARS-CoV-2 in Brazil. *Science.* 2020. 369(6508):1255-1260

**Cardinal, R. N.,Meiser-Stedman, C. E.,Christmas, D. M.,Price, A. C.,Denman, C.,Underwood, B. R.,Chen, S.,Banerjee, S.,White, S. R.,Su, L.,Ford, T. J.,Chamberlain, S. R.,Walsh, C. M.**. Simulating a Community Mental Health Service During the COVID-19 Pandemic: Effects of Clinician-Clinician Encounters, Clinician-Patient-Family Encounters, Symptom-Triggered Protective Behaviour, and Household Clustering. *Frontiers in psychiatry Frontiers Research Foundation.* 2021. 12:620842

**Cazelles, B.,Comiskey, C.,Nguyen-Van-Yen, B.,Champagne, C.,Roche, B.**. Parallel trends in the transmission of SARS-CoV-2 and retail/recreation and public transport mobility during non-lockdown periods. *International Journal of Infectious Diseases.* 2021. 104:693-695

**Champredon, D.,Fazil, A.,Ogden, N. H.**. Simple mathematical modelling approaches to assessing the transmission risk of SARS-CoV-2 at gatherings. *Canada Communicable Disease Report.* 2021. 47:184-194

**Chang, S.,Pierson, E.,Koh, P. W.,Gerardin, J.,Redbird, B.,Grusky, D.,Leskovec, J.**. Mobility network models of COVID-19 explain inequities and inform reopening. *Nature.* 2021. 589:82-87

**Chen, L.,Ban, G.,Long, E.,Kalonji, G.,Cheng, Z.,Zhang, L.,Guo, S.**. Estimation of the SARS-CoV-2 transmission probability in confined traffic space and evaluation of the mitigation strategies. *Environmental Science & Pollution Research.* 2021. 28:42204-42216

**Cheng, P., Luo, K., Xiao, S., Yang, H., Hang, J., Ou, C., Cowling, B. J., Yen, H. L., Hui, D. S., Hu, S., Li, Y.**. Predominant airborne transmission and insignificant fomite transmission of SARS-CoV-2 in a two-bus COVID-19 outbreak originating from the same pre-symptomatic index case. *Journal of Hazardous Materials.* 2021. 425:128051

**Cheng, T., Lu, T., Liu, Y., Gao, X., Zhang, X.**. Revealing spatiotemporal transmission patterns and stages of COVID-19 in China using individual patients' trajectory data. *Computational Urban Science.* 2021. 1:9

**Chiba, A.**. The effectiveness of mobility control, shortening of restaurants' opening hours, and working from home on control of COVID-19 spread in Japan. *Health Place.* 2021. 70:102622

**Chin, W. C. B.,Bouffanais, R.**. Spatial super-spreaders and super-susceptibles in human movement networks. *Scientific Reports.* 2020. 10:18642

**Choi, S. B.,Ahn, I.**. Forecasting imported COVID-19 cases in South Korea using mobile roaming data. *PLoS ONE.* 2020. 15(11 November) (no pagination):#pages#

**Chong, K. C.,Cheng, W.,Zhao, S.,Ling, F.,Mohammad, K. N.,Wang, M.,Zee, B. C. Y.,Wei, L.,Xiong, X.,Liu, H.,Wang, J.,Chen, E.**. Transmissibility of coronavirus disease 2019 in Chinese cities with different dynamics of imported cases. *PeerJ.* 2020. 8 (no pagination):#pages#

**Clifford, S.,Quilty, B. J.,Russell, T. W.,Liu, Y.,Chan, Y. D.,Pearson, C. A. B.,Eggo, R. M.,Endo, A.,Group, Cmmid Covid-Working,Flasche, S.,Edmunds, W. J.,Centre for Mathematical Modelling of Infectious Diseases, Covid-Working Group**. Strategies to reduce the risk of SARS-CoV-2 importation from international travellers: modelling estimations for the United Kingdom, July 2020. *Euro Surveillance: Bulletin Europeen sur les Maladies Transmissibles = European Communicable Disease Bulletin.* 2021. 26:09

**Coelho, F. C.,Lana, R. M.,Cruz, O. G.,Villela, D. A. M.,Bastos, L. S.,Pastore, Y. Piontti A.,Davis, J. T.,Vespignani, A.,Codeco, C. T.,Gomes, M. F. C.**. Assessing the spread of COVID-19 in Brazil: Mobility, morbidity and social vulnerability. *PLoS ONE [Electronic Resource].* 2020. 15:e0238214

**Cortellessa, G.,Stabile, L.,Arpino, F.,Faleiros, D. E.,van den Bos, W.,Morawska, L.,Buonanno, G.**. Close proximity risk assessment for SARS-CoV-2 infection. *Science of the Total Environment.* 2021. 794 (no pagination):#pages#

**Cotman, Z. J.,Bowden, M. J.,Richter, B. P.,Phelps, J. H.,Dibble, C. J.**. Factors affecting aerosol SARS-CoV-2 transmission via HVAC systems; a modeling study. *PLoS Computational Biology.* 2021. 17:e1009474

**Cuevas, E.**. An agent-based model to evaluate the COVID-19 transmission risks in facilities. *Computers in Biology & Medicine.* 2020. 121:103827

**Dacic, S. D.,Miljkovic, M. N.,Jovanovic, M. C.**. Dental care during the Covid-19 pandemic - To treat or not to treat?. *Journal of Infection in Developing Countries.* 2020. 14(10):1111-1116

**Dadlani, S.**. SARS-CoV-2 Transmission in a Dental Practice in Spain: After the Outbreak. *International Journal of Dentistry.* 2020. 2020 (no pagination):#pages#

**Del Brutto, O. H.,Costa, A. F.,Garcia, H. H.**. Images in clinical tropical medicine: Incident SARS-CoV-2 infection and a shared Latrine. *American Journal of Tropical Medicine and Hygiene.* 2020. 103(3):941-942

**Dhillon, P.,Breuer, M.,Hirst, N.**. COVID-19 breakthroughs: separating fact from fiction. *FEBS Journal.* 2020. 287(17):3612-3632

**Di Spirito, F., Iacono, V. J., Alfredo, I., Alessandra, A., Sbordone, L., Lanza, A.**. Evidence-based recommendations on periodontal practice and the management of periodontal patients during and after the covid-19 era: Challenging infectious diseases spread by airborne transmission. *Open Dentistry Journal.* 2021. 15(1):325-336

**Dubey, H.,Tiwari, A. K.,Singh, P.,Mehta, P.,Srivastava, S.,Mehdi, S. H.**. 2019-nCoV routes, current trends and future dental challenges in India. *International Journal of Research in Pharmaceutical Sciences.* 2020. 11(Special Issue 1):1230-1239

**Dzinamarira, T.,Mhango, M.,Dzobo, M.,Ngara, B.,Chitungo, I.,Makanda, P.,Atwine, J.,Nkambule, S. J.,Musuka, G.**. Risk factors for COVID-19 among healthcare workers. A protocol for a systematic review and meta-analysis. *PLoS ONE.* 2021. 16(5 May) (no pagination):#pages#

**Chen, Q.,Gao, Y.,Wang, C. S.,Kang, K.,Yu, H.,Zhao, M. Y.,Yu, K. J.**. Exploration of transmission chain and prevention of the recurrence of coronavirus disease 2019 in Heilongjiang Province due to in-hospital transmission. *World Journal of Clinical Cases.* 2021. 9:5420-5426

**Ehrhardt, J.,Ekinci, A.,Krehl, H.,Meincke, M.,Finci, I.,Klein, J.,Geisel, B.,Wagner-Wiening, C.,Eichner, M.,Brockmann, S. O.**. Transmission of SARS-CoV-2 in children aged 0 to 19 years in childcare facilities and schools after their reopening in May 2020, Baden-Wurttemberg, Germany. *Eurosurveillance.* 2020. 25(36) (no pagination):#pages#

**Elizondo, V.,Harkins, G. W.,Mabvakure, B.,Smidt, S.,Zappile, P.,Marier, C.,Maurano, M. T.,Perez, V.,Mazza, N.,Beloso, C.,Ifran, S.,Fernandez, M.,Santini, A.,Perez, V.,Estevez, V.,Nin, M.,Manrique, G.,Perez, L.,Ross, F.,Boschi, S.,Zubillaga, M. N.,Balleste, R.,Dellicour, S.,Heguy, A.,Duerr, R.**. SARS-CoV-2 genomic characterization and clinical manifestation of the COVID-19 outbreak in Uruguay. *Emerging Microbes & Infections.* 2021. 10:51-65

**Ghosh, P.,Mollah, M. M.**. The The risk of public mobility from hotspots of COVID-19 during travel restriction in Bangladesh. *Journal of Infection in Developing Countries.* 2020. 14:732-736

**Okyere, I.,Chuku, E. O.,Ekumah, B.,Angnuureng, D. B.,Boakye-Appiah, J. K.,Mills, D. J.,Babanawo, R.,Asare, N. K.,Aheto, D. W.,Crawford, B.**. Physical distancing and risk of COVID-19 in small-scale fisheries: a remote sensing assessment in coastal Ghana. *Scientific Reports.* 2020. 10:22407

**Oude Munnink, B. B.,Sikkema, R. S.,Nieuwenhuijse, D. F.,Molenaar, R. J.,Munger, E.,Molenkamp, R.,van der Spek, A.,Tolsma, P.,Rietveld, A.,Brouwer, M.,Bouwmeester-Vincken, N.,Harders, F.,Hakze-van der Honing, R.,Wegdam-Blans, M. C. A.,Bouwstra, R. J.,GeurtsvanKessel, C.,van der Eijk, A. A.,Velkers, F. C.,Smit, L. A. M.,Stegeman, A.,van der Poel, W. H. M.,Koopmans, M. P. G.**. Transmission of SARS-CoV-2 on mink farms between humans and mink and back to humans. *Science.* 2021. 371:172-177

**Park, A. W.**. Trip duration modifies spatial spread of infectious diseases. *Proceedings of the National Academy of Sciences of the United States of America.* 2020. 117(37):22637-22638

**Price, J. R.,Mookerjee, S.,Dyakova, E.,Myall, A.,Leung, W.,Weise, A. Y.,Shersing, Y.,Brannigan, E. T.,Galletly, T.,Muir, D.,Randell, P.,Davies, F.,Bolt, F.,Barahona, M.,Otter, J. A.,Holmes, A. H.**. Development and Delivery of a Real-time Hospital-onset COVID-19 Surveillance System Using Network Analysis. *Clinical Infectious Diseases.* 2021. 72:82-89

**Rego, N.,Salazar, C.,Paz, M.,Costabile, A.,Fajardo, A.,Ferres, I.,Perbolianachis, P.,Fernandez-Calero, T.,Noya, V.,Machado, M. R.,Brandes, M.,Arce, R.,Arleo, M.,Possi, T.,Reyes, N.,Bentancor, M. N.,Lizasoain, A.,Bortagaray, V.,Moller, A.,Chappos, O.,Nin, N.,Hurtado, J.,Duquia, M.,Gonzalez, M. B.,Griffero, L.,Mendez, M.,Techera, M. P.,Zanetti, J.,Pereira, E.,Rivera, B.,Maidana, M.,Alonso, M.,Smircich, P.,Arantes, I.,Mir, D.,Alonso, C.,Medina, J.,Albornoz, H.,Colina, R.,Bello, G.,Moreno, P.,Moratorio, G.,Iraola, G.,Spangenberg, L.**. Emergence and Spread of a B.1.1.28-Derived P.6 Lineage with Q675H and Q677H Spike Mutations in Uruguay. *Viruses.* 2021. 13:10

**Semakula, M.,Niragire, F.,Umutoni, A.,Nsanzimana, S.,Ndahindwa, V.,Rwagasore, E.,Nyatanyi, T.,Remera, E.,Faes, C.**. The secondary transmission pattern of COVID-19 based on contact tracing in Rwanda. *BMJ Global Health.* 2021. 6:06

**Sugg, M. M.,Spaulding, T. J.,Lane, S. J.,Runkle, J. D.,Harden, S. R.,Hege, A.,Iyer, L. S.**. Mapping community-level determinants of COVID-19 transmission in nursing homes: A multi-scale approach. *Science of the Total Environment.* 2021. 752:141946

**da Cruz, P. P. A.,Crema-Cruz, L. C.,Campos, F. S.**. Modeling transmission dynamics of severe acute respiratory syndrome coronavirus 2 in Sao Paulo, Brazil. *Revista da Sociedade Brasileira de Medicina Tropical.* 2021. 54:1-8

**Daon, Y.,Thompson, R. N.,Obolski, U.**. Estimating COVID-19 outbreak risk through air travel. *Journal of Travel Medicine.* 2020. 27:20

**Das, A.,Ghosh, S.,Das, K.,Basu, T.,Dutta, I.,Das, M.**. Living environment matters: Unravelling the spatial clustering of COVID-19 hotspots in Kolkata megacity, India. *Sustainable Cities and Society.* 2021. 65:102577

**Davis, J. T.,Chinazzi, M.,Perra, N.,Mu, K.,Piontti, A. P. Y.,Ajelli, M.,Dean, N. E.,Gioannini, C.,Litvinova, M.,Merler, S.,Rossi, L.,Sun, K.,Xiong, X.,Halloran, M. E.,Longini, I. M.,Viboud, C.,Vespignani, A.**. Cryptic transmission of SARS-CoV-2 and the first COVID-19 wave in Europe and the United States. *MedRxiv : the Preprint Server for Health Sciences.* 2021. 26:26

**De Salazar, P. M.,Niehus, R.,Taylor, A.,Buckee, C. O.,Lipsitch, M.**. Identifying Locations with Possible Undetected Imported Severe Acute Respiratory Syndrome Coronavirus 2 Cases by Using Importation Predictions. *Emerging Infectious Diseases.* 2020. 26:1465-1469

**Deforche, K.,Vercauteren, J.,Muller, V.,Vandamme, A. M.**. Behavioral changes before lockdown and decreased retail and recreation mobility during lockdown contributed most to controlling COVID-19 in Western countries. *BMC Public Health.* 2021. 21:654

**Deol, A. K.,Scarponi, D.,Beckwith, P.,Yates, T. A.,Karat, A. S.,Yan, A. W. C.,Baisley, K. S.,Grant, A. D.,White, R. G.,McCreesh, N.**. Estimating ventilation rates in rooms with varying occupancy levels: Relevance for reducing transmission risk of airborne pathogens. *PLoS ONE [Electronic Resource].* 2021. 16:e0253096

**Dietrich, W. L.,Bennett, J. S.,Jones, B. W.,Hosni, M. H.**. Laboratory Modeling of SARS-CoV-2 Exposure Reduction Through Physically Distanced Seating in Aircraft Cabins Using Bacteriophage Aerosol - November 2020. *MMWR - Morbidity & Mortality Weekly Report.* 2021. 70:595-599

**Dong, M.,Zhang, X.,Yang, K.,Liu, R.,Chen, P.**. Forecasting the COVID-19 transmission in Italy based on the minimum spanning tree of dynamic region network. *PeerJ.* 2021. 9 (no pagination):#pages#

**Donnat, C.,Bunbury, F.,Kreindler, J.,Liu, D.,Filippidis, F. T.,El-Osta, A.,Esko, T.,Harris, M.**. Predicting COVID-19 Transmission to Inform the Management of Mass Events: a model-based approach. *JMIR Public Health and Surveillance.* 2021. 18:18

**Dordevic, J.,Papic, I.,Suvak, N.**. A two diffusion stochastic model for the spread of the new corona virus SARS-CoV-2. *Chaos Solitons & Fractals.* 2021. 148:110991

**DuPre, N. C.,Karimi, S.,Zhang, C. H.,Blair, L.,Gupta, A.,Alharbi, L. M. A.,Alluhibi, M.,Mitra, R.,McKinney, W. P.,Little, B.**. County-level demographic, social, economic, and lifestyle correlates of COVID-19 infection and death trajectories during the first wave of the pandemic in the United States. *Science of the Total Environment.* 2021. 786 (no pagination):#pages#

**Dy, L. F.,Rabajante, J. F.**. A COVID-19 infection risk model for frontline health care workers. *Network Modeling Analysis in Health Informatics and Bioinformatics.* 2020. 9(1) (no pagination):#pages#

**da Silva Pedrosa, M.,Sipert, C. R.,Nogueira, F. N.**. Are the salivary glands the key players in spreading COVID-19 asymptomatic infection in dental practice?. *Journal of Medical Virology.* 2021. 93(1):204-205

**Daci, R.,Natarajan, S. K.,Johnson, M. D.**. Safety considerations for neurosurgical procedures during the COVID-19 pandemic. *Neurosurgery.* 2020. 87(2):E239-E240

**Danesh-Meyer, H. V.,McGhee, C. N. J.**. Implications of COVID-19 for Ophthalmologists. *American Journal of Ophthalmology.* 2021. 223:108-118

**Davoli, F.,Poletti, V.,Stella, F.**. Three-chamber chest drain system in the COVID-19 era: is there a risk of further transmission?. *Advances in Respiratory Medicine.* 2020. 88:366-368

**De Miguel Buckley, R.,Diaz-Menendez, M.**. Go to gate: COVID-19 imported cases in Madrid and the potential role of airport transmissions. *Transactions of the Royal Society of Tropical Medicine and Hygiene.* 2021. 115(7):731-732

**de Niet, A.,Waanders, B. L.,Walraven, I.**. The role of children in the transmission of mild SARS-CoV-2 infection. *Acta Paediatrica, International Journal of Paediatrics.* 2020. 109(8):1687

**Debenham, L.,Reynolds, J.**. Climbing Gyms as Possible High-Risk Transmission Locations in Microbial Outbreaks. *Wilderness & Environmental Medicine.* 2020. 31:375-376

**Dehghani, R.,Kassiri, H.**. A brief review on the possible role of houseflies and cockroaches in the mechanical transmission of coronavirus disease 2019 (Covid-19). *Archives of Clinical Infectious Diseases.* 2020. 15(COVID-19) (no pagination):#pages#

**Delamou, A.,Sidibe, S.,Camara, A.,Traore, M. S.,Toure, A.,Van Damme, W.**. Tackling the COVID-19 pandemic in West Africa: Have we learned from Ebola in Guinea?. *Preventive Medicine Reports.* 2020. 20 (no pagination):#pages#

**Dhand, R.,Li, J.**. Coughs and Sneezes: Their Role in Transmission of Respiratory Viral Infections, including SARS-CoV-2. *American Journal of Respiratory and Critical Care Medicine.* 2020. 202(5):651-659

**Di Gennaro, F.,Marotta, C.,Storto, M.,D'Avanzo, C.,Foschini, N.,Maffei, L.,de Gaetano, G.,Centonze, D.,Iezzi, E.**. SARS-CoV-2 transmission and outcome in neuro-rehabilitation patients hospitalized at neuroscience hospital in Italy. *Mediterranean Journal of Hematology and Infectious Diseases.* 2020. 12(1) (no pagination):#pages#

**Donaldson, A. I.**. Aerosols in meat plants as possible cause of Covid-19 spread. *Veterinary Record.* 2020. 187(1):34-35

**Donnelly, S. C.**. COVID-19-the risk of secondary transmission among households quantified. *Qjm.* 2020. 113:839

**dos Santos, R. P.,Ferreira, S. A. L.,da Fontoura Carvalho, O. L.,Dalmora, C. H.,Ruiz, R. D.,Menezes, A.,Vaz, T. A.**. COVID-19 transmission events in school staff in a Brazilian prospective-cohort. *Infection Control and Hospital Epidemiology..* 2020. #volume#:#pages#

**Downs, L. O.,Eyre, D. W.,O'Donnell, D.,Jeffery, K.**. Home-based SARS-CoV-2 lateral flow antigen testing in hospital workers. *Journal of Infection.* 2021. 82(2):282-327

**Deng, X.,Gu, W.,Federman, S.,du Plessis, L.,Pybus, O. G.,Faria, N. R.,Wang, C.,Yu, G.,Bushnell, B.,Pan, C. Y.,Guevara, H.,Sotomayor-Gonzalez, A.,Zorn, K.,Gopez, A.,Servellita, V.,Hsu, E.,Miller, S.,Bedford, T.,Greninger, A. L.,Roychoudhury, P.,Starita, L. M.,Famulare, M.,Chu, H. Y.,Shendure, J.,Jerome, K. R.,Anderson, C.,Gangavarapu, K.,Zeller, M.,Spencer, E.,Andersen, K. G.,MacCannell, D.,Paden, C. R.,Li, Y.,Zhang, J.,Tong, S.,Armstrong, G.,Morrow, S.,Willis, M.,Matyas, B. T.,Mase, S.,Kasirye, O.,Park, M.,Masinde, G.,Chan, C.,Yu, A. T.,Chai, S. J.,Villarino, E.,Bonin, B.,Wadford, D. A.,Chiu, C. Y.**. Genomic surveillance reveals multiple introductions of SARS-CoV-2 into Northern California. *Science.* 2020. 369(6503):582-587

**Jia, H. L.,Li, P.,Liu, H. J.,Zhong, J. Y.,Qin, P. Z.,Su, W. Z.,Zheng, Y. F.,Li, K. B.,Zeng, Q.,Li, J. H.,Li, L. Z.,Cao, L.,Wu, J. B.,Chen, Y. Y.,Jia, L. L.,Song, H. B.,Zhang, Q. W.,Yang, G.,Jing, C. X.,Bo, X. C.,Zhang, Z. B.,Di, B.,Xiao, C. L.,Ni, M.**. Genomic elucidation of a covid-19 resurgence and local transmission of sars-cov-2 in guangzhou, china. *Journal of Clinical Microbiology.* 2021. 59(8) (no pagination):#pages#

**Lemieux, J. E.,Siddle, K. J.,Shaw, B. M.,Loreth, C.,Schaffner, S. F.,Gladden-Young, A.,Adams, G.,Fink, T.,Tomkins-Tinch, C. H.,Krasilnikova, L. A.,DeRuff, K. C.,Rudy, M.,Bauer, M. R.,Lagerborg, K. A.,Normandin, E.,Chapman, S. B.,Reilly, S. K.,Anahtar, M. N.,Lin, A. E.,Carter, A.,Myhrvold, C.,Kemball, M. E.,Chaluvadi, S.,Cusick, C.,Flowers, K.,Neumann, A.,Cerrato, F.,Farhat, M.,Slater, D.,Harris, J. B.,Branda, J. A.,Hooper, D.,Gaeta, J. M.,Baggett, T. P.,O'Connell, J.,Gnirke, A.,Lieberman, T. D.,Philippakis, A.,Burns, M.,Brown, C. M.,Luban, J.,Ryan, E. T.,Turbett, S. E.,LaRocque, R. C.,Hanage, W. P.,Gallagher, G. R.,Madoff, L. C.,Smole, S.,Pierce, V. M.,Rosenberg, E.,Sabeti, P. C.,Park, D. J.,MacInnis, B. L.**. Phylogenetic analysis of SARS-CoV-2 in Boston highlights the impact of superspreading events. *Science.* 6529. 371:05

**Lemieux, J. E.,Siddle, K. J.,Shaw, B. M.,Loreth, C.,Schaffner, S. F.,Gladden-Young, A.,Adams, G.,Fink, T.,Tomkins-Tinch, C. H.,Krasilnikova, L. A.,DeRuff, K. C.,Rudy, M.,Bauer, M. R.,Lagerborg, K. A.,Normandin, E.,Chapman, S. B.,Reilly, S. K.,Anahtar, M. N.,Lin, A. E.,Carter, A.,Myhrvold, C.,Kemball, M. E.,Chaluvadi, S.,Cusick, C.,Flowers, K.,Neumann, A.,Cerrato, F.,Farhat, M.,Slater, D.,Harris, J. B.,Branda, J.,Hooper, D.,Gaeta, J. M.,Baggett, T. P.,O'Connell, J.,Gnirke, A.,Lieberman, T. D.,Philippakis, A.,Burns, M.,Brown, C. M.,Luban, J.,Ryan, E. T.,Turbett, S. E.,LaRocque, R. C.,Hanage, W. P.,Gallagher, G. R.,Madoff, L. C.,Smole, S.,Pierce, V. M.,Rosenberg, E.,Sabeti, P. C.,Park, D. J.,Maclnnis, B. L.**. Phylogenetic analysis of SARS-CoV-2 in the Boston area highlights the role of recurrent importation and superspreading events. *MedRxiv : the Preprint Server for Health Sciences.* 2020. 25:25

**Li, K. K.,Woo, Y. M.,Stirrup, O.,Hughes, J.,Ho, A.,Filipe, A. D. S.,Johnson, N.,Smollett, K.,Mair, D.,Carmichael, S.,Tong, L.,Nichols, J.,Aranday-Cortes, E.,Brunker, K.,Parr, Y. A.,Nomikou, K.,McDonald, S. E.,Niebel, M.,Asamaphan, P.,Sreenu, V. B.,Robertson, D. L.,Taggart, A.,Jesudason, N.,Shah, R.,Shepherd, J.,Singer, J.,Taylor, A. H. M.,Cousland, Z.,Price, J.,Lees, J. S.,Jones, T. P. W.,Lopez, C. V.,MacLean, A.,Starinskij, I.,Gunson, R.,Morris, S. T. W.,Thomson, P. C.,Geddes, C. C.,Traynor, J. P.,Breuer, J.,Thomson, E. C.,Mark, P. B.,Covid- Genomics UK consortium**. Genetic epidemiology of SARS-CoV-2 transmission in renal dialysis units - A high risk community-hospital interface. *Journal of Infection.* 2021. 83:96-103

**Murall, C. L., Fournier, E., Galvez, J. H., N'Guessan, A., Reiling, S. J., Quirion, P. O., Naderi, S., Roy, A. M., Chen, S. H., Stretenowich, P., Bourgey, M., Bujold, D., Gregoire, R., Lepage, P., St-Cyr, J., Willet, P., Dion, R., Charest, H., Lathrop, M., Roger, M., Bourque, G., Ragoussis, J., Shapiro, B. J., Moreira, S.**. A small number of early introductions seeded widespread transmission of SARS-CoV-2 in Quebec, Canada. *Genome Medicine.* 2021. 13:169

**Siddle, K. J., Krasilnikova, L. A., Moreno, G. K., Schaffner, S. F., Vostok, J., Fitzgerald, N. A., Lemieux, J. E., Barkas, N., Loreth, C., Specht, I., Tomkins-Tinch, C. H., Silbert, J., Schaeffer, B., Taylor, B. P., Loftness, B., Johnson, H., Schubert, P. L., Shephard, H. M., Doucette, M., Fink, T., Lang, A. S., Baez, S., Beauchamp, J., Hennigan, S., Buzby, E., Ash, S., Brown, J., Clancy, S., Cofsky, S., Gagne, L., Hall, J., Harrington, R., Gionet, G. L., DeRuff, K. C., Vodzak, M. E., Adams, G. C., Dobbins, S. T., Slack, S. D., Reilly, S. K., Anderson, L. M., Cipicchio, M. C., DeFelice, M. T., Grimsby, J. L., Anderson, S. E., Blumenstiel, B. S., Meldrim, J. C., Rooke, H. M., Vicente, G., Smith, N. L., Messer, K. S., Reagan, F. L., Mandese, Z. M., Lee, M. D., Ray, M. C., Fisher, M. E., Ulcena, M. A., Nolet, C. M., English, S. E., Larkin, K. L., Vernest, K., Chaluvadi, S., Arvidson, D., Melchiono, M., Covell, T., Harik, V., Brock-Fisher, T., Dunn, M., Kearns, A., Hanage, W. P., Bernard, C., Philippakis, A., Lennon, N. J., Gabriel, S. B., Gallagher, G. R., Smole, S., Madoff, L. C., Brown, C. M., Park, D. J., MacInnis, B. L., Sabeti, P. C.**. Evidence of transmission from fully vaccinated individuals in a large outbreak of the SARS-CoV-2 Delta variant in Provincetown, Massachusetts. *MedRxiv : the Preprint Server for Health Sciences.* 2021. 20:20

**Yang, X.,Dong, N.,Chan, E. W.,Chen, S.**. Genetic cluster analysis of SARS-CoV-2 and the identification of those responsible for the major outbreaks in various countries. *Emerging Microbes & Infections.* 2020. 9:1287-1299

**Ebrahim, S. H.,Memish, Z. A.**. COVID-19: preparing for superspreader potential among Umrah pilgrims to Saudi Arabia. *Lancet.* 2020. 395:e48

**Echternach, M.,Gantner, S.,Peters, G.,Westphalen, C.,Benthaus, T.,Jakubass, B.,Kuranova, L.,Dollinger, M.,Kniesburges, S.**. Impulse dispersion of aerosols during singing and speaking: A potential COVID-19 transmission pathway. *American Journal of Respiratory and Critical Care Medicine.* 2020. 202(11):1584-1587

**Eichelberger, L.,Dev, S.,Howe, T.,Barnes, D. L.,Bortz, E.,Briggs, B. R.,Cochran, P.,Dotson, A. D.,Drown, D. M.,Hahn, M. B.,Mattos, K.,Aggarwal, S.**. Implications of inadequate water and sanitation infrastructure for community spread of COVID-19 in remote Alaskan communities. *Science of the Total Environment.* 2021. 776 (no pagination):#pages#

**Eldin, C.,Lagier, J. C.,Mailhe, M.,Gautret, P.**. Probable aircraft transmission of Covid-19 in-flight from the Central African Republic to France. *Travel Medicine and Infectious Disease.* 2020. 35 (no pagination):#pages#

**Elliott, J. A.,Kenyon, R.,Kelliher, G.,Gillis, A. E.,Tierney, S.,Ridgway, P. F.**. Nosocomial SARS-CoV-2 transmission in postoperative infection and mortality: analysis of 14 798 procedures. *British Journal of Surgery.* 2020. 107(13):1708-1712

**Emami, N.,Tanner, T.,Ogundipe, F.,Hawn, V. S.,Rubin, R.,Skae, C. C.,Shiloh, A. L.,Keene, A. B.**. Drape to prevent disease transmission during endotracheal intubation. *American Journal of Infection Control.* 2021. 49:387-388

**Essa, M. F.,Elbashir, E.,Batarfi, K.,Alharbi, M.**. Lack of transmission of SARS-CoV-2 by platelet transfusion from a COVID-19-positive donor in a hematopoietic stem cell transplantation patient. *Pediatric Blood and Cancer.* 2021. 68(2) (no pagination):#pages#

**Ebrahim, S. H.,Memish, Z. A.**. COVID-19 - the role of mass gatherings. *Travel Medicine and Infectious Disease.* 2020. 34 (no pagination):#pages#

**Emrani, J., Ahmed, M., Jeffers-Francis, L., Teleha, J. C., Mowa, N., Newman, R. H., Thomas, M. D.**. SARS-COV-2, infection, transmission, transcription, translation, proteins, and treatment: A review. *International Journal of Biological Macromolecules..* 2021. #volume#:#pages#

**Edwards, N. J.,Widrick, R.,Wilmes, J.,Breisch, B.,Gerschefske, M.,Sullivan, J.,Potember, R.,Espinoza-Calvio, A.**. Reducing COVID-19 airborne transmission risks on public transportation buses: an empirical study on aerosol dispersion and control. *Aerosol Science and Technology..* 2021. #volume#:#pages#

**Eiche, T.,Kuster, M.**. Aerosol release by healthy people during speaking: Possible contribution to the transmission of sars-cov-2. *International Journal of Environmental Research and Public Health.* 2020. 17(23):1-14

**Eilersen, A.,Sneppen, K.**. SARS-CoV-2 superspreading in cities vs the countryside. *APMIS.* 2021. 129:401-407

**Ekumah, B.,Armah, F. A.,Yawson, D. O.,Quansah, R.,Nyieku, F. E.,Owusu, S. A.,Odoi, J. O.,Afitiri, A. R.**. Disparate on-site access to water, sanitation, and food storage heighten the risk of COVID-19 spread in Sub-Saharan Africa. *Environmental Research.* 2020. 189 (no pagination):#pages#

**Emery, J. C.,Russell, T. W.,Liu, Y.,Hellewell, J.,Pearson, C. A. B.,Knight, G. M.,Eggo, R. M.,Kucharski, A. J.,Funk, S.,Flasche, S.,Houben, R. M. G. J.,Atkins, K. E.,Klepac, P.,Endo, A.,Jarvis, C. I.,Davies, N. G.,Rees, E. M.,Meakin, S. R.,Rosello, A.,van Zandvoort, K.,Munday, J. D.,Edmunds, W. J.,Jombart, T.,Auzenbergs, M.,Nightingale, E. S.,Jit, M.,Abbott, S.,Simons, D.,Bosse, N. I.,Leclerc, Q. J.,Procter, S. R.,Villabona-Arenas, C. J.,Tully, D. C.,Deol, A. K.,Sun, F. Y.,Hue, S.,Foss, A. M.,Prem, K.,Medley, G.,Gimma, A.,Lowe, R.,Clifford, S.,Quaife, M.,Diamond, C.,Gibbs, H. P.,Quilty, B. J.,O'Reilly, K.**. The contribution of asymptomatic sars-cov-2 infections to transmission on the diamond princess cruise ship. *eLife.* 2020. 9:1-68

**Enright, J.,Hill, E. M.,Stage, H. B.,Bolton, K. J.,Nixon, E. J.,Fairbanks, E. L.,Tang, M. L.,Brooks-Pollock, E.,Dyson, L.,Budd, C. J.,Hoyle, R. B.,Schewe, L.,Gog, J. R.,Tildesley, M. J.**. SARS-CoV-2 infection in UK university students: lessons from September-December 2020 and modelling insights for future student return. *Royal Society Open Science.* 2021. 8:210310

**Evans, S., Stimson, J., Pople, D., Bhattacharya, A., Hope, R., White, P. J., Robotham, J. V.**. Quantifying the contribution of pathways of nosocomial acquisition of COVID-19 in English hospitals. *International Journal of Epidemiology.* 2021. 04:04

**Fallon, A.,Dukelow, T.,Kennelly, S. P.,O'Neill, D.**. COVID-19 in nursing homes. *Qjm.* 2020. 113:391-392

**Fantini, M. P.,Reno, C.,Biserni, G. B.,Savoia, E.,Lanari, M.**. COVID-19 and the re-opening of schools: A policy maker's dilemma. *Italian Journal of Pediatrics.* 2020. 46(1) (no pagination):#pages#

**Flasche, S.,Edmunds, W. J.**. The role of schools and school-aged children in SARS-CoV-2 transmission. *The Lancet Infectious Diseases.* 2021. 21(3):298-299

**Fong, M. W.,Cowling, B. J.,Leung, G. M.,Wu, P.**. Letter to the editor: COVID-19 cases among school-aged children and school-based measures in Hong Kong, July 2020. *Eurosurveillance.* 2020. 25:#pages#

**Furuse, Y.,Tsuchiya, N.,Miyahara, R.,Yasuda, I.,Sando, E.,Ko, Y. K.,Imamura, T.,Morimoto, K.,Imamura, T.,Shobugawa, Y.,Nagata, S.,Tokumoto, A.,Jindai, K.,Suzuki, M.,Oshitani, H.**. COVID-19 case-clusters and transmission chains in the communities in Japan. *Journal of Infection.* 2021. 11:11

**Fan, C.,Cai, T.,Gai, Z.,Wu, Y.**. The Relationship between the Migrant Population's Migration Network and the Risk of COVID-19 Transmission in China-Empirical Analysis and Prediction in Prefecture-Level Cities. *International Journal of Environmental Research & Public Health [Electronic Resource].* 2020. 17:11

**Farthing, T. S.,Lanzas, C.**. Assessing the efficacy of interventions to control indoor SARS-Cov-2 transmission: an agent-based modeling approach. *MedRxiv : the Preprint Server for Health Sciences.* 2021. 22:22

**Fasona, M. J.,Okolie, C. J.,Otitoloju, A. A.**. Spatial drivers of COVID-19 vulnerability in Nigeria. *The Pan African medical journal.* 2021. 39:19

**Feng, Y.,Li, Q.,Tong, X.,Wang, R.,Zhai, S.,Gao, C.,Lei, Z.,Chen, S.,Zhou, Y.,Wang, J.,Yan, X.,Xie, H.,Chen, P.,Xv, X.,Liu, S.,Jin, Y.,Wang, C.,Hong, Z.,Luan, K.,Wei, C.,Xu, J.,Jiang, H.,Xiao, C.,Guo, Y.**. Spatiotemporal spread pattern of the COVID-19 cases in China. *PLoS ONE.* 2020. 15(12 December) (no pagination):#pages#

**Feng, Y.,Marchal, T.,Sperry, T.,Yi, H.**. Influence of wind and relative humidity on the social distancing effectiveness to prevent COVID-19 airborne transmission: A numerical study. *Journal of Aerosol Science.* 2020. 147 (no pagination):#pages#

**Fierce, L.,Robey, A. J.,Hamilton, C.**. Simulating near-field enhancement in transmission of airborne viruses with a quadrature-based model. *Indoor air..* 2021. 23:#pages#

**Fontes, D.,Reyes, J.,Ahmed, K.,Kinzel, M.**. A study of fluid dynamics and human physiology factors driving droplet dispersion from a human sneeze. *Physics of Fluids.* 2020. 32:111904

**Foster, A., Kinzel, M.**. SARS-CoV-2 transmission in classroom settings: Effects of mitigation, age, and Delta variant. *Physics of Fluids.* 2021. 33:113311

**Foster, A.,Kinzel, M.**. Estimating COVID-19 exposure in a classroom setting: A comparison between mathematical and numerical models. *Physics of Fluids.* 2021. 33:021904

**Fan, C.,Lee, S.,Yang, Y.,Oztekin, B.,Li, Q.,Mostafavi, A.**. Effects of population co-location reduction on cross-county transmission risk of COVID-19 in the United States. *Applied Network Science.* 2021. 6:14

**Figueiredo Filho, D. B.,Silva, L. E. O.**. Social distancing and severe acute respiratory syndrome coronavirus 2 transmission: A case study from araraquara, Sao Paulo, Brazil. *Revista da Sociedade Brasileira de Medicina Tropical.* 2021. 54 (no pagination):#pages#

**Fitriangga, A.,Rahardjo, W.,Alex,,Pramulya, M.**. The distribution of COVID-19 cases using spatial analysis to support surveillance program in Pontianak City. *Teikyo Medical Journal.* 2021. 44(1):585-597

**Foster, P.,Cheung, T.,Craft, P.,Baran, K.,Kryskow, M.,Knowles, R.,Toia, A.,Galvez, C.,Bowling, A.,DiSiena, M.**. Novel Approach to Reduce Transmission of COVID-19 During Tracheostomy. *Journal of the American College of Surgeons.* 2020. 230(6):1102-1104

**Fouda, B.,Tram, H. P. B.,Makram, O. M.,Abdalla, A. S.,Singh, T.,Hung, I. C.,Hemmeda, L.,Alahmar, M.,Raut, A.,ElHawary, A. S.,Awad, D.,Huy, N. T.**. Identifying SARS-CoV2 transmission cluster category: An analysis of country government database. *BMC Proceedings. Conference: 4th Annual Student Medical Summit. Virtual..* 2021. 15:#pages#

**Fouda, B.,Tram, H. P. B.,Makram, O. M.,Abdalla, A. S.,Singh, T.,Hung, I. C.,Raut, A.,Hemmeda, L.,Alahmar, M.,ElHawary, A. S.,Awad, D. M.,Huy, N. T.**. Identifying SARS-CoV2 transmission cluster category: An analysis of country government database. *Journal of Infection and Public Health.* 2021. 14:461-467

**Fung, H. F.,Martinez, L.,Alarid-Escudero, F.,Salomon, J. A.,Studdert, D. M.,Andrews, J. R.,Goldhaber-Fiebert, J. D.,Stanford, Cide Coronavirus Simulation Model Modeling Group**. The Household Secondary Attack Rate of Severe Acute Respiratory Syndrome Coronavirus 2 (SARS-CoV-2): A Rapid Review. *Clinical Infectious Diseases.* 2021. 73:S138-S145

**Galow, L., Haag, L., Kahre, E., Blankenburg, J., Dalpke, A. H., Luck, C., Berner, R., Armann, J. P.**. Lower household transmission rates of SARS-CoV-2 from children compared to adults. *Journal of Infection.* 2021. 83(1):e34-e36

**Gandolfi, M. G.,Zamparini, F.,Spinelli, A.,Sambri, V.,Prati, C.**. Risks of aerosol contamination in dental procedures during the second wave of COVID-19-experience and proposals of innovative IPC in dental practice. *International Journal of Environmental Research and Public Health.* 2020. 17(23):1-12

**Ganesan, M.,Renganathan, J.,Vasuki, V.,Joseph, R.**. S gene drop-out predicts super spreader H69del/V70del mutated SARS-CoV-2 virus. *Asian Pacific Journal of Tropical Medicine.* 2021. 14(5):236-237

**Ge, R.,Tian, M.,Gu, Q.,Chen, P.,Shen, Y.,Qi, Y.,Yan, Y.,Chen, Z.**. The role of close contacts tracking management in COVID-19 prevention: A cluster investigation in Jiaxing, China. *Journal of Infection.* 2020. 81(1):e71-e74

**George, R.,George, A.**. COVID-19 in South Africa: An occupational disease. *South African Medical Journal.* 2020. 110(8):705

**Georgiou, G. P.,Kilani, A.**. The use of aspirated consonants during speech may increase the transmission of COVID-19. *Medical Hypotheses.* 2020. 144 (no pagination):#pages#

**Gerami, P.,Liszewski, W.**. Risk assessment of outpatient dermatology practice in the setting of the COVID-19 pandemic. *Journal of the American Academy of Dermatology.* 2020. 83:1538-1539

**Goh, Y.,Chua, W.,Lee, J. K. T.,Ang, B. W. L.,Liang, C. R.,Tan, C. A.,Choong, D. A. W.,Hoon, H. X.,Ong, M. K. L.,Quek, S. T.**. Operational Strategies to Prevent Coronavirus Disease 2019 (COVID-19) Spread in Radiology: Experience From a Singapore Radiology Department After Severe Acute Respiratory Syndrome. *Journal of the American College of Radiology.* 2020. 17:717-723

**Granados, J.,Ceballos, M.,Amariles, P.**. Proposed Individual Risk Score for Educational Institutions: The Role of Health Surveillance for the SARS-CoV-2 Risk Assessment in the Schools. *Journal of Occupational & Environmental Medicine.* 2021. 63:e257-e258

**Gu, H., Krishnan, P., Ng, D. Y. M., Chang, L. D. J., Liu, G. Y. Z., Cheng, S. S. M., Hui, M. M. Y., Fan, M. C. Y., Wan, J. H. L., Lau, L. H. K., Cowling, B. J., Peiris, M., Poon, L. L. M.**. Probable Transmission of SARS-CoV-2 Omicron Variant in Quarantine Hotel, Hong Kong, China, November 2021. *Emerging infectious diseases.* 2021. 28:#pages#

**Gu, Y.,Lu, J.,Su, W.,Liu, Y.,Xie, C.,Yuan, J.**. Transmission of SARS-CoV-2 in the karaoke room: An outbreak of COVID-19 in Guangzhou, China, 2020. *Journal of Epidemiology and Global Health.* 2021. 11(1):6-9

**Gu, Y.,Lu, J.,Yuan, J.**. The necessity of detailed epidemiological investigation in the early stage of an outbreak: lessons from a six-case cluster of COVID-19 in Guangzhou, China. *Australian & New Zealand Journal of Public Health.* 2020. 44:428-429

**Guilamo-Ramos, V., Benzekri, A., Thimm-Kaiser, M., Hidalgo, A., Perlman, D. C.**. Reconsidering assumptions of adolescent and young adult severe acute respiratory syndrome coronavirus 2 transmission dynamics. *Clinical Infectious Diseases.* 2021. 73:S146-S163

**Gunn, R. A.,Bellettiere, J.,Garfein, R. S.,Long, K. C.,Binkin, N. J.,Anderson, C. A. M.**. Identifying COVID-19 Cases and Social Groups at High Risk of Transmission: A Strategy to Reduce Community Spread. *Public Health Reports.* 2021. 136:259-263

**Gunthe, S. S.,Patra, S. S.**. Impact of international travel dynamics on domestic spread of 2019-nCoV in India: Origin-based risk assessment in importation of infected travelers. *Globalization and Health.* 2020. 16(1) (no pagination):#pages#

**Guo, W.,Chan, B. H.,Chng, C. K.,Shi, A. H.**. Two Cases of Inadvertent Dental Aerosol Exposure to COVID-19 Patients. *Annals of the Academy of Medicine, Singapore.* 2020. 49:514-516

**Gabor, C. R.,Clark, C.,Papadakis, G.,Clark, K.,Olsburgh, J.,Barnett, N.**. Covid-19 infection rates/outcomes in clinically extremely vulnerable renal replacement therapy patients admitted for procedures during the pandemic. *Transplant International.* 2021. 34(SUPPL 1):266-267

**Gambaro, F.,Behillil, S.,Baidaliuk, A.,Donati, F.,Albert, M.,Alexandru, A.,Vanpeene, M.,Bizard, M.,Brisebarre, A.,Barbet, M.,Derrar, F.,van der Werf, S.,Enouf, V.,Simon-Loriere, E.**. Introductions and early spread of SARS-CoV-2 in France, 24 January to 23 March 2020. *Euro Surveillance: Bulletin Europeen sur les Maladies Transmissibles = European Communicable Disease Bulletin.* 2020. 25:07

**Ge, Z. Y.,Yang, L. M.,Xia, J. J.,Fu, X. H.,Zhang, Y. Z.**. Possible aerosol transmission of COVID-19 and special precautions in dentistry. *Journal of Zhejiang University SCIENCE B.* 2020. 21:361-368

**Goldstein, E.,Lipsitch, M.,Cevik, M.**. On the effect of age on the transmission of SARS-CoV-2 in households, schools, and the community. *Journal of Infectious Diseases.* 2021. 223(3):362-369

**Gonzalez-Candelas, F.,Shaw, M. A.,Phan, T.,Kulkarni-Kale, U.,Paraskevis, D.,Luciani, F.,Kimura, H.,Sironi, M.**. One year into the pandemic: Short-term evolution of SARS-CoV-2 and emergence of new lineages. *Infection, Genetics and Evolution.* 2021. 92 (no pagination):#pages#

**Goodwin, L.,Hayward, T.,Krishan, P.,Nolan, G.,Nundy, M.,Ostrishko, K.,Attili, A.,Carceles, S. B.,Epelle, E. I.,Gabl, R.,Pappa, E. J.,Stajuda, M.,Zen, S.,Dozier, M.,Anderson, N.,Viola, I. M.,McQuillan, R.**. Which factors influence the extent of indoor transmission of SARS-CoV-2? A rapid evidence review. *Journal of Global Health.* 2021. 11:10002

**Gutsell, J.,Yang, Y.,Jeffrey, M.,Conway-Morris, A.,Mahroof, R.,Martin, J.**. Reducing breathing system transmission of Covid-19: The Addenbrooke's experience. *Intensive Care Medicine Experimental. Conference: 33rd European Society of Intensive Care Medicine Annual Congress, ESICM.* 2020. 8:#pages#

**Ganyani, T.,Kremer, C.,Chen, D.,Torneri, A.,Faes, C.,Wallinga, J.,Hens, N.**. Estimating the generation interval for coronavirus disease (COVID-19) based on symptom onset data, March 2020. *Euro Surveillance: Bulletin Europeen sur les Maladies Transmissibles = European Communicable Disease Bulletin.* 2020. 25:04

**Garcia, W.,Mendez, S.,Fray, B.,Nicolas, A.**. Model-based assessment of the risks of viral transmission in non-confined crowds. *Safety Science.* 2021. 144:105453

**Gardner, B. J.,Marm Kilpatrick, A.**. Contact tracing efficiency, transmission heterogeneity, and accelerating COVID-19 epidemics. *PLoS Computational Biology.* 2021. 17(6) (no pagination):#pages#

**Giardina, J., Bilinski, A., Fitzpatrick, M. C., Kendall, E. A., Linas, B. P., Salomon, J., Ciaranello, A. L.**. Model-estimated relationship between elementary school-related SARS-CoV-2 transmission, mitigation interventions, and vaccination coverage across community incidence levels. *MedRxiv : the Preprint Server for Health Sciences.* 2021. 16:16

**Godin, A.,Xia, Y.,Buckeridge, D. L.,Mishra, S.,Douwes-Schultz, D.,Shen, Y.,Lavigne, M.,Drolet, M.,Schmidt, A. M.,Brisson, M.,Maheu-Giroux, M.**. The role of case importation in explaining differences in early SARS-CoV-2 transmission dynamics in Canada-A mathematical modeling study of surveillance data. *International Journal of Infectious Diseases.* 2021. 102:254-259

**Gomes, D. S.,Andrade, L. A.,Ribeiro, C. J. N.,Peixoto, M. V. S.,Lima, Svma,Duque, A. M.,Cirilo, T. M.,Goes, M. A. O.,Lima, Agcf,Santos, M. B.,Araujo, Kcgm,Santos, A. D.**. Risk clusters of COVID-19 transmission in northeastern Brazil: prospective space-time modelling. *Epidemiology & Infection.* 2020. 148:e188

**Goyal, A.,Reeves, D. B.,Cardozo-Ojeda, E. F.,Schiffer, J. T.,Mayer, B. T.**. Wrong person, place and time: viral load and contact network structure predict SARS-CoV-2 transmission and super-spreading events. *MedRxiv : the Preprint Server for Health Sciences.* 2020. 28:28

**Goyal, A.,Reeves, D. B.,Cardozo-Ojeda, E. F.,Schiffer, J. T.,Mayer, B. T.**. Viral load and contact heterogeneity predict SARS-CoV-2 transmission and super-spreading events. *eLife.* 2021. 10:23

**Guagliardo, S. A. J.,Prasad, P. V.,Rodriguez, A.,Fukunaga, R.,Novak, R. T.,Ahart, L.,Reynolds, J.,Griffin, I.,Wiegand, R.,Quilter, L. A. S.,Morrison, S.,Jenkins, K.,Wall, H. K.,Treffiletti, A.,White, S. B.,Regan, J.,Tardivel, K.,Freeland, A.,Brown, C.,Wolford, H.,Johansson, M. A.,Cetron, M. S.,Slayton, R. B.,Friedman, C. R.**. Cruise ship travel in the era of COVID-19: A summary of outbreaks and a model of public health interventions. *Clinical Infectious Diseases.* 2021. 12:12

**Gupta, M.,Mohanta, S. S.,Rao, A.,Parameswaran, G. G.,Agarwal, M.,Arora, M.,Mazumder, A.,Lohiya, A.,Behera, P.,Bansal, A.,Kumar, R.,Meena, V. P.,Tiwari, P.,Mohan, A.,Bhatnagar, S.**. Transmission dynamics of the COVID-19 epidemic in India and modeling optimal lockdown exit strategies. *International Journal of Infectious Diseases.* 2021. 103:579-589

**Gursakal, N.,Batmaz, B.,Aktuna, G.**. Drawing transmission graphs for COVID-19 in the perspective of network science. *Epidemiology & Infection.* 2020. 148:e269

**Han, Q.,Lin, Q.,Ni, Z.,You, L.**. Uncertainties about the transmission routes of 2019 novel coronavirus. *Influenza and other Respiratory Viruses.* 2020. 14(4):470-471

**Hara, T.,Yamamoto, C.,Sawada, R.,Ohara, T.,Oka, K.,Iwai, N.,Inada, Y.,Tsuji, T.,Okuda, T.,Komaki, T.,Kagawa, K.**. Infection risk in a gastroenterological ward during a nosocomial COVID-19 infection event. *Journal of Medical Virology.* 2021. 93(1):30-31

**Harries, A. D.,Martinez, L.,Chakaya, J. M.**. SARS-CoV-2: how safe is it to fly and what can be done to enhance protection?. *Transactions of the Royal Society of Tropical Medicine & Hygiene.* 2021. 115:117-119

**Harris, R. J.,Hall, J. A.,Zaidi, A.,Andrews, N. J.,Dunbar, J. K.,Dabrera, G.**. Effect of vaccination on household transmission of sars-cov-2 in england. *New England Journal of Medicine.* 2021. 385(8):759-760

**Hb, V. K.,Manuel, E.**. Risk factors and transmission dynamics of COVID-19 among resident doctors-a retrospective analysis in a non-COVID tertiary care centre. *Antimicrobial Resistance and Infection Control. Conference: 6th International Conference on Prevention and Infection Control, ICPIC.* 2021. 10:#pages#

**Henry, B. F.**. Reducing COVID-19 outbreaks in prisons through public health-centred policies. *The Lancet Public Health.* 2021. 6(10):e701-e702

**Heudorf, U.,Gottschalk, R.**. The risk of asymptomatic and symptomatic covid-19 infec tion among schoolteachers and day-care workers compared to hospital and nursing-home staff. *Deutsches Arzteblatt International.* 2021. 118(12):213-214

**Hibino, M.,Iwabuchi, S.,Munakata, H.**. SARS-CoV-2 IgG seroprevalence among medical staff in a general hospital that treated patients with COVID-19 in Japan: retrospective evaluation of nosocomial infection control. *Journal of Hospital Infection.* 2021. 107:103-104

**Hindson, J.**. COVID-19: faecal-oral transmission?. *Nature Reviews Gastroenterology and Hepatology.* 2020. 17(5):259

**Hirota, K.**. Air contamination with SARS-CoV-2 in the operating room. *Journal of Anesthesia.* 2021. 35:333-336

**Honein, M. A.,Barrios, L. C.,Brooks, J. T.**. Data and Policy to Guide Opening Schools Safely to Limit the Spread of SARS-CoV-2 Infection. *JAMA - Journal of the American Medical Association.* 2021. 325(9):823-824

**Hosseininasab, A.,Khalooei, A.**. SARS-COV-2 infection in children in close contact with households with COVID-19. *Antimicrobial Resistance and Infection Control. Conference: 6th International Conference on Prevention and Infection Control, ICPIC.* 2021. 10:#pages#

**Humphreys, H.,Fitzpatrick, F.**. Airborne transmission of covid-19: Implications for irish hospitals. *Irish Medical Journal.* 2020. 113(7):1-3

**Ikizler, T. A.,Kliger, A. S.**. Minimizing the risk of COVID-19 among patients on dialysis. *Nature Reviews Nephrology.* 2020. 16:311-313

**Haddad, N.,Clapham, H. E.,Abou Naja, H.,Saleh, M.,Farah, Z.,Ghosn, N.,Mrad, P.,Howard, N.**. Calculating the serial interval of SARS-CoV-2 in Lebanon using 2020 contact-tracing data. *BMC Infectious Diseases.* 2021. 21:1053

**Haider, N.,Yavlinsky, A.,Simons, D.,Osman, A. Y.,Ntoumi, F.,Zumla, A.,Kock, R.**. Passengers' destinations from China: low risk of Novel Coronavirus (2019-nCoV) transmission into Africa and South America. *Epidemiology & Infection.* 2020. 148:e41

**Hancean, M. G.,Lerner, J.,Perc, M.,Ghita, M. C.,Bunaciu, D. A.,Stoica, A. A.,Mihaila, B. E.**. The role of age in the spreading of COVID-19 across a social network in Bucharest. *Journal of Complex Networks.* 2021. 9:cnab026

**Harmon, M.,Lau, J.**. The Facility Infection Risk EstimatorTM: A web application tool for comparing indoor risk mitigation strategies by estimating airborne transmission risk. *Indoor and Built Environment..* 2021. #volume#:#pages#

**Harrichandra, A.,Ierardi, A. M.,Pavilonis, B.**. An estimation of airborne SARS-CoV-2 infection transmission risk in New York City nail salons. *Toxicology & Industrial Health.* 2020. 36:634-643

**Hassan, A. M.,Megahed, N. A.**. COVID-19 and urban spaces: A new integrated CFD approach for public health opportunities. *Building & Environment.* 2021. 204:108131

**He, R.,Liu, W.,Elson, J.,Vogt, R.,Maranville, C.,Hong, J.**. Airborne transmission of COVID-19 and mitigation using box fan air cleaners in a poorly ventilated classroom. *Physics of Fluids.* 2021. 33:057107

**Head, J. R.,Andrejko, K. L.,Cheng, Q.,Collender, P. A.,Phillips, S.,Boser, A.,Heaney, A. K.,Hoover, C. M.,Wu, S. L.,Northrup, G. R.,Click, K.,Bardach, N. S.,Lewnard, J. A.,Remais, J. V.**. School closures reduced social mixing of children during COVID-19 with implications for transmission risk and school reopening policies. *Journal of the Royal Society Interface.* 2021. 18:20200970

**Hetherington, R., Toufique Hasan, A. B. M., Khan, A., Roy, D., Salehin, M., Wadud, Z.**. Exposure risk analysis of COVID-19 for a ride-sharing motorbike taxi. *Physics of Fluids.* 2021. 33:113319

**Hill, E. M., Atkins, B. D., Keeling, M. J., Tildesley, M. J., Dyson, L.**. Modelling SARS-CoV-2 transmission in a UK university setting. *Epidemics.* 2021. 36 (no pagination):#pages#

**Hill, E. M.,Atkins, B. D.,Keeling, M. J.,Dyson, L.,Tildesley, M. J.**. A network modelling approach to assess non-pharmaceutical disease controls in a worker population: An application to SARS-CoV-2. *PLoS Computational Biology.* 2021. 17:e1009058

**Hilton, J.,Keeling, M. J.**. Estimation of country-level basic reproductive ratios for novel Coronavirus (SARS-CoV-2/COVID-19) using synthetic contact matrices. *PLoS Computational Biology.* 2020. 16:e1008031

**Ho, C. K.**. Modeling airborne pathogen transport and transmission risks of SARS-CoV-2. *Applied Mathematical Modelling.* 2021. 95:297-319

**Holmdahl, I.,Kahn, R.,Hay, J. A.,Buckee, C. O.,Mina, M. J.**. Estimation of Transmission of COVID-19 in Simulated Nursing Homes With Frequent Testing and Immunity-Based Staffing. *JAMA Network Open.* 2021. 4:e2110071

**Hong, K., Yum, S., Kim, J., Yoo, D., Chun, B. C.**. Epidemiology and Regional Predictors of COVID-19 Clusters: A Bayesian Spatial Analysis Through a Nationwide Contact Tracing Data. *Frontiers in Medicine.* 2021. 8:753428

**Hong, K.,Yum, S. J.,Kim, J. H.,Chun, B. C.**. Re-estimation of basic reproduction number of COVID-19 based on the epidemic curve by symptom onset date. *Epidemiology & Infection.* 2021. 149:e53

**Hu, M.,Lin, H.,Wang, J.,Xu, C.,Tatem, A. J.,Meng, B.,Zhang, X.,Liu, Y.,Wang, P.,Wu, G.,Xie, H.,Lai, S.**. Risk of Coronavirus Disease 2019 Transmission in Train Passengers: an Epidemiological and Modeling Study. *Clinical Infectious Diseases.* 2021. 72:604-610

**Huang, J.,Kwan, M. P.,Kan, Z.**. The superspreading places of COVID-19 and the associated built-environment and socio-demographic features: A study using a spatial network framework and individual-level activity data. *Health & Place.* 2021. 72:102694

**Huang, L. S.,Li, L.,Dunn, L.,He, M.**. Taking account of asymptomatic infections: A modeling study of the COVID-19 outbreak on the Diamond Princess cruise ship. *PLoS ONE [Electronic Resource].* 2021. 16:e0248273

**Hussein, T.,Londahl, J.,Thuresson, S.,Alsved, M.,Al-Hunaiti, A.,Saksela, K.,Aqel, H.,Junninen, H.,Mahura, A.,Kulmala, M.**. Indoor Model Simulation for COVID-19 Transport and Exposure. *International Journal of Environmental Research & Public Health [Electronic Resource].* 2021. 18:12

**Huttel, F. B.,Iversen, A. M.,Hansen, M. B.,Ersboll, B. K.,Ellermann-Eriksen, S.,Olsen, N. L.**. Analysis of social interactions and risk factors relevant to the spread of infectious diseases at hospitals and nursing homes. *PLoS ONE.* 2021. 16(9 September) (no pagination):#pages#

**Hammer, A. S.,Quaade, M. L.,Rasmussen, T. B.,Fonager, J.,Rasmussen, M.,Mundbjerg, K.,Lohse, L.,Strandbygaard, B.,Jorgensen, C. S.,Alfaro-Nunez, A.,Rosenstierne, M. W.,Boklund, A.,Halasa, T.,Fomsgaard, A.,Belsham, G. J.,Botner, A.**. SARS-CoV-2 transmission between mink (neovison vison) and Humans, Denmark. *Emerging Infectious Diseases.* 2021. 27(2):547-551

**He, R.,Gao, L.,Trifonov, M.,Hong, J.**. Aerosol generation from different wind instruments. *Journal of Aerosol Science.* 2021. 151 (no pagination):#pages#

**Hirose, R., Miyazaki, H., Bandou, R., Watanabe, N., Yoshida, T., Daidoji, T., Itoh, Y., Nakaya, T.**. Stability of SARS-CoV-2 and influenza virus varies across different paper types. *Journal of Infection & Chemotherapy.* 2021. 13:13

**Holgate, S. L.,Dramowski, A.,Van Niekerk, M.,Hassan, H.,Prinsloo, Y.,Bekker, A.**. Healthcare-Associated Severe Acute Respiratory Syndrome Coronavirus 2 Transmission in a Neonatal Unit: The Importance of Universal Masking, Hand Hygiene, and Symptom Screening in Containment. *Journal of the Pediatric Infectious Diseases Society.* 2021. 10(5):665-668

**Hu, Q.,He, L.,Zhang, Y.**. Community Transmission via Indirect Media-To-Person Route: A Missing Link in the Rapid Spread of COVID-19. *Frontiers in Public Health.* 2021. 9:687937

**Ibrahim, J. E.,Aitken, G.**. A Proactive Nursing Home Risk Stratification Model for Disaster Response: Lessons Learned from COVID-19 to Optimize Resource Allocation. *Journal of the American Medical Directors Association.* 2021. 22(9):1831-1839.e1

**Irfan, O.,Li, J.,Tang, K.,Wang, Z.,Bhutta, Z. A.**. Risk of infection and transmission of SARS-CoV-2 among children and adolescents in households, communities and educational settings: A systematic review and meta-analysis. *Journal of Global Health.* 2021. 11:05013

**Illingworth, C. J.,Hamilton, W. L.,Warne, B.,Routledge, M.,Popay, A.,Jackson, C.,Fieldman, T.,Meredith, L. W.,Houldcroft, C. J.,Hosmillo, M.,Jahun, A. S.,Caller, L. G.,Caddy, S. L.,Yakovleva, A.,Hall, G.,Khokhar, F. A.,Feltwell, T.,Pinckert, M. L.,Georgana, I.,Chaudhry, Y.,Curran, M. D.,Parmar, S.,Sparkes, D.,Rivett, L.,Jones, N. K.,Sridhar, S.,Forrest, S.,Dymond, T.,Grainger, K.,Workman, C.,Ferris, M.,Gkrania-Klotsas, E.,Brown, N. M.,Weekes, M. P.,Baker, S.,Peacock, S. J.,Goodfellow, I. G.,Gouliouris, T.,de Angelis, D.,Torok, M. E.**. Superspreaders drive the largest outbreaks of hospital onset COVID-19 infections. *eLife.* 2021. 10:24

**Islam, A.,Sayeed, M. A.,Rahman, M. K.,Ferdous, J.,Islam, S.,Hassan, M. M.**. Geospatial dynamics of COVID-19 clusters and hotspots in Bangladesh. *Transboundary & Emerging Diseases.* 2021. 01:01

**Islam, N.,Bukhari, Q.,Jameel, Y.,Shabnam, S.,Erzurumluoglu, A. M.,Siddique, M. A.,Massaro, J. M.,D'Agostino, R. B.**. COVID-19 and climatic factors: A global analysis. *Environmental Research.* 2021. 193 (no pagination):#pages#

**Islam, T.,Lahijani, M. S.,Srinivasan, A.,Namilae, S.,Mubayi, A.,Scotch, M.**. From bad to worse: airline boarding changes in response to COVID-19. *Royal Society Open Science.* 2021. 8:201019

**Ing, A. J.,Cocks, C.,Green, J. P.**. COVID-19: in the footsteps of Ernest Shackleton. *Thorax.* 2020. 75:693-694

**Isaacs, D.,Britton, P.,Howard-Jones, A.,Kesson, A.,Khatami, A.,Marais, B.,Nayda, C.,Outhred, A.**. To what extent do children transmit SARS-CoV-2 virus?. *Journal of Paediatrics & Child Health.* 2020. 56:978-979

**Iwasaki, N.,Sekino, M.,Egawa, T.,Yamashita, K.,Hara, T.**. Use of a plastic barrier curtain to minimize droplet transmission during tracheal extubation in patients with COVID-19. *Acute Medicine and Surgery.* 2020. 7(1) (no pagination):#pages#

**Javid, B.,Bassler, D.,Bryant, M. B.,Cevik, M.,Tufekci, Z.,Baral, S.**. Should masks be worn outdoors?. *The BMJ.* 2021. 373 (no pagination):#pages#

**Jiang, Y.,Niu, W.,Wang, Q.,Zhao, H.,Meng, L.,Zhang, C.**. Characteristics of a family cluster of Severe Acute Respiratory Syndrome Coronavirus 2 in Henan, China. *Journal of Infection.* 2020. 81(2):e46-e48

**Joob, B.,Wiwanitkit, V.**. Outbreak of COVID-19 in Thailand: Time serial analysis on imported and local transmission cases. *International Journal of Preventive Medicine.* 2020. 11:#pages#

**Joshi, R. K.,Ray, R. K.,Adhya, S.,Chauhan, V. P. S.,Pani, S.**. Spread of COVID-19 by asymptomatic cases: evidence from military quarantine facilities. *BMJ Military Health.* 2021. 167:217-218

**Jarvis, K. F.,Kelley, J. B.**. Temporal dynamics of viral load and false negative rate influence the levels of testing necessary to combat COVID-19 spread. *Scientific Reports.* 2021. 11:9221

**Jen, T. H.,Chien, T. W.,Yeh, Y. T.,Lin, J. J.,Kuo, S. C.,Chou, W.**. Geographic risk assessment of COVID-19 transmission using recent data: An observational study. *Medicine.* 2020. 99:e20774

**Jing, Q. L.,Liu, M. J.,Yuan, J.,Zhang, Z. B.,Zhang, A. R.,Dean, N. E.,Luo, L.,Ma, M.,Longini, I.,Kenah, E.,Lu, Y.,Ma, Y.,Jalali, N.,Fang, L. Q.,Yang, Z. C.,Yang, Y.**. Household Secondary Attack Rate of COVID-19 and Associated Determinants. *MedRxiv : the Preprint Server for Health Sciences.* 2020. 15:15

**Johansson, M. A.,Quandelacy, T. M.,Kada, S.,Prasad, P. V.,Steele, M.,Brooks, J. T.,Slayton, R. B.,Biggerstaff, M.,Butler, J. C.**. SARS-CoV-2 Transmission From People Without COVID-19 Symptoms. *JAMA Network Open.* 2021. 4:e2035057

**Johnson, K. D.,Beiglbock, M.,Eder, M.,Grass, A.,Hermisson, J.,Pammer, G.,Polechova, J.,Toneian, D.,Wolfl, B.**. Disease momentum: Estimating the reproduction number in the presence of superspreading. *Infectious Disease Modelling.* 2021. 6:706-728

**Johnson, K. E.,Lachmann, M.,Stoddard, M.,Pasco, R.,Fox, S. J.,Meyers, L. A.,Chakravarty, A.**. Detecting in-school transmission of SARS-CoV-2 from case ratios and documented clusters. *MedRxiv : the Preprint Server for Health Sciences.* 2021. 28:28

**Johnson, K. E.,Stoddard, M.,Nolan, R. P.,White, D. E.,Hochberg, N. S.,Chakravarty, A.**. In the long shadow of our best intentions: Model-based assessment of the consequences of school reopening during the COVID-19 pandemic. *PLoS ONE [Electronic Resource].* 2021. 16:e0248509

**Jones, B.,Sharpe, P.,Iddon, C.,Hathway, E. A.,Noakes, C. J.,Fitzgerald, S.**. Modelling uncertainty in the relative risk of exposure to the SARS-CoV-2 virus by airborne aerosol transmission in well mixed indoor air. *Building & Environment.* 2021. 191:107617

**Jung, S. M.,Endo, A.,Akhmetzhanov, A. R.,Nishiura, H.**. Predicting the effective reproduction number of COVID-19: Inference using human mobility, temperature, and risk awareness. *International Journal of Infectious Diseases.* 2021. 07:07

**Jung, S. M.,Endo, A.,Kinoshita, R.,Nishiura, H.**. Projecting a second wave of COVID-19 in Japan with variable interventions in high-risk settings. *Royal Society Open Science.* 2021. 8:202169

**Jamal, M.,Shah, M.,Almarzooqi, S. H.,Aber, H.,Khawaja, S.,El Abed, R.,Alkhatib, Z.,Samaranayake, L. P.**. Overview of transnational recommendations for COVID-19 transmission control in dental care settings. *Oral Diseases.* 2021. 27(S3):655-664

**James, R. H.,Doyle, C. P.,Cooper, D. J.**. Descriptive record of the activity of military critical care transfer teams deployed to London in April 2020 to undertake transfer of patients with COVID-19. *BMJ Military Health.* 2020. 28:28

**Jernigan, D. B.,Cdc Covid- Response Team**. Update: Public Health Response to the Coronavirus Disease 2019 Outbreak - United States, February 24, 2020. *MMWR - Morbidity & Mortality Weekly Report.* 2020. 69:216-219

**Jia, Y.,Xiang, Y.,Guo, S.,Guo, L.,Guo, L.,Cheng, Z.,Zhang, Y.,Zhang, L.,Long, E.**. Analysis on the risk of respiratory virus transmission by air conditioning system operation based on experimental evidence. *Environmental Science & Pollution Research.* 2021. 29:29

**Jian, M. J.,Chung, H. Y.,Chang, C. K.,Hsieh, S. S.,Lin, J. C.,Yeh, K. M.,Chen, C. W.,Chang, F. Y.,Hung, K. S.,Liu, M. T.,Yang, J. R.,Chang, T. Y.,Tang, S. H.,Perng, C. L.,Shang, H. S.**. Genomic analysis of early transmissibility assessment of the D614G mutant strain of SARS-CoV-2 in travelers returning to Taiwan from the United States of America. *PeerJ.* 2021. 9:e11991

**Judson, S. D.,Munster, V. J.**. Nosocomial Transmission of Emerging Viruses via Aerosol-Generating Medical Procedures. *Viruses.* 2019. 11:12

**Kadi, N.,Khelfaoui, M.**. Population density, a factor in the spread of COVID-19 in Algeria: statistic study. *Bulletin of the National Research Centre (Cairo).* 2020. 44:138

**Kain, M. P.,Childs, M. L.,Becker, A. D.,Mordecai, E. A.**. Chopping the tail: How preventing superspreading can help to maintain COVID-19 control. *Epidemics.* 2021. 34:100430

**Kamgar, F.,Hughes, R.,Lucherini, S.**. PIN49 Developing a Predictive Algorithm to Identify the Key Factors Impacting on the Transmission and Fatalities from COVID-19. *Value in Health.* 2021. 24(Supplement 1):S114

**Kan, Z.,Kwan, M. P.,Huang, J.,Wong, M. S.,Liu, D.**. Comparing the space-time patterns of high-risk areas in different waves of COVID-19 in Hong Kong. *Transactions in Gis.* 2021. 12:12

**Kang, Q.,Song, X.,Xin, X.,Chen, B.,Chen, Y.,Ye, X.,Zhang, B.**. Machine Learning-Aided Causal Inference Framework for Environmental Data Analysis: A COVID-19 Case Study. *Environmental Science & Technology.* 2021. 24:24

**Ke, R.,Martinez, P. P.,Smith, R. L.,Gibson, L. L.,Mirza, A.,Conte, M.,Gallagher, N.,Luo, C. H.,Jarrett, J.,Conte, A.,Liu, T.,Farjo, M.,Walden, K. K. O.,Rendon, G.,Fields, C. J.,Wang, L.,Fredrickson, R.,Edmonson, D. C.,Baughman, M. E.,Chiu, K. K.,Choi, H.,Scardina, K. R.,Bradley, S.,Gloss, S. L.,Reinhart, C.,Yedetore, J.,Quicksall, J.,Owens, A. N.,Broach, J.,Barton, B.,Lazar, P.,Heetderks, W. J.,Robinson, M. L.,Mostafa, H. H.,Manabe, Y. C.,Pekosz, A.,McManus, D. D.,Brooke, C. B.**. Daily sampling of early SARS-CoV-2 infection reveals substantial heterogeneity in infectiousness. *MedRxiv : the Preprint Server for Health Sciences.* 2021. 12:12

**Kenji, Mizumoto, Gerardo, Chowell**. Transmission potential of the New Corona (COVID-19) onboard the Princess Cruises Ship, 2020. *#journal#.* 2020. #volume#:#pages#

**Khan, A.,Ali, M.,Iqbal, W.,Imran, M.**. Effect of high and low risk susceptibles in the transmission dynamics of COVID-19 and control strategies. *PLoS ONE [Electronic Resource].* 2021. 16:e0257354

**Kiang, M. V.,Chin, E. T.,Huynh, B. Q.,Chapman, L. A. C.,Rodriguez-Barraquer, I.,Greenhouse, B.,Rutherford, G. W.,Bibbins-Domingo, K.,Havlir, D.,Basu, S.,Lo, N. C.**. Routine asymptomatic testing strategies for airline travel during the COVID-19 pandemic: a simulation analysis. *MedRxiv : the Preprint Server for Health Sciences.* 2020. 11:11

**Kim, J. H., Lee, H., Won, Y. S., Son, W. S., Im, J.**. Rapid transmission of coronavirus disease 2019 within a religious sect in South Korea: A mathematical modeling study. *Epidemics.* 2021. 37:100519

**Kim, S.,Choi, S.,Ko, Y.,Ki, M.,Jung, E.**. Risk estimation of the SARS-CoV-2 acute respiratory disease outbreak outside China. *Theoretical Biology & Medical Modelling.* 2020. 17:9

**Klausner, Z.,Fattal, E.,Hirsch, E.,Shapira, S. C.**. A single holiday was the turning point of the COVID-19 policy of Israel. *International Journal of Infectious Diseases.* 2020. 101:368-373

**Koeppel, L.,Gottschalk, C.,Welker, A.,Knorr, B.,Denkinger, C. M.**. Prediction of local COVID-19 spread in Heidelberg. *F1000Research.* 2020. 9 (no pagination):#pages#

**Kolinski, J. M.,Schneider, T. M.**. Superspreading events suggest aerosol transmission of SARS-CoV-2 by accumulation in enclosed spaces. *Physical Review.* 2021. 103:033109

**Kolnes, N. H.,Eikeland, S. N.,Ersdal, T. A.,Braut, G. S.**. Estimating the consequences of a COVID-19 super spreader: A stochastic model of a night on the town. *Scandinavian Journal of Public Health.* 2021. #volume#:14034948211031400

**Kraay, A. N. M.,Hayashi, M. A. L.,Berendes, D. M.,Sobolik, J. S.,Leon, J. S.,Lopman, B. A.**. Risk for Fomite-Mediated Transmission of SARS-CoV-2 in Child Daycares, Schools, Nursing Homes, and Offices. *Emerging Infectious Diseases.* 2021. 27:1229-1231

**Kranjac, A. W.,Kranjac, D.**. County-Level Factors That Influenced the Trajectory of COVID-19 Incidence in the New York City Area. *Health Security.* 2021. 19:S27-S33

**Kremer, C.,Torneri, A.,Boesmans, S.,Meuwissen, H.,Verdonschot, S.,Driessche, K. V.,Althaus, C. L.,Faes, C.,Hens, N.**. Quantifying superspreading for COVID-19 using Poisson mixture distributions. *MedRxiv : the Preprint Server for Health Sciences.* 2020. 30:30

**Kucharski, A. J.,Klepac, P.,Conlan, A. J. K.,Kissler, S. M.,Tang, M. L.,Fry, H.,Gog, J. R.,Edmunds, W. J.,Cmmid Covid- working group**. Effectiveness of isolation, testing, contact tracing, and physical distancing on reducing transmission of SARS-CoV-2 in different settings: a mathematical modelling study. *The Lancet Infectious Diseases.* 2020. 20:1151-1160

**Kudryashova, O. B.,Muravlev, E. V.,Antonnikova, A. A.,Titov, S. S.**. Propagation of viral bioaerosols indoors. *PLoS ONE [Electronic Resource].* 2021. 16:e0244983

**Kwan, T. H.,Wong, N. S.,Yeoh, E. K.,Lee, S. S.**. Mining relationships between transmission clusters from contact tracing data: An application for investigating COVID-19 outbreak. *Journal of the American Medical Informatics Association.* 2021. 08:08

**Kain, D. C., McCreight, L. J., Johnstone, J.**. Dealing with coronavirus disease 2019 (COVID-19) outbreaks in long-term care homes: A protocol for room moving and cohorting. *Infection Control and Hospital Epidemiology.* 2021. 42(11):1402-1403

**Kanamori, H.,Weber, D. J.,Rutala, W. A.**. Role of the Healthcare Surface Environment in Severe Acute Respiratory Syndrome Coronavirus 2 (SARS-CoV-2) Transmission and Potential Control Measures. *Clinical Infectious Diseases.* 2021. 72(11):2052-2061

**Kelvin, A. A.,Halperin, S.**. COVID-19 in children: the link in the transmission chain. *The Lancet Infectious Diseases.* 2020. 20:633-634

**Khan, R. F.,Meyer, J. D.**. How does the hierarchy of controls integrate with the epidemiologic triangle to help address and understand transmission of SARS-COV-2?. *Journal of Occupational and Environmental Medicine.* 2020. 62(11):E665-E668

**Kharbach, Y.,Khallouk, A.**. Contamination risk in urology operating room during the COVID-19 pandemic. *Sao Paulo Medical Journal.* 2020. 138(4):345-346

**Kim, H. J.,Kwon, Y. H.,Jeon, S. W.,Nam, S. Y.,Lee, H. S.,Lee, J. S.,Cho, C. M.,Kwon, K. T.,Ham, J. Y.,Kim, C.**. Unexpected exposure to coronavirus disease at the endoscopic room: What should we do?. *Korean Journal of Helicobacter and Upper Gastrointestinal Research.* 2020. 20(3):248-250

**Kobayashi, H.,Takimoto, T.,Kitaoka, H.,Kijima, T.**. Aerosol spread with use of high-flow nasal cannulae: a computational fluid dynamics analysis. *Journal of Hospital Infection.* 2020. 106(1):204-205

**Kooraki, S.,Hosseiny, M.,Myers, L.,Gholamrezanezhad, A.**. Re: Ventilation-Perfusion Scans During the Coronavirus Disease 2019 (COVID-19) Outbreak. *Journal of the American College of Radiology.* 2020. 17(6):698-699

**Kraemer, M. U. G., Pybus, O. G., Fraser, C., Cauchemez, S., Rambaut, A., Cowling, B. J.**. Monitoring key epidemiological parameters of SARS-CoV-2 transmission. *Nature Medicine.* 2021. 27:1854-1855

**Kuba, Y., Shingaki, A., Nidaira, M., Kakita, T., Maeshiro, N., Oyama, M., Kudeken, T., Miyagi, A., Yamauchi, M., Kyan, H.**. Characteristics of household transmission of covid-19 during its outbreak in okinawa, japan from february to may 2020. *Japanese Journal of Infectious Diseases.* 2021. 74(6):579-583

**Kumar, D.,Meena, D. S.,Garg, M. K.,Misra, S.**. Super-spreader resurgence in COVID-19: Past encounters and future repercussion. *Journal of Family Medicine & Primary Care.* 2020. 9:5404-5405

**Kuttiatt, V. S.,Menon, R. P.,Abraham, P. R.,Sharma, S.**. Should Schools Reopen Early or Late? - Transmission Dynamics of COVID-19 in Children. *Indian Journal of Pediatrics.* 2020. 87(9):755-756

**Kwaan, H. C.**. Coronavirus Disease 2019: The Role of the Fibrinolytic System from Transmission to Organ Injury and Sequelae. *Seminars in Thrombosis and Hemostasis.* 2020. 46(7):841-844

**Kamga, C.,Eickemeyer, P.**. Slowing the spread of COVID-19: Review of "Social distancing" interventions deployed by public transit in the United States and Canada. *Transport Policy.* 2021. 106:25-36

**Kapoor, D. A.,Latino, K.,Hodes, G.,Anderson, A. E.,Anderson, J.,Cognetti, M.,Patel, C.**. The Impact of Systematic Safety Precautions on COVID-19 Risk Exposure and Transmission Rates in Outpatient Healthcare Workers. *Reviews in Urology.* 2020. 22:93-101

**Karia, R.,Gupta, I.,Khandait, H.,Yadav, A.**. COVID-19 and its Modes of Transmission. *SN Comprehensive Clinical Medicine.* 2020. 2(10):1798-1801

**Kaushal, D.,Nair, N. P.,Soni, K.,Goyal, A.,Choudhury, B.,Rajan, N.**. Endoscopy in Otorhinolaryngology During Corona Outbreak: A Proposal for Safe Practice. *Indian Journal of Otolaryngology & Head & Neck Surgery.* 2020. #volume#:1-6

**Kayode, A. J., Banji-Onisile, F. O., Olaniran, A. O., Okoh, A. I.**. An overview of the pathogenesis, transmission, diagnosis, and management of endemic human coronaviruses: A reflection on the past and present episodes and possible future outbreaks. *Pathogens.* 2021. 10(9) (no pagination):#pages#

**Koh, W. C.,Naing, L.,Chaw, L.,Rosledzana, M. A.,Alikhan, M. F.,Jamaludin, S. A.,Amin, F.,Omar, A.,Shazli, A.,Griffith, M.,Pastore, R.,Wong, J.**. What do we know about SARS-CoV-2 transmission? A systematic review and meta-analysis of the secondary attack rate and associated risk factors. *PLoS ONE [Electronic Resource].* 2020. 15:e0240205

**Krause, Heather U. S. Government Accountability Office**. Air Travel and Communicable Diseases: Status of Research Efforts and Action Still Needed to Develop Federal Preparedness Plan. *#journal#.* 2020. #volume#:16p-16p

**Kumar, S.,Jha, S.,Rai, S. K.**. Significance of super spreader events in COVID-19. *Indian Journal of Public Health.* 2020. 64:S139-S141

**Landeros, A.,Ji, X.,Lange, K.,Stutz, T. C.,Xu, J.,Sehl, M. E.,Sinsheimer, J. S.**. An examination of school reopening strategies during the SARS-CoV-2 pandemic. *PLoS ONE.* 2021. 16(5 May) (no pagination):#pages#

**Lau, M. S. Y.,Grenfell, B.,Thomas, M.,Bryan, M.,Nelson, K.,Lopman, B.**. Characterizing superspreading events and age-specific infectiousness of SARS-CoV-2 transmission in Georgia, USA. *Proceedings of the National Academy of Sciences of the United States of America.* 2020. 117:22430-22435

**Laurio Dizon, R.**. The heterogeneous age-mixing model of estimating the covid cases of different local government units in the National Capital Region, Philippines. *Clinical Epidemiology and Global Health.* 2021. 9:12-16

**Lee, H., Han, C., Jung, J., Lee, S.**. Analysis of superspreading potential from transmission clusters of covid-19 in south korea. *International Journal of Environmental Research and Public Health.* 2021. 18(24) (no pagination):#pages#

**Lee, J. H.,Rounds, M.,McGain, F.,Schofield, R.,Skidmore, G.,Wadlow, I.,Kevin, K.,Stevens, A.,Marshall, C.,Irving, L.,Kainer, M.,Buising, K.,Monty, J.**. Effectiveness of portable air filtration on reducing indoor aerosol transmission: preclinical observational trials. *Journal of Hospital Infection.* 2021. 22:22

**Lee, W.,Choi, H. M.,Heo, S.,Fong, K. C.,Yang, J.,Park, C.,Kim, H.,Bell, M. L.**. Urban environments and COVID-19 in three Eastern states of the United States. *Science of the Total Environment.* 2021. 779 (no pagination):#pages#

**Lelieveld, J.,Helleis, F.,Borrmann, S.,Cheng, Y.,Drewnick, F.,Haug, G.,Klimach, T.,Sciare, J.,Su, H.,Poschl, U.**. Model Calculations of Aerosol Transmission and Infection Risk of COVID-19 in Indoor Environments. *International Journal of Environmental Research & Public Health [Electronic Resource].* 2020. 17:03

**Leung, K.,Shum, M. H. H.,Leung, G. M.,Lam, T. T. Y.,Wu, J. T.**. Early transmissibility assessment of the N501Y mutant strains of SARS-CoV-2 in the United Kingdom, October to November 2020. *Eurosurveillance.* 2020. 26:#pages#

**Lewer, D.,Braithwaite, I.,Bullock, M.,Eyre, M. T.,White, P. J.,Aldridge, R. W.,Story, A.,Hayward, A. C.**. COVID-19 among people experiencing homelessness in England: a modelling study. *The Lancet Respiratory Medicine.* 2020. 8:1181-1191

**Li, B.,Peng, Y.,He, H.,Wang, M.,Feng, T.**. Built environment and early infection of COVID-19 in urban districts: A case study of Huangzhou. *Sustainable Cities and Society.* 2021. 66:102685

**Li, C.,Tang, H.**. Comparison of COVID-19 infection risks through aerosol transmission in supermarkets and small shops. *Sustainable Cities and Society.* 2022. 76:103424

**Li, H., Shankar, S. N., Witanachchi, C. T., Lednicky, J. A., Loeb, J. C., Alam, M. M., Fan, Z. H., Mohamed, K., Eiguren-Fernandez, A., Wu, C. Y.**. Environmental Surveillance and Transmission Risk Assessments for SARS-CoV-2 in a Fitness Center. *Aerosol & Air Quality Research.* 2021. 21:#pages#

**Li, H.,Leong, F. Y.,Xu, G.,Ge, Z.,Kang, C. W.,Lim, K. H.**. Dispersion of evaporating cough droplets in tropical outdoor environment. *Physics of Fluids.* 2020. 32:113301

**Li, H.,Leong, F. Y.,Xu, G.,Kang, C. W.,Lim, K. H.,Tan, B. H.,Loo, C. M.**. Airborne dispersion of droplets during coughing: a physical model of viral transmission. *Scientific Reports.* 2021. 11:4617

**Li, M.,Liu, K.,Song, Y.,Wang, M.,Wu, J.**. Serial Interval and Generation Interval for Imported and Local Infectors, Respectively, Estimated Using Reported Contact-Tracing Data of COVID-19 in China. *Frontiers in Public Health.* 2020. 8:577431

**Li, M.,Zhang, Z.,Cao, W.,Liu, Y.,Du, B.,Liu, Q.,Uddin, M. N.,Jiang, S.,Chen, C.,Zhang, Y.,Wang, X.**. Identifying novel factors associated with COVID-19 transmission and fatality using the machine learning approach. *Science of the Total Environment.* 2021. 764 (no pagination):#pages#

**Li, Y.,Campbell, H.,Kulkarni, D.,Harpur, A.,Nundy, M.,Wang, X.,Nair, H.**. The temporal association of introducing and lifting non-pharmaceutical interventions with the time-varying reproduction number (R) of SARS-CoV-2: a modelling study across 131 countries. *The Lancet Infectious Diseases.* 2021. 21(2):193-202

**Li, Y.,Hu, T.,Gai, X.,Zhang, Y.,Zhou, X.**. Transmission Dynamics, Heterogeneity and Controllability of SARS-CoV-2: A Rural-Urban Comparison. *International Journal of Environmental Research & Public Health [Electronic Resource].* 2021. 18:14

**Lim, C. Y.,Bohn, M. K.,Lippi, G.,Ferrari, M.,Loh, T. P.,Yuen, K. Y.,Adeli, K.,Horvath, A. R.,Ifcc Task Force on COVID**. Staff rostering, split team arrangement, social distancing (physical distancing) and use of personal protective equipment to minimize risk of workplace transmission during the COVID-19 pandemic: A simulation study. *Clinical Biochemistry.* 2020. 86:15-22

**Lim, J. T., Maung, K., Tan, S. T., Ong, S. E., Lim, J. M., Koo, J. R., Sun, H., Park, M., Tan, K. W., Yoong, J., Cook, A. R., Dickens, B. S. L.**. Estimating direct and spill-over impacts of political elections on COVID-19 transmission using synthetic control methods. *PLoS Computational Biology.* 2021. 17(5) (no pagination):#pages#

**Liu, F.,Luo, Z.,Li, Y.,Zheng, X.,Zhang, C.,Qian, H.**. Revisiting physical distancing threshold in indoor environment using infection-risk-based modeling. *Environment International.* 2021. 153 (no pagination):#pages#

**Liu, H.,He, S.,Shen, L.,Hong, J.**. Simulation-based study of COVID-19 outbreak associated with air-conditioning in a restaurant. *Physics of Fluids.* 2021. 33:023301

**Liu, M.,Li, Z.,Zhu, Y.,Liu, Y.,Wang, X.,Tao, L.,Guo, X.**. The spatial clustering analysis of COVID-19 and its associated factors in mainland China at the prefecture level. *Science of the Total Environment.* 2021. 777 (no pagination):#pages#

**Liu, P.,McQuarrie, L.,Song, Y.,Colijn, C.**. Modelling the impact of household size distribution on the transmission dynamics of COVID-19. *Journal of the Royal Society Interface.* 2021. 18(177) (no pagination):#pages#

**Liu, W.,Liu, L.,Xu, C.,Fu, L.,Wang, Y.,Nielsen, P. V.,Zhang, C.**. Exploring the potentials of personalized ventilation in mitigating airborne infection risk for two closely ranged occupants with different risk assessment models. *Energy & Buildings.* 2021. 253:111531

**Liu, Y.,Gu, Z.,Xia, S.,Shi, B.,Zhou, X. N.,Shi, Y.,Liu, J.**. What are the underlying transmission patterns of COVID-19 outbreak? An age-specific social contact characterization. *EClinicalMedicine.* 2020. 22:100354

**Loo, B. P. Y.,Tsoi, K. H.,Wong, P. P. Y.,Lai, P. C.**. Identification of superspreading environment under COVID-19 through human mobility data. *Scientific Reports.* 2021. 11:4699

**Lu, H.,Weintz, C.,Pace, J.,Indana, D.,Linka, K.,Kuhl, E.**. Are college campuses superspreaders? A data-driven modeling study. *Computer Methods in Biomechanics & Biomedical Engineering.* 2021. 24:1136-1145

**Luo, L., Wen, W., Wang, C. Y., Zhou, M., Ni, J., Jiang, J., Chen, J., Wang, M. W., Feng, Z., Cheng, Y. R.**. Exploring the pattern of early COVID-19 transmission caused by population migration based on 14 cities in Hubei Province, China. *Risk Management and Healthcare Policy.* 2021. 14:4393-4399

**Lau, E. H. Y.,Leung, G. M.**. Reply to: Is presymptomatic spread a major contributor to COVID-19 transmission?. *Nature Medicine.* 2020. 26(10):1534-1535

**Lednicky, J. A.**. A practical and economic approach for assessing potential SARS-CoV-2 transmission risk in COVID-19 patients. *Clinical Infectious Diseases.* 2020. 01:01

**Lee, B. U.**. Why does the sars-cov-2 delta voc spread so rapidly? Universal conditions for the rapid spread of respiratory viruses, minimum viral loads for viral aerosol generation, effects of vaccination on viral aerosol generation, and viral aerosol clouds. *International Journal of Environmental Research and Public Health.* 2021. 18(18) (no pagination):#pages#

**Lee, B.,Raszka, W. V.**. COVID-19 in children: Looking forward, not back. *Pediatrics.* 2021. 147(1) (no pagination):#pages#

**Lee, B.,Raszka, W. V.**. COVID-19 transmission and children: The child is not to blame. *Pediatrics.* 2020. 146(2) (no pagination):#pages#

**Lee, E. J.,Kim, D. H.,Chang, S. H.,Suh, S. B.,Lee, J.,Lee, H.,Han, M. S.**. Absence of SARS-CoV-2 transmission from children in isolation to Guardians, South Korea. *Emerging Infectious Diseases.* 2021. 27(1):308-310

**Lee, J. I.,Bullington, B. W.,Simon, M. S.,Crossman, D. J.,Evans, A. T.,McNairy, M. L.**. COVID-19 Infections Among General Internal Medicine Faculty at a New York Teaching Hospital: a Descriptive Report. *Journal of General Internal Medicine.* 2021. 36(4):1153-1155

**Leeds, C.**. COVID 19: Health care workers, risks, protection and transmission. *The Lancet Regional Health. Europe.* 2021. 1:100022

**Lesser, K.,Whittaker, G. R.**. Vaccination for COVID-19: benchmarks in public health and virus transmission. *Public Health.* 2021. 197:e23

**Leung, N. H. L.**. Transmissibility and transmission of respiratory viruses. *Nature Reviews. Microbiology.* 2021. 19:528-545

**Li, C.,Zhao, W.,He, C.,Wu, D.,Yue, Y.,Chen, Y.**. COVID-19 prevention and control strategies for psychiatric hospitals. *Psychiatry Research.* 2020. 289 (no pagination):#pages#

**Li, Y.,Ren, B.,Peng, X.,Hu, T.,Li, J.,Gong, T.,Tang, B.,Xu, X.,Zhou, X.**. Saliva is a non-negligible factor in the spread of COVID-19. *Molecular Oral Microbiology.* 2020. 35:141-145

**Liu, C. K.,Ghai, S.,Waikar, S. S.,Weiner, D. E.**. COVID-19 Infection Risk Among Hemodialysis Patients in Long-term Care Facilities. *Kidney Medicine.* 2020. 2(6):810-811

**Liu, Y.,Eggo, R. M.,Kucharski, A. J.**. Secondary attack rate and superspreading events for SARS-CoV-2. *Lancet.* 2020. 395:e47

**Lu, C. W.,Liu, X. F.,Jia, Z. F.**. 2019-nCoV transmission through the ocular surface must not be ignored. *The Lancet.* 2020. 395(10224):e39

**Luo, C.,Yao, L.,Zhang, L.,Yao, M.,Chen, X.,Wang, Q.,Shen, H.**. Possible Transmission of Severe Acute Respiratory Syndrome Coronavirus 2 (SARS-CoV-2) in a Public Bath Center in Huai'an, Jiangsu Province, China. *JAMA Network Open.* 2020. 3:e204583

**Luzzi, J. R.,Navarro, R.,Dinardo, C. L.**. COVID-19: Further evidence of no transfusion transmission. *Transfusion and Apheresis Science.* 2021. 60(1) (no pagination):#pages#

**Lance, C. G.**. PAP therapy increases the risk of transmission of COVID-19. *Cleveland Clinic Journal of Medicine.* 2020. 05:05

**Lane, C. R.,Sherry, N. L.,Porter, A. F.,Duchene, S.,Horan, K.,Andersson, P.,Wilmot, M.,Turner, A.,Dougall, S.,Johnson, S. A.,Sait, M.,Goncalves da Silva, A.,Ballard, S. A.,Hoang, T.,Stinear, T. P.,Caly, L.,Sintchenko, V.,Graham, R.,McMahon, J.,Smith, D.,Leong, L. E.,Meumann, E. M.,Cooley, L.,Schwessinger, B.,Rawlinson, W.,van Hal, S. J.,Stephens, N.,Catton, M.,Looker, C.,Crouch, S.,Sutton, B.,Alpren, C.,Williamson, D. A.,Seemann, T.,Howden, B. P.**. Genomics-informed responses in the elimination of COVID-19 in Victoria, Australia: an observational, genomic epidemiological study. *The lancet. Public Health.* 2021. 6:e547-e556

**Laxminarayan, R.,Wahl, B.,Dudala, S. R.,Gopal, K.,Mohan, C. B.,Neelima, S.,Jawahar Reddy, K. S.,Radhakrishnan, J.,Lewnard, J. A.**. Epidemiology and transmission dynamics of COVID-19 in two Indian states. *Science.* 2020. 370(6517) (no pagination):#pages#

**Leclerc, Q. J.,Fuller, N. M.,Knight, L. E.,Cmmid Covid- Working Group,Funk, S.,Knight, G. M.**. What settings have been linked to SARS-CoV-2 transmission clusters?. *Wellcome Open Research.* 2020. 5:83

**Lei, H.,Xu, X.,Xiao, S.,Wu, X.,Shu, Y.**. Household transmission of COVID-19-a systematic review and meta-analysis. *Journal of Infection.* 2020. 81(6):979-997

**Li, A.,Rieg, G.,Maldonado, A. M.,Concepcion, J.**. Effectiveness of personal protective equipment in preventing transmission of COVID-19 in healthcare workers. *Open Forum Infectious Diseases.* 2020. 7(SUPPL 1):S314

**Li, H.,Meng, S.,Tong, H.**. How to control cruise ship disease risk? Inspiration from the research literature. *Marine Policy.* 2021. 132:104652

**Li, X.,Xia, W. Y.,Jiang, F.,Liu, D. Y.,Lei, S. Q.,Xia, Z. Y.,Wu, Q. P.**. Review of the risk factors for SARS-CoV-2 transmission. *World Journal of Clinical Cases.* 2021. 9:1499-1512

**Li, X.,Xu, W.,Dozier, M.,He, Y.,Kirolos, A.,Lang, Z.,Song, P.,Theodoratou, E.,Uncover,**. The role of children in the transmission of SARS-CoV2: updated rapid review. *Journal of Global Health.* 2020. 10:021101

**Li, Y.,Zhang, R.,Zhao, J.,Molina, M. J.**. Understanding transmission and intervention for the COVID-19 pandemic in the United States. *Science of the Total Environment.* 2020. 748 (no pagination):#pages#

**Locas, A.,Brassard, J.,Rose-Martel, M.,Lambert, D.,Green, A.,Deckert, A.,Illing, M.**. A comprehensive risk pathway of the qualitative likelihood of human exposure to SARS-CoV-2 from the food chain. *Journal of Food Protection.* 2021. 09:09

**Loewenstein, K.,Saito, E.,Linder, H.**. Lessons Learned From a Mental Health Hospital: Managing COVID-19. *Journal of Nursing Administration.* 2020. 50:598-604

**Ma, X.,Lin, J.,Fang, S.**. Precautions in ophthalmic practice in a hospital with the risk of COVID-19: experience from China. *Acta Opthalmologica.* 2020. 98:e520-e521

**Madison, A. A.,Way, B. M.,Beauchaine, T. P.,Kiecolt-Glaser, J. K.**. Risk assessment and heuristics: How cognitive shortcuts can fuel the spread of COVID-19. *Brain, Behavior, and Immunity.* 2021. 94:6-7

**Maghami, S.,Yusoff, R.**. A simple, effective method of minimising viral transmission during fibreoptic bronchoscopy. *Anaesthesia and Intensive Care.* 2020. 48(6):488-490

**Mahapure, K. S.,Kulkarni, N. S.**. Asymptomatic Transmission of Severe Acute Respiratory Syndrome-Coronavirus 2 within a Family Cluster of 26 Cases: Why Quarantine is Important?. *Journal of global infectious diseases.* 2020. 12:115-116

**Marchetti, F.,Tamburlini, G.**. Time to go back to school: several good reasons beyond low infection risk. *BMJ.* 2020. 370:m2625

**Marinaccio, A.,Guerra, R.,Iavicoli, S.**. Work a key determinant in COVID-19 risk. *The Lancet Global Health.* 2020. 8(11):e1368

**Mat, N. F. C.,Edinur, H. A.,Razab, M. K. A. A.,Safuan, S.**. A single mass gathering resulted in massive transmission of COVID-19 infections in Malaysia with further international spread. *Journal of Travel Medicine.* 2020. 27(3) (no pagination):#pages#

**Mehta, S.,Machado, F.,Kwizera, A.,Papazian, L.,Moss, M.,Azoulay, E.,Herridge, M.**. COVID-19: a heavy toll on health-care workers. *The Lancet Respiratory Medicine.* 2021. 9(3):226-228

**Messali, S.,Bertelli, A.,Campisi, G.,Zani, A.,Ciccozzi, M.,Caruso, A.,Caccuri, F.**. A cluster of the new SARS-CoV-2 B.1.621 lineage in Italy and sensitivity of the viral isolate to the BNT162b2 vaccine. *Journal of Medical Virology.* 2021. 93(12):6468-6470

**Michaels, D.,Wagner, G. R.**. Occupational Safety and Health Administration (OSHA) and Worker Safety during the COVID-19 Pandemic. *JAMA - Journal of the American Medical Association.* 2020. 324(14):1389-1390

**Miller, F. D.,La Croix, S.,Brown, T.,Ramsey, L. T.,Morens, D.**. Unique pattern of COVID-19 infection in the State of Hawai'i. *International Journal of Infectious Diseases.* 2021. 103:298-299

**Miller, R.,Englund, K.**. Transmission and risk factors of OF COVID-19. *Cleveland Clinic Journal of Medicine.* 2020. 14:14

**Millones-Gomez, P. A.**. Clinical dental care: Is there a risk of SARS-CoV2 infection?. *Pesquisa Brasileira em Odontopediatria e Clinica Integrada.* 2020. 20:1

**Mohseni, A. H.,Taghinezhad, S. S.,Xu, Z.,Fu, X.**. Body fluids may contribute to human-to-human transmission of severe acute respiratory syndrome coronavirus 2: Evidence and practical experience. *Chinese Medicine (United Kingdom).* 2020. 15(1) (no pagination):#pages#

**Monsalud, C. F. L.,Lind, M. F. G.,Hines, C.,Schora, D.,Grant, J.,McElvania, E.,Singh, K.**. Mitigating Staff Shortages: Risk of Permitting Healthcare Workers to Return to Work after COVID-19 Exposure. *Infection Control and Hospital Epidemiology..* 2021. #volume#:#pages#

**Morawska, L.,Milton, D. K.**. It Is Time to Address Airborne Transmission of Coronavirus Disease 2019 (COVID-19). *Clinical Infectious Diseases.* 2020. 71(9):2311-2313

**Moreno, G.,Braun, K.,Pray, I.,Grande, K.,Jovaag, A.,Baker, D.,Baczenas, J.,Accola, M.,Kelly, G.,Rehrauer, W.,O'Connor, S.,Westergaard, R.,Friedrich, T.,O'Connor, D.**. Characterizing SARS-CoV-2 spread on college campuses. *Journal of the International AIDS Society.* 2021. 24(SUPPL 1):107-108

**Mowbray, N. G.,Ansell, J.,Horwood, J.,Cornish, J.,Rizkallah, P.,Parker, A.,Wall, P.,Spinelli, A.,Torkington, J.**. Safe management of surgical smoke in the age of COVID-19. *British Journal of Surgery.* 2020. 107:1406-1413

**Mueller, U. E.,Omosehin, O.,Akinkunmi, A. E.,Ayanbadejo, J. O.,Somefun, E. O.,Momah-Haruna, A. P.**. Contact Tracing in an African Megacity during COVID 19: Lessons Learned. *African Journal of Reproductive Health.* 2020. 24:27-31

**Muller, N.,Kunze, M.,Steitz, F.,Saad, N. J.,Muhlemann, B.,Beheim-Schwarzbach, J. I.,Schneider, J.,Drosten, C.,Murajda, L.,Kochs, S.,Ruscher, C.,Walter, J.,Zeitlmann, N.,Corman, V. M.**. Severe Acute Respiratory Syndrome Coronavirus 2 Outbreak Related to a Nightclub, Germany, 2020. *Emerging Infectious Diseases.* 2020. 27:645-648

**Munro, A.,Bowen, A. C.,Cevik, M.**. COVID-19, children and schools: overlooked and at risk. *Medical Journal of Australia.* 2021. 214(4):188-188.e1

**Madewell, Z. J.,Yang, Y.,Longini, I. M., Jr.,Halloran, M. E.,Dean, N. E.**. Household transmission of SARS-CoV-2: a systematic review and meta-analysis of secondary attack rate. *MedRxiv : the Preprint Server for Health Sciences.* 2020. 31:31

**Madewell, Z. J.,Yang, Y.,Longini, I. M., Jr.,Halloran, M. E.,Dean, N. E.**. Factors Associated With Household Transmission of SARS-CoV-2: An Updated Systematic Review and Meta-analysis. *JAMA Network Open.* 2021. 4:e2122240

**Madewell, Z. J.,Yang, Y.,Longini, I. M., Jr.,Halloran, M. E.,Dean, N. E.**. Household Transmission of SARS-CoV-2: A Systematic Review and Meta-analysis. *JAMA Network Open.* 2020. 3:e2031756

**Maggiulli, R.,Giancani, A.,Fabozzi, G.,Dovere, L.,Tacconi, L.,Amendola, M. G.,Cimadomo, D.,Ubaldi, F. M.,Rienzi, L.**. Assessment and management of the risk of SARS-CoV-2 infection in an IVF laboratory. *Reproductive Biomedicine Online.* 2020. 41:385-394

**Majra, D.,Benson, J.,Pitts, J.,Stebbing, J.**. SARS-CoV-2 (COVID-19) superspreader events. *Journal of Infection.* 2021. 82:36-40

**Marchesan, J. T., Warner, B. M., Byrd, K. M.**. The "oral" history of COVID-19: Primary infection, salivary transmission, and post-acute implications. *Journal of Periodontology.* 2021. 92(10):1357-1367

**Marcone, V.**. Reduction of contagion risks by sarscov-2 (Covid-19) in air-conditioned work environments. *Pain Physician.* 2020. 23(4 Special Issue):S475-S481

**Mariani, A.,Capurso, G.,Marasco, G.,Bertani, H.,Crino, S. F.,Magarotto, A.,Tringali, A.,Pasquale, L.,Arcidiacono, P. G.,Zagari, R. M.**. Factors associated with risk of COVID-19 contagion for endoscopy healthcare workers: A survey from the Italian society of digestive endoscopy. *Digestive & Liver Disease.* 2021. 53:534-539

**Marin-Garcia, D.,Moyano-Campos, J. J.,Bienvenido-Huertas, J. D.**. Distances of transmission risk of COVID-19 inside dwellings and evaluation of the effectiveness of reciprocal proximity warning sounds. *Indoor Air.* 2021. 31:335-347

**Meher, K. Prakash**. Eat, Pray, Work: A meta-analysis of COVID-19 Transmission Risk in Common Activities of Work and Leisure. *medRxiv.* 2020. #volume#:#pages#

**Melikov, A. K.,Ai, Z. T.,Markov, D. G.**. Intermittent occupancy combined with ventilation: An efficient strategy for the reduction of airborne transmission indoors. *Science of the Total Environment.* 2020. 744 (no pagination):#pages#

**Memish, Z. A.,Steffen, R.,White, P.,Dar, O.,Azhar, E. I.,Sharma, A.,Zumla, A.**. Mass gatherings medicine: public health issues arising from mass gathering religious and sporting events. *The Lancet.* 2019. 393(10185):2073-2084

**Merckx, J.,Labrecque, J. A.,Kaufman, J. S.**. Transmission of SARS-CoV-2 by Children. *Deutsches Arzteblatt International.* 2020. 117:553-560

**Meyerowitz, E. A.,Richterman, A.,Gandhi, R. T.,Sax, P. E.**. Transmission of SARS-CoV-2: A Review of Viral, Host, and Environmental Factors. *Annals of Internal Medicine.* 2021. 174:69-79

**Moon, J.,Ryu, B. H.**. Transmission risks of respiratory infectious diseases in various confined spaces: A meta-analysis for future pandemics. *Environmental Research.* 2021. 202:111679

**Mukhra, R.,Krishan, K.,Kanchan, T.**. Possible modes of transmission of Novel coronavirus SARS-CoV-2: a review. *Acta Bio-Medica de l Ateneo Parmense.* 2020. 91:e2020036

**Munjal, M.,Ahmed, S. M.,Garg, R.,Das, S.,Chatterjee, N.,Mittal, K.,Javeri, Y.,Saxena, S.,Khunteta, S.**. The Transport Medicine Society Consensus Guidelines for the Transport of Suspected or Confirmed COVID-19 Patients. *Indian Journal of Critical Care Medicine.* 2020. 24:763-770

**Murray, A. T.**. Planning for classroom physical distancing to minimize the threat of COVID-19 disease spread. *PLoS ONE.* 2020. 15(12 December) (no pagination):#pages#

**Marc, A.,Kerioui, M.,Blanquart, F.,Bertrand, J.,Mitja, O.,Corbacho-Monne, M.,Marks, M.,Guedj, J.**. Quantifying the relationship between sars-cov-2 viral load and infectiousness. *eLife.* 2021. 10 (no pagination):#pages#

**Martines, M. R.,Ferreira, R. V.,Toppa, R. H.,Assuncao, L. M.,Desjardins, M. R.,Delmelle, E. M.**. Detecting space-time clusters of COVID-19 in Brazil: mortality, inequality, socioeconomic vulnerability, and the relative risk of the disease in Brazilian municipalities. *Journal of Geographical Systems.* 2021. #volume#:1-30

**Mas, J. F.**. Stage 1 registered report: Spatiotemporal patterns of the COVID-19 epidemic in Mexico at the municipality level. *PeerJ.* 2021. 9 (no pagination):#pages#

**Massad, E.,Amaku, M.,Tadeu Covas, D.,Fernandes Lopez, L.,Coutinho, F. A. B.**. Estimating the effects of reopening of schools on the course of the epidemic of COVID-19. *Epidemiology & Infection.* 2021. 149:e86

**Mathai, V.,Das, A.,Bailey, J. A.,Breuer, K.**. Airflows inside passenger cars and implications for airborne disease transmission. *Science Advances.* 2021. 7:01

**Matrajt, L.,Eaton, J.,Leung, T.,Dimitrov, D.,Schiffer, J. T.,Swan, D. A.,Janes, H.**. Optimizing vaccine allocation for COVID-19 vaccines shows the potential role of single-dose vaccination. *Nature Communications.* 2021. 12(1) (no pagination):#pages#

**Mbuvha, R.,Marwala, T.**. Bayesian inference of COVID-19 spreading rates in South Africa. *PLoS ONE.* 2020. 15(8) (no pagination):#pages#

**McAloon, C. G.,Wall, P.,Griffin, J.,Casey, M.,Barber, A.,Codd, M.,Gormley, E.,Butler, F.,Mc, V. Messam L. L.,Walsh, C.,Teljeur, C.,Smyth, B.,Nolan, P.,Green, M. J.,O'Grady, L.,Culhane, K.,Buckley, C.,Carroll, C.,Doyle, S.,Martin, J.,More, S. J.**. Estimation of the serial interval and proportion of pre-symptomatic transmission events of COVID- 19 in Ireland using contact tracing data. *BMC Public Health.* 2021. 21:805

**McCarthy, J. E.,Dewitt, B. D.,Dumas, B. A.,McCarthy, M. T.**. Modeling the relative risk of SARS-CoV-2 infection to inform risk-cost-benefit analyses of activities during the SARS-CoV-2 pandemic. *PLoS ONE [Electronic Resource].* 2021. 16:e0245381

**McCarthy, Z.,Xiao, Y.,Scarabel, F.,Tang, B.,Bragazzi, N. L.,Nah, K.,Heffernan, J. M.,Asgary, A.,Murty, V. K.,Ogden, N. H.,Wu, J.**. Quantifying the shift in social contact patterns in response to non-pharmaceutical interventions. *Journal of Mathematics in Industry.* 2020. 10:28

**McGee, R. S.,Homburger, J. R.,Williams, H. E.,Bergstrom, C. T.,Zhou, A. Y.**. Model-driven mitigation measures for reopening schools during the COVID-19 pandemic. *Proceedings of the National Academy of Sciences of the United States of America.* 2021. 118:28

**McLaughlin, J. M., Khan, F., Pugh, S., Angulo, F. J., Schmitt, H. J., Isturiz, R. E., Jodar, L., Swerdlow, D. L.**. County-level Predictors of Coronavirus Disease 2019 (COVID-19) Cases and Deaths in the United States: What Happened, and Where Do We Go from Here?. *Clinical infectious diseases : an official publication of the Infectious Diseases Society of America.* 2021. 73(7):e1814-e1821

**Mehta, S. H.,Clipman, S. J.,Wesolowski, A.,Solomon, S. S.**. Holiday gatherings, mobility and SARS-CoV-2 transmission: results from 10 US states following Thanksgiving. *Scientific Reports.* 2021. 11:17328

**Mikszewski, A., Stabile, L., Buonanno, G., Morawska, L.**. The vaccination threshold for SARS-CoV-2 depends on the indoor setting and room ventilation. *BMC Infectious Diseases.* 2021. 21:1193

**Miller, G. F.,Greening, B., Jr.,Rice, K. L.,Arifkhanova, A.,Meltzer, M. I.,Coronado, F.**. Modeling the Transmission of Covid-19: Impact of Mitigation Strategies in Prekindergarten-Grade 12 Public Schools, United States, 2021. *Journal of Public Health Management & Practice.* 2021. 30:30

**Min, J.**. Does social trust slow down or speed up the transmission of COVID-19?. *PLoS ONE.* 2020. 15(12 December) (no pagination):#pages#

**Mitze, T.,Kosfeld, R.**. The propagation effect of commuting to work in the spatial transmission of COVID-19. *Journal of Geographical Systems.* 2021. #volume#:1-27

**Mizumoto, K.,Kagaya, K.,Chowell, G.**. Effect of a wet market on coronavirus disease (COVID-19) transmission dynamics in China, 2019-2020. *International Journal of Infectious Diseases.* 2020. 97:96-101

**Mizumoto, K.,Kagaya, K.,Chowell, G.**. Early epidemiological assessment of the transmission potential and virulence of coronavirus disease 2019 (COVID-19) in Wuhan City, China, January-February, 2020. *BMC Medicine.* 2020. 18(1) (no pagination):#pages#

**Moghadas, S. M.,Fitzpatrick, M. C.,Sah, P.,Pandey, A.,Shoukat, A.,Singer, B. H.,Galvani, A. P.**. The implications of silent transmission for the control of COVID-19 outbreaks. *Proceedings of the National Academy of Sciences of the United States of America.* 2020. 117:17513-17515

**Moghadas, S. M.,Fitzpatrick, M. C.,Shoukat, A.,Zhang, K.,Galvani, A. P.**. Simulated Identification of Silent COVID-19 Infections Among Children and Estimated Future Infection Rates With Vaccination. *JAMA Network Open.* 2021. 4:e217097

**Mokhtari, R.,Jahangir, M. H.**. The effect of occupant distribution on energy consumption and COVID-19 infection in buildings: A case study of university building. *Building & Environment.* 2021. 190:107561

**Moritz, S.,Gottschick, C.,Horn, J.,Popp, M.,Langer, S.,Klee, B.,Purschke, O.,Gekle, M.,Ihling, A.,Zimmermann, F. D. L.,Mikolajczyk, R.**. The risk of indoor sports and culture events for the transmission of COVID-19. *Nature communications.* 2021. 12:5096

**Morozova, O.,Li, Z. R.,Crawford, F. W.**. One year of modeling and forecasting COVID-19 transmission to support policymakers in Connecticut. *MedRxiv : the Preprint Server for Health Sciences.* 2021. 23:23

**Morozova, O.,Li, Z. R.,Crawford, F. W.**. One year of modeling and forecasting COVID-19 transmission to support policymakers in Connecticut. *Scientific Reports.* 2021. 11:20271

**Muller, K.,Muller, P. A.**. Mathematical modelling of the spread of COVID-19 on a university campus. *Infectious Disease Modelling.* 2021. 6:1025-1045

**Munday, J. D.,Jarvis, C. I.,Gimma, A.,Wong, K. L. M.,van Zandvoort, K.,Cmmid Covid- Working Group,Funk, S.,Edmunds, W. J.**. Estimating the impact of reopening schools on the reproduction number of SARS-CoV-2 in England, using weekly contact survey data. *BMC Medicine.* 2021. 19:233

**Munday, J. D.,Sherratt, K.,Meakin, S.,Endo, A.,Pearson, C. A. B.,Hellewell, J.,Abbott, S.,Bosse, N. I.,Cmmid Covid- Working Group,Atkins, K. E.,Wallinga, J.,Edmunds, W. J.,van Hoek, A. J.,Funk, S.**. Implications of the school-household network structure on SARS-CoV-2 transmission under school reopening strategies in England. *Nature communications.* 2021. 12:1942

**Mutlu, M.M., Aksoy, I.C., Alver, Y.**. Covid-19 transmission risk minimization at public transportation stops using differential evolution algorithm. *Eur J Trans Infrastructure Res.* 2021. 21:#pages#

**Nadal, M.,Lassel, L.,Denis, M.,Gibelin, A.,Fournier, S.,Menard, L.,Goulet, H.,Abdi, B.,Farthoukh, M.,Pialoux, G.**. Role of super-spreader phenomenon in a Covid-19 cluster among healthcare workers in a Primary Care Hospital. *Journal of Infection.* 2021. 82(5):e13-e15

**Naqvi, K.,Mubeen, S. M.,Ali Shah, S. M.**. Challenges in providing oral and dental health services in COVID-19 pandemic. *JPMA - Journal of the Pakistan Medical Association.* 2020. 70:S113-S117

**Nash, D.,Rane, M. S.,Chang, M.,Kulkarni, S. G.,You, W. X.,Zimba, R.,Berry, A.,Mirzayi, C.,Kochhar, S.,Maroko, A.,Robertson, M. M.,Westmoreland, D. A.,Parcesepe, A.,Grov, C.**. Recent sars-cov-2 seroconversion in a national prospective cohort of us adults. *Topics in Antiviral Medicine.* 2021. 29(1):246

**Nguyen, D.,Sarani, N.,Marshall, K. D.,Cannon, C. M.,Jacobsen, R. C.,Pirotte, A.,Pittenger, C.,Wong, E. K.,Dodson, N. P.,LaCapra, M.,Howe, K.**. CODE BLUE-19: Proposal to Mitigate COVID-19 Transmission in the Emergency Department for Out-of-hospital Cardiac Arrest. *The Western Journal of Emergency Medicine.* 2020. 21:71-77

**Nir-Paz, R.,Grotto, I.,Strolov, I.,Salmon, A.,Mandelboim, M.,Mendelson, E.,Regev-Yochay, G.**. Absence of in-flight transmission of SARS-CoV-2 likely due to use of face masks on board. *Journal of Travel Medicine.* 2020. 27:#pages#

**Noh, J. Y., Song, J. Y., Hyun, H. J., Yoon, J. G., Seong, H., Cheong, H. J., Yoon, S. Y., Yang, J. S., Lee, J. Y., Kim, W. J.**. Risk factors for SARS-CoV-2 transmission in non-household clusters. *Journal of Infection.* 2021. 83(2):e22-e24

**Normile, D.,Cohen, J.,Enserink, M.,Huihui, B.**. As normalcy returns, can China keep COVID-19 at bay? Infected travelers pose a continuing threat, but local coronavirus transmission still occurs as well. *Science.* 2020. 368(6486):18-19

**Novazzi, F.,Cassaniti, I.,Piralla, A.,Di Sabatino, A.,Bruno, R.,Baldanti, F.**. SARS-CoV-2 positivity in rectal swabs: implication for possible transmission. *Journal of Global Antimicrobial Resistance.* 2020. 22:754-755

**Nurunnabi, M.**. The preventive strategies of COVID-19 pandemic in Saudi Arabia. *Journal of Microbiology, Immunology and Infection.* 2021. 54(1):127-128

**Nembhard, M. D.,Burton, D. J.,Cohen, J. M.**. Ventilation use in nonmedical settings during COVID-19: Cleaning protocol, maintenance, and recommendations. *Toxicology & Industrial Health.* 2020. 36:644-653

**Nielsen, P. V.,Xu, C.**. Multiple airflow patterns in human microenvironment and the influence on short-distance airborne cross-infection - A review. *Indoor and Built Environment..* 2021. #volume#:#pages#

**Nikolai, L. A.,Meyer, C. G.,Kremsner, P. G.,Velavan, T. P.**. Asymptomatic SARS Coronavirus 2 infection: Invisible yet invincible. *International Journal of Infectious Diseases.* 2020. 100:112-116

**Noorimotlagh, Z.,Jaafarzadeh, N.,Martinez, S. S.,Mirzaee, S. A.**. A systematic review of possible airborne transmission of the COVID-19 virus (SARS-CoV-2) in the indoor air environment. *Environmental Research.* 2021. 193 (no pagination):#pages#

**Nova, N.**. Cross-Species Transmission of Coronaviruses in Humans and Domestic Mammals, What Are the Ecological Mechanisms Driving Transmission, Spillover, and Disease Emergence?. *Frontiers in Public Health.* 2021. 9:717941

**Nascimento, M. L. F.**. A multivariate analysis on spatiotemporal evolution of Covid-19 in Brazil. *Infectious Disease Modelling.* 2020. 5:670-680

**Ng, V.,Fazil, A.,Waddell, L. A.,Bancej, C.,Turgeon, P.,Otten, A.,Atchessi, N.,Ogden, N. H.**. Projected effects of nonpharmaceutical public health interventions to prevent resurgence of SARS-CoV-2 transmission in Canada. *CMAJ Canadian Medical Association Journal.* 2020. 192:E1053-E1064

**Nguyen, L. K. N.,Howick, S.,McLafferty, D.,Anderson, G. H.,Pravinkumar, S. J.,Van Der Meer, R.,Megiddo, I.**. Evaluating intervention strategies in controlling coronavirus disease 2019 (COVID-19) spread in care homes: An agent-based model. *Infection Control & Hospital Epidemiology.* 2021. 42:1060-1070

**Nguyen, L. K. N.,Howick, S.,McLafferty, D.,Anderson, G. H.,Pravinkumar, S. J.,Van Der Meer, R.,Megiddo, I.**. Evaluating intervention strategies in controlling COVID-19 spread in care homes: An agent-based model. *Infection Control and Hospital Epidemiology..* 2020. #volume#:#pages#

**Niehus, R.,De Salazar, P. M.,Taylor, A. R.,Lipsitch, M.**. Using observational data to quantify bias of traveller-derived COVID-19 prevalence estimates in Wuhan, China. *The Lancet Infectious Diseases.* 2020. 20:803-808

**Niu, B.,Liang, R.,Zhang, S.,Zhang, H.,Qu, X.,Su, Q.,Zheng, L.,Chen, Q.**. Epidemic analysis of COVID-19 in Italy based on spatiotemporal geographic information and Google Trends. *Transboundary & Emerging Diseases.* 2021. 68:2384-2400

**Notari, A., Torrieri, G.**. COVID-19 transmission risk factors. *Pathogens and Global Health.* 2021. #volume#:1-32

**Ntounis, N.,Mumford, C.,Lorono-Leturiondo, M.,Parker, C.,Still, K.**. How safe is it to shop? Estimating the amount of space needed to safely social distance in various retail environments. *Safety Science.* 2020. 132:104985

**Ohia, C.,Salawu, M. M.**. COVID-19 pandemic and civil unrests in Africa: implication of recent #EndSARS protests for increased community transmission in Nigeria. *The Pan African medical journal.* 2020. 37:47

**Oliver, D.**. David Oliver: Heed HSIB on covid transmission in hospital. *The BMJ.* 2020. 371 (no pagination):#pages#

**Orenes-Pinero, E.,Bano, F.,Navas-Carrillo, D.,Moreno-Docon, A.,Marin, J. M.,Misiego, R.,Ramirez, P.**. Evidences of SARS-CoV-2 virus air transmission indoors using several untouched surfaces: A pilot study. *Science of the Total Environment.* 2021. 751 (no pagination):#pages#

**Orouba, Almilaji, Peter, Thomas**. Air recirculation role in the infection with COVID-19, lessons learned from Diamond Princess cruise ship. *#journal#.* 2020. #volume#:#pages#

**O'Donoghue, A.,Dechen, T.,Pavlova, W.,Boals, M.,Moussa, G.,Madan, M.,Thakkar, A.,DeFalco, F. J.,Stevens, J. P.**. Reopening businesses and risk of COVID-19 transmission. *Npj Digital Medicine.* 2021. 4:51

**O'Driscoll, M.,Harry, C.,Donnelly, C. A.,Cori, A.,Dorigatti, I.**. A Comparative Analysis of Statistical Methods to Estimate the Reproduction Number in Emerging Epidemics, With Implications for the Current Coronavirus Disease 2019 (COVID-19) Pandemic. *Clinical Infectious Diseases.* 2021. 73:e215-e223

**O'Driscoll, M.,Ribeiro Dos Santos, G.,Wang, L.,Cummings, D. A. T.,Azman, A. S.,Paireau, J.,Fontanet, A.,Cauchemez, S.,Salje, H.**. Age-specific mortality and immunity patterns of SARS-CoV-2. *Nature.* 2021. 590(7844):140-145

**Olanrewaju, A.,AbdulAziz, A.,Preece, C. N.,Shobowale, K.**. Evaluation of measures to prevent the spread of COVID-19 on the construction sites. *Cleaner Engineering and Technology.* 2021. 5:100277

**Ooi, C. C.,Suwardi, A.,Ou Yang, Z. L.,Xu, G.,Tan, C. K. I.,Daniel, D.,Li, H.,Ge, Z.,Leong, F. Y.,Marimuthu, K.,Ng, O. T.,Lim, S. B.,Lim, P.,Mak, W. S.,Cheong, W. C. D.,Loh, X. J.,Kang, C. W.,Lim, K. H.**. Risk assessment of airborne COVID-19 exposure in social settings. *Physics of Fluids.* 2021. 33:087118

**Ozdenerol, E.,Seboly, J.**. Lifestyle Effects on the Risk of Transmission of COVID-19 in the United States: Evaluation of Market Segmentation Systems. *International Journal of Environmental Research & Public Health [Electronic Resource].* 2021. 18:30

**Pan, D., Sze, S., Martin, C. A., Nazareth, J., Woolf, K., Baggaley, R. F., Hollingsworth, T. D., Khunti, K., Nellums, L. B., Pareek, M.**. Covid-19 and ethnicity: We must seek to understand the drivers of higher transmission. *The BMJ.* 2021. 375 (no pagination):#pages#

**Pandit, J. J.**. Correct probability estimates for the risk of an anaesthetist contracting the SARS-CoV-2 virus after aerosol-generating procedures. *Anaesthesia and Intensive Care.* 2021. 49(1):77

**Pecho-Silva, S.,Arteaga-Livias, K.,Rodriguez-Morales, A. J.**. Airborne SARS-CoV-2: Weighing the evidence for its role in community transmission. *Journal of Preventive Medicine and Public Health.* 2020. 53(3):178-179

**Phan, L. T.,Luong, Q. C.,Nguyen, T. V.,Nguyen, H. T.,Le, H. Q.,Nguyen, T. T.,Cao, T. M.,Pham, Q. D.**. Importation and human-to-human transmission of a novel coronavirus in Vietnam. *New England Journal of Medicine.* 2020. 382(9):872-874

**Philip, K. E. J.,Lewis, A.,Buttery, S. C.,McCabe, C.,Fancourt, D.,Orton, C. M.,Polkey, M. I.,Hopkinson, N. S.**. Aerosol transmission of SARS-CoV-2: Inhalation as well as exhalation matters for COVID-19. *American Journal of Respiratory and Critical Care Medicine.* 2021. 203(8):1041-1042

**Plaat, F.,Campbell, J. P.**. Is spinal anaesthesia an aerosol-generating procedure? Transmission of SARS-CoV-2 from patient to anaesthetist. *British Journal of Anaesthesia.* 2020. 125(3):e315

**Pocock, K.,Close, R. M.,McAuley, J.**. The porous boundaries between communities and correctional facilities: The introduction of a medical recovery site resulting in reduced COVID-19 household transmission tied to recently incarcerated individuals. *Open Forum Infectious Diseases.* 2020. 7(SUPPL 1):S308-S309

**Pollack, R. J.**. Discussion: Community evidence of severe acute respiratory syndrome coronavirus 2 (SARS-CoV-2) transmission through air. Atmospheric Environment 2020, 118083. *Atmospheric Environment.* 2021. 254 (no pagination):#pages#

**Pombal, R.,Hosegood, I.,Powell, D.**. Risk of COVID-19 during Air Travel. *JAMA - Journal of the American Medical Association.* 2020. 324(17):1798

**Poole, S.,Brendish, N. J.,Tanner, A. R.,Clark, T. W.**. Physical distancing in schools for SARS-CoV-2 and the resurgence of rhinovirus. *The Lancet Respiratory Medicine.* 2020. 8(12):e92-e93

**Pourkarim, M. R.,Thijssen, M.,Lemey, P.,Vandamme, A. M.,Van Ranst, M.**. Air conditioning system usage and SARS-CoV-2 transmission dynamics in Iran. *Medical Hypotheses.* 2020. 143 (no pagination):#pages#

**Prather, K. A.,Wang, C. C.,Schooley, R. T.**. Reducing transmission of SARS-CoV-2: Masks and testing are necessary to combat asymptomatic spread in aerosols and droplets. *Science.* 2020. 368(6498):1422-1424

**Propper, R. E.**. Is sweat a possible route of transmission of SARS-CoV-2?. *Experimental Biology and Medicine.* 2020. 245(12):997-998

**Pan, J.,Bardhan, R.,Jin, Y.**. Spatial distributive effects of public green space and COVID-19 infection in London. *Urban Forestry & Urban Greening.* 2021. 62:127182

**Pan, Y.,Liu, H.,Chu, C.,Li, X.,Liu, S.,Lu, S.**. Transmission routes of SARS-CoV-2 and protective measures in dental clinics during the COVID-19 pandemic. *American Journal of Dentistry.* 2020. 33:129-134

**Pang, J. K.,Jones, S. P.,Waite, L. L.,Olson, N. A.,Armstrong, J. W.,Atmur, R. J.,Cummins, J. J.**. Probability and estimated risk of SARS-CoV-2 transmission in the air travel system. *Travel Medicine & Infectious Disease.* 2021. 43:102133

**Park, O.,Park, Y. J.,Park, S. Y.,Kim, Y. M.,Lee, J.,Park, E.,Kim, D.,Jeon, B. H.,Ryu, B.,Ko, D.,Kim, E.,Kim, H.,Lee, H.,Gwack, J.,Jo, J.,Lee, J. H.,Hyun, J.,Kim, J.,Park, J. K.,Lee, S.,Kim, S. S.,Shin, S. H.,Choi, S. W.,Kim, T.,Kim, U. N.,Woo, Y.,Jin, Y.,Jang, Y. S.,Park, Y.,Yum, M.**. Contact transmission of Covid-19 in South Korea: Novel investigation techniques for tracing contacts. *Osong Public Health and Research Perspectives.* 2020. #volume#:60-63

**Parker, J.,Boles, C.,Leleck, O.,Buerger, A.,Egnot, N.,Sundermann, A.,Bussmann, E.**. Advancing toward normal operations for arenas and stadiums. *Toxicology and Industrial Health.* 2020. 36(9):718-727

**Pasteur, Institut, Guyane, Institut Pasteur de la, Cayenne, Centre Hospitalier Andrée Rosemon de, Guadeloupe, Institut Pasteur de la, Guadeloupe, Centre Hospitalier de la, Nouvelle-Calédonie, Institut Pasteur de, Nouvelle-Calédonie, Centre Hospitalier Territorial de**. Household Transmission Investigation Study for COVID-19 in Tropical Regions. *#journal#.* 2020. #volume#:#pages#

**Pearce, L. A.,Vaisey, A.,Keen, C.,Calais-Ferreira, L.,Foulds, J. A.,Young, J. T.,Southalan, L.,Borschmann, R.,Gray, R.,Sturup-Toft, S.,Kinner, S. A.**. A rapid review of early guidance to prevent and control COVID-19 in custodial settings. *Health & Justice.* 2021. 9:27

**Peng, Z., Pineda Rojas, A. L., Kropff, E., Bahnfleth, W., Buonanno, G., Dancer, S. J., Kurnitski, J., Li, Y., Loomans, Mglc, Marr, L. C., Morawska, L., Nazaroff, W., Noakes, C., Querol, X., Sekhar, C., Tellier, R., Greenhalgh, T., Bourouiba, L., Boerstra, A., Tang, J. W., Miller, S. L., Jimenez, J. L.**. Correction to Practical Indicators for Risk of Airborne Transmission in Shared Indoor Environments and Their Application to COVID-19 Outbreaks. *Environmental Science & Technology.* 2022. 14:14

**Przekwas, A.,Chen, Z.**. Washing hands and the face may reduce COVID-19 infection. *Medical Hypotheses.* 2020. 144 (no pagination):#pages#

**Paltiel, A. D.,Schwartz, J. L.**. Assessing COVID-19 Prevention Strategies to Permit the Safe Opening of Residential Colleges in Fall 2021. *Annals of internal medicine..* 2021. 31:#pages#

**Pang, S.,Xiao, J.,Fang, Y.**. Risk assessment model and application of COVID-19 virus transmission in closed environments at sea. *Sustainable Cities and Society.* 2021. 74:103245

**Parajuli, R. R.,Mishra, B.,Banstola, A.,Ghimire, B. R.,Poudel, S.,Sharma, K.,Dixit, S. M.,Sah, S. K.,Simkhada, P.,van Teijlingen, E.**. Multidisciplinary approach to COVID-19 risk communication: a framework and tool for individual and regional risk assessment. *Scientific Reports.* 2020. 10:21650

**Parhizkar, H., Van Den Wymelenberg, K. G., Haas, C. N., Corsi, R. L.**. A Quantitative Risk Estimation Platform for Indoor Aerosol Transmission of COVID-19. *Risk Analysis.* 2021. 28:28

**Park, S. W.,Cornforth, D. M.,Dushoff, J.,Weitz, J. S.**. The time scale of asymptomatic transmission affects estimates of epidemic potential in the COVID-19 outbreak. *Epidemics.* 2020. 31 (no pagination):#pages#

**Park, S.,Choi, Y.,Song, D.,Kim, E. K.**. Natural ventilation strategy and related issues to prevent coronavirus disease 2019 (COVID-19) airborne transmission in a school building. *Science of the Total Environment.* 2021. 789 (no pagination):#pages#

**Parsons, T. L.,Worden, L.**. Assessing the Risk of Cascading COVID-19 Outbreaks from Prison-to-Prison Transfers. *MedRxiv : the Preprint Server for Health Sciences.* 2021. 27:27

**Pasaribu, U. S.,Mukhaiyar, U.,Huda, N. M.,Sari, K. N.,Indratno, S. W.**. Modelling COVID-19 growth cases of provinces in java Island by modified spatial weight matrix GSTAR through railroad passenger's mobility. *Heliyon.* 2021. 7:e06025

**Pasco, R. F.,Fox, S. J.,Johnston, S. C.,Pignone, M.,Meyers, L. A.**. Estimated Association of Construction Work With Risks of COVID-19 Infection and Hospitalization in Texas. *JAMA Network Open.* 2020. 3:e2026373

**Pathela, P.,Crawley, A.,Weiss, D.,Maldin, B.,Cornell, J.,Purdin, J.,Schumacher, P. K.,Marovich, S.,Li, J.,Daskalakis, D.,N. Y. C. Serosurvey Team**. Seroprevalence of Severe Acute Respiratory Syndrome Coronavirus 2 Following the Largest Initial Epidemic Wave in the United States: Findings From New York City, 13 May to 21 July 2020. *Journal of Infectious Diseases.* 2021. 224:196-206

**Pavilonis, B.,Ierardi, A. M.,Levine, L.,Mirer, F.,Kelvin, E. A.**. Estimating aerosol transmission risk of SARS-CoV-2 in New York City public schools during reopening. *Environmental Research.* 2021. 195:110805

**Peng, Z., Rojas, A. L. P., Kropff, E., Bahnfleth, W., Buonanno, G., Dancer, S. J., Kurnitski, J., Li, Y., Loomans, Mglc, Marr, L. C., Morawska, L., Nazaroff, W., Noakes, C., Querol, X., Sekhar, C., Tellier, R., Greenhalgh, T., Bourouiba, L., Boerstra, A., Tang, J. W., Miller, S. L., Jimenez, J. L.**. Practical Indicators for Risk of Airborne Transmission in Shared Indoor Environments and Their Application to COVID-19 Outbreaks. *Environmental Science & Technology.* 2022. 56:1125-1137

**Perez-Segura, V.,Caro-Carretero, R.,Rua, A.**. Multivariate Analysis of Risk Factors of the COVID-19 Pandemic in the Community of Madrid, Spain. *International Journal of Environmental Research & Public Health [Electronic Resource].* 2021. 18:01

**Pham, Q. D.,Stuart, R. M.,Nguyen, T. V.,Luong, Q. C.,Tran, Q. D.,Pham, T. Q.,Phan, L. T.,Dang, T. Q.,Tran, D. N.,Do, H. T.,Mistry, D.,Klein, D. J.,Abeysuriya, R. G.,Oron, A. P.,Kerr, C. C.**. Estimating and mitigating the risk of COVID-19 epidemic rebound associated with reopening of international borders in Vietnam: a modelling study. *The Lancet Global Health.* 2021. 9:e916-e924

**Popa, A.,Genger, J. W.,Nicholson, M. D.,Penz, T.,Schmid, D.,Aberle, S. W.,Agerer, B.,Lercher, A.,Endler, L.,Colaco, H.,Smyth, M.,Schuster, M.,Grau, M. L.,Martinez-Jimenez, F.,Pich, O.,Borena, W.,Pawelka, E.,Keszei, Z.,Senekowitsch, M.,Laine, J.,Aberle, J. H.,Redlberger-Fritz, M.,Karolyi, M.,Zoufaly, A.,Maritschnik, S.,Borkovec, M.,Hufnagl, P.,Nairz, M.,Weiss, G.,Wolfinger, M. T.,von Laer, D.,Superti-Furga, G.,Lopez-Bigas, N.,Puchhammer-Stockl, E.,Allerberger, F.,Michor, F.,Bock, C.,Bergthaler, A.**. Genomic epidemiology of superspreading events in Austria reveals mutational dynamics and transmission properties of SARS-CoV-2. *Science Translational Medicine.* 2020. 12:09

**Post, L. A.,Benishay, E. T.,Moss, C. B.,Murphy, R. L.,Achenbach, C. J.,Ison, M. G.,Resnick, D.,Singh, L. N.,White, J.,Chaudhury, A. S.,Boctor, M. J.,Welch, S. B.,Oehmke, J. F.**. Surveillance metrics of SARS-CoV-2 transmission in Central Asia: Longitudinal trend analysis. *Journal of Medical Internet Research.* 2021. 23(2) (no pagination):#pages#

**Pozderac, C.,Skinner, B.**. Superspreading of SARS-CoV-2 in the USA. *PLoS ONE [Electronic Resource].* 2021. 16:e0248808

**Procter, S. R.,Abbas, K.,Flasche, S.,Griffiths, U.,Hagedorn, B.,O'Reilly, K. M.,Waterlow, N. R.,Villabona-Arenas, C. J.,Munday, J. D.,Medley, G. F.,Lowe, R.,Mee, P.,Liu, Y.,Gimma, A.,van Zandvoort, K.,Hellewell, J.,Tully, D. C.,Brady, O.,Auzenbergs, M.,Knight, G. M.,Kucharski, A. J.,Barnard, R. C.,Waites, W.,Edmunds, W. J.,Bosse, N. I.,Endo, A.,Finch, E.,Russell, T. W.,Chan, Y. W. D.,Quaife, M.,Eggo, R. M.,Prem, K.,Pung, R.,Jombart, T.,Quilty, B. J.,Clifford, S.,Koltai, M.,Gibbs, H. P.,Abbott, S.,Jarvis, C. I.,Jafari, Y.,Klepac, P.,Krauer, F.,Sun, F. Y.,Funk, S.,Sandmann, F. G.,Nightingale, E. S.,Lei, J.,Meakin, S. R.,Rosello, A.,Pearson, C. A. B.,Hodgson, D.,McCarthy, C. V.,Foss, A. M.,Atkins, K. E.,Jit, M.**. SARS-CoV-2 infection risk during delivery of childhood vaccination campaigns: a modelling study. *BMC Medicine.* 2021. 19(1) (no pagination):#pages#

**Purkayastha, S.,Bhattacharyya, R.,Bhaduri, R.,Kundu, R.,Gu, X.,Salvatore, M.,Ray, D.,Mishra, S.,Mukherjee, B.**. A comparison of five epidemiological models for transmission of SARS-CoV-2 in India. *BMC Infectious Diseases.* 2021. 21(1) (no pagination):#pages#

**Qian, G.,Yang, N.,Ma, A. H. Y.,Wang, L.,Li, G.,Chen, X.,Chen, X.**. COVID-19 Transmission Within a Family Cluster by Presymptomatic Carriers in China. *Clinical Infectious Diseases.* 2020. 71:861-862

**Qing, H.,Li, Z.,Yang, Z.,Shi, M.,Huang, Z.,Song, J.,Song, Z.**. The possibility of COVID-19 transmission from eye to nose. *Acta Ophthalmologica.* 2020. 98(3):e388

**Qiu, S.,Li, P.,Jia, H.,Du, X.,Liu, H.,Wang, H.,Yang, M.,Wang, L.,Song, H.**. Familial cluster of SARS-CoV-2 infection associated with a railway journey. *Journal of Travel Medicine.* 2020. 27(5) (no pagination):#pages#

**Quental, K. N.,Leite, A. L.,Feitosa, A. D. N. A.,Oliveira, Z. N. P. D.,Tavares, L. V. D. S.,Tavares, W. G. D. S.,Pinheiro, E. F.,Lacsina, J. R.,DeSouza-Vieira, T.,Silva, J. B. N. F.**. SARS-CoV-2 co-infection with dengue virus in Brazil: A potential case of viral transmission by a health care provider to household members. *Travel Medicine and Infectious Disease.* 2021. 40 (no pagination):#pages#

**Quraishi, S. A.,Berra, L.,Nozari, A.**. Indoor temperature and relative humidity in hospitals: Workplace considerations during the novel coronavirus pandemic. *Occupational and Environmental Medicine.* 2020. 77(7):508

**Qi, B.,Tan, J.,Zhang, Q.,Cao, M.,Wang, X.,Zou, Y.**. Unfixed movement route model, non-overcrowding and social distancing reduce the spread of covid-19 in sporting facilities. *International Journal of Environmental Research and Public Health.* 2021. 18(15) (no pagination):#pages#

**Qifang, Bi, Justin, Lessler, Isabella, Eckerle, Stephen, A. Lauer, Laurent, Kaiser, Nicolas, Vuilleumier, Derek, A. T. Cummings, Antoine, Flahault, Dusan, Petrovic, Idris, Guessous, Silvia, Stringhini, Andrew, S. Azman, SEROCoV-POP**. Household Transmission of SARS-COV-2: Insights from a Population-based Serological Survey. *#journal#.* 2020. #volume#:#pages#

**Quaranta, G.,Formica, G.,Machado, J. T.,Lacarbonara, W.,Masri, S. F.**. Understanding COVID-19 nonlinear multi-scale dynamic spreading in Italy. *Nonlinear Dynamics.* 2020. #volume#:1-37

**Rahim, A.,Maqbool, A.,Rana, T.**. Monitoring social distancing under various low light conditions with deep learning and a single motionless time of flight camera. *PLoS ONE.* 2021. 16(2 February) (no pagination):#pages#

**Ramesh, N.,Siddaiah, A.,Joseph, B.**. Tackling corona virus disease 2019 (COVID 19) in workplaces. *Indian Journal of Occupational and Environmental Medicine.* 2020. 24(1):16-18

**Ramirez, J. D.,Florez, C.,Munoz, M.,Hernandez, C.,Castillo, A.,Gomez, S.,Rico, A.,Pardo, L.,Barros, E. C.,Castaneda, S.,Ballesteros, N.,Martinez, D.,Vega, L.,Jaimes, J. E.,Cruz-Saavedra, L.,Herrera, G.,Patino, L. H.,Teheran, A. A.,Gonzalez-Reiche, A. S.,Hernandez, M. M.,Sordillo, E. M.,Simon, V.,van Bakel, H.,Paniz-Mondolfi, A.**. The arrival and spread of SARS-CoV-2 in Colombia. *Journal of Medical Virology.* 2021. 93:1158-1163

**Ramya, G.,Guru, C. S.,Banodhe, G. K.,Dominic, D.,Sharma, H. B.**. Resumption to swimming post COVID 19 lockdown. *Journal of Clinical and Diagnostic Research.* 2021. 15(1):CE01-CE05

**Rezaei, M.,Netz, R. R.**. Airborne virus transmission via respiratory droplets: Effects of droplet evaporation and sedimentation. *Current Opinion in Colloid and Interface Science.* 2021. 55 (no pagination):#pages#

**Rochwerg, B.,Schunemann, H. J.,Akl, E. A.**. Ventilation Techniques and Risk for Transmission of Coronavirus Disease, Including COVID-19. *Annals of internal medicine.* 2020. 173(10):860

**Rosca, E. C.,Heneghan, C.,Spencer, E. A.,Brassey, J.,Pluddemann, A.,Onakpoya, I. J.,Evans, D. H.,Conly, J. M.,Jefferson, T.**. Transmission of SARS-CoV-2 associated with aircraft travel: A systematic review. *Journal of Travel Medicine.* 2021. 03:03

**Rasmussen, S.,Petersen, M. S.,Hoiby, N.**. SARS-CoV-2 infection dynamics in Denmark, February through October 2020: Nature of the past epidemic and how it may develop in the future. *PLoS ONE.* 2021. 16(4 April) (no pagination):#pages#

**Ren, C.,Xi, C.,Wang, J.,Feng, Z.,Nasiri, F.,Cao, S. J.,Haghighat, F.**. Mitigating COVID-19 infection disease transmission in indoor environment using physical barriers. *Sustainable Cities and Society.* 2021. 74:103175

**Reynolds, C.,Ng, S.,Yang, W.**. Factors affecting the transmission of SARS-CoV-2 in school settings. *MedRxiv : the Preprint Server for Health Sciences.* 2021. 22:22

**Risbeck, M. J.,Bazant, M. Z.,Jiang, Z.,Lee, Y. M.,Drees, K. H.,Douglas, J. D.**. Modeling and Multiobjective Optimization of Indoor Airborne Disease Transmission Risk and Associated Energy Consumption for Building HVAC Systems. *Energy & Buildings.* 2021. #volume#:111497

**Rocha-Melogno, L., Crank, K., Bergin, M. H., Gray, G. C., Bibby, K., Deshusses, M. A.**. Quantitative risk assessment of COVID-19 aerosol transmission indoors: a mechanistic stochastic web application. *Environmental Technology.* 2021. #volume#:1-12

**Rotejanaprasert, C.,Lawpoolsri, S.,Pan-Ngum, W.,Maude, R. J.**. Preliminary estimation of temporal and spatiotemporal dynamic measures of COVID- 19 transmission in Thailand. *PLoS ONE.* 2020. 15(9 September) (no pagination):#pages#

**Rufach, D.,Santos, S.,Terebiznik, M.**. Simulation of pediatric intubation using a low-cost videolaryngoscope in the setting of the COVID-19 pandemic. *Archivos Argentinos de Pediatria.* 2021. 119:270-272

**Rui, R.,Tian, M.,Tang, M. L.,Ho, G. T.,Wu, C. H.**. Analysis of the Spread of COVID-19 in the USA with a Spatio-Temporal Multivariate Time Series Model. *International Journal of Environmental Research & Public Health [Electronic Resource].* 2021. 18:18

**Russell, T. W.,Wu, J. T.,Clifford, S.,Edmunds, W. J.,Kucharski, A. J.,Jit, M.**. Effect of internationally imported cases on internal spread of COVID-19: a mathematical modelling study. *The Lancet Public Health.* 2021. 6(1):e12-e20

**Rypdal, M.,Rypdal, V.,Jakobsen, P. K.,Ytterstad, E.,Lovsletten, O.,Klingenberg, C.,Rypdal, K.**. Modelling suggests limited change in the reproduction number from reopening norwegian kindergartens and schools during the COVID-19 pandemic. *PLoS ONE.* 2021. 16(2 February) (no pagination):#pages#

**Ran, J.,Zhao, S.,Han, L.,Qiu, Y.,Cao, P.,Yang, Z.,Chong, M. K. C.,Yang, L.,Wang, M. H.,He, D.**. Effects of particulate matter exposure on the transmissibility and case fatality rate of COVID-19: A nationwide ecological study in China. *Journal of Travel Medicine.* 2020. 27(6):1-4

**Rankin, D. A.,Howard, L.,Fernandez, K. N.,Talj, R.,Haddadin, Z.,Spieker, A. J.,Halasa, N. B.**. Outbreak of COVID-19 among school auction attendees: Was it a "silent auction" or "silent transmission"?. *Open Forum Infectious Diseases.* 2020. 7(SUPPL 1):S306-S307

**Ranshing, S.,Lavania, M.,Potdar, V.,Patwardhan, S.,Prayag, P. S.,Jog, S.,Kelkar, D.,Sawant, P.,Shinde, M.,Chavan, N.**. Transmission of COVID-19 infection within a family cluster in Pune, India. *Indian Journal of Medical Research.* 2021. 19:19

**Rao, Us V.,Arakeri, G.,Subash, A.,Thakur, S.**. Droplet nuclei aerosol and Covid 19 - a risk to healthcare staff. *British Journal of Oral and Maxillofacial Surgery.* 2020. 58(7):870-871

**Ritwik, P.,Patterson, K. K.,Alfonzo-Echeverri, E.**. What Is Best for the Child? Pediatric Dental Care during COVID-19. *Journal of Clinical Ethics.* 2021. 32:215-223

**Rivory, J.,Beaugendre, E.,Yvon, C.,Ploteau, F.,Condat, B.,Rostain, F.,Pioche, M.**. Why not use the Easybreath snorkeling mask to prevent COVID-19 transmission during endoscopy procedures when FFP2 are lacking?. *Endoscopy.* 2020. 52(8):E306-E307

**Rocha, I. C. N.,Pelayo, M. G. A.,Rackimuthu, S.**. Kumbh Mela Religious Gathering as a Massive Superspreading Event: Potential Culprit for the Exponential Surge of COVID-19 Cases in India. *American Journal of Tropical Medicine & Hygiene.* 2021. 30:30

**Rochwerg, B.,Solo, K.,Darzi, A.,Chen, G.,Khamis, A. M.**. Update Alert: Ventilation Techniques and Risk for Transmission of Coronavirus Disease, Including COVID-19. *Annals of internal medicine.* 2020. 173(6):W122

**Ross, K. A.,Almuzam, S.,Britton, P. N.,Howard-Jones, A. R.,Isaacs, D.,Kesson, A.,Nayda, C.**. What risk do aerosol-generating procedures pose to health-care workers?. *Journal of Paediatrics and Child Health.* 2020. 56(10):1639-1640

**Rowland, T. A. J.,Whitaker, H.,Jeffery-Smith, A.,Lang, N.,Sendall, K.,McLaren, R.,Brown, K. E.,Ramsay, M.,Ladhani, S. N.,Zambon, M.**. Seropositivity and risk factors for SARS-CoV-2 infection in staff working in care homes during the COVID-19 pandemic. *Journal of Infection.* 2021. 82(4):84-123

**Ruiz-Hornillos, J.,Kilimajer Astudillo, J.,Seoane-Rodriguez, M.,Henriquez-Santana, A.**. Risk of SARS-CoV-2 exposure in a hospital-based allergy practice. *Clinical & Experimental Allergy.* 2021. 51:141-143

**Ruiz-Lozano, R. E.,Cardenas-De La Garza, J. A.,Ibarra-Nava, I.,Garza-Garza, L. A.,Hernandez-Camarena, J. C.**. The role of football as a super-spreading event in the SARS-CoV-2 pandemic. *Journal of Sports Medicine & Physical Fitness.* 2020. 60:1408-1409

**Safdar, N.,Moreno, G. K.,Braun, K. M.,Friedrich, T. C.,O'Connor, D. H.**. Using virus sequencing to determine source of SARS-CoV-2 transmission for healthcare worker. *Emerging Infectious Diseases.* 2020. 26(10):2489-2491

**Saki, M.,Haseli, S.,Iranpour, P.**. Oral Radiology Center as a Potential Source of COVID-19 Transmission; Points to Consider. *Academic Radiology.* 2020. 27(7):1047-1048

**Sasaki, S.,Nishikawa, J.,Sakaida, I.**. Use of a glove-covered mouthpiece during upper endoscopy to prevent COVID-19 transmission. *Clinical Endoscopy.* 2021. 54(2):289-290

**Sassano, M.,McKee, M.,Ricciardi, W.,Boccia, S.**. Transmission of SARS-CoV-2 and Other Infections at Large Sports Gatherings: A Surprising Gap in Our Knowledge. *Frontiers in Medicine.* 2020. 7 (no pagination):#pages#

**Schiavone, M.,Gasperetti, A.,Mitacchione, G.,Viecca, M.,Forleo, G. B.**. Response to: COVID-19 re-infection. Vaccinated individuals as a potential source of transmission. *European Journal of Clinical Investigation.* 2021. 51:e13544

**Schunemann, H. J.,Akl, E. A.,Chou, R.,Chu, D. K.,Loeb, M.,Lotfi, T.,Mustafa, R. A.,Neumann, I.,Saxinger, L.,Sultan, S.,Mertz, D.**. Use of facemasks during the COVID-19 pandemic. *The Lancet Respiratory Medicine.* 2020. 8(10):954-955

**Schwartz, K. L.,Murti, M.,Finkelstein, M.,Leis, J. A.,Fitzgerald-Husek, A.,Bourns, L.,Meghani, H.,Saunders, A.,Allen, V.,Yaffe, B.**. Lack of COVID-19 transmission on an international flight. *Cmaj.* 2020. 192(15):E410

**Schwendicke, F.**. Saliva is a potential source of Covid-19, and appropriate protection measures should be applied in dental practice. *Evidence-based dentistry.* 2020. 21(2):62

**Shim, M. J.,Kang, Y. J.**. COVID-19 in South Korea: Focusing on club infections. *Journal of Pure and Applied Microbiology.* 2020. 14(4):2299-2302

**Shoib, S.,Nagendrappa, S.,Grigo, O.,Rehman, S.,Ransing, R.**. Factors associated with COVID-19 outbreak-related suicides in India. *Asian Journal of Psychiatry.* 2020. 53 (no pagination):#pages#

**Shrivastava, S. R.,Shrivastava, P. S.**. Measures to contain the coronavirus disease 2019 pandemic in workplace settings: Public health perspective. *JMS - Journal of Medical Society.* 2020. 34(2):117-118

**Siddiqui, R.,Khamis, M.,Ibrahim, T.,Khan, N. A.**. Irrigation System and COVID-19 Recurrence: A Potential Risk Factor in the Transmission of SARS-CoV-2. *Acs Chemical Neuroscience.* 2020. 11:2903-2905

**Signorelli, C.,Odone, A.,Ricco, M.,Bellini, L.,Croci, R.,Oradini-Alacreu, A.,Fiacchini, D.,Burioni, R.**. Major sports events and the transmission of SARS-CoV-2: analysis of seven case-studies in Europe. *Acta Bio-Medica de l Ateneo Parmense.* 2020. 91:242-244

**Singh, H.,Singh, J.,Khubaib, M.,Jamal, S.,Sheikh, J.,Kohli, S.,Hasnain, S.,Rahman, S.**. Mapping the genomic landscape & diversity of COVID-19 based on >3950 clinical isolates of SARS-CoV-2: Likely origin & transmission dynamics of isolates sequenced in India. *Indian Journal of Medical Research.* 2020. 151(5):474-478

**Siqueira-Batista, R.,Motta, O. J. R. D.,Braga, L. M.,Gomes, A. P.**. Airborne Transmission of Severe Acute Respiratory Syndrome Coronavirus 2 and Protection of Health Care Professionals. *Infectious Diseases in Clinical Practice.* 2020. 28(5):e16

**Slifka, M. K.,Messer, W. B.,Amanna, I. J.**. Analysis of COVID-19 Transmission: Low Risk of Presymptomatic Spread?. *Archives of Pathology & Laboratory Medicine.* 2020. 144:1161-1162

**Smiley, R.**. Spinal anaesthesia and COVID-19 transmission to anaesthetists. Comment on Br J Anaesth 2020; 124: 670-5. *British Journal of Anaesthesia.* 2020. 125(2):e247-e248

**Smith-Norowitz, T. A.,Norowitz, Y. M.,Kohlhoff, S.,Hammerschlag, M. R.**. SARS-CoV-2 Positivity rates are lower in school compared with local rates in Brooklyn, New York. *Acta Paediatrica, International Journal of Paediatrics.* 2021. 110(9):2576-2577

**Soe, W. M. A. R.,Balakrishnan, A.,Adhiyaman, V.**. Nosocomial COVID-19 on a green ward. *Clinical Medicine, Journal of the Royal College of Physicians of London.* 2020. 20(6):E282

**Soleimani, M.,Merajikhah, A.,Beigi-Khoozani, A.**. The risk of transmitting the coronavirus to the perioperative team through aerosols produced in the operating room bathrooms. *Perioperative Care and Operating Room Management.* 2021. 24:100179

**Sollena, P.,Cappilli, S.,Piccerillo, A.,Chiricozzi, A.,Peris, K.**. COVID-19 hygiene measures: hand eczema and insights into ACE2 and integrins as key molecules for SARS-CoV-2 cutaneous transmission. *International Journal of Dermatology.* 2020. 59(11):1409-1410

**Sorbello, J. G.**. Ventilation Techniques and Risk for Transmission of Coronavirus Disease, Including COVID-19. *Annals of internal medicine.* 2020. 173(10):860

**Soriano, J. B.,Anzueto, A.,Anticevich, S. B.,Kaplan, A.,Miravitlles, M.,Usmani, O.,Papadopoulos, N. G.,Puggioni, F.,Walter Canonica, G.,Roche, N.**. Face masks, respiratory patients and COVID-19. *European Respiratory Journal.* 2020. 56(5) (no pagination):#pages#

**Spinelli, M. A., Glidden, D. V., Gennatas, E. D., Bielecki, M., Beyrer, C., Rutherford, G., Chambers, H., Goosby, E., Gandhi, M.**. Importance of non-pharmaceutical interventions in lowering the viral inoculum to reduce susceptibility to infection by SARS-CoV-2 and potentially disease severity. *The Lancet Infectious Diseases.* 2021. 21(9):e296-e301

**Subbaraman, N.**. How do vaccinated people spread Delta? What the science says. *Nature.* 2021. 596:327-328

**Sugimoto, H.,Kohama, T.**. Chest tube with air leaks is a potential "super spreader" of COVID-19. *American Journal of Infection Control.* 2020. 48:969

**Setlur, R.,Jaiswal, A.,Jahan, N.**. Preventing exposure to COVID-19 in the operation theatre and intensive care unit. *Journal of Anaesthesiology Clinical Pharmacology.* 2020. 36:S127-S133

**Shah, K.,Saxena, D.,Mavalankar, D.**. Secondary attack rate of COVID-19 in household contacts: a systematic review. *Qjm.* 2020. 113:841-850

**Shao, W.,Xie, J.,Zhu, Y.**. Mediation by human mobility of the association between temperature and COVID-19 transmission rate. *Environmental Research.* 2021. 194 (no pagination):#pages#

**Sharma, S.,Dash, P. K.,Sharma, S. K.,Srivastava, A.,Kumar, J. S.,Karothia, B. S.,Chelvam, K. T.,Singh, S.,Gupta, A.,Yadav, R. G.,Yadav, R.,Greeshma, T. S.,Kushwaha, P. K.,Kumar, R. B.,Nagar, D. P.,Nandan, M.,Kumar, S.,Thavaselvam, D.,Dubey, D. K.**. Emergence and expansion of highly infectious spike protein D614G mutant SARS-CoV-2 in central India. *Scientific Reports.* 2021. 11:18126

**Shen, J.,Duan, H.,Zhang, B.,Ji, J. S.,Wang, J.,Pan, L.,Wang, X.,Zhao, K.,Ying, B.,Tang, S.,Zhang, J.,Liang, C.,Sun, H.,Lv, Y.,Li, Y.,Li, T.,Li, L.,Liu, H.,Zhang, L.,Wang, L.,Shi, X.**. Prevention and control of COVID-19 in public transportation: Experience from China. *Environmental Pollution.* 2020. Part 2. 266 (no pagination):#pages#

**Siebach, M. K.,Piedimonte, G.,Ley, S. H.**. COVID-19 in childhood: Transmission, clinical presentation, complications and risk factors. *Pediatric Pulmonology.* 2021. 56:1342-1356

**Signorelli, C.,Odone, A.,Stirparo, G.,Cereda, D.,Gramegna, M.,Trivelli, M.,Rezza, G.**. SARS-CoV-2 transmission in the Lombardy Region: the increase of household contagion and its implication for containment measures. *Acta Bio-Medica de l Ateneo Parmense.* 2020. 91:e2020195

**Silva, R. R.,Ribeiro, C. J. N.,Moura, T. R.,Santos, M. B.,Santos, A. D.,Tavares, D. S.,Santos, P. L.**. Basic sanitation: a new indicator for the spread of COVID-19?. *Transactions of the Royal Society of Tropical Medicine & Hygiene.* 2021. 115:832-840

**Simeone Andrulli, MD, Hospital, A. Manzoni**. Transmission of COVID-19 Virus Among Patients and Staff in Dialysis Centers. *#journal#.* 2020. #volume#:#pages#

**Slifka, M. K.,Gao, L.**. Is presymptomatic spread a major contributor to COVID-19 transmission?. *Nature Medicine.* 2020. 26(10):1531-1533

**Sobouti, F.,Savasari, A. M.,Aryana, M.,Mesgarani, A.**. Coronavirus as a new challenge for infection control in dentistry: A literature review. [Persian]. *Journal of Mazandaran University of Medical Sciences.* 2020. 30(186):185-194

**Sommerstein, R.,Fux, C. A.,Vuichard-Gysin, D.,Abbas, M.,Widmer, A.,Balmelli, C.,Eisenring, M. C.,Harbarth, S.,Marschall, J.,Pittet, D.,Sax, H.,Schlegel, M.,Schweiger, A.,Senn, L.,Troillet, N.,Widmer, A. F.,Zanetti, G.**. Risk of SARS-CoV-2 transmission by aerosols, the rational use of masks, and protection of healthcare workers from COVID-19. *Antimicrobial Resistance and Infection Control.* 2020. 9(1) (no pagination):#pages#

**Sopeyin, A.,Hornsey, E.,Okwor, T.,Alimi, Y.,Raji, T.,Mohammed, A.,Moges, H.,Onwuekwe, E. V. C.,Minja, F. J.,Gon, G.,Ogbuagu, O.,Ogunsola, F.,Paintsil, E.**. Transmission risk of respiratory viruses in natural and mechanical ventilation environments: implications for SARS-CoV-2 transmission in Africa. *BMJ Global Health.* 2020. 5:08

**South Australian, Health, Medical Research, Institute**. Prevention of SARS-CoV-2 (COVID-19) transmission in residential aged care using ultraviolet light (PETRA): a parallel crossover randomised controlled trial. *#journal#.* 2021. #volume#:#pages#

**Spahn, C.,Hipp, A. M.,Schubert, B.,Axt, M. R.,Stratmann, M.,Schmolder, C.,Richter, B.**. Airflow and air velocity measurements while playing wind instruments, with respect to risk assessment of a sars-cov-2 infection. *International Journal of Environmental Research and Public Health.* 2021. 18(10) (no pagination):#pages#

**Storer, T. W.,Latham, N. K.,Bhasin, S.**. Maximizing Participant and Staff Safety During Assessment of Physical Function in the COVID-19 Era. *Journal of the American Geriatrics Society.* 2021. 69(1):12-17

**Sunkari, E. D.,Korboe, H. M.,Abu, M.,Kizildeniz, T.**. Sources and routes of SARS-CoV-2 transmission in water systems in Africa: Are there any sustainable remedies?. *Science of the Total Environment.* 2021. 753 (no pagination):#pages#

**Sage, L.,Albertini, M.,Scherer, S.**. The spreading of SARS-CoV-2: Interage contacts and networks degree distribution. *PLoS ONE [Electronic Resource].* 2021. 16:e0256036

**Sahasranaman, A.,Jensen, H. J.**. Spread of COVID-19 in urban neighbourhoods and slums of the developing world. *Journal of the Royal Society Interface.* 2021. 18:20200599

**Saidan, M. N.,Shbool, M. A.,Arabeyyat, O. S.,Al-Shihabi, S. T.,Abdallat, Y. A.,Barghash, M. A.,Saidan, H.**. Estimation of the probable outbreak size of novel coronavirus (COVID-19) in social gathering events and industrial activities. *International Journal of Infectious Diseases.* 2020. 98:321-327

**Santana-Cibrian, M.,Acuna-Zegarra, M. A.,Velasco-Hernandez, J. X.**. Lifting mobility restrictions and the effect of superspreading events on the short-term dynamics of COVID-19. *Mathematical Biosciences & Engineering: MBE.* 2020. 17:6240-6258

**Saraswathi, S.,Mukhopadhyay, A.,Shah, H.,Ranganath, T. S.**. Social network analysis of COVID-19 transmission in Karnataka, India. *Epidemiology & Infection.* 2020. 148:e230

**Sardar, T.,Rana, S.**. Effective Lockdown and Role of Hospital-Based COVID-19 Transmission in Some Indian States: An Outbreak Risk Analysis. *Risk Analysis.* 2021. 05:05

**Sartorius, B.,Lawson, A. B.,Pullan, R. L.**. Modelling and predicting the spatio-temporal spread of COVID-19, associated deaths and impact of key risk factors in England. *Scientific Reports.* 2021. 11:5378

**Saw, L. H.,Leo, B. F.,Nor, N. S. M.,Yip, C. W.,Ibrahim, N.,Hamid, H. H. A.,Latif, M. T.,Lin, C. Y.,Nadzir, M. S. M.**. Modeling aerosol transmission of SARS-CoV-2 from human-exhaled particles in a hospital ward. *Environmental science and pollution research international..* 2021. 25:#pages#

**Scarpone, C.,Brinkmann, S. T.,Grose, T.,Sonnenwald, D.,Fuchs, M.,Walker, B. B.**. A multimethod approach for county-scale geospatial analysis of emerging infectious diseases: a cross-sectional case study of COVID-19 incidence in Germany. *International Journal of Health Geographics [Electronic Resource].* 2020. 19:32

**Schade, W.,Reimer, V.,Seipenbusch, M.,Willer, U.**. Experimental Investigation of Aerosol and CO<sub>2</sub> Dispersion for Evaluation of COVID-19 Infection Risk in a Concert Hall. *International Journal of Environmental Research & Public Health [Electronic Resource].* 2021. 18:16

**Schade, W.,Reimer, V.,Seipenbusch, M.,Willer, U.,Hubner, E. G.**. Viral aerosol transmission of SARS-CoV-2 from simulated human emission in a concert hall. *International Journal of Infectious Diseases.* 2021. 107:12-14

**Schijven, J.,Vermeulen, L. C.,Swart, A.,Meijer, A.,Duizer, E.,de Roda Husman, A. M.**. Quantitative Microbial Risk Assessment for Airborne Transmission of SARS-CoV-2 via Breathing, Speaking, Singing, Coughing, and Sneezing. *Environmental Health Perspectives.* 2021. 129:47002

**Schultz, M.,Soolaki, M.**. Analytical approach to solve the problem of aircraft passenger boarding during the coronavirus pandemic. *Transportation Research Part C-Emerging Technologies.* 2021. 124:102931

**Scott, N.,Palmer, A.,Delport, D.,Abeysuriya, R.,Stuart, R. M.,Kerr, C. C.,Mistry, D.,Klein, D. J.,Sacks-Davis, R.,Heath, K.,Hainsworth, S. W.,Pedrana, A.,Stoove, M.,Wilson, D.,Hellard, M. E.**. Modelling the impact of relaxing COVID-19 control measures during a period of low viral transmission. *Medical Journal of Australia.* 2021. 214:79-83

**Scungio, M.,Crognale, S.,Lelli, D.,Carota, E.,Calabro, G.**. Characterization of the bioaerosol in a natural thermal cave and assessment of the risk of transmission of SARS-CoV-2 virus. *Environmental Geochemistry & Health.* 2021. 08:08

**Seno, H.**. An SIS model for the epidemic dynamics with two phases of the human day-to-day activity. *Journal of Mathematical Biology.* 2020. 80:2109-2140

**Sha, H.,Zhang, X.,Qi, D.**. Optimal control of high-rise building mechanical ventilation system for achieving low risk of COVID-19 transmission and ventilative cooling. *Sustainable Cities and Society.* 2021. 74:103256

**Shanmugam, R.,Ledlow, G.,Singh, K. P.**. Predicting COVID-19 cases with unknown homogeneous or heterogeneous resistance to infectivity. *PLoS ONE [Electronic Resource].* 2021. 16:e0254313

**Shao, S.,Zhou, D.,He, R.,Li, J.,Zou, S.,Mallery, K.,Kumar, S.,Yang, S.,Hong, J.**. Risk assessment of airborne transmission of COVID-19 by asymptomatic individuals under different practical settings. *Journal of Aerosol Science.* 2021. 151 (no pagination):#pages#

**Sharma, S. K.,Bangia, A.,Alshehri, M.,Bhardwaj, R.**. Nonlinear dynamics for the spread of pathogenesis of COVID-19 pandemic. *Journal of Infection and Public Health.* 2021. 14:817-831

**Shi, K. W.,Huang, Y. H.,Quon, H.,Ou-Yang, Z. L.,Wang, C.,Jiang, S. C.**. Quantifying the risk of indoor drainage system in multi-unit apartment building as a transmission route of SARS-CoV-2. *Science of the Total Environment.* 2021. 762 (no pagination):#pages#

**Shim, E.,Tariq, A.,Chowell, G.**. Spatial variability in reproduction number and doubling time across two waves of the COVID-19 pandemic in South Korea, February to July, 2020. *International Journal of Infectious Diseases.* 2021. 102:1-9

**Shumsky, R. A.,Debo, L.,Lebeaux, R. M.,Nguyen, Q. P.,Hoen, A. G.**. Retail store customer flow and COVID-19 transmission. *Proceedings of the National Academy of Sciences of the United States of America.* 2021. 118:16

**Siam, Z. S.,Arifuzzaman, M.,Ahmed, M. S.,Khan, F. A.,Rashid, M. H.,Islam, M. S.**. Dynamics of COVID-19 transmission in Dhaka and Chittagong: Two business hubs of Bangladesh. *Clinical Epidemiology and Global Health.* 2021. 10 (no pagination):#pages#

**Sinha, K., Yadav, M. S., Verma, U., Murallidharan, J. S., Kumar, V.**. Effect of recirculation zones on the ventilation of a public washroom. *Physics of Fluids.* 2021. 33:117101

**Sinnige, J. S.,Kooij, F. O.,van Schuppen, H.,Hollmann, M. W.,Sperna Weiland, N. H.**. Protection of healthcare workers during aerosol-generating procedures with local exhaust ventilation. *British Journal of Anaesthesia.* 2021. 126:e220-e222

**Skittrall, J. P.**. SARS-CoV-2 screening: effectiveness and risk of increasing transmission. *Journal of the Royal Society Interface.* 2021. 18:20210164

**Skums, P.,Kirpich, A.,Icer Baykal, P.,Zelikovsky, A.,Chowell, G.**. Global transmission network of SARS-CoV-2: from outbreak to pandemic. *MedRxiv : the Preprint Server for Health Sciences.* 2020. 27:27

**Smith, T. P.,Flaxman, S.,Gallinat, A. S.,Kinosian, S. P.,Stemkovski, M.,Juliette, T. Unwin H.,Watson, O. J.,Whittaker, C.,Cattarino, L.,Dorigatti, I.,Tristem, M.,Pearse, W. D.**. Temperature and population density influence SARS-CoV-2 transmission in the absence of nonpharmaceutical interventions. *Proceedings of the National Academy of Sciences of the United States of America.* 2021. 118(25) (no pagination):#pages#

**Somsen, G. A.,van Rijn, C.,Kooij, S.,Bem, R. A.,Bonn, D.**. Small droplet aerosols in poorly ventilated spaces and SARS-CoV-2 transmission. *The Lancet Respiratory Medicine.* 2020. 8(7):658-659

**Son, W. S.,R. ISEWIDs Team**. Individual-based simulation model for COVID-19 transmission in Daegu, Korea. *Epidemiology and health.* 2020. 42:e2020042

**Srivastava, S.,Zhao, X.,Manay, A.,Chen, Q.**. Effective ventilation and air disinfection system for reducing coronavirus disease 2019 (COVID-19) infection risk in office buildings. *Sustainable Cities and Society.* 2021. 75:103408

**Stabile, L.,Pacitto, A.,Mikszewski, A.,Morawska, L.,Buonanno, G.**. Ventilation procedures to minimize the airborne transmission of viruses in classrooms. *Building & Environment.* 2021. 202:108042

**Stefan, Moritz, Cornelia, Gottschick, Johannes, Horn, Mario, Popp, Susan, Langer, Bianca, Klee, Oliver, Purschke, Michael, Gekle, Angelika, Ihling, Rafael, Mikolajczyk**. The Risk of Indoor Sports and Culture Events for the Transmission of COVID-19 (Restart-19). *#journal#.* 2020. #volume#:#pages#

**Steyn, N.,Plank, M. J.,James, A.,Binny, R. N.,Hendy, S. C.,Lustig, A.**. Managing the risk of a COVID-19 outbreak from border arrivals. *Journal of the Royal Society Interface.* 2021. 18(177) (no pagination):#pages#

**Sun, C. L. F.,Zuccarelli, E.,Zerhouni, E. G. A.,Lee, J.,Muller, J.,Scott, K. M.,Lujan, A. M.,Levi, R.**. Predicting Coronavirus Disease 2019 Infection Risk and Related Risk Drivers in Nursing Homes: A Machine Learning Approach. *Journal of the American Medical Directors Association.* 2020. 21:1533-1538.e6

**Sun, Y.,Xie, J.,Hu, X.**. Detecting Spatial Clusters of Coronavirus Infection Across London During the Second Wave. *Applied Spatial Analysis & Policy.* 2021. #volume#:1-15

**Sun, Z.,Di, L.,Sprigg, W.,Tong, D.,Casal, M.**. Community venue exposure risk estimator for the COVID-19 pandemic. *Health & Place.* 2020. 66:102450

**Susswein, Z.,Bansal, S.**. Characterizing superspreading of SARS-CoV-2 : from mechanism to measurement. *MedRxiv : the Preprint Server for Health Sciences.* 2020. 11:11

**Tambyah, P. A.,Conly, J.,Voss, A.**. Community Outbreak Investigation of SARS-CoV-2 Transmission among Bus Riders in Eastern China - More Detailed Studies Are Needed. *JAMA Internal Medicine.* 2021. 181(5):719-720

**Tan, C.,Xiao, Y.,Wu, Y.,Wu, A.,Li, C.**. Gastrointestinal endoscopy operation-A potential transmission risk for SARS-CoV-2. *American Journal of Infection Control.* 2020. 48:1125-1126

**Tan, J.,Yang, C.**. Severe Acute Respiratory Syndrome Coronavirus 2 (SARS-CoV-2) Transmission in Patients With Cancer Still Being Described. *Clinical Oncology.* 2020. 32(7):478

**Tandon, V.,Raheja, A.**. Modified Suction Apparatus to Reduce the Transmission Risk of COVID-19 among Healthcare Providers. *Neurology India.* 2020. 68:1170-1171

**Tang, Z.,Sun, B.,Xu, B.**. A quick evaluation method of nosocomial infection risk for cancer hospitals during the COVID-19 pandemic. *Journal of Cancer Research & Clinical Oncology.* 2020. 146:1891-1892

**Teheran, A. A.,Camero, G.,Prado, R.,Moreno, B.,Trujillo, H.,Ramirez, R. A.,Miranda, D. C.,Paniz-Mondolfi, A.,Ramirez, J. D.**. Presumptive asymptomatic COVID-19 carriers' estimation and expected person-to-person spreading among repatriated passengers returning from China. *Travel Medicine & Infectious Disease.* 2020. 37:101688

**Tekam, B.**. Modes of transmission of covid-19. *International Journal of Research in Pharmaceutical Sciences.* 2020. 11(Special Issue 1):1734-1738

**Tenforde, M. W.,Feldstein, L. R.,Lindsell, C. J.,Patel, M. M.,Self, W. H.,Keipp Talbot, H.,Grijalva, C. G.,Rice, T. W.,Baughman, A. H.,McClellan, R.,Wang, L.,Hart, K. W.,Shapiro, N. I.,Kassem, A. M.,Sciarratta, C. N.,Dzuris, N.,Griggs, E. P.,Smith, E. R.,Ogokeh, C. E.,Wu, M.,Kim, S. S.,Marcet, P. L.,Siddula, A.**. Exposures in adult outpatients with COVID-19 infection during early community transmission, Tennessee. *Influenza and other Respiratory Viruses.* 2021. 15(1):175-177

**Thampi, N.,Sander, B.,Science, M.**. Preventing the introduction of SARS-CoV-2 into school settings. *Cmaj.* 2021. 193(1):E24-E25

**Tilmanne, A.,De Crombrugghe, G.,Al-Husni Al-Keilani, M.,Le Loc'h, G.,Delvenne, V.,Smeesters, P. R.**. A well-controlled Covid-19 cluster in a semi-closed adolescent psychiatry inpatient facility. *Clinical Microbiology & Infection.* 2021. 27:153-154

**Tobias, A.,Molina, T.**. Is temperature reducing the transmission of COVID-19 ?. *Environmental Research.* 2020. 186 (no pagination):#pages#

**Tokuda, Y., Sakihama, T., Aoki, M., Taniguchi, K., Deshpande, G. A., Suzuki, S., Uda, S., Kurokawa, K.**. COVID-19 outbreak on the Diamond Princess Cruise Ship in February 2020. *J Gen Fam Med.* 2020. 21:95-97

**Torjesen, I.**. Covid-19: Risk of aerosol transmission to staff outside of intensive care is likely to be higher than predicted. *BMJ.* 2021. 372:n354

**Tosta, E.**. Transmission of severe acute respiratory syndrome coronavirus 2 through asymptomatic carriers and aerosols: A major public health challenge. *Revista Da Sociedade Brasileira de Medicina Tropical.* 2020. 53:e20200669

**Thomas, R.,Lotfi, T.,Morgano, G. P.,Darzi, A.,Reinap, M.**. Update Alert 2: Ventilation Techniques and Risk for Transmission of Coronavirus Disease, Including COVID-19. *Annals of internal medicine.* 2020. 173(11):W152-W153

**Tian, T.,Huo, X.**. Secondary attack rates of COVID-19 in diverse contact settings, a meta-analysis. *Journal of Infection in Developing Countries.* 2020. 14:1361-1367

**Tian, Y.,Gong, Y. H.,Liu, P. Y.,Wang, S.,Xu, X. H.,Wang, X. Y.,Huang, Y. G.**. Infection Prevention Strategy in Operating Room during Coronavirus Disease 2019 (COVID-19) Outbreak. *Chinese Medical Sciences Journal.* 2020. 35:114-120

**Tinto, B.,Salinas, S.,Dicko, A.,Kagone, T. S.,Traore, I.,de Rekeneire, N.,Bicaba, B. W.,Hien, H.,Meda, N.,van de Perre, P.,Kania, D.,Simonin, Y.**. Spreading of SARS-CoV-2 in West Africa and assessment of risk factors. *Epidemiology & Infection.* 2020. 148:e213

**Trivedi, M.**. Covid-19: impact and dealings in orthodontic Practice design Post viral outbreak and Lockdown. *Biomedical and Pharmacology Journal.* 2020. 13(3):1387-1391

**Trujillo, A.,Arango, F.,Osorio, C.,Ocampo, F.,Melo, L.,Gil, L.**. Pediatric anesthesia techniques during the COVID-19 pandemic. *Signa Vitae.* 2020. 16(2):8-13

**Tummers, F. H.,Draaisma, W. A.,Demirkiran, A.,Brouwer, T. A.,Lagerveld, B. W.,van Schrojenstein Lantman, E. S.,Spijkers, K.,Coppus, S. F.,Jansen, F. W.**. Potential Risk and Safety Measures in Laparoscopy in COVID-19 Positive Patients. *Surgical Innovation.* 2021. #volume#:15533506211003527

**Tzampoglou, P.,Loukidis, D.**. Investigation of the importance of climatic factors in COVID-19 worldwide intensity. *International Journal of Environmental Research and Public Health.* 2020. 17(21):1-25

**Taboe, H. B.,Salako, K. V.,Tison, J. M.,Ngonghala, C. N.,Glele Kakai, R.**. Predicting COVID-19 spread in the face of control measures in West Africa. *Mathematical Biosciences.* 2020. 328 (no pagination):#pages#

**Tang, B.,Bragazzi, N. L.,Li, Q.,Tang, S.,Xiao, Y.,Wu, J.**. An updated estimation of the risk of transmission of the novel coronavirus (2019-nCov). *Infectious Disease Modelling.* 2020. 5:248-255

**Tang, L., Liu, M., Ren, B., Chen, J., Liu, X., Wu, X., Huang, W., Tian, J.**. Transmission in home environment associated with the second wave of COVID-19 pandemic in India. *Environmental Research.* 2022. Part A. 204 (no pagination):#pages#

**Tariq, A.,Banda, J. M.,Skums, P.,Dahal, S.,Castillo-Garsow, C.,Espinoza, B.,Brizuela, N. G.,Saenz, R. A.,Kirpich, A.,Luo, R.,Srivastava, A.,Gutierrez, H.,Chan, N. G.,Bento, A. I.,Jimenez-Corona, M. E.,Chowell, G.**. Transmission dynamics and forecasts of the COVID-19 pandemic in Mexico, March- December 2020. *PLoS ONE.* 2021. 16(7 July) (no pagination):#pages#

**Tariq, A.,Undurraga, E. A.,Laborde, C. C.,Vogt-Geisse, K.,Luo, R.,Rothenberg, R.,Chowell, G.**. Transmission dynamics and control of covid-19 in chile, march-october, 2020. *PLoS Neglected Tropical Diseases.* 2021. 15(1):1-20

**Tepekule, B.,Hauser, A.,Kachalov, V. N.,Andresen, S.,Scheier, T.,Schreiber, P. W.,Gunthard, H. F.,Kouyos, R. D.**. Assessing the potential impact of transmission during prolonged viral shedding on the effect of lockdown relaxation on COVID-19. *PLoS Computational Biology.* 2021. 17(1) (no pagination):#pages#

**Thomas Frederick, Johnson, Lisbeth, A. Hordley, Matthew, P. Greenwell, Luke, C. Evans**. Effect of park use and landscape structure on COVID-19 transmission rates. *#journal#.* 2020. #volume#:#pages#

**Tofighi, M., Asgary, A., Merchant, A. A., Shafiee, M. A., Najafabadi, M. M., Nadri, N., Aarabi, M., Heffernan, J., Wu, J.**. Modelling COVID-19 transmission in a hemodialysis centre using simulation generated contacts matrices. *PLoS ONE.* 2021. 16(11 November) (no pagination):#pages#

**Toth, D. J. A., Beams, A. B., Keegan, L. T., Zhang, Y., Greene, T., Orleans, B., Seegert, N., Looney, A., Alder, S. C., Samore, M. H.**. High variability in transmission of SARS-CoV-2 within households and implications for control. *PLoS ONE [Electronic Resource].* 2021. 16:e0259097

**Trentini, F.,Guzzetta, G.,Galli, M.,Zardini, A.,Manenti, F.,Putoto, G.,Marziano, V.,Gamshie, W. N.,Tsegaye, A.,Greblo, A.,Melegaro, A.,Ajelli, M.,Merler, S.,Poletti, P.**. Modeling the interplay between demography, social contact patterns, and SARS-CoV-2 transmission in the South West Shewa Zone of Oromia Region, Ethiopia. *BMC Medicine.* 2021. 19(1) (no pagination):#pages#

**Truszkowska, A.,Thakore, M.,Zino, L.,Butail, S.,Caroppo, E.,Jiang, Z. P.,Rizzo, A.,Porfiri, M.**. Designing the Safe Reopening of US Towns Through High-Resolution Agent-Based Modeling. *Advanced Theory and Simulations.* 2021. #volume#:2100157

**Tupper, P.,Boury, H.,Yerlanov, M.,Colijn, C.**. Event-specific interventions to minimize COVID-19 transmission. *Proceedings of the National Academy of Sciences of the United States of America.* 2020. 117:32038-32045

**Tupper, P.,Colijn, C.**. COVID-19 in schools: Mitigating classroom clusters in the context of variable transmission. *PLoS Computational Biology.* 2021. 17:e1009120

**University of North Carolina, Chapel Hill, Translational, North Carolina, Institute, Clinical Sciences**. COVID-19 Household Transmission Study. *#journal#.* 2020. #volume#:#pages#

**Ursachi, C. S.,Munteanu, F. D.,Cioca, G.**. The safety of slaughterhouse workers during the pandemic crisis. *International Journal of Environmental Research and Public Health.* 2021. 18(5):1-10

**Ulimwengu, J.,Kibonge, A.**. Spatial spillover and COVID-19 spread in the U.S. *BMC public health.* 2021. 21(1):1765

**Ute Muti-Schuenemann, G. E., Szczeklik, W., Solo, K., Khabsa, J., Thomas, R., Borowiack, E., Khamis, A. M., Hneiny, L., Darzi, A., Harrison, L., Bak, A., Bongnanni, A., Morgano, G. P., Stalteri, R., Hajizadeh, A., Lotfi, T., Reinap, M., Rochwerg, B., Akl, E. A., Schunemann, H. J.**. Update Alert 3: Ventilation Techniques and Risk for Transmission of Coronavirus Disease, Including COVID-19. *Annals of Internal Medicine.* 2021. 14:14

**Valent, F.,Gallo, T.,Mazzolini, E.,Pipan, C.,Sartor, A.,Merelli, M.,Bontempo, G.,Marzinotto, S.,Curcio, F.,Tascini, C.**. A cluster of COVID-19 cases in a small Italian town: a successful example of contact tracing and swab collection. *Clinical Microbiology and Infection.* 2020. 26(8):1112-1114

**Van Damme, W.,Dahake, R.,van de Pas, R.,Vanham, G.,Assefa, Y.**. COVID-19: Does the infectious inoculum dose-response relationship contribute to understanding heterogeneity in disease severity and transmission dynamics?. *Medical Hypotheses.* 2021. 146:110431

**Van Overmeire, R.,Bilsen, J.**. COVID-19: the risks for funeral directors. *Journal of Public Health.* 2020. 42:655

**Veer, K.**. Risk of animals spreading covid-19 to people. *Infectious Disorders - Drug Targets.* 2021. 21(4):478-479

**Velasco, M.,Guijarro, C.**. SARS-CoV-2 reinfection in a closed setting: lessons for the community. *The Lancet Respiratory Medicine.* 2021. 9(7):675-677

**Viner, R. M.,Bonell, C.,Drake, L.,Jourdan, D.,Davies, N.,Baltag, V.,Jerrim, J.,Proimos, J.,Darzi, A.**. Reopening schools during the COVID-19 pandemic: Governments must balance the uncertainty and risks of reopening schools against the clear harms associated with prolonged closure. *Archives of Disease in Childhood.* 2021. 106(2):111-113

**Vrancken, B.,Dellicour, S.,Mehta, S. R.,Strathdee, S.,Smith, D. M.,Chaillon, A.**. Dynamics of the covid-19 epidemic at the California-mexico border. *Topics in Antiviral Medicine.* 2021. 29(1):244

**Vardoulakis, S.,Espinoza Oyarce, D. A.,Donner, E.**. Transmission of COVID-19 and other infectious diseases in public washrooms: A systematic review. *Science of the Total Environment.* 2021. 803:149932

**Vashisht, D.,Neema, S.,Venugopalan, R.,Pathania, V.,Sandhu, S.,Vasudevan, B.**. Dermatology practice in the times of the COVID-19 pandemic. *Indian Journal of Dermatology, Venereology & Leprology.* 2021. 87:603-610

**Viner, R. M.,Russell, S. J.,Croker, H.,Packer, J.,Ward, J.,Stansfield, C.,Mytton, O.,Bonell, C.,Booy, R.**. School closure and management practices during coronavirus outbreaks including COVID-19: a rapid systematic review. *The Lancet Child and Adolescent Health.* 2020. 4(5):397-404

**Vythilingam, S., Quint, M., Newsom, R., Hicks, A.**. The use of aerosol generating procedures (AGPs) during the COVID-19 pandemic in the diagnosis of lung cancer: a narrative review. *Mediastinum.* 2021. 5:29

**VanderWaal, K.,Black, L.,Hodge, J.,Bedada, A.,Dee, S.**. Modeling transmission dynamics and effectiveness of worker screening programs for SARS-CoV-2 in pork processing plants. *PLoS ONE.* 2021. 16(9 September) (no pagination):#pages#

**Viscariello, N.,Evans, S.,Parker, S.,Schofield, D.,Miller, B.,Gardner, S.,Fong de Los Santos, L.,Hallemeier, C.,Jordan, L.,Kim, E.,Ford, E.**. A multi-institutional assessment of COVID-19-related risk in radiation oncology. *Radiotherapy & Oncology.* 2020. 153:296-302

**Vuorinen, V.,Aarnio, M.,Alava, M.,Alopaeus, V.,Atanasova, N.,Auvinen, M.,Balasubramanian, N.,Bordbar, H.,Erasto, P.,Grande, R.,Hayward, N.,Hellsten, A.,Hostikka, S.,Hokkanen, J.,Kaario, O.,Karvinen, A.,Kivisto, I.,Korhonen, M.,Kosonen, R.,Kuusela, J.,Lestinen, S.,Laurila, E.,Nieminen, H. J.,Peltonen, P.,Pokki, J.,Puisto, A.,Raback, P.,Salmenjoki, H.,Sironen, T.,Osterberg, M.**. Modelling aerosol transport and virus exposure with numerical simulations in relation to SARS-CoV-2 transmission by inhalation indoors. *Safety Science.* 2020. 130 (no pagination):#pages#

**Walke, H. T.,Honein, M. A.,Redfield, R. R.**. Preventing and Responding to COVID-19 on College Campuses. *JAMA - Journal of the American Medical Association.* 2020. 324(17):1727-1728

**Wan, K. H., Chow, V. W. S., Lam, D. S. C.**. Risk of SARS-CoV-2 Transmission via Corneal Transplant from Donors with COVID-19. *JAMA Ophthalmology.* 2021. 139(8):922-923

**Wang, D.,Zhou, M.,Nie, X.,Qiu, W.,Yang, M.,Wang, X.,Xu, T.,Ye, Z.,Feng, X.,Xiao, Y.,Chen, W.**. Epidemiological characteristics and transmission model of Corona Virus Disease 2019 in China. *Journal of Infection.* 2020. 80(5):e25-e27

**Wang, J.,Zhou, M.,Liu, F.**. Reasons for healthcare workers becoming infected with novel coronavirus disease 2019 (COVID-19) in China. *Journal of Hospital Infection.* 2020. 105:100-101

**Wang, X.,Du, Z.,Johnson, K. E.,Pasco, R. F.,Fox, S. J.,Lachmann, M.,McLellan, J. S.,Meyers, L. A.**. Effects of COVID-19 vaccination timing and risk prioritization on mortality rates, United States. *Emerging Infectious Diseases.* 2021. 27(7):1976-1979

**Wei, C.,Yuan, Y.,Cheng, Z.**. A super-spreader of SARS-CoV-2 in incubation period among health-care workers. *Respiratory Research.* 2020. 21:327

**Wen, H.,Barnett, M. L.,Saloner, B.**. Clinical Risk Factors for COVID-19 Among People With Substance Use Disorders. *Psychiatric Services.* 2020. 71:1308

**White, E. M.,Santostefano, C. M.,Feifer, R. A.,Kosar, C. M.,Blackman, C.,Gravenstein, S.,Mor, V.**. Asymptomatic and Presymptomatic Severe Acute Respiratory Syndrome Coronavirus 2 Infection Rates in a Multistate Sample of Skilled Nursing Facilities. *JAMA Internal Medicine.* 2020. 180:1709-1711

**Wolfensberger, A.,Schreiber, P. W.,Frey, A.,Scharer, V.,Hundal, C. O.,Saleschus, D.,Vazquez, M.,Zingg, W.**. Nosocomial COVID-19 in a tertiary care center - incidence and secondary attack rates after exposure. *Antimicrobial Resistance and Infection Control. Conference: 6th International Conference on Prevention and Infection Control, ICPIC.* 2021. 10:#pages#

**Wongsawat, J.,Moolasart, V.,Srikirin, P.,Srijareonvijit, C.,Vaivong, N.,Uttayamakul, S.,Disthakumpa, A.**. Risk of novel coronavirus 2019 transmission from children to caregivers: A case series. *Journal of Paediatrics and Child Health.* 2020. 56(6):984-985

**Walsh, K. A.,Tyner, B.,Broderick, N.,Harrington, P.,O'Neill, M.,Fawsitt, C. G.,Cardwell, K.,Smith, S. M.,Connolly, M. A.,Ryan, M.**. Effectiveness of public health measures to prevent the transmission of SARS-CoV-2 at mass gatherings: A rapid review. *Reviews in Medical Virology.* 2021. #volume#:e2285

**Walsh, S.,Chowdhury, A.,Braithwaite, V.,Russell, S.,Birch, J. M.,Ward, J. L.,Waddington, C.,Brayne, C.,Bonell, C.,Viner, R. M.,Mytton, O. T.**. Do school closures and school reopenings affect community transmission of COVID-19? A systematic review of observational studies. *BMJ Open.* 2021. 11(8) (no pagination):#pages#

**Wang, Q.,Dong, W.,Yang, K.,Ren, Z.,Huang, D.,Zhang, P.,Wang, J.**. Temporal and spatial analysis of COVID-19 transmission in China and its influencing factors. *International Journal of Infectious Diseases.* 2021. 105:675-685

**Wang, Y.,Deng, Z.,Shi, D.**. How effective is a mask in preventing COVID-19 infection?. *Medical Devices and Sensors.* 2021. 4(1) (no pagination):#pages#

**Wataganara, T.,Ruangvutilert, P.,Sunsaneevithayakul, P.,Sutantawibul, A.,Chuchotirot, M.,Phattanachindakun, B.,Russameecharoen, K.**. Minimizing cross transmission of SARS-CoV-2 in obstetric ultrasound during COVID-19 pandemic. *Journal of Perinatal Medicine.* 2020. 48(9):931-942

**Wu, J. K. Y.,Ryan, C. M.,Hiebert, R. J.,Han, Z.,Liu, A.,Jeong, C. H.,Mubareka, S.,Evans, G. J.,Chow, C. W.**. Aerosol generation during pulmonary function testing: Monitoring during different testing modalities. *Canadian Journal of Respiratory, Critical Care, and Sleep Medicine..* 2021. #volume#:#pages#

**Wagner, J.,Sparks, T. L.,Miller, S.,Chen, W.,Macher, J. M.,Waldman, J. M.**. Modeling the impacts of physical distancing and other exposure determinants on aerosol transmission. *Journal of Occupational & Environmental Hygiene.* 2021. #volume#:1-15

**Wang, D.,Tayarani, M.,Yueshuai He, B.,Gao, J.,Chow, J. Y. J.,Oliver Gao, H.,Ozbay, K.**. Mobility in post-pandemic economic reopening under social distancing guidelines: Congestion, emissions, and contact exposure in public transit. *Transportation Research. Part A, Policy & Practice.* 2021. 153:151-170

**Wang, K.,Zhao, S.,Liao, Y.,Zhao, T.,Wang, X.,Zhang, X.,Jiao, H.,Li, H.,Yin, Y.,Wang, M. H.,Xiao, L.,Wang, L.,He, D.**. Estimating the serial interval of the novel coronavirus disease (COVID-19) based on the public surveillance data in Shenzhen, China, from 19 January to 22 February 2020. *Transboundary & Emerging Diseases.* 2020. 67:2818-2822

**Wang, L.,Didelot, X.,Yang, J.,Wong, G.,Shi, Y.,Liu, W.,Gao, G. F.,Bi, Y.**. Inference of person-to-person transmission of COVID-19 reveals hidden super-spreading events during the early outbreak phase. *Nature communications.* 2020. 11:5006

**Wang, X. L.,Lin, X.,Yang, P.,Wu, Z. Y.,Li, G.,McGoogan, J. M.,Jiao, Z. T.,He, X. J.,Li, S. Q.,Shi, H. H.,Wang, J. Y.,Lai, S. J.,Huang, C.,Wang, Q. Y.**. Coronavirus disease 2019 outbreak in Beijing's Xinfadi Market, China: a modeling study to inform future resurgence response. *Infectious Diseases of Poverty.* 2021. 10:62

**Wang, Z.,Yang, W.,Hua, P.,Zhang, J.,Krebs, P.**. Transmission risk of SARS-CoV-2 in the watershed triggered by domestic wastewater discharge. *Science of the Total Environment.* 2021. #volume#:150888

**Webb, G.**. A COVID-19 epidemic model predicting the effectiveness of vaccination in the US. *Infectious Disease Reports.* 2021. 13(3):654-667

**Wells, C. R.,Townsend, J. P.,Pandey, A.,Moghadas, S. M.,Krieger, G.,Singer, B.,McDonald, R. H.,Fitzpatrick, M. C.,Galvani, A. P.**. Optimal COVID-19 quarantine and testing strategies. *MedRxiv : the Preprint Server for Health Sciences.* 2020. 30:30

**Wibowo, D. H.**. When can physical distancing be relaxed? A health production function approach for COVID-19 control policy. *BMC Public Health.* 2021. 21:1037

**Wilder, B.,Charpignon, M.,Killian, J. A.,Ou, H. C.,Mate, A.,Jabbari, S.,Perrault, A.,Desai, A. N.,Tambe, M.,Majumder, M. S.**. Modeling between-population variation in COVID-19 dynamics in Hubei, Lombardy, and New York City. *Proceedings of the National Academy of Sciences of the United States of America.* 2020. 117(41):25904-25910

**Wodarz, D.,Komarova, N. L.,Schang, L. M.**. Role of high-dose exposure in transmission hot zones as a driver of SARS-CoV-2 dynamics. *Journal of the Royal Society Interface.* 2021. 18(176) (no pagination):#pages#

**Wong, F.,Collins, J. J.**. Evidence that coronavirus superspreading is fat-tailed. *Proceedings of the National Academy of Sciences of the United States of America.* 2020. 117:29416-29418

**Xu, R.,Rahmandad, H.,Gupta, M.,DiGennaro, C.,Ghaffarzadegan, N.,Amini, H.,Jalali, M. S.**. Weather, air pollution, and SARS-CoV-2 transmission: a global analysis. *The lancet. Planetary Health.* 2021. 5:e671-e680

**Xu, C.,Liu, W.,Luo, X.,Huang, X.,Nielsen, P. V.**. Prediction and control of aerosol transmission of SARS-CoV-2 in ventilated context: from source to receptor. *Sustainable Cities and Society.* 2022. 76:103416

**Yao, Y.,Pan, J.,Liu, Z.,Meng, X.,Kan, H.,Wang, W.**. No association of COVID-19 transmission with temperature or UV radiation in Chinese cities. *European Respiratory Journal.* 2020. 55(5) (no pagination):#pages#

**Yasin, A. I.,Soysal, P.**. Preventing geriatric oncology patients from the spread of COVID-19. *Journal of Oncological Science.* 2020. 6(2):131-132
**Yang, C.,Wang, J.**. Modeling the transmission of COVID-19 in the US - A case study. *Infectious Disease Modelling.* 2021. 6:195-211

**Yang, C.,Wang, J.**. Transmission rates and environmental reservoirs for COVID-19 - a modeling study. *Journal of biological dynamics.* 2021. 15(1):86-108

**Yang, X. D.,Li, H. L.,Cao, Y. E.**. Influence of meteorological factors on the covid-19 transmission with season and geographic location. *International Journal of Environmental Research and Public Health.* 2021. 18(2):1-13

**Yin, G.,Jin, H.**. Comparison of Transmissibility of Coronavirus Between Symptomatic and Asymptomatic Patients: Reanalysis of the Ningbo COVID-19 Data. *JMIR Public Health and Surveillance.* 2020. 6:e19464

**Yu, Y.,Li, C.,Yang, W.,Xu, W.**. Determining the critical factors of air-conditioning innovation using an integrated model of fuzzy Kano-QFD during the COVID-19 pandemic: The perspective of air purification. *PLoS ONE.* 2021. 16(7 July) (no pagination):#pages#

**Zachreson, C.,Mitchell, L.,Lydeamore, M. J.,Rebuli, N.,Tomko, M.,Geard, N.**. Risk mapping for COVID-19 outbreaks in Australia using mobility data. *Journal of the Royal Society Interface.* 2021. 18:20200657

**Zafarnejad, R.,Griffin, P. M.**. Assessing school-based policy actions for COVID-19: An agent-based analysis of incremental infection risk. *Computers in Biology & Medicine.* 2021. 134:104518

**Zaneti, R. N.,Girardi, V.,Spilki, F. R.,Mena, K.,Westphalen, A. P. C.,da Costa Colares, E. R.,Pozzebon, A. G.,Etchepare, R. G.**. Quantitative microbial risk assessment of SARS-CoV-2 for workers in wastewater treatment plants. *Science of the Total Environment.* 2021. 754:142163

**Zaplotnik, Z.,Gavric, A.,Medic, L.**. Simulation of the COVID-19 epidemic on the social network of Slovenia: Estimating the intrinsic forecast uncertainty. *PLoS ONE.* 2020. 15(8 August) (no pagination):#pages#

**Zhang, J.,Litvinova, M.,Wang, W.,Wang, Y.,Deng, X.,Li, M.,Zheng, W.,Yi, L.,Chen, X.,Wu, Q.,Liang, Y.,Wang, X.,Yang, J.,Sun, K.,Longini, I. M.,Halloran, M. E.,Wu, P.,Cowling, B. J.,Merler, S.,Viboud, C.,Vespignani, A.,Ajelli, M.,Yu, H.**. Evolving epidemiology and transmission dynamics of coronavirus disease 2019 outside Hubei province, China: a descriptive and modelling study. *The Lancet Infectious Diseases.* 2020. 20(7):793-802

**Zhang, J.,Qin, F.,Qin, X.,Li, J.,Tian, S.,Lou, J.,Kang, X.,Lian, H.,Niu, S.,Zhang, W.,Chen, Y.**. Transmission of SARS-CoV-2 during air travel: a descriptive and modelling study. *Annals of Medicine.* 2021. 53:1569-1575

**Zhang, J.,Zhang, Y.,Kang, J. Y.,Chen, S.,He, Y.,Han, B.,Liu, M. F.,Lu, L.,Li, L.,Yi, Z.,Chen, L.**. Potential transmission chains of variant B.1.1.7 and co-mutations of SARS-CoV-2. *Cell Discovery.* 2021. 7:44

**Zhang, M., Shrestha, P., Liu, X., Turnaoglu, T., DeGraw, J., Schafer, D., Love, N.**. Computational fluid dynamics simulation of SARS-CoV-2 aerosol dispersion inside a grocery store. *Building & Environment.* 2021. #volume#:108652

**Zhang, S., Diao, M., Yu, W., Pei, L., Lin, Z., Chen, D.**. Estimation of the reproductive number of novel coronavirus (COVID-19) and the probable outbreak size on the Diamond Princess cruise ship: A data-driven analysis. *Int J Infect Dis.* 2020. 93:201-204

**Zhang, X.,Ji, Z.,Yue, Y.,Liu, H.,Wang, J.**. Infection Risk Assessment of COVID-19 through Aerosol Transmission: a Case Study of South China Seafood Market. *Environmental Science & Technology.* 2021. 55:4123-4133

**Zhang, Z.,Han, T.,Yoo, K. H.,Capecelatro, J.,Boehman, A. L.,Maki, K.**. Disease transmission through expiratory aerosols on an urban bus. *Physics of Fluids.* 2021. 33:015116

**Zhao, S.**. Estimating the time interval between transmission generations when negative values occur in the serial interval data: using COVID-19 as an example. *Mathematical Biosciences & Engineering: MBE.* 2020. 17:3512-3519

**Zhao, S.,Zhao, Y.,Tang, B.,Gao, D.,Guo, Z.,Chong, M. K. C.,Musa, S. S.,Cai, Y.,Wang, W.,He, D.,Wang, M. H.**. Shrinkage in serial intervals across transmission generations of COVID-19. *Journal of Theoretical Biology.* 2021. 529:110861

**Zhao, Z. Y.,Zhu, Y. Z.,Xu, J. W.,Hu, S. X.,Hu, Q. Q.,Lei, Z.,Rui, J.,Liu, X. C.,Wang, Y.,Yang, M.,Luo, L.,Yu, S. S.,Li, J.,Liu, R. Y.,Xie, F.,Su, Y. Y.,Chiang, Y. C.,Zhao, B. H.,Cui, J. A.,Yin, L.,Su, Y. H.,Zhao, Q. L.,Gao, L. D.,Chen, T. M.**. A five-compartment model of age-specific transmissibility of SARS-CoV-2. *Infectious Diseases of Poverty.* 2020. 9(1) (no pagination):#pages#

**Zhou, L.,Yao, M.,Zhang, X.,Hu, B.,Li, X.,Chen, H.,Zhang, L.,Liu, Y.,Du, M.,Sun, B.,Jiang, Y.,Zhou, K.,Hong, J.,Yu, N.,Ding, Z.,Xu, Y.,Hu, M.,Morawska, L.,Grinshpun, S. A.,Biswas, P.,Flagan, R. C.,Zhu, B.,Liu, W.,Zhang, Y.**. Breath-, air- and surface-borne SARS-CoV-2 in hospitals. *Journal of Aerosol Science.* 2021. 152:105693

**Zhou, Y. H.,Ma, K.,Xiao, P.,Ye, R. Z.,Zhao, L.,Cui, X. M.,Cao, W. C.**. An Optimal Nucleic Acid Testing Strategy for COVID-19 during the Spring Festival Travel Rush in Mainland China: A Modelling Study. *International Journal of Environmental Research & Public Health [Electronic Resource].* 2021. 18:12

**Zu, J., Shen, M., Fairley, C. K., Li, M., Li, G., Rong, L., Xiao, Y., Zhuang, G., Zhang, L., Li, Y.**. Investigating the relationship between reopening the economy and implementing control measures during the COVID-19 pandemic. *Public Health.* 2021. 200:15-21

**Zakka, K.,Erridge, S.,Chidambaram, S.,Beatty, J. W.,Kynoch, M.,Kinross, J.,Purkayastha, S.,PanSurg collaborative, group**. Electrocautery, Diathermy, and Surgical Energy Devices: Are Surgical Teams at Risk During the COVID-19 Pandemic?. *Annals of Surgery.* 2020. 272:e257-e262

**Zhang, X. S.,Duchaine, C.**. SARS-CoV-2 and Health Care Worker Protection in Low-Risk Settings: a Review of Modes of Transmission and a Novel Airborne Model Involving Inhalable Particles. *Clinical Microbiology Reviews.* 2020. 34:16

**Zhao, X. Y.,Shen, Z. Q.,Sun, L. T.,Cheng, L.,Wang, M. Y.,Zhang, X. F.,Xu, B.,Tian, L. L.,Miao, Y. Q.,Wu, X. Y.,Zou, K.,Li, J. Y.**. A network meta-analysis of secondary attack rates of COVID-19 in different contact environments. *Epidemiology and Infection..* 2021. #volume#:#pages#

**Zhu, Y.,Bloxham, C. J.,Hulme, K. D.,Sinclair, J. E.,Tong, Z. W. M.,Steele, L. E.,Noye, E. C.,Lu, J.,Xia, Y.,Chew, K. Y.,Pickering, J.,Gilks, C.,Bowen, A. C.,Short, K. R.**. A Meta-analysis on the Role of Children in Severe Acute Respiratory Syndrome Coronavirus 2 in Household Transmission Clusters. *Clinical Infectious Diseases.* 2021. 72:e1146-e1153

**Zielinski, S.,Botero, C. M.**. Beach tourism in times of COVID-19 pandemic: Critical issues, knowledge gaps and research opportunities. *International Journal of Environmental Research and Public Health.* 2020. 17(19):1-19

**Zuo, Y. Y.,Uspal, W. E.,Wei, T.**. Airborne Transmission of COVID-19: Aerosol Dispersion, Lung Deposition, and Virus-Receptor Interactions. *Acs Nano.* 2020. 25:25

**Zhang, W.,Cheng, W.,Luo, L.,Ma, Y.,Xu, C.,Qin, P.,Zhang, Z.**. Secondary Transmission of Coronavirus Disease from Presymptomatic Persons, China. *Emerging Infectious Diseases.* 2020. 26:1924-1926

**Zhang, X. A.,Fan, H.,Qi, R. Z.,Zheng, W.,Zheng, K.,Gong, J. H.,Fang, L. Q.,Liu, W.**. Importing coronavirus disease 2019 (COVID-19) into China after international air travel. *Travel Medicine and Infectious Disease.* 2020. 35 (no pagination):#pages#

**Zhang, Y.,Wen, J.,Chen, C.,Zeng, L.,Yang, L.,Huang, C.,Feng, A.,Miao, X.,Alamgir, M.,Rao, B.,Li, Y.,Tao, J.**. Challenges and countermeasures in the prevention of nosocomial infections of SARS-CoV-2 before resumption of work: Implications for the dermatology department. *Journal of the American Academy of Dermatology.* 2020. 83:961-963

**Zhao, S.,Liang, X.**. A re-analysis to identify the structural breaks in COVID-19 transmissibility during the early phase of the outbreak in South Korea. *International Journal of Infectious Diseases.* 2020. 100:10-11

**Zhao, S.,Zhuang, Z.,Cao, P.,Ran, J.,Gao, D.,Lou, Y.,Yang, L.,Cai, Y.,Wang, W.,He, D.,Wang, M. H.**. Quantifying the association between domestic travel and the exportation of novel coronavirus (2019-nCoV) cases from Wuhan, China in 2020: a correlational analysis. *Journal of Travel Medicine.* 2020. 27:13

**Zhou, J.,Tan, Y.,Li, D.,He, X.,Yuan, T.,Long, Y.**. Observation and analysis of 26 cases of asymptomatic SARS-COV2 infection. *Journal of Infection.* 2020. 81(1):e69-e70

**Zhu, Y.,Chen, L.,Ji, H.,Xi, M.,Fang, Y.,Li, Y.**. The Risk and Prevention of Novel Coronavirus Pneumonia Infections Among Inpatients in Psychiatric Hospitals. *Neuroscience Bulletin.* 2020. 36:299-302

# **Excluded settings:**

**Alqayoudhi, A.,Al Manji, A.,Al Khalili, S.,Al Maani, A.,Alkindi, H.,Alyaquobi, F.,Al Rawahi, B.,Al-Jardani, A.,Al Wahaibi, A.,Al-Abri, S.**. The role of children and adolescents in the transmission of SARS-CoV-2 virus within family clusters: A large population study from Oman. *Journal of Infection and Public Health.* 2021. 14:1590-1594

**Altamirano, J.,Govindarajan, P.,Leary, S.,Pinsky, B. A.,Blomkalns, A. L.,Maldonado, Y.**. Natural history of shedding and household transmission of SARS-CoV- 2. *Academic Emergency Medicine.* 2021. 28(SUPPL 1):S229

**Angulo-Bazan, Y.,Solis-Sanchez, G.,Cardenas, F.,Jorge, A.,Acosta, J.,Cabezas, C.**. Household transmission of SARS-CoV-2 (COVID-19) in Lima, Peru. *Cadernos de Saude Publica.* 2021. 37:e00238720

**Bart, S. M.,Flaherty, E.,Alpert, T.,Carlson, S.,Fasulo, L.,Earnest, R.,White, E. B.,Dickens, N.,Brito, A. F.,Grubaugh, N. D.,Hadler, J. L.,Sosa, L. E.**. Multiple Transmission Chains within COVID-19 Cluster, Connecticut, USA, 2020<sup>1</sup>. *Emerging Infectious Diseases.* 2021. 27:2669-2672

**Bernardes-Souza, B.,Junior, S. R. C.,Santos, C. A.,Neto, Rmdn,Bottega, F. C.,Godoy, D. C.,Freitas, B. L.,Silva, D. L. G.,Brinker, T. J.,Nascimento, R. A.,Tupinambas, U.,Reis, A. B.,Coura-Vital, W.**. Logistics Workers Are a Key Factor for SARS-CoV-2 Spread in Brazilian Small Towns: Case-Control Study. *JMIR Public Health and Surveillance.* 2021. 7:e30406

**Bi, Q.,Wu, Y.,Mei, S.,Ye, C.,Zou, X.,Zhang, Z.,Liu, X.,Wei, L.,Truelove, S. A.,Zhang, T.,Gao, W.,Cheng, C.,Tang, X.,Wu, X.,Wu, Y.,Sun, B.,Huang, S.,Sun, Y.,Zhang, J.,Ma, T.,Lessler, J.,Feng, T.**. Epidemiology and transmission of COVID-19 in 391 cases and 1286 of their close contacts in Shenzhen, China: a retrospective cohort study. *The Lancet Infectious Diseases.* 2020. 20:911-919

**Bistaraki, A., Roussos, S., Tsiodras, S., Sypsa, V.**. Age-dependent effects on infectivity and susceptibility to SARS-CoV-2 infection: results from nationwide contact tracing data in Greece. *Infectious Diseases.* 2021. #volume#:1-10

**Broccia, M. M.,de Knegt, Veve,Mills, Ehaeha,Moller, Alal,Gnesin, F. F.,Fischer, Tktk,Zylyftari, N. N.,Blomberg, Snsn,Andersen, Mpmp,Schou, M. M.,Fosbol, E. E.,Kragholm, K. K.,Christensen, Hchc,Polcwiartek, Lblb,Phelps, M. M.,Kober, L. L.,Torp-Pedersen, C. C.**. Household exposure to SARS-CoV-2 and association with COVID-19 severity: a Danish nationwide cohort study. *Clinical Infectious Diseases.* 2021. 24:24

**Buchan, S. A.,Tibebu, S.,Daneman, N.,Whelan, M.,Vanniyasingam, T.,Murti, M.,Brown, K. A.**. Increased household secondary attacks rates with Variant of Concern SARS-CoV-2 index cases. *Clinical Infectious Diseases.* 2021. 09:09

**Burke, R. M.,Calderwood, L.,Killerby, M. E.,Ashworth, C. E.,Berns, A. L.,Brennan, S.,Bressler, J. M.,Morano, L. H.,Lewis, N. M.,Markus, T. M.,Newton, S. M.,Read, J. S.,Rissman, T.,Taylor, J.,Tate, J. E.,Midgley, C. M.,Covid- Case Investigation Form Working Group**. Patterns of Virus Exposure and Presumed Household Transmission among Persons with Coronavirus Disease, United States, January-April 2020. *Emerging Infectious Diseases.* 2021. 27:2323-2332

**Cerami, C.,Popkin-Hall, Z. R.,Rapp, T.,Tompkins, K.,Zhang, H.,Muller, M. S.,Basham, C.,Whittelsey, M.,Chhetri, S. B.,Smith, J.,Litel, C.,Lin, K. D.,Churiwal, M.,Khan, S.,Rubinstein, R.,Claman, F.,Mollan, K.,Wohl, D.,Premkumar, L.,Powers, K. A.,Juliano, J. J.,Lin, F. C.,Lin, J. T.**. Household transmission of SARS-CoV-2 in the United States: living density, viral load, and disproportionate impact on communities of color. *Clinical Infectious Diseases.* 2021. 12:12

**Cerami, C.,Rapp, T.,Lin, F. C.,Tompkins, K.,Basham, C.,Muller, M. S.,Whittelsey, M.,Zhang, H.,Chhetri, S. B.,Smith, J.,Litel, C.,Lin, K.,Churiwal, M.,Khan, S.,Claman, F.,Rubinstein, R.,Mollan, K.,Wohl, D.,Premkumar, L.,Juliano, J. J.,Lin, J. T.**. High household transmission of SARS-CoV-2 in the United States: living density, viral load, and disproportionate impact on communities of color. *MedRxiv : the Preprint Server for Health Sciences.* 2021. 12:12

**Cohen, C., Kleynhans, J., von Gottberg, A., McMorrow, M. L., Wolter, N., Bhiman, J. N., Moyes, J., du Plessis, M., Carrim, M., Buys, A., Martinson, N. A., Kahn, K., Tollman, S., Lebina, L., Wafawanaka, F., du Toit, J., Gomez-Olive, F. X., Dawood, F. S., Mkhencele, T., Sun, K., Viboud, C., group, Phirst, Tempia, S.**. SARS-CoV-2 incidence, transmission and reinfection in a rural and an urban setting: results of the PHIRST-C cohort study, South Africa, 2020-2021. *MedRxiv : the Preprint Server for Health Sciences.* 2021. 04:04

**D'Onofrio, L. E., Jr.,Buono, F. D.,Cooper, M. A. R.**. Cohabitation COVID-19 transmission rates in a United States suburban community: A retrospective study of familial infections. *Public Health.* 2021. 192:30-32

**Dattner, I.,Goldberg, Y.,Katriel, G.,Yaari, R.,Gal, N.,Miron, Y.,Ziv, A.,Sheffer, R.,Hamo, Y.,Huppert, A.**. The role of children in the spread of COVID-19: Using household data from Bnei Brak, Israel, to estimate the relative susceptibility and infectivity of children. *PLoS Computational Biology.* 2021. 17(2) (no pagination):#pages#

**de Gier, B., Andeweg, S., Backer, J. A., surveillance, Rivm Covid-, epidemiology, team, Hahne, S. J., van den Hof, S., de Melker, H. E., Knol, M. J., surveillance, Rivm Covid-, epidemiology, team**. Vaccine effectiveness against SARS-CoV-2 transmission to household contacts during dominance of Delta variant (B.1.617.2), the Netherlands, August to September 2021. *Euro Surveillance: Bulletin Europeen sur les Maladies Transmissibles = European Communicable Disease Bulletin.* 2021. 26:11

**Farronato, M.,Dolci, C.,Boccalari, E.,Izadi, S.,Rios, L. H. S.,Festa, M.,Panetta, V.,De Vito, D.,Tartaglia, G. M.**. Serological profile of children and young adults with at least one sars-cov-2 positive cohabitant: An observational study. *International Journal of Environmental Research and Public Health.* 2021. 18(4):1-12

**Ghosh, A. K.,Venkatraman, S.,Soroka, O.,Reshetnyak, E.,Rajan, M.,An, A.,Chae, J. K.,Gonzalez, C.,Prince, J.,DiMaggio, C.,Ibrahim, S.,Safford, M. M.,Hupert, N.**. Association between overcrowded households, multigenerational households, and COVID-19: a cohort study. *Public Health.* 2021. 198:273-279

**Gomaa, M. R.,El Rifay, A. S.,Shehata, M.,Kandeil, A.,Nabil Kamel, M.,Marouf, M. A.,GabAllah, M.,El Taweel, A.,Kayed, A. E.,Kutkat, O.,Moatasim, Y.,Mahmoud, S. H.,Abo Shama, N. M.,El Sayes, M.,Mostafa, A.,El-Shesheny, R.,McKenzie, P. P.,Webby, R. J.,Kayali, G.,Ali, M. A.**. Incidence, household transmission, and neutralizing antibody seroprevalence of Coronavirus Disease 2019 in Egypt: Results of a community-based cohort. *PLoS Pathogens.* 2021. 17:e1009413

**Gray, A.,Wang, Y.,Grovit-Ferbas, K.,Tobin, N.,Brooker, S.,Ganz, P.,Aldrovandi, G.**. The role of children in the transmission of SARS-COV-2 in households of immunocompromised persons. *Pediatric Blood and Cancer.* 2021. 68(SUPPL 3):S81

**Gunadi,,Wibawa, H.,Hakim, M. S.,Marcellus,,Trisnawati, I.,Khair, R. E.,Triasih, R.,Irene,,Afiahayati,,Iskandar, K.,Siswanto,,Anggorowati, N.,Daniwijaya, E. W.,Supriyati, E.,Nugrahaningsih, D. A. A.,Budiono, E.,Retnowulan, H.,Puspadewi, Y.,Puspitawati, I.,Sianipar, O.,Afandy, D.,Simanjaya, S.,Widitjiarso, W.,Puspitarani, D. A.,Fahri, F.,Riawan, U.,Fauzi, A. R.,Kalim, A. S.,Ananda, N. R.,Setyati, A.,Setyowireni, D.,Laksanawati, I. S.,Arguni, E.,Nuryastuti, T.,Wibawa, T.,the Yogyakarta-Central Java, Covid-study group**. Molecular epidemiology of SARS-CoV-2 isolated from COVID-19 family clusters. *BMC Medical Genomics [Electronic Resource].* 2021. 14:144

**Hall, J. A.,Harris, R. J.,Zaidi, A.,Woodhall, S. C.,Dabrera, G.,Dunbar, J. K.**. HOSTED-England's Household Transmission Evaluation Dataset: preliminary findings from a novel passive surveillance system of COVID-19. *International Journal of Epidemiology.* 2021. 50:743-752

**Hayward, A.,Fragaszy, E.,Kovar, J.,Nguyen, V.,Beale, S.,Byrne, T.,Aryee, A.,Hardelid, P.,Wijlaars, L.,Fong, W. L. E.,Geismar, C.,Patel, P.,Shrotri, M.,Navaratnam, A. M. D.,Nastouli, E.,Spyer, M.,Killingley, B.,Cox, I.,Lampos, V.,McKendry, R. A.,Liu, Y.,Cheng, T.,Johnson, A. M.,Michie, S.,Gibbs, J.,Gilson, R.,Rodger, A.,Aldridge, R. W.**. Risk factors, symptom reporting, healthcare-seeking behaviour and adherence to public health guidance: protocol for Virus Watch, a prospective community cohort study. *BMJ Open.* 2021. 11:e048042

**Hsu, C. Y.,Wang, J. T.,Huang, K. C.,Fan, A. C.,Yeh, Y. P.,Chen, S. L.**. Household transmission but without the community-acquired outbreak of COVID-19 in Taiwan. *Journal of the Formosan Medical Association.* 2021. 120 Suppl 1:S38-S45

**Hu, P.,Ma, M.,Jing, Q.,Ma, Y.,Gan, L.,Chen, Y.,Liu, J.,Wang, D.,Zhang, Z.,Zhang, D.**. Retrospective study identifies infection related risk factors in close contacts during COVID-19 epidemic. *International Journal of Infectious Diseases.* 2021. 103:395-401

**Hubiche, T.,Phan, A.,Leducq, S.,Rapp, J.,Fertitta, L.,Aubert, H.,Barbarot, S.,Chiaverini, C.,Giraudeau, B.,Lasek, A.,Mallet, S.,Labarelle, A.,Piram, M.,McCuaig, C.,Martin, L.,Monitor, L.,Nicol, I.,Bissuel, M.,Bellissen, A.,Jullien, D.,Lesort, C.,Vabres, P.,Maruani, A.,Research Group of the Societe Francaise de Dermatologie, Pediatrique**. Acute acral eruptions in children during the COVID-19 pandemic: Characteristics of 103 children and their family clusters. *Annales de Dermatologie et de Venereologie.* 2021. 148:94-100

**Jashaninejad, R.,Doosti-Irani, A.,Karami, M.,Keramat, F.,Mirzaei, M.**. Transmission of COVID-19 and its Determinants among Close Contacts of COVID-19 Patients Running title. *Journal of Research in Health Sciences.* 2021. 21:e00514

**Jing, Q. L.,Liu, M. J.,Zhang, Z. B.,Fang, L. Q.,Yuan, J.,Zhang, A. R.,Dean, N. E.,Luo, L.,Ma, M. M.,Longini, I.,Kenah, E.,Lu, Y.,Ma, Y.,Jalali, N.,Yang, Z. C.,Yang, Y.**. Household secondary attack rate of COVID-19 and associated determinants in Guangzhou, China: a retrospective cohort study. *The Lancet Infectious Diseases.* 2020. 20:1141-1150

**Julin, C. H., Robertson, A. H., Hungnes, O., Tunheim, G., Bekkevold, T., Laake, I., Aune, I. F., Killengreen, M. F., Strand, T. R., Rykkvin, R., Dorenberg, D. H., Stene-Johansen, K., Berg, E. S., Bodin, J. E., Oftung, F., Steens, A., Naess, L. M.**. Household Transmission of SARS-CoV-2: A Prospective Longitudinal Study Showing Higher Viral Load and Increased Transmissibility of the Alpha Variant Compared to Previous Strains. *Microorganisms.* 2021. 9:17

**Katlama, C., Dudoit, Y., Huyard, J., Blanc, C., Soulie, C., Schneider, L., Faycal, A., Lenclume, L., Hamani, N., Qatib, N., Tubiana, R., Seang, S., Sellem, B., Costagliola, D., Palich, R., Valantin, M. A., Assoumou, L.**. Household transmission of SARS-CoV-2 infection in the Paris/Ile-de-France area. *European Journal of Clinical Microbiology & Infectious Diseases.* 2022. 08:08

**Kawasuji, H.,Takegoshi, Y.,Kaneda, M.,Ueno, A.,Miyajima, Y.,Kawago, K.,Fukui, Y.,Yoshida, Y.,Kimura, M.,Yamada, H.,Sakamaki, I.,Tani, H.,Morinaga, Y.,Yamamoto, Y.**. Transmissibility of COVID-19 depends on the viral load around onset in adult and symptomatic patients. *PLoS ONE.* 2020. 15(12 December) (no pagination):#pages#

**Kevin, L. Schwartz, Camille, Achonu, Sarah, A. Buchan, Kevin, A. Brown, Brenda, Lee, Michael, Whelan, Julie, H. C. Wu, Gary, Garber**. Healthcare Worker COVID-19 Cases in Ontario, Canada: A Cross-sectional Study. *#journal#.* 2020. #volume#:#pages#

**Kim, J.,Choe, Y. J.,Lee, J.,Park, Y. J.,Park, O.,Han, M. S.,Kim, J. H.,Choi, E. H.**. Role of children in household transmission of COVID-19. *Archives of Disease in Childhood.* 2021. 106:709-711

**Kuba, Y.,Shingaki, A.,Nidaira, M.,Kakita, T.,Maeshiro, N.,Oyama, M.,Kudeken, T.,Miyagi, A.,Yamauchi, M.,Kyan, H.**. The characteristics of household transmission during COVID-19 outbreak in Okinawa, Japan from February to May 2020. *Japanese Journal of Infectious Diseases.* 2021. 30:30

**Kuwelker, K.,Zhou, F.,Blomberg, B.,Lartey, S.,Brokstad, K. A.,Trieu, M. C.,Bansal, A.,Madsen, A.,Krammer, F.,Mohn, K. G.,Tondel, C.,Linchausen, D. W.,Cox, R. J.,Langeland, N.,Bergen, Covid-research group**. Attack rates amongst household members of outpatients with confirmed COVID-19 in Bergen, Norway: A case-ascertained study. *The Lancet Regional Health. Europe.* 2021. 3:100014

**Laws, R. L.,Chancey, R. J.,Rabold, E. M.,Chu, V. T.,Lewis, N. M.,Fajans, M.,Reses, H. E.,Duca, L. M.,Dawson, P.,Conners, E. E.,Gharpure, R.,Yin, S.,Buono, S.,Pomeroy, M.,Yousaf, A. R.,Owusu, D.,Wadhwa, A.,Pevzner, E.,Battey, K. A.,Njuguna, H.,Fields, V. L.,Salvatore, P.,O'Hegarty, M.,Vuong, J.,Gregory, C. J.,Banks, M.,Rispens, J.,Dietrich, E.,Marcenac, P.,Matanock, A.,Pray, I.,Westergaard, R.,Dasu, T.,Bhattacharyya, S.,Christiansen, A.,Page, L.,Dunn, A.,Atkinson-Dunn, R.,Christensen, K.,Kiphibane, T.,Willardson, S.,Fox, G.,Ye, D.,Nabity, S. A.,Binder, A.,Freeman, B. D.,Lester, S.,Mills, L.,Thornburg, N.,Hall, A. J.,Fry, A. M.,Tate, J. E.,Tran, C. H.,Kirking, H. L.**. Symptoms and Transmission of SARS-CoV-2 Among Children - Utah and Wisconsin, March-May 2020. *Pediatrics.* 2021. 147:01

**Lewis, N. M.,Chu, V. T.,Ye, D.,Conners, E. E.,Gharpure, R.,Laws, R. L.,Reses, H. E.,Freeman, B. D.,Fajans, M.,Rabold, E. M.,Dawson, P.,Buono, S.,Yin, S.,Owusu, D.,Wadhwa, A.,Pomeroy, M.,Yousaf, A.,Pevzner, E.,Njuguna, H.,Battey, K. A.,Tran, C. H.,Fields, V. L.,Salvatore, P.,O'Hegarty, M.,Vuong, J.,Chancey, R.,Gregory, C.,Banks, M.,Rispens, J. R.,Dietrich, E.,Marcenac, P.,Matanock, A. M.,Duca, L.,Binder, A.,Fox, G.,Lester, S.,Mills, L.,Gerber, S. I.,Watson, J.,Schumacher, A.,Pawloski, L.,Thornburg, N. J.,Hall, A. J.,Kiphibane, T.,Willardson, S.,Christensen, K.,Page, L.,Bhattacharyya, S.,Dasu, T.,Christiansen, A.,Pray, I. W.,Westergaard, R. P.,Dunn, A. C.,Tate, J. E.,Nabity, S. A.,Kirking, H. L.**. Household Transmission of SARS-CoV-2 in the United States. *Clinical infectious diseases : an official publication of the Infectious Diseases Society of America..* 2020. 16:#pages#

**Li, F.,Li, Y. Y.,Liu, M. J.,Fang, L. Q.,Dean, N. E.,Wong, G. W. K.,Yang, X. B.,Longini, I.,Halloran, M. E.,Wang, H. J.,Liu, P. L.,Pang, Y. H.,Yan, Y. Q.,Liu, S.,Xia, W.,Lu, X. X.,Liu, Q.,Yang, Y.,Xu, S. Q.**. Household transmission of SARS-CoV-2 and risk factors for susceptibility and infectivity in Wuhan: a retrospective observational study. *The Lancet Infectious Diseases.* 2021. 21:617-628

**Li, J.,Ding, J.,Chen, L.,Hong, L.,Yu, X.,Ye, E.,Sun, G.,Zhang, B.,Zhang, X.,Sun, Q.**. Epidemiological and clinical characteristics of three family clusters of COVID-19 transmitted by latent patients in China. *Epidemiology & Infection.* 2020. 148:e137

**Li, W.,Zhang, B.,Lu, J.,Liu, S.,Chang, Z.,Peng, C.,Liu, X.,Zhang, P.,Ling, Y.,Tao, K.,Chen, J.**. Characteristics of Household Transmission of COVID-19. *Clinical Infectious Diseases.* 2020. 71:1943-1946

**Li, Y.,Liu, J.,Yang, Z.,Yu, J.,Xu, C.,Zhu, A.,Zhang, H.,Yang, X.,Zhao, X.,Ren, M.,Li, Z.,Cui, J.,Zhao, H.,Ren, X.,Sun, C.,Cheng, Y.,Chen, Q.,Chang, Z.,Sun, J.,Rodewald, L. E.,Wang, L.,Feng, L.,Gao, G. F.,Feng, Z.,Li, Z.**. Transmission of Severe Acute Respiratory Syndrome Coronavirus 2 to Close Contacts, China, January-February 2020. *Emerging Infectious Diseases.* 2021. 27:2288-2293

**Lindstrom, J. C.,Engebretsen, S.,Kristoffersen, A. B.,Ro, G. O. I.,Palomares, A. D.,Engo-Monsen, K.,Madslien, E. H.,Forland, F.,Nygard, K. M.,Hagen, F.,Gantzel, G.,Wiklund, O.,Frigessi, A.,de Blasio, B. F.**. Increased transmissibility of the alpha SARS-CoV-2 variant: evidence from contact tracing data in Oslo, January to February 2021. *Infectious Diseases.* 2021. #volume#:1-6

**Loconsole, D.,Sallustio, A.,Centrone, F.,Casulli, D.,Ferrara, M. M.,Sanguedolce, A.,Accogli, M.,Chironna, M.**. An autochthonous outbreak of the sars-cov-2 p.1 variant of concern in southern italy, april 2021. *Tropical Medicine and Infectious Disease.* 2021. 6(3) (no pagination):#pages#

**Luo, L.,Liu, D.,Liao, X.,Wu, X.,Jing, Q.,Zheng, J.,Liu, F.,Yang, S.,Bi, H.,Li, Z.,Liu, J.,Song, W.,Zhu, W.,Wang, Z.,Zhang, X.,Huang, Q.,Chen, P.,Liu, H.,Cheng, X.,Cai, M.,Yang, P.,Yang, X.,Han, Z.,Tang, J.,Ma, Y.,Mao, C.**. Contact Settings and Risk for Transmission in 3410 Close Contacts of Patients With COVID-19 in Guangzhou, China : A Prospective Cohort Study. *Annals of Internal Medicine.* 2020. 173:879-887

**Lyngse, F. P., Kirkeby, C., Halasa, T., Andreasen, V., Skov, R. L., Moller, F. T., Krause, T. G., Molbak, K.**. Nationwide study on SARS-CoV-2 transmission within households from lockdown to reopening, Denmark, 27 February 2020 to 1 August 2020. *Euro Surveillance: Bulletin Europeen sur les Maladies Transmissibles = European Communicable Disease Bulletin.* 2022. 27:02

**Makinde, O. A.,Akinyemi, J. O.,Ntoimo, L. F.,Ajaero, C. K.,Ononokpono, D.,Banda, P. C.,Adewoyin, Y.,Petlele, R.,Ugwu, H.,Odimegwu, C. O.**. Risk assessment for COVID-19 transmission at household level in sub-Saharan Africa: evidence from DHS. *Genus.* 2021. 77:24

**Maltezou, H. C.,Vorou, R.,Papadima, K.,Kossyvakis, A.,Spanakis, N.,Gioula, G.,Exindari, M.,Metallidis, S.,Lourida, A. N.,Raftopoulos, V.,Froukala, E.,Martinez-Gonzalez, B.,Mitsianis, A.,Roilides, E.,Mentis, A.,Tsakris, A.,Papa, A.**. Transmission dynamics of SARS-CoV-2 within families with children in Greece: A study of 23 clusters. *Journal of Medical Virology.* 2021. 93:1414-1420

**Marks, M.,Millat-Martinez, P.,Ouchi, D.,Roberts, C. H.,Alemany, A.,Corbacho-Monne, M.,Ubals, M.,Tobias, A.,Tebe, C.,Ballana, E.,Bassat, Q.,Baro, B.,Vall-Mayans, M.,G. Beiras C,Prat, N.,Ara, J.,Clotet, B.,Mitja, O.**. Transmission of COVID-19 in 282 clusters in Catalonia, Spain: a cohort study. *The Lancet Infectious Diseases.* 2021. 21:629-636

**Martinez, D. A.,Klein, E. Y.,Parent, C.,Prieto, D.,Bigelow, B. F.,Saxton, R. E.,Page, K. R.**. Latino Household Transmission of SARS-CoV-2. *Clinical Infectious Diseases.* 2021. 31:31

**McLean, H. Q.,Grijalva, C. G.,Hanson, K. E.,Zhu, Y. G.,Deyoe, J. E.,Meece, J. K.,Halasa, N. B.,Chappell, J. D.,Mellis, A.,Reed, C.,Belongia, E. A.,Talbot, H. K.,Rolfes, M. A.**. Household Transmission and Clinical Features of SARS-CoV-2 Infections by Age in 2 US Communities. *MedRxiv : the Preprint Server for Health Sciences.* 2021. 20:20

**Methi, F., Hart, R. K., Godoy, A. A., Jorgensen, S. B., Kacelnik, O., Telle, K. E.**. Transmission of SARS-CoV-2 into and within immigrant households: nationwide registry study from Norway. *Journal of Epidemiology & Community Health.* 2021. 20:20

**Metlay, J. P.,Haas, J. S.,Soltoff, A. E.,Armstrong, K. A.**. Household Transmission of SARS-CoV-2. *JAMA Network Open.* 2021. 4:e210304

**Miller, E.,Waight, P. A.,Andrews, N. J.,McOwat, K.,Brown, K. E.,Katja, H.,Ijaz, S.,Letley, L.,Haskins, D.,Sinnathamby, M.,Cuthbertson, H.,Hallis, B.,Parimalanathan, V.,de Lusignan, S.,Lopez-Bernal, J.**. Transmission of SARS-CoV-2 in the household setting: A prospective cohort study in children and adults in England. *Journal of Infection.* 2021. 83:483-489

**Montecucco, A., Dini, G., Rahmani, A., Kusznir Vitturi, B., Barletta, C., Pellegrini, L., Manca, A., Orsi, A., Bruzzone, B., Ricucci, V., De Pace, V., Guarona, G., Boccotti, S., Signori, A., Icardi, G., Durando, P.**. Investigating SARS-CoV-2 transmission among co-workers in a University of Northern Italy during COVID-19 pandemic: an observational study. *Medicina del Lavoro.* 2021. 112:429-435

**Musa, S.,Kissling, E.,Valenciano, M.,Dizdar, F.,Blazevic, M.,Joguncic, A.,Palo, M.,Merdrignac, L.,Pebody, R.,Jorgensen, P.**. Household transmission of SARS-CoV-2: a prospective observational study in Bosnia and Herzegovina, August - December 2020. *International Journal of Infectious Diseases.* 2021. 29:29

**Ng, D. C., Tan, K. K., Chin, L., Cheng, X. L., Vijayakulasingam, T., Liew, D. W. X., Zainol Abidin, N. Z., Lee, M. L., Ganasegeran, K., Khoo, E. J.**. Risk factors associated with household transmission of SARS-CoV-2 in Negeri Sembilan, Malaysia. *Journal of Paediatrics & Child Health.* 2021. 01:01

**Ng, O. T., Koh, V., Chiew, C. J., Marimuthu, K., Thevasagayam, N. M., Mak, T. M., Chua, J. K., Ong, S. S. H., Lim, Y. K., Ferdous, Z., Johari, A. K. B., Chen, M. I., Maurer-Stroh, S., Cui, L., Lin, R. T. P., Tan, K. B., Cook, A. R., Leo, P. Y., Lee, P. V. J.**. Impact of Delta Variant and Vaccination on SARS-CoV-2 Secondary Attack Rate Among Household Close Contacts. *The Lancet Regional Health. Western Pacific.* 2021. 17:100299

**Nsekuye, O.,Rwagasore, E.,Muhimpundu, M. A.,El-Khatib, Z.,Ntabanganyimana, D.,Kamayirese, E. N.,Ruyange, L.,Umutoni, A.,Adeline, A. K.,Ntaganira, J.,Nsazimana, S.,Omolo, J.**. Investigation of Four Clusters of Severe Acute Respiratory Syndrome Coronavirus 2 (SARS-CoV-2) in Rwanda, 2020. *International Journal of Environmental Research & Public Health [Electronic Resource].* 2021. 18:30

**Ogata, T.,Irie, F.,Ogawa, E.,Ujiie, S.,Seki, A.,Wada, K.,Tanaka, H.**. Secondary Attack Rate among Non-Spousal Household Contacts of Coronavirus Disease 2019 in Tsuchiura, Japan, August 2020-February 2021. *International Journal of Environmental Research & Public Health [Electronic Resource].* 2021. 18:25

**Palka-Kotlowska, M.,Custodio-Cabello, S.,Oliveros-Acebes, E.,Khosravi-Shahi, P.,Cabezon-Gutierrez, L.**. Review of risk of COVID-19 in cancer patients and their cohabitants. *International Journal of Infectious Diseases.* 2021. 105:15-20

**Paul, L. A.,Daneman, N.,Brown, K. A.,Johnson, J.,van Ingen, T.,Joh, E.,Wilson, S. E.,Buchan, S. A.**. Characteristics associated with household transmission of SARS-CoV-2 in Ontario, Canada: A cohort study. *Clinical Infectious Diseases.* 2021. 05:05

**Paul, L. A.,Daneman, N.,Schwartz, K. L.,Science, M.,Brown, K. A.,Whelan, M.,Chan, E.,Buchan, S. A.**. Association of Age and Pediatric Household Transmission of SARS-CoV-2 Infection. *JAMA Pediatrics.* 2021. 16:16

**Peng, D.,Zhang, J.,Ji, Y.,Pan, D.**. Risk factors for redetectable positivity in recovered COVID-19 children. *Pediatric Pulmonology.* 2020. 55:3602-3609

**Pett, J.,McAleavey, P.,McGurnaghan, P.,Spiers, R.,O'Doherty, M.,Patterson, L.,Johnston, J.**. Epidemiology of COVID-19 in Northern Ireland, 26 February 2020-26 April 2020. *Epidemiology & Infection.* 2021. 149:e36

**Pitzer, V. E.,Cohen, T.**. Household studies provide key insights on the transmission of, and susceptibility to, SARS-CoV-2. *The Lancet Infectious Diseases.* 2020. 20(10):1103-1104

**Pritsch, M.,Radon, K.,Bakuli, A.,Le Gleut, R.,Olbrich, L.,Guggenbuehl Noller, J. M.,Saathoff, E.,Castelletti, N.,Gari, M.,Putz, P.,Schalte, Y.,Frahnow, T.,Wolfel, R.,Rothe, C.,Pletschette, M.,Metaxa, D.,Forster, F.,Thiel, V.,Ries, F.,Diefenbach, M. N.,Froschl, G.,Bruger, J.,Winter, S.,Frese, J.,Puchinger, K.,Brand, I.,Kroidl, I.,Hasenauer, J.,Fuchs, C.,Wieser, A.,Hoelscher, M.,On Behalf Of The KoCo Study, Group**. Prevalence and Risk Factors of Infection in the Representative COVID-19 Cohort Munich. *International Journal of Environmental Research & Public Health [Electronic Resource].* 2021. 18:30

**Pung, R.,Park, M.,Cook, A. R.,Lee, V. J.**. Age-related risk of household transmission of COVID-19 in Singapore. *Influenza & Other Respiratory Viruses.* 2021. 15:206-208

**Rajmohan, P.,Jose, P.,Thodi, J.,Thomas, J.,Raphael, L.,Krishna, S.,Gopinathan, U.,Kuttichira, P.**. Dynamics of transmission of COVID-19 cases and household contacts: A prospective cohort study. *Journal of Acute Disease.* 2021. 10(4):162-168

**Ratovoson, R.,Razafimahatratra, R.,Randriamanantsoa, L.,Raberahona, M.,Rabarison, H. J.,Rahaingovahoaka, F. N.,Andriamasy, E. H.,Herindrainy, P.,Razanajatovo, N.,Andriamandimby, S. F.,Dussart, P.,Schoenhals, M.,Randria, M. J. D.,Heraud, J. M.,Randremanana, R. V.**. Household transmission of COVID-19 among the earliest cases in Antananarivo, Madagascar. *Influenza & Other Respiratory Viruses.* 2021. 10:10

**Reukers, D. F. M.,van Boven, M.,Meijer, A.,Rots, N.,Reusken, C.,Roof, I.,van Gageldonk-Lafeber, A. B.,van der Hoek, W.,van den Hof, S.**. High infection secondary attack rates of SARS-CoV-2 in Dutch households revealed by dense sampling. *Clinical Infectious Diseases.* 2021. 02:02

**Salihefendic, N.,Zildzic, M.,Huseinagic, H.,Ahmetagic, S.,Salihefendic, D.,Masic, I.**. Intrafamilial Spread of COVID-19 Infection Within Population in Bosnia and Herzegovina. *Materia Sociomedica.* 2021. 33:4-9

**Schwartz, K. L.,Achonu, C.,Buchan, S. A.,Brown, K. A.,Lee, B.,Whelan, M.,Wu, J. H.,Garber, G.**. Epidemiology, clinical characteristics, household transmission, and lethality of severe acute respiratory syndrome coronavirus-2 infection among healthcare workers in Ontario, Canada. *PLoS ONE [Electronic Resource].* 2020. 15:e0244477

**Shah, K.,Kandre, Y.,Mavalankar, D.**. Secondary attack rate in household contacts of COVID-19 Paediatric index cases: a study from Western India. *Journal of Public Health.* 2021. 43:243-245

**Singanayagam, A., Hakki, S., Dunning, J., Madon, K. J., Crone, M. A., Koycheva, A., Derqui-Fernandez, N., Barnett, J. L., Whitfield, M. G., Varro, R., Charlett, A., Kundu, R., Fenn, J., Cutajar, J., Quinn, V., Conibear, E., Barclay, W., Freemont, P. S., Taylor, G. P., Ahmad, S., Zambon, M., Ferguson, N. M., Lalvani, A., Investigators, Ataccc Study**. Community transmission and viral load kinetics of the SARS-CoV-2 delta (B.1.617.2) variant in vaccinated and unvaccinated individuals in the UK: a prospective, longitudinal, cohort study. *The Lancet Infectious Diseases.* 2021. 29:29

**Somekh, E.,Gleyzer, A.,Heller, E.,Lopian, M.,Kashani-Ligumski, L.,Czeiger, S.,Schindler, Y.,Lessing, J. B.,Stein, M.**. The role of children in the dynamics of intra family coronavirus 2019 spread in densely populated area. *Pediatric Infectious Disease Journal.* 2020. 39(8):E202-E204

**Soriano-Arandes, A.,Gatell, A.,Serrano, P.,Biosca, M.,Campillo, F.,Capdevila, R.,Fabrega, A.,Lobato, Z.,Lopez, N.,Moreno, A. M.,Poblet, M.,Riera-Bosch, M. T.,Rius, N.,Ruiz, M.,Sanchez, A.,Valldeperez, C.,Vila, M.,Pineda, V.,Lazcano, U.,Diaz, Y.,Reyes-Uruena, J.,Soler-Palacin, P.**. Household SARS-CoV-2 transmission and children: a network prospective study. *Clinical infectious diseases : an official publication of the Infectious Diseases Society of America..* 2021. 12:#pages#

**Stich, M., Elling, R., Renk, H., Janda, A., Garbade, S. F., Muller, B., Krausslich, H. G., Fabricius, D., Zernickel, M., Meissner, P., Huzly, D., Grulich-Henn, J., Haddad, A., Gorne, T., Spielberger, B., Fritsch, L., Nieters, A., Hengel, H., Dietz, A. N., Stamminger, T., Ganzenmueller, T., Ruetalo, N., Peter, A., Remppis, J., Iftner, T., Jeltsch, K., Waterboer, T., Franz, A. R., Hoffmann, G. F., Engel, C., Debatin, K. M., Tonshoff, B., Henneke, P.**. Transmission of Severe Acute Respiratory Syndrome Coronavirus 2 in Households with Children, Southwest Germany, May-August 2020. *Emerging Infectious Diseases.* 2021. 27:3009-3019

**Tanaka, M. L., Marentes Ruiz, C. J., Malhotra, S., Turner, L., Peralta, A., Lee, Y., Jumarang, J., Perez, S. E., Navarro, J., Dien Bard, J., Gordon, A., Allen, E. K., Thomas, P. G., Pannaraj, P. S.**. SARS-CoV-2 Transmission Dynamics in Households With Children, Los Angeles, California. *Frontiers in Pediatrics.* 2021. 9:752993

**Telle, K.,Jorgensen, S. B.,Hart, R.,Greve-Isdahl, M.,Kacelnik, O.**. Secondary attack rates of COVID-19 in Norwegian families: a nation-wide register-based study. *European Journal of Epidemiology.* 2021. 36:741-748

**Villarino, E.,Deng, X.,Kemper, C. A.,Jorden, M. A.,Bonin, B.,Rudman, S. L.,Han, G. S.,Yu, G.,Wang, C.,Federman, S.,Bushnell, B.,C. Z. B. COVIDTracker Consortium,Wadford, D. A.,Lin, W.,Tao, Y.,Paden, C. R.,Bhatnagar, J.,MacCannell, T.,Tong, S.,Batson, J.,Chiu, C. Y.**. Introduction, Transmission Dynamics, and Fate of Early Severe Acute Respiratory Syndrome Coronavirus 2 Lineages in Santa Clara County, California. *Journal of Infectious Diseases.* 2021. 224:207-217

**Wang, Z.,Ma, W.,Zheng, X.,Wu, G.,Zhang, R.**. Household transmission of SARS-CoV-2. *Journal of Infection.* 2020. 81(1):179-182

**Wilkinson, K.,Chen, X.,Shaw, S.**. Secondary attack rate of COVID-19 in household contacts in the Winnipeg Health Region, Canada. *Canadian Journal of Public Health. Revue Canadienne de Sante Publique.* 2021. 112:12-16

**Wu, J.,Huang, Y.,Tu, C.,Bi, C.,Chen, Z.,Luo, L.,Huang, M.,Chen, M.,Tan, C.,Wang, Z.,Wang, K.,Liang, Y.,Huang, J.,Zheng, X.,Liu, J.**. Household Transmission of SARS-CoV-2, Zhuhai, China, 2020. *Clinical Infectious Diseases.* 2020. 71:2099-2108

**Xiao, F.,Chen, B.,Xiao, T.,Lee, S. K.,Yan, K.,Hu, L.**. Children with SARS-CoV-2 infection during an epidemic in China (outside of Hubei province). *Annals of Translational Medicine.* 2020. 8(14) (no pagination):#pages#

**Xin, H.,Jiang, F.,Xue, A.,Liang, J.,Zhang, J.,Yang, F.,Han, Y.**. Risk factors associated with occurrence of COVID-19 among household persons exposed to patients with confirmed COVID-19 in Qingdao Municipal, China. *Transboundary & Emerging Diseases.* 2021. 68:782-788

**Yan, X.,Wang, J.,Yao, J.,Estill, J.,Wu, S.,Lu, J.,Liang, B.,Li, H.,Tao, S.,Bai, H.,Liu, H.,Chen, Y.,Covid- evidence,recommendations working, group**. A cross-sectional study of the epidemic situation on COVID-19 in Gansu Province, China - a big data analysis of the national health information platform. *BMC Infectious Diseases.* 2021. 21:146

**Yang, M. C.,Hung, P. P.,Wu, Y. K.,Peng, M. Y.,Chao, Y. C.,Su, W. L.**. A three-generation family cluster with COVID-19 infection: should quarantine be prolonged?. *Public Health.* 2020. 185:31-33

**Yang, S.,Feng, X.,Du, P.,He, D.,Estill, J.,Yang, L.,Wen, C.,Luo, J.,Wang, X.,Lu, L.,Li, T.,Tang, X.,Li, W.,Xu, H.,Liu, E.,Cao, P.,Chen, Y.**. Family exposure and the impact of containment measures to children with coronavirus disease 2019 outside Hubei, China: a cross-sectional study. *Translational Pediatrics.* 2021. 10(1):92-102

**Yao, L.,Tang, P.,Jiang, H.,Gu, B.,Xu, P.,Wang, X.,Yu, X.,Zhang, J.,Pang, Y.,Wu, M.**. Household Clusters of Severe Acute Respiratory Syndrome Coronavirus 2 (SARS-CoV-2) Infection in Suzhou, China. *BioMed Research International.* 2021. 2021:5565549

**Yi, B.,Fen, G.,Cao, D.,Cai, Y.,Qian, L.,Li, W.,Wen, Z.,Sun, X.**. Epidemiological and clinical characteristics of 214 families with COVID-19 in Wuhan, China. *International Journal of Infectious Diseases.* 2021. 105:113-119

**Brinkley-Rubinstein, L.,LeMasters, K.,Nguyen, P.,Nowotny, K.,Cloud, D.,Volfovsky, A.**. The association between intersystem prison transfers and COVID-19 incidence in a state prison system. *PLoS ONE [Electronic Resource].* 2021. 16:e0256185

**Crispim, J. A.,Ramos, A. C. V.,Berra, T. Z.,Santos, M. S. D.,Santos, F. L. D.,Alves, L. S.,Costa, Fbpd,Arcencio, R. A.**. Impact and trend of COVID-19 in the Brazilian prison system: an ecological study. *Ciencia & Saude Coletiva.* 2021. 26:169-178

**Dunne, E. M.,Morgan, E.,Wells-Moore, B.,Pierson, S.,Zakroff, S.,Haskell, L.,Link, K.,Powell, J.,Holland, I.,Elgethun, K.,Ball, C.,Haugen, R.,Hahn, C. G.,Carter, K. K.,Starr, C.**. COVID-19 Outbreaks in Correctional Facilities with Work-Release Programs - Idaho, July-November 2020. *MMWR - Morbidity & Mortality Weekly Report.* 2021. 70:589-594

**Mahale, P.,Rothfuss, C.,Bly, S.,Kelley, M.,Bennett, S.,Huston, S. L.,Robinson, S.**. Multiple COVID-19 Outbreaks Linked to a Wedding Reception in Rural Maine - August 7-September 14, 2020. *MMWR - Morbidity & Mortality Weekly Report.* 2020. 69:1686-1690

**Morgan, R. C.,Bailey, R. K.,Knox, L.,Reid, T. N.**. Prisons and Immigrant Detention Centers as Structural Determinants of Coronavirus Transmission and Case Severity. *Journal of the National Medical Association.* 2020. 112(5 Supplement):S21

**Nomani, M. Z. M.,Hussain, Z.**. Health care in prisons and detention homes during COVID-19 pandemic in India. *European Journal of Molecular and Clinical Medicine.* 2021. 8(1):1488-1492

**Ward, J. A.,Parish, K.,DiLaura, G.,Dolovich, S.,Saloner, B.**. COVID-19 Cases Among Employees of U.S. Federal and State Prisons. *American Journal of Preventive Medicine.* 2021. 60:840-844

**Cossaboom, C. M.,Medley, A. M.,Spengler, J. R.,Kukielka, E. A.,Goryoka, G. W.,Baird, T.,Bhavsar, S.,Campbell, S.,Campbell, T. S.,Christensen, D.,Condrey, J. A.,Dawson, P.,Doty, J. B.,Feldpausch, A.,Gabel, J.,Jones, D.,Lim, A.,Loiacono, C. M.,Jenkins-Moore, M.,Moore, A.,Noureddine, C.,Ortega, J.,Poulsen, K.,Rooney, J. A.,Rossow, J.,Sheppard, K.,Sweet, E.,Stoddard, R.,Tell, R. M.,Wallace, R. M.,Williams, C.,Barton Behravesh, C.**. Low SARS-CoV-2 Seroprevalence and No Active Infections among Dogs and Cats in Animal Shelters with Laboratory-Confirmed COVID-19 Human Cases among Employees. *Biology.* 2021. 10:11

**Molyneaux, A., Hankinson, E., Kaban, M., Svensson, M. S., Cheyne, S. M., Nijman, V.**. Primate Selfies and Anthropozoonotic Diseases: Lack of Rule Compliance and Poor Risk Perception Threatens Orangutans. *Folia Primatologica.* 2021. 25:25

**Kim, C.,Kim, Y. M.,Heo, N.,Park, E.,Choi, S.,Kim, N.,Kown, D.,Park, Y. J.,Choi, B.,Ha, B.,Jeong, G.,Park, C.,Park, S.,Lee, H.**. COVID-19 Outbreak in a Military Unit in Korea. *Epidemiology and health.* 2021. #volume#:e2021065

**Lalani, T.,Lee, T. K.,Laing, E. D.,Ritter, A.,Cooper, E.,Lee, M.,Baker, M.,Baldino, T.,McAdoo, T.,Phogat, S.,Samuels, E.,Nguyen, H.,Broder, C. C.,Epsi, N.,Richard, S. A.,Warkentien, T. E.,Millar, E. V.,Burgess, T.,Kronmann, K. C.**. SARS-CoV-2 Infections and Serologic Responses among Military Personnel Deployed on the USNS COMFORT to New York City during the COVID-19 Pandemic. *Open Forum Infectious Diseases.* 2021. 8(2) (no pagination):#pages#

**Letizia, A. G.,Ramos, I.,Obla, A.,Goforth, C.,Weir, D. L.,Ge, Y.,Bamman, M. M.,Dutta, J.,Ellis, E.,Estrella, L.,George, M. C.,Gonzalez-Reiche, A. S.,Graham, W. D.,van de Guchte, A.,Gutierrez, R.,Jones, F.,Kalomoiri, A.,Lizewski, R.,Lizewski, S.,Marayag, J.,Marjanovic, N.,Millar, E. V.,Nair, V. D.,Nudelman, G.,Nunez, E.,Pike, B. L.,Porter, C.,Regeimbal, J.,Rirak, S.,Santa Ana, E.,Sealfon, R. S. G.,Sebra, R.,Simons, M. P.,Soares-Schanoski, A.,Sugiharto, V.,Termini, M.,Vangeti, S.,Williams, C.,Troyanskaya, O. G.,van Bakel, H.,Sealfon, S. C.**. SARS-CoV-2 Transmission among Marine Recruits during Quarantine. *New England Journal of Medicine.* 2020. 383:2407-2416

**Marcus, J. E.,Frankel, D. N.,Pawlak, M. T.,Casey, T. M.,Cybulski, R. J., Jr.,Enriquez, E.,Okulicz, J. F.,Yun, H. C.**. Risk Factors Associated With COVID-19 Transmission Among US Air Force Trainees in a Congregant Setting. *JAMA Network Open.* 2021. 4:e210202

**Rocha, A. L. S.,Pinheiro, J. R.,Nakamura, T. C.,da Silva, J. D. S.,Rocha, B. G. S.,Klein, R. C.,Birbrair, A.,Amorim, J. H.**. Fomites and the environment did not have an important role in COVID-19 transmission in a Brazilian mid-sized city. *Scientific Reports.* 2021. 11:15960

**Yuan, J.,Chen, Z.,Gong, C.,Liu, H.,Li, B.,Li, K.,Chen, X.,Xu, C.,Jing, Q.,Liu, G.,Qin, P.,Liu, Y.,Zhong, Y.,Huang, L.,Zhu, B. P.,Yang, Z.**. Sewage as a Possible Transmission Vehicle During a Coronavirus Disease 2019 Outbreak in a Densely populated Community: Guangzhou, China, April 2020. *Clinical infectious diseases : an official publication of the Infectious Diseases Society of America..* 2020. 12:#pages#

**Zuckerman, N. S.,Pando, R.,Bucris, E.,Drori, Y.,Lustig, Y.,Erster, O.,Mor, O.,Mendelson, E.,Mandelboim, M.**. Comprehensive Analyses of SARS-CoV-2 Transmission in a Public Health Virology Laboratory. *Viruses.* 2020. 12:05

# **Modes of transmission:**

**Allen, N.,Ni Riain, U.,Conlon, N.,Ferenczi, A.,Carrion Martin, A. I.,Domegan, L.,Walsh, C.,Doherty, L.,O'Farrelly, C.,Higgins, E.,Kerr, C.,McGrath, J.,Fleming, C.,Bergin, C.**. Prevalence of Antibodies to SARS-CoV-2 in Irish Hospital Healthcare Workers. *Epidemiology and Infection..* 2021. #volume#:#pages#

**Almadhi, M. A.,Abdulrahman, A.,Sharaf, S. A.,AlSaad, D.,Stevenson, N. J.,Atkin, S. L.,AlQahtani, M. M.**. The high prevalence of asymptomatic SARS-CoV-2 infection reveals the silent spread of COVID-19. *International Journal of Infectious Diseases.* 2021. 105:656-661

**Anand, P.,Allen, H. L.,Ferrer, R. L.,Gold, N.,Gonzales Martinez, R. M.,Kontopantelis, E.,Krause, M.,Vergunst, F.**. Work-related and personal predictors of COVID-19 transmission: evidence from the UK and USA. *Journal of epidemiology and community health..* 2021. 12:#pages#

**Arons, M. M.,Hatfield, K. M.,Reddy, S. C.,Kimball, A.,James, A.,Jacobs, J. R.,Taylor, J.,Spicer, K.,Bardossy, A. C.,Oakley, L. P.,Tanwar, S.,Dyal, J. W.,Harney, J.,Chisty, Z.,Bell, J.,Methner, M.,Paul, P.,Carlson, C. M.,McLaughlin, H. P.,Thornburg, N.,Tong, S.,Tamin, A.,Tao, Y.,Uehara, A.,Harcourt, J.,Clark, S.,Brostrom-Smith, C.,Page, L. C.,Kay, M.,Lewis, J.,Montgomery, P.,Stone, N. D.,Clark, T. A.,Honein, M. A.,Duchin, J. S.,Jernigan, J. A.**. Presymptomatic SARS-CoV-2 infections and transmission in a skilled nursing facility. *New England Journal of Medicine.* 2020. 382(22):2081-2090

**Backhaus, I., Hermsen, D., Timm, J., Boege, F., Lubke, N., Gobels, K., von der Lieth, D., Dragano, N.**. Underascertainment of COVID-19 cases among first responders: a seroepidemiological study. *Occupational Medicine.* 2021. 09:09

**Bae, S. H.,Shin, H.,Koo, H. Y.,Lee, S. W.,Yang, J. M.,Yon, D. K.**. Asymptomatic Transmission of SARS-CoV-2 on Evacuation Flight. *Emerging Infectious Diseases.* 2020. 26:2705-2708

**Beale, S.,Braithwaite, I.,Navaratnam, A. M.,Hardelid, P.,Rodger, A.,Aryee, A.,Byrne, T. E.,Fong, E. W. L.,Fragaszy, E.,Geismar, C.,Kovar, J.,Nguyen, V.,Patel, P.,Shrotri, M.,Aldridge, R.,Hayward, A.,Virus Watch, Collaborative**. Deprivation and exposure to public activities during the COVID-19 pandemic in England and Wales. *Journal of Epidemiology & Community Health.* 2021. 12:12

**Boutzoukas, A. E., Zimmerman, K. O., Benjamin, D. K., DeMuri, G. P., Kalu, I. C., Smith, M. J., McGann, K. A., Koval, S., Brookhart, M. A., Butteris, S. M.**. Secondary Transmission of COVID-19 in K-12 Schools: Findings From 2 States. *Pediatrics.* 2022. 149 (no pagination):#pages#

**Bullard, J.,Funk, D.,Dust, K.,Garnett, L.,Tran, K.,Bello, A.,Strong, J. E.,Lee, S. J.,Waruk, J.,Hedley, A.,Alexander, D.,Van Caeseele, P.,Loeppky, C.,Poliquin, G.**. Infectivity of severe acute respiratory syndrome coronavirus 2 in children compared with adults. *CMAJ Canadian Medical Association Journal.* 2021. 193:E601-E606

**Burn, E.,Tebe, C.,Fernandez-Bertolin, S.,Aragon, M.,Recalde, M.,Roel, E.,Prats-Uribe, A.,Prieto-Alhambra, D.,Duarte-Salles, T.**. The natural history of symptomatic COVID-19 during the first wave in Catalonia. *Nature communications.* 2021. 12:777

**Chen, D.,Hu, C.,Su, F.,Song, Q.,Wang, Z.**. Exposure to SARS-CoV-2 in a high transmission setting increases the risk of severe COVID-19 compared with exposure to a low transmission setting?. *Journal of Travel Medicine.* 2020. 27:#pages#

**Coppeta, L., Ferrari, C., Mazza, A., Trabucco Aurilio, M., Rizza, S.**. Factors associated with pre-vaccination sars-cov-2 infection risk among hospital nurses facing covid-19 outbreak. *International Journal of Environmental Research and Public Health.* 2021. 18(24) (no pagination):#pages#

**Daubert, G.,Gillet, G.,Guet, L.,Marini, H.,Merle, V.**. General Practitioners' Practice premises and Risk of Viral Cross-Transmission: A French Observational Multicenter Study. *Journal of Primary Care & Community Health.* 2021. 12:21501327211043734

**Dinh, C.,Gallouche, M.,Terrisse, H.,Gam, K.,Giner, C.,Giai, J.,Bosson, J. L.,Lambert-Lacroix, S.,Landelle, C.**. Risk factors of nosocomial COVID-19 at Grenoble Alpes university hospital. *Antimicrobial Resistance and Infection Control. Conference: 6th International Conference on Prevention and Infection Control, ICPIC.* 2021. 10:#pages#

**Djuric, O., Pepe, D., Vicentini, M., Bonvicini, L., Rossi, P. G., Pezzotti, P., Urdiales, A. M., Marino, M., Formisano, D., Formoso, G., Bedeschi, E., Perilli, C., Venturi, I., Bisaccia, E., Larosa, E., Cassinadri, M., Cilloni, S., Campari, C., Gioia, F., Broccoli, S., Ottone, M., Pattacini, P., Besutti, G., Iotti, V., Spaggiari, L., Mancuso, P., Nitrosi, A., Foracchia, M., Colla, R., Zerbini, A., Massari, M., Ferrari, A. M., Pinotti, M., Facciolongo, N., Lattuada, I., Trabucco, L., de Pietri, S., Danelli, G. F., Albertazzi, L., Bellesia, E., Canovi, S., Corradini, M., Fasano, T., Magnani, E., Pilia, A., Polese, A., Incerti, S. S., Zaldini, P., Bonelli, E., Orsola, B., Revelli, M., Salvarani, C., Venturelli, F.**. Secondary transmission of SARS-CoV-2 in educational settings in Northern Italy from September 2020 to April 2021: a population-based study. *medRxiv..* 2021. 06:#pages#

**Gilliam, W. S.,Malik, A. A.,Shafiq, M.,Klotz, M.,Reyes, C.,Humphries, J. E.,Murray, T.,Elharake, J. A.,Wilkinson, D.,Omer, S. B.**. COVID-19 transmission in US child care programs. *Pediatrics.* 2021. 147(1) (no pagination):#pages#

**Graham, N. S. N.,Junghans, C.,Downes, R.,Sendall, C.,Lai, H.,McKirdy, A.,Elliott, P.,Howard, R.,Wingfield, D.,Priestman, M.,Ciechonska, M.,Cameron, L.,Storch, M.,Crone, M. A.,Freemont, P. S.,Randell, P.,McLaren, R.,Lang, N.,Ladhani, S.,Sanderson, F.,Sharp, D. J.**. SARS-CoV-2 infection, clinical features and outcome of COVID-19 in United Kingdom nursing homes. *Journal of Infection.* 2020. 81(3):411-419

**Hancean, M. G.,Perc, M.,Lerner, J.**. Early spread of COVID-19 in Romania: imported cases from Italy and human-to-human transmission networks. *Royal Society Open Science.* 2020. 7:200780

**Hwang, M.,Bae, J. M.**. Sources of Infection Among Confirmed Cases of COVID-19 in Jeju Province, Korea. *Journal of Preventive Medicine & Public Health / Yebang Uihakhoe Chi.* 2021. 54:245-250

**Jeewandara, C., Guruge, D., Jayathilaka, D., Deshan Madhusanka, P. A., Pushpakumara, P. D., Tanussiya Ramu, S., Sepali Aberathna, I., Saubhagya Rasikangani Danasekara, D. R., Pathmanathan, T., Gunatilaka, B., Malavige, S., Dias, Y., Wijayamuni, R., Ogg, G. S., Malavige, G. N.**. Transmission dynamics, clinical characteristics and sero-surveillance in the COVID-19 outbreak in a population dense area of Colombo, Sri Lanka April- May 2020. *PLoS ONE [Electronic Resource].* 2021. 16:e0257548

**Jinadatha, C.,Jones, L. D.,Choi, H.,Chatterjee, P.,Hwang, M.,Redmond, S. N.,Navas, M. E.,Zabarsky, T. F.,Bhullar, D.,Cadnum, J. L.,Donskey, C. J.**. Transmission of SARS-CoV-2 in Inpatient and Outpatient Settings in a Veterans Affairs Health Care System. *Open Forum Infectious Diseases.* 2021. 8:ofab328

**Jones, B.,Phillips, G.,Kemp, S.,Payne, B.,Hart, B.,Cross, M.,Stokes, K. A.**. SARS-CoV-2 transmission during rugby league matches: do players become infected after participating with SARS-CoV-2 positive players?. *British Journal of Sports Medicine.* 2021. 55:807-813

**Kakimoto, K., Kamiya, H., Yamagishi, T., Matsui, T., Suzuki, M., Wakita, T.**. Initial Investigation of Transmission of COVID-19 Among Crew Members During Quarantine of a Cruise Ship - Yokohama, Japan, February 2020. *MMWR Morb Mortal Wkly Rep.* 2020. 69:312-313

**Kriger, O.,Lustig, Y.,Cohen, C.,Amit, S.,Biber, A.,Barkai, G.,Talmi, L.,Gefen-Halevi, S.,Mechnik, B.,Regev-Yochay, G.**. The Sheba Medical Center healthcare workers' children's school: can we open schools safely?. *Clinical Microbiology and Infection.* 2021. 27(3):474.e1-474.e3

**Mack, C. D.,DiFiori, J.,Tai, C. G.,Shiue, K. Y.,Grad, Y. H.,Anderson, D. J.,Ho, D. D.,Sims, L.,LeMay, C.,Mancell, J.,Maragakis, L. L.**. SARS-CoV-2 Transmission Risk Among National Basketball Association Players, Staff, and Vendors Exposed to Individuals With Positive Test Results After COVID-19 Recovery During the 2020 Regular and Postseason. *JAMA Internal Medicine.* 2021. 181:960-966

**Martinez-Baz, I., Trobajo-Sanmartin, C., Burgui, C., Casado, I., Castilla, J.**. Transmission of SARS-CoV-2 infection and risk factors in a cohort of close contacts. *Postgraduate Medicine.* 2022. #volume#:1-9

**Masarone, M.,Vaccaro, E.,Sciorio, R.,Torre, P.,Della Vecchia, A.,Aglitti, A.,Caliulo, R.,Borrelli, A.,Persico, M.**. Characterisation of asymptomatic patients and efficacy of preventive measures for SARS-CoV-2 infection in a large population of Southern Italy: A cohort study. *BMJ Open.* 2021. 11(5) (no pagination):#pages#

**Michelle, Murti, Camille, Achonu, Brendan, T. Smith, Kevin, A. Brown, Jin Hee, Kim, James, Johnson, Saranyah, Ravindran, Sarah, A. Buchan**. COVID-19 Workplace Outbreaks by Industry Sector and their Associated Household Transmission, Ontario, Canada, January to June, 2020. *#journal#.* 2020. #volume#:#pages#

**Mirjam Jeanne Dorine, Dautzenberg, Andrea, Eikelenboom-Boskamp, Jacqueline, Janssen, Miranda, Drabbe, Ewoud de, Jong, Eefke, Weesendorp, Marion, Koopmans, Andreas, Voss**. Healthcare workers in elderly care: a source of silent SARS-CoV-2 transmission?. *#journal#.* 2020. #volume#:#pages#

**Mo, Y.,Eyre, D. W.,Lumley, S. F.,Walker, T. M.,Shaw, R. H.,O'Donnell, D.,Butcher, L.,Jeffery, K.,Donnelly, C. A.,Cooper, B. S.**. Transmission of community- And hospital-acquired SARS-CoV-2 in hospital settings in the UK: A cohort study. *PLoS Medicine.* 2021. 18(10) (no pagination):#pages#

**Murewanhema, G.,Burukai, T. V.,Chiwaka, L.,Maunganidze, F.,Munodawafa, D.,Pote, W.,Mufunda, J.**. The effect of increased mobility on SARS-CoV-2 transmission: a descriptive study of the trends of COVID-19 in Zimbabwe between December 2020 and January 2021. *The Pan African medical journal.* 2021. 39:125

**Papanikolaou, I. S.,Tziatzios, G.,Chatzidakis, A.,Facciorusso, A.,Crino, S. F.,Gkolfakis, P.,Deriban, G.,Tadic, M.,Hauser, G.,Vezakis, A.,Jovanovic, I.,Muscatiello, N.,Meneghetti, A.,Miltiadou, K.,Stardelova, K.,Lackovic, A.,Bourou, M. Z.,Djuranovic, S.,Triantafyllou, K.**. COVID-19 in the endoscopy unit: How likely is transmission of infection? Results from an international, multicenter study. *World Journal of Gastrointestinal Endoscopy.* 2021. 13:416-425

**Plucinski, M. M., Wallace, M., Uehara, A., Kurbatova, E. V., Tobolowsky, F. A., Schneider, Z. D., Ishizumi, A., Bozio, C. H., Kobayashi, M., Toda, M., Stewart, A., Wagner, R. L., Moriarty, L. F., Murray, R., Queen, K., Tao, Y., Paden, C., Mauldin, M. R., Zhang, J., Li, Y., Elkins, C. A., Lu, X., Herzig, C. T. A., Novak, R., Bower, W., Medley, A. M., Acosta, A. M., Knust, B., Cantey, P. T., Pesik, N. T., Halsey, E. S., Cetron, M. S., Tong, S., Marston, B. J., Friedman, C. R.**. Coronavirus Disease 2019 (COVID-19) in Americans Aboard the Diamond Princess Cruise Ship. *Clin Infect Dis.* 2021. 72:e448-e457

**Rathish, B., Wilson, A., Warrier, A., Prakash, S., Babu, R., Joy, S., Bhattacharjee, S.**. Analysis of an outbreak of nosocomial COVID-19 at a tertiary care centre in South India. *Journal of the Royal College of Physicians of Edinburgh.* 2021. 51:332-337

**Safer, M.,Letaief, H.,Hechaichi, A.,Harizi, C.,Dhaouadi, S.,Bouabid, L.,Darouiche, S.,Gharbi, D.,Elmili, N.,Ben Salah, H.,Hammami, M.,Talmoudi, K.,Moussa, R.,Charaa, N.,Termiz, H.,Ltaief, F.,Tounekti, H.,Makhlouf, M.,Belguith Sriha, A.,Ben Fredj, M.,Khalfallah, S.,Jabrane, H.,McHirgui, S.,Amich, C.,Dabghi, R.,Anez, Z.,Abdelkader, L.,Mhamdi, M.,Ouerfeli, N.,Zoghlami, S.,Bougatef, S.,Chahed, M. K.,Bouafif Ben Alaya, N.**. Identification of transmission chains and clusters associated with COVID-19 in Tunisia. *BMC Infectious Diseases.* 2021. 21:453

**Satharasinghe, D. A.,Parakatawella, Pmsdk,Premarathne, Jmkjk,Jayasooriya, Ljpap,Prathapasinghe, G. A.,Yeap, S. K.**. Evolutionary and genomic analysis of four SARS-CoV-2 isolates circulating in March 2020 in Sri Lanka; Additional evidence on multiple introduction and further transmission. *Epidemiology & Infection.* 2021. 149:e78

**Schneider, S.,Piening, B.,Nouri-Pasovsky, P. A.,Kruger, A. C.,Gastmeier, P.,Aghdassi, S. J. S.**. SARS-Coronavirus-2 cases in healthcare workers may not regularly originate from patient care: lessons from a university hospital on the underestimated risk of healthcare worker to healthcare worker transmission. *Antimicrobial Resistance and Infection Control.* 2020. 9(1) (no pagination):#pages#

**Shamez, N. Ladhani, Georgina, Ireland, Frances, Baawuah, Joanne, Beckmann, Ifeanichukwu, O. Okike, Shazaad, Ahmad, Joanna, Garstang, Andrew, J. Brent, Bernadette, Brent, Felicity, Aiano, Zahin, Amin-Chowdhury, Meaghan, Kall, Ray, Borrow, Ezra, Linley, Maria, Zambon, John, Poh, Lenesha, Warrener, Angie, Lackenby, Joanna, Ellis, Gayatri, Amirthalingam, Kevin, E. Brown, Mary, E. Ramsay**. Emergence of the Delta Variant and risk of SARS-CoV-2 infection in secondary school students and staff: prospective surveillance in 18 schools, England. *#journal#.* 2021. #volume#:#pages#

**Sikkens, J. J.,Buis, D. T. P.,Peters, E. J. G.,Dekker, M.,Schinkel, M.,Reijnders, T. D. Y.,Schuurman, A. R.,de Brabander, J.,Lavell, A. H. A.,Maas, J. J.,Koopsen, J.,Han, A. X.,Russell, C. A.,Schinkel, J.,Jonges, M.,Matamoros, S.,Jurriaans, S.,van Mansfeld, R.,Wiersinga, W. J.,Smulders, Y. M.,de Jong, M. D.,Bomers, M. K.**. Serologic Surveillance and Phylogenetic Analysis of SARS-CoV-2 Infection Among Hospital Health Care Workers. *JAMA Network Open.* 2021. 4:e2118554

**Souch, J. M.,Cossman, J. S.,Hayward, M. D.**. Interstates of Infection: Preliminary Investigations of Human Mobility Patterns in the COVID-19 Pandemic. *Journal of Rural Health.* 2021. 37:266-271

**Taylor, C. A.,Boulos, C.,Almond, D.**. Livestock plants and COVID-19 transmission. *Proceedings of the National Academy of Sciences of the United States of America.* 2020. 117:31706-31715

**Telford, C. T.,Bystrom, C.,Fox, T.,Holland, D. P.,Wiggins-Benn, S.,Mandani, A.,McCloud, M.,Shah, S.**. COVID-19 Infection Prevention and Control Adherence in Long-Term Care Facilities, Atlanta, Georgia. *Journal of the American Geriatrics Society.* 2021. 69:581-586

**Thompson, J. W., Jr.,Mikolajewski, A. J.,Kissinger, P.,McCrossen, P.,Smither, A.,Chamarthi, G. D.,Lin, Z.,Tian, D.**. An Epidemiologic Study of COVID-19 Patients in a State Psychiatric Hospital: High Penetrance With Early CDC Guidelines. *Psychiatric Services.* 2020. 71:1285-1287

**Tucek, M.**. COVID-19 in the Czech Republic 2020: probable transmission of the coronavirus SARS-CoV-2. *Central European Journal of Public Health.* 2021. 29:159-161

**Venkatachalam, I.,Conceicao, E. P.,Aung, M. K.,How, M. K. B.,Wee, L. E.,Sim, J. X. Y.,Tan, B. H.,Ling, M. L.**. Healthcare workers as a sentinel surveillance population in the early phase of the COVID-19 pandemic. *Singapore Medical Journal.* 2021. 22:22

**Vijh, R., Ng, C. H., Shirmaleki, M., Bharmal, A.**. Factors associated with transmission of COVID-19 in long-term care facility outbreaks. *Journal of Hospital Infection..* 2021. #volume#:#pages#

**Vivier, E.,Pariset, C.,Rio, S.,Armand, S.,Doroszewski, F.,Richard, D.,Chardon, M.,Romero, G.,Metral, P.,Pecquet, M.,Didelot, A.**. Specific exposure of ICU staff to SARS-CoV-2 seropositivity: a wide seroprevalence study in a French city-center hospital. *Annals of Intensive Care.* 2021. 11(1) (no pagination):#pages#

**Wang, P.,Lian, Z.,Chen, Y.,Qi, Y.,Chen, H.,An, X.**. Investigation of a Cluster of 2019 Novel Coronavirus Disease (COVID-19) with Possible Transmission During the Incubation Period - Shenyang City, China, 2020. *China CDC Weekly.* 2020. 2:125-127

**Wang, Y.,Chen, R.,Hu, F.,Lan, Y.,Yang, Z.,Zhan, C.,Shi, J.,Deng, X.,Jiang, M.,Zhong, S.,Liao, B.,Deng, K.,Tang, J.,Guo, L.,Jiang, M.,Fan, Q.,Li, M.,Liu, J.,Shi, Y.,Deng, X.,Xiao, X.,Kang, M.,Li, Y.,Guan, W.,Li, Y.,Li, S.,Li, F.,Zhong, N.,Tang, X.**. Transmission, viral kinetics and clinical characteristics of the emergent SARS-CoV-2 Delta VOC in Guangzhou, China. *EClinicalMedicine.* 2021. 40:101129

**Wee, L. E. I.,Sim, X. Y. J.,Conceicao, E. P.,Aung, M. K.,Tan, K. Y.,Ko, K. K. K.,Wong, H. M.,Wijaya, L.,Tan, B. H.,Venkatachalam, I.,Ling, M. L.**. Containing COVID-19 outside the isolation ward: The impact of an infection control bundle on environmental contamination and transmission in a cohorted general ward. *American Journal of Infection Control.* 2020. 48(9):1056-1061

**Wong, J. C. C.,Hapuarachchi, H. C.,Arivalan, S.,Tien, W. P.,Koo, C.,Mailepessov, D.,Kong, M.,Nazeem, M.,Lim, M.,Ng, L. C.**. Environmental contamination of SARS-CoV-2 in a non-healthcare setting. *International Journal of Environmental Research and Public Health.* 2021. 18(1):1-10

**Yuan, Y.,He, J.,Gong, L.,Li, W.,Jiang, L.,Liu, J.,Chen, Q.,Yu, J.,Hou, S.,Shi, Y.,Lu, S.,Zhang, Z.,Ge, Y.,Sa, N.,He, L.,Wu, J.,Sun, Y.,Liu, Z.**. Molecular epidemiology of SARS-CoV-2 clusters caused by asymptomatic cases in Anhui Province, China. *BMC Infectious Diseases.* 2020. 20:930

**Avwioro, G. O., Egwunyenga, A., Iyiola, S., Odibo, E., Onyije, F., Oyinbo, C. A., Avwioro, T., Enitan, S. S., Mgbere, O.**. Commercial motorcycle operators pose high risk for community transmission of coronavirus disease 2019 (COVID-19) in South-South Nigeria. *Scienceafrique.* 2022. 15:e01065

**Barocas, J. A.,Blackstone, E.,Bouton, T. C.,Kimmel, S. D.,Caputo, A.,Porter, S. J.,Walley, A. Y.**. Prevalence of Covid-19 Infection and Subsequent Cohorting in a Residential Substance Use Treatment Program in Boston, MA. *Journal of Addiction Medicine.* 2020. 14:e261-e263

**Braun, K. M.,Moreno, G. K.,Buys, A.,Somsen, E. D.,Bobholz, M.,Accola, M. A.,Anderson, L.,Rehrauer, W. M.,Baker, D. A.,Safdar, N.,Lepak, A. J.,O'Connor, D. H.,Friedrich, T. C.**. Viral sequencing reveals US healthcare personnel rarely become infected with SARS-CoV-2 through patient contact. *Clinical infectious diseases : an official publication of the Infectious Diseases Society of America..* 2021. 15:#pages#

**Chong, D. W., Jayaraj, V. J., Ng, C. W., Sam, I. C., Said, M. A., Ahmad Zaki, R., Hairi, N. N., Nik Farid, N. D., Hoe, V. C., Isahak, M., Ponnampalavanar, S., Syed Omar, S. F., Kamaruzzaman, S. B., Ong, H. C., Hasmukharay, K., Hasnan, N., Kamarulzaman, A., Chan, Y. F., Chong, Y. M., Rampal, S.**. Propagation of a hospital-associated cluster of COVID-19 in Malaysia. *BMC Infectious Diseases.* 2021. 21:1238

**Coleman, K. K.,Tay, D. J. W.,Sen Tan, K.,Ong, S. W. X.,Son, T. T.,Koh, M. H.,Chin, Y. Q.,Nasir, H.,Mak, T. M.,Chu, J. J. H.,Milton, D. K.,Chow, V. T. K.,Tambyah, P. A.,Chen, M.,Wai, T. K.**. Viral Load of SARS-CoV-2 in Respiratory Aerosols Emitted by COVID-19 Patients while Breathing, Talking, and Singing. *Clinical Infectious Diseases.* 2021. 06:06

**Das, D.,Ramachandran, G.**. Risk analysis of different transport vehicles in India during COVID-19 pandemic. *Environmental Research.* 2021. 199 (no pagination):#pages#

**Emecen, A. N.,Basoglu Sensoy, E.,Sezgin, E.,Yildirim Ustuner, B.,Keskin, S.,Siyve, N.,Celik, S. G.,Bayrak, G.,Senturk Durukan, N.,Coskun Beyan, A.,Ergor, A.,Unal, B.,Ergor, G.**. Transmission dynamics and timing of key events for SARS-CoV-2 infection in healthcare workers. *Infectious Diseases.* 2021. 53(7):531-537

**Ghinai, I.,Davis, E. S.,Mayer, S.,Toews, K. A.,Huggett, T. D.,Snow-Hill, N.,Perez, O.,Hayden, M. K.,Tehrani, S.,Landi, A. J.,Crane, S.,Bell, E.,Hermes, J. M.,Desai, K.,Godbee, M.,Jhaveri, N.,Borah, B.,Cable, T.,Sami, S.,Nozicka, L.,Chang, Y. S.,Jagadish, A.,Chee, M.,Thigpen, B.,Llerena, C.,Tran, M.,Surabhi, D. M.,Smith, E. D.,Remus, R. G.,Staszcuk, R.,Figueroa, E.,Leo, P.,Detmer, W. M.,Lyon, E.,Carreon, S.,Hoferka, S.,Ritger, K. A.,Jasmin, W.,Nagireddy, P.,Seo, J. Y.,Fricchione, M. J.,Kerins, J. L.,Black, S. R.,Butler, L. M.,Howard, K.,McCauley, M.,Fraley, T.,Arwady, M. A.,Gretsch, S.,Cunningham, M.,Pacilli, M.,Ruestow, P. S.,Mosites, E.,Avery, E.,Longcoy, J.,Lynch, E. B.,Layden, J. E.**. Risk Factors for Severe Acute Respiratory Syndrome Coronavirus 2 Infection in Homeless Shelters in Chicago, Illinois - March-May, 2020. *Open Forum Infectious Diseases.* 2020. 7(11) (no pagination):#pages#

**Guo, X.,Wang, J.,Hu, D.,Wu, L.,Gu, L.,Wang, Y.,Zhao, J.,Zeng, L.,Zhang, J.,Wu, Y.**. Survey of COVID-19 Disease Among Orthopaedic Surgeons in Wuhan, People's Republic of China. *Journal of Bone & Joint Surgery - American Volume.* 2020. 102:847-854

**Holmes, N.,Virani, S.,Relwani, J.**. Hospital transmission rates of the SARS-CoV 2 disease amongst orthopaedic in-patients in a secondary care centre: A quantitative review. *Journal of Clinical Orthopaedics and Trauma.* 2021. 16:43-48

**Ibiebele, J.,Silkaitis, C.,Dolgin, G.,Bolon, M.,JaneCullen,,Zembower, T.**. Occupational COVID-19 Exposures and Secondary Cases among Healthcare Personnel. *American journal of infection control..* 2021. 07:#pages#

**Jang, S.,Han, S. H.,Rhee, J. Y.**. Cluster of Coronavirus Disease Associated with Fitness Dance Classes, South Korea. *Emerging Infectious Diseases.* 2020. 26:1917-1920

**Klompas, M.,Baker, M. A.,Rhee, C.**. Airborne Transmission of SARS-CoV-2: Theoretical Considerations and Available Evidence. *JAMA - Journal of the American Medical Association.* 2020. 324(5):441-442

**Kondilis, E.,Papamichail, D.,McCann, S.,Carruthers, E.,Veizis, A.,Orcutt, M.,Hargreaves, S.**. The impact of the COVID-19 pandemic on refugees and asylum seekers in Greece: A retrospective analysis of national surveillance data from 2020. *EClinicalMedicine.* 2021. 37:100958

**Leite, A., Leao, T., Soares, P., Severo, M., Moniz, M., Lucas, R., Aguiar, P., Meireles, P., Lunet, N., Nunes, C., Barros, H.**. A Case-Control Study of Contextual Factors for SARS-CoV-2 Transmission. *Frontiers in Public Health.* 2021. 9:772782

**Leung, K. S.,Ng, T. T.,Wu, A. K.,Yau, M. C.,Lao, H. Y.,Choi, M. P.,Tam, K. K.,Lee, L. K.,Wong, B. K.,Man Ho, A. Y.,Yip, K. T.,Lung, K. C.,Liu, R. W.,Tso, E. Y.,Leung, W. S.,Chan, M. C.,Ng, Y. Y.,Sin, K. M.,Fung, K. S.,Chau, S. K.,To, W. K.,Que, T. L.,Shum, D. H.,Yip, S. P.,Yam, W. C.,Siu, G. K.**. Territorywide Study of Early Coronavirus Disease Outbreak, Hong Kong, China. *Emerging Infectious Diseases.* 2021. 27:196-204

**Li, G. G.,Lv, Z.,Wang, Y. S.,Li, J. F.,Feng, L. F.,Wang, M. F.,He, B.,Pan, X. L.**. Retrospective Analysis of 2019-nCov-Infected Cases in Dongyang, Southeastern China. *The Canadian Journal of Infectious Diseases & Medical Microbiology.* 2020. 2020:7056707

**Liu, S.,Yuan, S.,Sun, Y.,Zhang, B.,Wang, H.,Lu, J.,Tan, W.,Liu, X.,Zhang, Q.,Xia, Y.,Lyu, X.,Li, J.,Guo, Y.**. A COVID-19 Outbreak - Nangong City, Hebei Province, China, January 2021. *China CDC Weekly.* 2021. 3:401-404

**Lo Vecchio, A.,Pierri, L.,Poeta, M.,Vassallo, E.,Varelli, M.,Montella, E.,Guarino, A.,Bruzzese, E.**. Risk of SARS-CoV-2 transmission in health care personnel working in a pediatric COVID-19 unit. *Hospital Pediatrics.* 2021. 11(3):E42-E47

**Lyngse, F. P., Molbak, K., Skov, R. L., Christiansen, L. E., Mortensen, L. H., Albertsen, M., Moller, C. H., Krause, T. G., Rasmussen, M., Michaelsen, T. Y., Voldstedlund, M., Fonager, J., Steenhard, N., Danish Covid-19 Genome, Consortium, Kirkeby, C. T.**. Increased transmissibility of SARS-CoV-2 lineage B.1.1.7 by age and viral load. *Nature communications.* 2021. 12:7251

**Mahmood, M.,Ilyas, N. U.,Khan, M. F.,Hasrat, M. N.,Richwagen, N.**. Transmission frequency of COVID-19 through pre-symptomatic and asymptomatic patients in AJK: a report of 201 cases. *Virology Journal.* 2021. 18:138

**Mohiedden, M.,Said, A. M.,Ali, A. M.,Abdel Razik, M. M.,Gad, M. A.**. Healthcare workers infection rate in the era of coronavirus disease 2019-in tertiary teaching hospital. *Open Access Macedonian Journal of Medical Sciences.* 2021. 9(A):651-658

**Moore, G.,Rickard, H.,Stevenson, D.,Aranega-Bou, P.,Pitman, J.,Crook, A.,Davies, K.,Spencer, A.,Burton, C.,Easterbrook, L.,Love, H. E.,Summers, S.,Welch, S. R.,Wand, N.,Thompson, K. A.,Pottage, T.,Richards, K. S.,Dunning, J.,Bennett, A.**. Detection of SARS-CoV-2 within the healthcare environment: a multi-centre study conducted during the first wave of the COVID-19 outbreak in England. *Journal of Hospital Infection.* 2021. 108:189-196

**Moreno, T.,Pinto, R. M.,Bosch, A.,Moreno, N.,Alastuey, A.,Minguillon, M. C.,Anfruns-Estrada, E.,Guix, S.,Fuentes, C.,Buonanno, G.,Stabile, L.,Morawska, L.,Querol, X.**. Tracing surface and airborne SARS-CoV-2 RNA inside public buses and subway trains. *Environment International.* 2021. 147:106326

**Murray, A.,Englund, J.,Wilcox, N.,Heimonen, J.,Emanuels, A.,Chu, H.**. Comorbid Asthma Within a Prospective Household Study of Respiratory Viral Infections. *Journal of Allergy and Clinical Immunology.* 2021. 147(2 Supplement):AB149

**Mutha, A. S.,Beldar, A. S.,Desai, S.,Kumar, N.,Bhartiya, S.,Singh, T.**. Risk factors for reverse transcriptase polymerase chain reaction positivity for SARS-CoV-2 among health care workers in a group of tertiary care hospitals in Mumbai: A cross-sectional study. *Journal of Clinical and Diagnostic Research.* 2021. 15(4):FC18-FC21

**Nyasulu, J. C. Y.,Munthali, R. J.,Nyondo-Mipando, A. L.,Pandya, H.,Nyirenda, L.,Nyasulu, P. S.,Manda, S.**. COVID-19 pandemic in Malawi: Did public sociopolitical events gatherings contribute to its first-wave local transmission?. *International Journal of Infectious Diseases.* 2021. 106:269-275

**Piapan, L.,De Michieli, P.,Ronchese, F.,Rui, F.,Mauro, M.,Peresson, M.,Segat, L.,D'Agaro, P.,Negro, C.,Bovenzi, M.,Larese Filon, F.**. COVID-19 outbreak in healthcare workers in hospitals in Trieste, North-east Italy. *Journal of Hospital Infection.* 2020. 106(3):626-628

**Pisani, M., Anderson, L., Hatas, G., Safdar, N.**. A systems approach to understanding SARS-CoV-2 transmission among healthcare workers in a cluster. *American Journal of Infection Control.* 2021. 06:06

**Rogers, T. M.,Robinson, S. J.,Reynolds, L. E.,Ladva, C. N.,Burgos-Garay, M.,Whiteman, A.,Budge, H.,Soto, N.,Thompson, M.,Hunt, E.,Barson, T.,Boyd, A. T.**. Multifaceted Public Health Response to a COVID-19 Outbreak Among Meat-Processing Workers, Utah, March-June 2020. *Journal of Public Health Management & Practice.* 2021. 01:01

**Schumacher, Y. O.,Tabben, M.,Hassoun, K.,Al Marwani, A.,Al Hussein, I.,Coyle, P.,Abbassi, A. K.,Ballan, H. T.,Al-Kuwari, A.,Chamari, K.,Bahr, R.**. Resuming professional football (soccer) during the COVID-19 pandemic in a country with high infection rates: a prospective cohort study. *British Journal of Sports Medicine.* 2021. 55:1092-1098

**Sherby, M. R., Walsh, T. J., Lai, A. M., Neidich, J. A., Balls-Berry, J. E., Morris, S. M., Head, R., Prener, C. G., Newland, J. G., Gurnett, C. A., Baldenweck, M., Bono, K., Brodsky, V. B., Caburnay, C. A., Constantino, J. N., Dougherty, N. L., Dubois, J. M., Fritz, S. A., Gotto, G. S., Imbeah, A., Kalb, L. G., Liu, J., Maricque, B. B., McKay, V. R., Myers, L. S., Poor, T. J., Powell, B. J., Mueller, N. B., Schlaggar, B. L., Schmidt, A., Snider, E., Traughber, M. C., van Stone, M., Vestal, L., Wilcher-Roberts, M.**. SARS-CoV-2 screening testing in schools for children with intellectual and developmental disabilities. *Journal of Neurodevelopmental Disorders.* 2021. 13(1) (no pagination):#pages#

**Skubacz, K.,Hildebrandt, R.,Zgorska, A.,Dyduch, Z.,Samolej, K.,Smolinski, A.**. Transport of Aerosols in Underground Mine Workings in Terms of SARS-CoV-2 Virus Threat. *Molecules.* 2021. 26:08

**Song, R.,Han, B.,Song, M.,Wang, L.,Conlon, C. P.,Dong, T.,Tian, D.,Zhang, W.,Chen, Z.,Zhang, F.,Shi, M.,Li, X.**. Clinical and epidemiological features of COVID-19 family clusters in Beijing, China. *Journal of Infection.* 2020. 81:e26-e30

**Villanueva, F.,Notario, A.,Cabanas, B.,Martin, P.,Salgado, S.,Gabriel, M. F.**. Assessment of CO<sub>2</sub> and aerosol (PM<sub>2.5</sub>, PM<sub>10</sub>, UFP) concentrations during the reopening of schools in the COVID-19 pandemic: The case of a metropolitan area in Central-Southern Spain. *Environmental Research.* 2021. 197:111092

**Winslow, R. L., Zhou, J., Windle, E. F., Nur, I., Lall, R., Ji, C., Millar, J. E., Dark, P. M., Naisbitt, J., Simonds, A., Dunning, J., Barclay, W., Baillie, J. K., Perkins, G. D., Semple, M. G., McAuley, D. F., Green, C. A.**. SARS-CoV-2 environmental contamination from hospitalised patients with COVID-19 receiving aerosol-generating procedures. *Thorax.* 2021. 04:04

**Yong, S. E. F.,Anderson, D. E.,Wei, W. E.,Pang, J.,Chia, W. N.,Tan, C. W.,Teoh, Y. L.,Rajendram, P.,Toh, Mphs,Poh, C.,Koh, V. T. J.,Lum, J.,Suhaimi, N. M.,Chia, P. Y.,Chen, M. I.,Vasoo, S.,Ong, B.,Leo, Y. S.,Wang, L.,Lee, V. J. M.**. Connecting clusters of COVID-19: an epidemiological and serological investigation. *The Lancet Infectious Diseases.* 2020. 20:809-815

**Caggiano, G.,Triggiano, F.,Apollonio, F.,Diella, G.,Lopuzzo, M.,D'Ambrosio, M.,Fasano, F.,Stefanizzi, P.,Sorrenti, G. T.,Magarelli, P.,Sorrenti, D. P.,Marcotrigiano, V.,De Giglio, O.,Montagna, M. T.**. Sars-cov-2 rna and supermarket surfaces: A real or presumed threat?. *International Journal of Environmental Research and Public Health.* 2021. 18(17) (no pagination):#pages#

**Clouston, S. A. P., Morozova, O., Meliker, J. R.**. A wind speed threshold for increased outdoor transmission of coronavirus: an ecological study. *BMC Infectious Diseases.* 2021. 21:1194

**Conte, M.,Feltracco, M.,Chirizzi, D.,Trabucco, S.,Dinoi, A.,Gregoris, E.,Barbaro, E.,La Bella, G.,Ciccarese, G.,Belosi, F.,La Salandra, G.,Gambaro, A.,Contini, D.**. Airborne concentrations of SARS-CoV-2 in indoor community environments in Italy. *Environmental Science & Pollution Research.* 2021. 01:01

**D'Accolti, M.,Soffritti, I.,Passaro, A.,Zuliani, G.,Antonioli, P.,Mazzacane, S.,Manfredini, R.,Caselli, E.**. SARS-CoV-2 RNA contamination on surfaces of a COVID-19 ward in a hospital of Northern Italy: what risk of transmission?. *European Review for Medical & Pharmacological Sciences.* 2020. 24:9202-9207

**de Rooij, M. M. T.,Hakze-Van der Honing, R. W.,Hulst, M. M.,Harders, F.,Engelsma, M.,van de Hoef, W.,Meliefste, K.,Nieuwenweg, S.,Oude Munnink, B. B.,van Schothorst, I.,Sikkema, R. S.,van der Spek, A. N.,Spierenburg, M.,Spithoven, J.,Bouwstra, R.,Molenaar, R. J.,Koopmans, M.,Stegeman, A.,van der Poel, W. H. M.,Smit, L. A. M.**. Occupational and environmental exposure to SARS-CoV-2 in and around infected mink farms. *Occupational & Environmental Medicine.* 2021. 30:30

**Espinoza, E. P. S.,Cortes, M. F.,Noguera, S. V.,Paula, A. V.,Guimaraes, T.,Boas, L. S. V.,Park, M.,Silva, C. C. D.,Morales, I.,Perdigao Neto, L. V.,Tozetto-Mendoza, T. R.,Boszczowski, I.,Sabino, E. C.,Mendes-Correa, M. C.,Levin, A. S.,Costa, S. F.**. Are mobile phones part of the chain of transmission of SARS-CoV-2 in hospital settings?. *Revista do Instituto de Medicina Tropical de Sao Paulo.* 2021. 63:e74

**Hasan, A.,Nafie, K.,Abbadi, O.**. Histopathology laboratory paperwork as a potential risk of COVID-19 transmission among laboratory personnel. *#journal#.* 2020. #volume#:#pages#

**Hua, M.,Chen, X.,Cheng, L.,Chen, J.**. Should bike-sharing continue operating during the COVID-19 pandemic? Empirical findings from Nanjing, China. *Journal of Transport & Health.* 2021. 23:101264

**Jerry, J.,O'Regan, E.,O'Sullivan, L.,Lynch, M.,Brady, D.**. Do established infection prevention and control measures prevent spread of SARS-CoV-2 to the hospital environment beyond the patient room?. *Journal of Hospital Infection.* 2020. 105(4):589-592

**Kim, U. J.,Lee, S. Y.,Lee, J. Y.,Lee, A.,Kim, S. E.,Choi, O. J.,Lee, J. S.,Kee, S. J.,Jang, H. C.**. Air and Environmental Contamination Caused by COVID-19 Patients: a Multi-Center Study. *Journal of Korean Medical Science.* 2020. 35:e332

**King, K. G.,Delclos, G. L.,Brown, E. L.,Emery, S. T.,Yamal, J. M.,Emery, R. J.**. An assessment of outpatient clinic room ventilation systems and possible relationship to disease transmission. *American Journal of Infection Control.* 2021. 49:808-812

**Lee, S. E.,Lee, D. Y.,Lee, W. G.,Kang, B.,Jang, Y. S.,Ryu, B.,Lee, S.,Bahk, H.,Lee, E.**. Detection of Novel Coronavirus on the Surface of Environmental Materials Contaminated by COVID-19 Patients in the Republic of Korea. *Osong Public Health and Research Perspectives.* 2020. 11(3):128-132

**Mendes, M.,Andrade Oliveira, A.,Pires, O.,Branca, F.,Beirao, M.,Santa-Cruz, A.,Carvalho, A.,Alves, J.**. Sampling Methods and Risk Stratification Regarding Environmental Contamination by SARS-CoV-2. *Acta Medica Portuguesa.* 2021. 20:20

**Minich, J. J.,Ali, F.,Marotz, C.,Belda-Ferre, P.,Chiang, L.,Shaffer, J. P.,Carpenter, C. S.,McDonald, D.,Gilbert, J.,Allard, S. M.,Allen, E. E.,Knight, R.,Sweeney, D. A.,Swafford, A. D.**. Feasibility of using alternative swabs and storage solutions for paired SARS-CoV-2 detection and microbiome analysis in the hospital environment. *Microbiome.* 2021. 9:25
[truncated: 78,095 more chars]
