# Supplementary material for: SARS-CoV-2 transmission risk for common group activities and settings: a living scoping review
Source: Eur J Public Health. 2023 Nov 23;34(1):196–201. doi: 10.1093/eurpub/ckad195 (PMC10843946; doi:10.1093/eurpub/ckad195)
Supplement: ckad195_Supplementary_Data [file ckad195_supplementary_data.zip › ckad195_Supplementary_Data/ejph-2023-07-om-0370-File010.docx]

# Appendix 7. Summary of evidence for transmission risk of COVID-19 and different settings and activities

| **Setting & Activity** | **Risk Level as reported by study author(s)** | **Preventative Measures reported** | **Time Period Studied** |
| --- | --- | --- | --- |
| **Accommodations** | | | |
| Chalet | High (1) | NR | February 2020 |
| Shared housing (e.g., dormitories) | Inconsistent: Unclear (2,3), Low (4), High (5) | Masking, disinfection, accessibility of alcohol pumps, social distancing | July 2020 – May 2021 |
| Shelters/Social housing | Unclear (6) | NR | May - August 2021 |
| **Business** | | | |
| Conference | High (7) | Social distancing | March - April 2020 |
| **Education** | | | |
| Kindergarten | Inconsistent: Unclear (6), Low (8–17) | Hand hygiene, mask wearing (adults) | June 2020 – August 2021 |
| Primary schools | Inconsistent: Unclear (6), Low (8,9,11–31),  Moderate(25) | Face masks; distancing; screening, handwashing, hybrid education, improved ventilation, bubbles | January 2020 – August 2021 |
| Secondary schools | Low (7,9,11–14,16,17,21,23–25,27–30,32) | Face masks; distancing; screening, handwashing, hybrid education, improved ventilation, bubbles | January 2020 - February 2021 |
| University | Inconsistent: Unclear (33),  High (34) | Face masks, social distancing, quarantine | August 2020 – November 2020 |
| **Events and entertainment** | | | |
| ^a^Social events | High (6,35–46) | Social distancing, face masks | April – June 2020 |
| Weddings | High (7,47) | Social distancing, public awareness of epidemic prevention and control | March - April 2020 |
| Hospitality venues | High (7) | Social distancing | March - April 2020 |
| Shopping | Inconsistent: Unclear (39,48),  Low (6),  High (49) | Social distancing, public awareness of epidemic prevention and control | January 2020 – August 2021 |
| **Healthcare** | | | |
| Community healthcare | Low (50) | Face masks | June 2020 |
| Hospitals | Inconsistent: Unclear (38,39,48,51–56),  Low (57–65),  High (66–81),  Moderate (82) | Face masks, hand hygiene, staff training, PPE, restricting visitors, environmental cleansing and disinfection, quarantine, testing, shorter shifts | March 2020 – March 2021 |
| ^b^Specialty care | Inconsistent: Low (83), Unclear (78,84) | Face masks, hand hygiene, PPE, environmental cleansing and disinfection, quarantine | February 2020 – June 2020 |
| Primary care | High (85) | Social distancing, wearing PPE, testing and contact tracing | March 2020 – June 2020 |
| Healthcare workers  (Transmission to household) | High (53,86–89) | Hand hygiene, face masks, physical distancing | March 2020 – June 2020 |
| Healthcare workers (transmission to patients) | Low (65,90) | Face masks and other infection control policies | October 2020 – April 2021 |
| Healthcare workers (transmission from patients) | Inconsistent: High (45,91–99), Unclear (100) | PPE, infection prevention programs | March – May 2020 |
| Residential and long-term care | High (7,42,46,53,65,85,101–105) | Face masks, hand hygiene, restricting visitors, physical distancing, enhanced cleaning | January – September 2020; March – April 2020 |
| **Hospitality** | | | |
| Cruise ships | High (106) | Ship based quarantine, enhanced health measures and access to onshore quarantine and isolation facilities | March 2020 – April 2020 |
| Tour guides | High (107) | NR | January – March 2020 |
| Waiter or bartenders | Low (107) | NR | January – March 2020 |
| Cooks | Low (107) | NR | January – March 2020 |
| Restaurants | Unclear (45,48,108) | Contact tracing and quarantine, and early introduction of social distancing measures | January 2020 – August 2021 |
| **Household** | | | |
| Family home | Inconsistent: Unclear (108–112),  High (48,51,91–93,113–115) | Public awareness of infection and control, hand hygiene, self-isolation | January 2020 – October 2020 |
| **Specialized services** | | | |
| Dental | Low (116) | PPE | May – October 2020 |
| ^C^Personal care services | Medium (45) | Contact tracing and quarantine, and early introduction of social distancing measures | January – June 2020 |
| **Sports and activities** | | | |
| Outdoor sports and activities | Inconsistent: Low (6,117–120),  High (121,122) | Quarantine for infected individuals, face masks, physical distancing, hygiene measures, temperature checks | July 2020 – August 2021 |
| Indoor sports and activities | High (25,123–125) | Masks, social distancing, hand hygiene | September 2020 - December 2020 |
| ^d^Camp | Low (31,126) | Screening, daily temperature checks, masks, hand hygiene, physical distancing, small cohorts, scheduled site cleanings, and staff COVID-19 education and workplace training | March – August 2020 |
| **Transportation** | | | |
| Bus or metro | High (48,127) | Public awareness of epidemic prevention and control, masks | January 2020 – November 2020 |
| Flight travel | Inconsistent: High (48,51,128–131),  Low (132),  Unclear (6) | Post-flight quarantine, contact tracing, distancing, masks | January 2020– August 2021 |
| Car sharing | Inconsistent: High (51,133), Low (6) | Masking | January 2020- August 2021 |
| Other modes of transportation | Inconsistent: Unclear (6,38,52,93,109,125),  Low (42),  High (49,91,134) | Physical distancing, masking, hand hygiene | January 2020 – August 2021 |
| **Other workplaces** | | | |
| Construction labour | High (39,107,135) | NR | January – March 2020 |
| Domestic housekeepers | High (107) | NR | January – March 2020 |
| Drivers (e.g., car, taxi, van) | High (107) | NR | January – March 2020 |
| Drivers (e.g., bus, train) | Low (107) | NR | January – March 2020 |
| Processing plants | Inconsistent: High (136), Unclear (137) | Masking, testing, ventilation, physical barriers, distancing, disinfection | March-October 2020 |
| Personal care workers | Low (107) | NR | January – March 2020 |
| Receptionists | Low (107) | NR | January – March 2020 |
| Salesperson | High (52,107,138,139) | Social distancing, masking | January – May 2020 |
| Religious professionals | High (107) | NR | January – March 2020 |

NR = Not reported

^a^ Social events are defined by the study authors and may include but is not limited to any social activity with one or more individuals such as dating, getting together with a neighbor or friends, banquet, dinner, karaoke, community gatherings, or birthday parties.

^b^Speciality care includes specific settings outside a general hospital setting (e.g., dialysis unit, physical therapy, outpatient care)

^c^ Personal services may include hair salons, beauty parlors, nail salons, spa, etc.

^d^Includes indoor/outdoor and summer camps

# References:

1. Danis K, Epaulard O, Benet T, Gaymard A, Campoy S, Botelho-Nevers E, et al. Cluster of Coronavirus Disease 2019 (COVID-19) in the French Alps, February 2020. Clin Infect Dis. 07 28;71(15):825–32.

2. Vang KE, Krow-Lucal ER, James AE, Cima MJ, Kothari A, Zohoori N, et al. Participation in Fraternity and Sorority Activities and the Spread of COVID-19 Among Residential University Communities - Arkansas, August 21-September 5, 2020. MMWR Morb Mortal Wkly Rep. 2021/01/08 ed. Jan 8;70(1):20–3.

3. Currie DW, Moreno GK, Delahoy MJ, Pray IW, Jovaag A, Braun KM, et al. Interventions to Disrupt Coronavirus Disease Transmission at a University, Wisconsin, USA, August-October 2020. Emerg Infect Dis. Nov;27(11):2776–85.

4. Bjorkman KK, Saldi TK, Lasda E, Bauer LC, Kovarik J, Gonzales PK, et al. Higher viral load drives infrequent SARS-CoV-2 transmission between asymptomatic residence hall roommates. J Infect Dis. Jul 24;24:24.

5. Akaishi T, Kushimoto S, Katori Y, Kure S, Igarashi K, Takayama S, et al. COVID-19 transmission in group living environments and households. Sci. 06 02;11(1):11616.

6. Grant R, Charmet T, Schaeffer L, Galmiche S, Madec Y, Von Platen C, et al. Impact of SARS-CoV-2 Delta variant on incubation, transmission settings and vaccine effectiveness: Results from a nationwide case-control study in France. The Lancet Regional Health - Europe. 2021 Nov;100278.

7. James A, Plank MJ, Hendy S, Binny RN, Lustig A, Steyn N. Model-free estimation of COVID-19 transmission dynamics from a complete outbreak. PLoS ONE. 2021;16(3):e0238800.

8. Meuris C, Kremer C, Geerinck A, Locquet M, Bruyere O, Defeche J, et al. Transmission of SARS-CoV-2 After COVID-19 Screening and Mitigation Measures for Primary School Children Attending School in Liege, Belgium. JAMA netw. 10 01;4(10):e2128757.

9. Bark D, Dhillon N, St-Jean M, Kinniburgh B, McKee G, Choi A. SARS-CoV-2 transmission in kindergarten to grade 12 schools in the Vancouver Coastal Health region: a descriptive epidemiologic study. CMAJ Open. Jul;9(3):E810–7.

10. Loenenbach A, Markus I, Lehfeld AS, An der Heiden M, Haas W, Kiegele M, et al. SARS-CoV-2 variant B.1.1.7 susceptibility and infectiousness of children and adults deduced from investigations of childcare centre outbreaks, Germany, 2021. Euro Surveill. 5;26(21):05.

11. Larosa E, Djuric O, Cassinadri M, Cilloni S, Bisaccia E, Vicentini M, et al. Secondary transmission of COVID-19 in preschool and school settings in northern Italy after their reopening in September 2020: a population-based study. Euro Surveill. 12;25(49):12.

12. Calvani M, Cantiello G, Cavani M, Lacorte E, Mariani B, Panetta V, et al. Reasons for SARS-CoV-2 infection in children and their role in the transmission of infection according to age: a case-control study. Ital. Sep 27;47(1):193.

13. Ismail SA, Saliba V, Lopez Bernal J, Ramsay ME, Ladhani SN. SARS-CoV-2 infection and transmission in educational settings: a prospective, cross-sectional analysis of infection clusters and outbreaks in England. Lancet Infect Dis. 3;21(3):344–53.

14. Gras-Le Guen C, Cohen R, Rozenberg J, Launay E, Levy-Bruhl D, Delacourt C. Reopening schools in the context of increasing COVID-19 community transmission: The French experience. Arch Pediatr. Apr;28(3):178–85.

15. Ulyte A, Radtke T, Abela IA, Haile SR, Berger C, Huber M, et al. Clustering and longitudinal change in SARS-CoV-2 seroprevalence in school children in the canton of Zurich, Switzerland: prospective cohort study of 55 schools. Bmj. 03 17;372:n616.

16. Zimmerman KO, Akinboyo IC, Brookhart MA, Boutzoukas AE, McGann KA, Smith MJ, et al. Incidence and secondary transmission of SARS-CoV-2 infections in schools. Pediatrics [Internet]. Apr;147(4) (no pagination). Available from: https://pediatrics.aappublications.org/content/147/4/e2020048090 https://login.proxy.bib.uottawa.ca/login?url=http://ovidsp.ovid.com/ovidweb.cgi?T=JS&CSC=Y&NEWS=N&PAGE=fulltext&D=emexa&AN=2011883927 https://ocul-uo.primo.exlibrisgroup.com/discovery/openurl?institution=01OCUL_UO&vid=01OCUL_UO:UO_DEFAULT&?sid=OVID:embase&id=pmid:33419869&id=doi:10.1542%2Fpeds.2020-048090&issn=0031-4005&isbn=&volume=147&issue=4&spage=e2020048090&pages=&date=2021&title=Pediatrics&atitle=Incidence+and+secondary+transmission+of+SARS-CoV-2+infections+in+schools&aulast=Zimmerman&pid=%3Cauthor%3EZimmerman+K.O.%3BAkinboyo+I.C.%3BBrookhart+M.A.%3BBoutzoukas+A.E.%3BMcGann+K.A.%3BSmith+M.J.%3BPanayotti+G.M.%3BArmstrong+S.C.%3BBristow+H.%3BParker+D.%3BZadrozny+S.%3BWeber+D.J.%3BBenjamin+D.K.%3C%2Fauthor%3E%3CAN%3E2011883927%3C%2FAN%3E%3CDT%3EArticle%3C%2FDT%3E

17. Zimmerman KO, Brookhart MA, Kalu IC, Boutzoukas AE, McGann KA, Smith MJ, et al. Community SARS-CoV-2 Surge and Within-School Transmission. Pediatrics. Jul 28;28:28.

18. Edward PR, Reyna ME, Daly MK, Hultquist JF, Muller WJ, Ozer EA, et al. Screening Students and Staff for Asymptomatic Coronavirus Disease 2019 in Chicago Schools. Journal of Pediatrics. Dec;239:74-80.e1.

19. Schoeps A, Hoffmann D, Tamm C, Vollmer B, Haag S, Kaffenberger T, et al. Surveillance of SARS-CoV-2 transmission in educational institutions, August to December 2020, Germany. Epidemiology and Infection [Internet]. Sep 22;149 (no pagination). Available from: http://journals.cambridge.org/action/displayJournal?jid=HYGhttps://login.proxy.bib.uottawa.ca/login?url=http://ovidsp.ovid.com/ovidweb.cgi?T=JS&CSC=Y&NEWS=N&PAGE=fulltext&D=emexb&AN=2014809695https://ocul-uo.primo.exlibrisgroup.com/discovery/openurl?institution=01OCUL_UO&vid=01OCUL_UO:UO_DEFAULT&?sid=OVID:embase&id=pmid:34549699&id=doi:10.1017%2FS0950268821002077&issn=0950-2688&isbn=&volume=149&issue=&spage=e213&pages=&date=2021&title=Epidemiology+and+Infection&atitle=Surveillance+of+SARS-CoV-2+transmission+in+educational+institutions%2C+August+to+December+2020%2C+Germany&aulast=Schoeps&pid=%3Cauthor%3ESchoeps+A.%3BHoffmann+D.%3BTamm+C.%3BVollmer+B.%3BHaag+S.%3BKaffenberger+T.%3BFerguson-Beiser+K.%3BKohlhase-Griebel+B.%3BBasenach+S.%3BMissal+A.%3BHofling+K.%3BMichels+H.%3BSchall+A.%3BKappes+H.%3BVogt+M.%3BJahn+K.%3BBarnighausen+T.%3BZanger+P.%3C%2Fauthor%3E%3CAN%3E2014809695%3C%2FAN%3E%3CDT%3EArticle%3C%2FDT%3E

20. Falk A, Benda A, Falk P, Steffen S, Wallace Z, Høeg TB. COVID-19 Cases and Transmission in 17 K-12 Schools - Wood County, Wisconsin, August 31-November 29, 2020. MMWR Morb Mortal Wkly Rep. 2021/01/29 ed. Jan 29;70(4):136–40.

21. Chua GT, Wong JSC, Lam I, Ho PPK, Chan WH, Yau FYS, et al. Clinical Characteristics and Transmission of COVID-19 in Children and Youths During 3 Waves of Outbreaks in Hong Kong. JAMA netw. 05 03;4(5):e218824.

22. Wada K, Okabe N, Shobugawa Y. Infection and transmission of COVID-19 among students and teachers in schools in Japan after the reopening in June 2020. BMJ Paediatrics Open [Internet]. Sep 29;4(1) (no pagination). Available from: http://bmjpaedsopen.bmj.com/ https://login.proxy.bib.uottawa.ca/login?url=http://ovidsp.ovid.com/ovidweb.cgi?T=JS&CSC=Y&NEWS=N&PAGE=fulltext&D=emed21&AN=632980888 https://ocul-uo.primo.exlibrisgroup.com/discovery/openurl?institution=01OCUL_UO&vid=01OCUL_UO:UO_DEFAULT&?sid=OVID:embase&id=pmid:&id=doi:10.1136%2Fbmjpo-2020-000854&issn=2399-9772&isbn=&volume=4&issue=1&spage=e000854&pages=&date=2020&title=BMJ+Paediatrics+Open&atitle=Infection+and+transmission+of+COVID-19+among+students+and+teachers+in+schools+in+Japan+after+the+reopening+in+June+2020&aulast=Wada&pid=%3Cauthor%3EWada+K.%3BOkabe+N.%3BShobugawa+Y.%3C%2Fauthor%3E%3CAN%3E632980888%3C%2FAN%3E%3CDT%3EArticle%3C%2FDT%3E

23. Heavey L, Casey G, Kelly C, Kelly D, McDarby G. No evidence of secondary transmission of COVID-19 from children attending school in Ireland, 2020. Eurosurveillance [Internet]. 2020;25(21). Available from: https://www.eurosurveillance.org/content/10.2807/1560-7917.ES.2020.25.21.2000903 https://login.proxy.bib.uottawa.ca/login?url=http://ovidsp.ovid.com/ovidweb.cgi?T=JS&CSC=Y&NEWS=N&PAGE=fulltext&D=emed21&AN=2006036925 https://ocul-uo.primo.exlibrisgroup.com/discovery/openurl?institution=01OCUL_UO&vid=01OCUL_UO:UO_DEFAULT&?sid=OVID:embase&id=pmid:32489179&id=doi:10.2807%2F1560-7917.ES.2020.25.21.2000903&issn=1025-496X&isbn=&volume=25&issue=21&spage=&pages=&date=2020&title=Eurosurveillance&atitle=No+evidence+of+secondary+transmission+of+COVID-19+from+children+attending+school+in+Ireland%2C+2020&aulast=Heavey&pid=%3Cauthor%3EHeavey+L.%3BCasey+G.%3BKelly+C.%3BKelly+D.%3BMcDarby+G.%3C%2Fauthor%3E%3CAN%3E2006036925%3C%2FAN%3E%3CDT%3EArticle%3C%2FDT%3E

24. Alonso S, Alvarez-Lacalle E, Catala M, Lopez D, Jordan I, Garcia-Garcia JJ, et al. Age-dependency of the Propagation Rate of Coronavirus Disease 2019 Inside School Bubble Groups in Catalonia, Spain. Pediatr Infect Dis J. Jul 27;27:27.

25. Gettings JR, Gold JAW, Kimball A, Forsberg K, Scott C, Uehara A, et al. SARS-CoV-2 transmission in a Georgia school district - United States, December 2020-January 2021. Clin Infect Dis. Apr 17;17:17.

26. Ladhani SN, Baawuah F, Beckmann J, Okike IO, Ahmad S, Garstang J, et al. SARS-CoV-2 infection and transmission in primary schools in England in June-December, 2020 (sKIDs): an active, prospective surveillance study. Lancet Child Adolesc Health. 6;5(6):417–27.

27. Macartney K, Quinn HE, Pillsbury AJ, Koirala A, Deng L, Winkler N, et al. Transmission of SARS-CoV-2 in Australian educational settings: a prospective cohort study. Lancet Child Adolesc Health. 11;4(11):807–16.

28. Mossong J, Mombaerts L, Veiber L, Pastore J, Coroller GL, Schnell M, et al. SARS-CoV-2 transmission in educational settings during an early summer epidemic wave in Luxembourg, 2020. BMC Infect Dis. May;21(1):417.

29. Theuring S, Thielecke M, van Loon W, Hommes F, Hulso C, von der Haar A, et al. SARS-CoV-2 infection and transmission in school settings during the second COVID-19 wave: a cross-sectional study, Berlin, Germany, November 2020. Euro Surveill. 8;26(34):08.

30. Thompson DA, Abbasizanjani H, Fry R, Marchant E, Griffiths L, Akbari A, et al. Staff-pupil SARS-CoV-2 infection pathways in schools in Wales: A population-level linked data approach. BMJ Paediatrics Open [Internet]. May 10;5(1) (no pagination). Available from: http://bmjpaedsopen.bmj.com/ https://login.proxy.bib.uottawa.ca/login?url=http://ovidsp.ovid.com/ovidweb.cgi?T=JS&CSC=Y&NEWS=N&PAGE=fulltext&D=emexb&AN=634995061 https://ocul-uo.primo.exlibrisgroup.com/discovery/openurl?institution=01OCUL_UO&vid=01OCUL_UO:UO_DEFAULT&?sid=OVID:embase&id=pmid:&id=doi:10.1136%2Fbmjpo-2021-001049&issn=2399-9772&isbn=&volume=5&issue=1&spage=e001049&pages=&date=2021&title=BMJ+Paediatrics+Open&atitle=Staff-pupil+SARS-CoV-2+infection+pathways+in+schools+in+Wales%3A+A+population-level+linked+data+approach&aulast=Thompson&pid=%3Cauthor%3EThompson+D.A.%3BAbbasizanjani+H.%3BFry+R.%3BMarchant+E.%3BGriffiths+L.%3BAkbari+A.%3BHollinghurst+J.%3BNorth+L.%3BLyons+J.%3BTorabi+F.%3BDavies+G.%3BGravenor+M.B.%3BLyons+R.A.%3C%2Fauthor%3E%3CAN%3E634995061%3C%2FAN%3E%3CDT%3EArticle%3C%2FDT%3E

31. Jordan I, e Sevilla MF, Fumado V, Bassat Q, Bonet-Carne E, Fortuny C, et al. Transmission of SARS-CoV-2 infection among children in summer schools applying stringent control measures in Barcelona, Spain. Clin Infect Dis. Mar 12;12:12.

32. Kirsten C, Unrath M, Luck C, Dalpke AH, Berner R, Armann J. SARS-CoV-2 seroprevalence in students and teachers: a longitudinal study from May to October 2020 in German secondary schools. BMJ Open. 06 10;11(6):e049876.

33. Valesano AL, Fitzsimmons WJ, Blair CN, Woods RJ, Gilbert J, Rudnik D, et al. SARS-CoV-2 Genomic Surveillance Reveals Little Spread From a Large University Campus to the Surrounding Community. Open forum infect. Nov;8(11):ofab518.

34. Denny TN, Andrews L, Bonsignori M, Cavanaugh K, Datto MB, Deckard A, et al. Implementation of a Pooled Surveillance Testing Program for Asymptomatic SARS-CoV-2 Infections on a College Campus - Duke University, Durham, North Carolina, August 2-October 11, 2020. MMWR Morb Mortal Wkly Rep. Nov 20;69(46):1743–7.

35. Kang CR, Lee JY, Park Y, Huh IS, Ham HJ, Han JK, et al. Coronavirus Disease Exposure and Spread from Nightclubs, South Korea. Emerg Infect Dis. 2020 Oct;26(10):2499–501.

36. Delaugerre C, Foissac F, Abdoul H, Masson G, Choupeaux L, Dufour E, et al. Prevention of SARS-CoV-2 transmission during a large, live, indoor gathering (SPRING): a non-inferiority, randomised, controlled trial. Lancet Infect Dis. Nov 26;26:26.

37. Whaley CM, Cantor J, Pera M, Jena AB. Assessing the association between social gatherings and covid-19 risk using birthdays. JAMA Internal Medicine. Aug;181(8):1090–9.

38. Lentz RJ, Colt H, Chen H, Cordovilla R, Popevic S, Tahura S, et al. Assessing coronavirus disease 2019 (COVID-19) transmission to healthcare personnel: The global ACT-HCP case-control study. Infect Control Hosp Epidemiol. 4;42(4):381–7.

39. Liu Y, Gu Z, Liu J. Uncovering transmission patterns of COVID-19 outbreaks: A region-wide comprehensive retrospective study in Hong Kong. EClinicalMedicine. 2021;100929.

40. Ai J, Shi N, Shi Y, Xu K, Dai Q, Liu W, et al. Epidemiologic characteristics and influencing factors of cluster infection of COVID-19 in Jiangsu Province. Epidemiol Infect. 02 10;149:e48.

41. Nakajo K, Nishiura H. Transmissibility of asymptomatic COVID-19: Data from Japanese clusters. Int J Infect Dis. Apr;105:236–8.

42. Martin-Sanchez M, Lim WW, Yeung A, Adam DC, Ali ST, Lau EHY, et al. COVID-19 transmission in Hong Kong despite universal masking. J Infect. 7;83(1):92–5.

43. Domenech-Montoliu S, Pac-Sa MR, Vidal-Utrillas P, Latorre-Poveda M, Del Rio-Gonzalez A, Ferrando-Rubert S, et al. Mass gathering events and COVID-19 transmission in Borriana (Spain): A retrospective cohort study. PLoS ONE. 2021;16(8):e0256747.

44. Kwok KO, Wei WI, Huang Y, Kam KM, Chan EYY, Riley S, et al. Evolving Epidemiological Characteristics of COVID-19 in Hong Kong From January to August 2020: Retrospective Study. J Med Internet Res. 04 16;23(4):e26645.

45. Wong NS, Lee SS, Kwan TH, Yeoh EK. Settings of virus exposure and their implications in the propagation of transmission networks in a COVID-19 outbreak. Lancet Reg Health West Pac. Nov;4:100052.

46. Brandl M, Selb R, Seidl-Pillmeier S, Marosevic D, Buchholz U, Rehmet S. Mass gathering events and undetected transmission of SARS-CoV-2 in vulnerable populations leading to an outbreak with high case fatality ratio in the district of Tirschenreuth, Germany. Epidemiol Infect. 2020 Oct 13;148:e252.

47. Ravindran AV, Balneaves LG, Faulkner G, Ortiz A, McIntosh D, Morehouse RL, et al. Canadian Network for Mood and Anxiety Treatments (CANMAT) 2016 Clinical Guidelines for the Management of Adults with Major Depressive Disorder: Section 5. Complementary and Alternative Medicine Treatments. Canadian Journal of Psychiatry - Revue Canadienne de Psychiatrie. 9;61(9):576–87.

48. Zhao P, Zhang N, Li Y. A Comparison of Infection Venues of COVID-19 Case Clusters in Northeast China. Int J Environ Res Public Health. 06 03;17(11):03.

49. Susan JH, Sarah B, Robert WA, Colette S, Clare F, Alex Y, et al. Relative contribution of leaving home for work or education, transport, shopping and other activities on risk of acquiring COVID-19 infection outside the household in the second wave of the pandemic in England and Wales. 2021 Dec; Available from: https://medrxiv.org/cgi/content/short/2021.12.08.21267458

50. Lan FY, Filler R, Mathew S, Buley J, Iliaki E, Bruno-Murtha LA, et al. Sociodemographic risk factors for coronavirus disease 2019 (COVID-19) infection among Massachusetts healthcare workers: A retrospective cohort study. Infect Control Hosp Epidemiol. Jan 28;1–6.

51. Burke RM, Balter S, Barnes E, Barry V, Bartlett K, Beer KD, et al. Enhanced contact investigations for nine early travel-related cases of SARS-CoV-2 in the United States. PLoS ONE. 2020;15(9):e0238342.

52. Sierpinski R, Pinkas J, Jankowski M, Juszczyk G, Topor-Madry R, Szumowski L. Occupational risks for SARS-CoV-2 infection: the Polish experience. Int J Occup Med Environ Health. Oct 20;33(6):781–9.

53. Carazo S, Laliberté D, Villeneuve J, Martin R, Deshaies P, Denis G, et al. Characterization and evolution of infection control practices among severe acute respiratory coronavirus virus 2 (SARS-CoV-2)–infected healthcare workers in acute-care hospitals and long-term care facilities in Québec, Canada, Spring 2020. Infection Control & Hospital Epidemiology. :1–9.

54. Walker A, Houwaart T, Finzer P, Ehlkes L, Tyshaieva A, Damagnez M, et al. Characterization of SARS-CoV-2 infection clusters based on integrated genomic surveillance, outbreak analysis and contact tracing in an urban setting. Clin Infect Dis. Jun 28;28:28.

55. Huang PY, Wu TS, Cheng CW, Chen CJ, Huang CG, Tsao KC, et al. A hospital cluster of COVID-19 associated with a SARS-CoV-2 superspreading event. Journal of Microbiology, Immunology and Infection. 2021;

56. Jung J, Lim YJ, Kim EO, Kim SH. Risk of Severe Acute Respiratory Syndrome Coronavirus 2 (SARS-CoV-2) Transmission Among Healthcare Workers Dining in Hospital Staff Cafeterias. J Korean Med Sci. 2021 Dec 22;37(2):e14.

57. Jung J, Lee J, Kim E, Namgung S, Kim Y, Yun M, et al. Frequent Occurrence of SARS-CoV-2 Transmission among Non-close Contacts Exposed to COVID-19 Patients. Journal of Korean medical science. 2021;36(33).

58. Gagneux-Brunon A, Pelissier C, Gagnaire J, Pillet S, Pozzetto B, Botelho-Nevers E, et al. SARS-CoV-2 infection: advocacy for training and social distancing in healthcare settings. Journal of Hospital Infection. 2020 Nov;106(3):610–2.

59. Ng DC, Tan KK, Chin L, Ali MM, Lee ML, Mahmood FM, et al. Clinical and epidemiological characteristics of children with COVID-19 in Negeri Sembilan, Malaysia. Int J Infect Dis. Jul;108:347–52.

60. Landoas A, Cazzorla F, Gallouche M, Larrat S, Nemoz B, Giner C, et al. SARS-CoV-2 nosocomial infection acquired in a French university hospital during the 1st wave of the Covid-19 pandemic, a prospective study. Antimicrob. 08 05;10(1):114.

61. Rasmussen A, Eustache E, Raviola G, Kaiser B, Grelotti DJ, Belkin GS. Development and validation of a Haitian Creole screening instrument for depression. Transcultural Psychiatry. 2015;52(1):33–57.

62. Cattelan AM, Sasset L, Di Meco E, Cocchio S, Barbaro F, Cavinato S, et al. An Integrated Strategy for the Prevention of SARS-CoV-2 Infection in Healthcare Workers: A Prospective Observational Study. Int J Environ Res Public Health. Aug 10;17(16):10.

63. Olmos C, Campana G, Monreal V, Pidal P, Sanchez N, Airola C, et al. SARS-CoV-2 infection in asymptomatic healthcare workers at a clinic in Chile. PLoS ONE. 2021;16(1):e0245913.

64. Dutta U, Sachan A, Premkumar M, Gupta T, Sahoo S, Grover S, et al. Multidimensional dynamic healthcare personnel (HCP)-centric model from a low-income and middle-income country to support and protect COVID-19 warriors: a large prospective cohort study. BMJ Open. 02 22;11(2):e043837.

65. Williams VR, dit Mieusement LM, Tomiczek N, Chan AK, Salt N, Leis JA. Risk of SARS-CoV-2 transmission from universally masked healthcare workers to patients or residents: A prospective cohort study. American Journal of Infection Control. 2021;49(11):1429–31.

66. Dimcheff DE, Schildhouse RJ, Hausman MS, Vincent BM, Markovitz E, Chensue SW, et al. Seroprevalence of severe acute respiratory syndrome coronavirus-2 (SARS-CoV-2) infection among Veterans Affairs healthcare system employees suggests higher risk of infection when exposed to SARS-CoV-2 outside the work environment. Infect Control Hosp Epidemiol. 4;42(4):392–8.

67. Oliveira MS, Lobo RD, Detta FP, Vieira-Junior JM, Castro TLS, Zambelli DB, et al. SARS-Cov-2 seroprevalence and risk factors among health care workers: Estimating the risk of COVID-19 dedicated units. Am J Infect Control. 9AD;49(9):1197–9.

68. Lai X, Wang M, Qin C, Tan L, Ran L, Chen D, et al. Coronavirus Disease 2019 (COVID-2019) Infection among Health Care Workers and Implications for Prevention Measures in a Tertiary Hospital in Wuhan, China. JAMA Network Open [Internet]. May 21;3(5) (no pagination). Available from: https://jamanetwork.com/journals/jamanetworkopen https://login.proxy.bib.uottawa.ca/login?url=http://ovidsp.ovid.com/ovidweb.cgi?T=JS&CSC=Y&NEWS=N&PAGE=fulltext&D=emexa&AN=631825224 https://ocul-uo.primo.exlibrisgroup.com/discovery/openurl?institution=01OCUL_UO&vid=01OCUL_UO:UO_DEFAULT&?sid=OVID:embase&id=pmid:32437575&id=doi:10.1001%2Fjamanetworkopen.2020.9666&issn=2574-3805&isbn=&volume=3&issue=5&spage=e209666&pages=&date=2020&title=JAMA+Network+Open&atitle=Coronavirus+Disease+2019+%28COVID-2019%29+Infection+among+Health+Care+Workers+and+Implications+for+Prevention+Measures+in+a+Tertiary+Hospital+in+Wuhan%2C+China&aulast=Lai&pid=%3Cauthor%3ELai+X.%3BWang+M.%3BQin+C.%3BTan+L.%3BRan+L.%3BChen+D.%3BZhang+H.%3BShang+K.%3BXia+C.%3BWang+S.%3BXu+S.%3BWang+W.%3C%2Fauthor%3E%3CAN%3E631825224%3C%2FAN%3E%3CDT%3EArticle%3C%2FDT%3E

69. Pandrowala A, Shaikh S, Balsekar M, Kirolkar S, Udani S. Characteristics and Transmission Dynamics of COVID-19 in Healthcare Workers in a Pediatric COVID-Care Hospital in Mumbai. Indian Pediatrics. Jun;58(6):568–71.

70. Costa SF, Giavina-Bianchi P, Buss L, Mesquita Peres CH, Rafael MM, Dos Santos LGN, et al. Severe Acute Respiratory Syndrome Coronavirus 2 (SARS-CoV-2) Seroprevalence and Risk Factors Among Oligo/Asymptomatic Healthcare Workers: Estimating the Impact of Community Transmission. Clin Infect Dis. 09 07;73(5):e1214–8.

71. Ran L, Chen X, Wang Y, Wu W, Zhang L, Tan X. Risk Factors of Healthcare Workers With Coronavirus Disease 2019: A Retrospective Cohort Study in a Designated Hospital of Wuhan in China. Clinical Infectious Diseases. 2020 Nov 19;71(16):2218–21.

72. Zabarsky TF, Bhullar D, Silva SY, Mana TSC, Ertle MT, Navas ME, et al. What are the sources of exposure in healthcare personnel with coronavirus disease 2019 infection? American Journal of Infection Control. 2021 Mar;49(3):392–5.

73. Wang Y, Wu W, Cheng Z, Tan X, Yang Z, Zeng X, et al. Super-factors associated with transmission of occupational COVID-19 infection among healthcare staff in Wuhan, China. Journal of Hospital Infection. 2020 Sep;106(1):25–34.

74. Chatterjee P, Anand T, Singh KJ, Rasaily R, Singh R, Das S, et al. Healthcare workers & SARS-CoV-2 infection in India: A case-control investigation in the time of COVID-19. Indian J Med Res. May;151(5):459–67.

75. Aranaz-Andres JM, McGee-Laso A, Galan JC, Canton R, Mira J, On Behalf Of The Team Of Work C. Activities and Perceived Risk of Transmission and Spread of SARS-CoV-2 among Specialists and Residents in a Third Level University Hospital in Spain. Int J Environ Res Public Health. 03 10;18(6):10.

76. Wenlock RD, Tausan M, Stoyle G, Hendron H, Buchanan O, Tait Z, et al. The epidemiology of hospital inpatient exposure to SARS-CoV-2: A cohort study. Infect Prev Pract. Sep;3(3):100173.

77. Mostafa A, Kandil S, El-Sayed MH, Girgis S, Hafez H, Yosef M, et al. Universal COVID-19 screening of 4040 health care workers in a resource-limited setting: an Egyptian pilot model in a university with 12 public hospitals and medical centers. Int J Epidemiol. 03 03;50(1):50–61.

78. Lidstrom AK, Sund F, Albinsson B, Lindback J, Westman G. Work at inpatient care units is associated with an increased risk of SARS-CoV-2 infection; a cross-sectional study of 8679 healthcare workers in Sweden. Ups J Med Sci. Nov;125(4):305–10.

79. Celebi G, Piskin N, Celik Beklevic A, Altunay Y, Salci Keles A, Tuz MA, et al. Specific risk factors for SARS-CoV-2 transmission among health care workers in a university hospital. Am J Infect Control. 10;48(10):1225–30.

80. Maltezou HC, Dedoukou X, Tsonou P, Tseroni M, Raftopoulos V, Pavli A, et al. Hospital factors associated with SARS-CoV-2 infection among healthcare personnel in Greece. J Hosp Infect. Mar;109:40–3.

81. Atsawawaranunt K, Kochakarn T, Kongklieng A, Panwijitkul P, Tragoolpua R, Jaradilokkul K, et al. COVID-19 Transmission among Healthcare Workers at a Quarantine Facility in Thailand: Genomic and Outbreak Investigations. The American Journal of Tropical Medicine and Hygiene. 2021;105(2):421.

82. Sharma S, Mohindra R, Rana K, Suri V, Bhalla A, Biswal M, et al. Assessment of Potential Risk Factors for 2019-Novel Coronavirus (2019-nCov) Infection among Health Care Workers in a Tertiary Care Hospital, North India. J. Jan;12:21501327211002100.

83. Thadhani R, Willetts J, Wang C, Larkin J, Zhang H, Fuentes LR, et al. Transmission of SARS-CoV-2 considering shared chairs in outpatient dialysis: a real-world case-control study. BMC Nephrol. 09 16;22(1):313.

84. Gianola S, Bargeri S, Campanini I, Corbetta D, Gambazza S, Innocenti T, et al. The Spread of COVID-19 Among 15,000 Physical Therapists in Italy: A Cross-Sectional Study. Phys Ther. 08 01;101(8):01.

85. Froberg M, Hassan SS, Pimenoff VN, Akterin S, Conneryd Lundgren K, Elfstrom KM, et al. Risk for SARS-CoV-2 infection in healthcare workers outside hospitals: A real-life immuno-virological study during the first wave of the COVID-19 epidemic. PLoS ONE. 2021;16(9):e0257854.

86. Wong LY, Tan AL, Leo YS, Lee VJM, Toh MPHS. Healthcare workers in Singapore infected with COVID-19: 23 January-17 April 2020. Influenza and other Respiratory Viruses. Mar;15(2):218–26.

87. Barry M, Robert AA, Temsah MH, Abdul Bari S, Akhtar MY, Al Nahdi F, et al. COVID-19 Community Transmission among Healthcare Workers at a Tertiary Care Cardiac Center. Med Sci (Basel). 06 30;9(3):30.

88. Mansoor S, Sharma KA, Ranjan P, Singhal S, Meena J, Kumari R, et al. A descriptive audit of healthcare workers exposed to COVID-19 at a tertiary care center in India. Int J Gynaecol Obstet. Jun;153(3):393–7.

89. Mendez-Echevarria A, Sainz T, e Felipe B, Alcolea S, Olbrich P, Goycochea-Valdivia WA, et al. High Rates of SARS-CoV-2 Family Transmission in Children of Healthcare Workers During the First Pandemic Wave in Madrid, Spain: Serologic Study. Pediatr Infect Dis J. 05 01;40(5):e185–8.

90. Baker MA, Fiumara K, Rhee C, Williams SA, Tucker R, Wickner P, et al. Low risk of COVID-19 among patients exposed to infected healthcare workers. Clinical infectious diseases : an official publication of the Infectious Diseases Society of America [Internet]. 2020;28. Available from: https://login.proxy.bib.uottawa.ca/login?url=http://ovidsp.ovid.com/ovidweb.cgi?T=JS&CSC=Y&NEWS=N&PAGE=fulltext&D=emed21&AN=632721153 https://ocul-uo.primo.exlibrisgroup.com/discovery/openurl?institution=01OCUL_UO&vid=01OCUL_UO:UO_DEFAULT&?sid=OVID:embase&id=pmid:32856692&id=doi:10.1093%2Fcid%2Fciaa1269&issn=1537-6591&isbn=&volume=&issue=&spage=&pages=&date=2020&title=Clinical+infectious+diseases+%3A+an+official+publication+of+the+Infectious+Diseases+Society+of+America&atitle=Low+risk+of+COVID-19+among+patients+exposed+to+infected+healthcare+workers&aulast=Baker&pid=%3Cauthor%3EBaker+M.A.%3BFiumara+K.%3BRhee+C.%3BWilliams+S.A.%3BTucker+R.%3BWickner+P.%3BResnick+A.%3BKlompas+M.%3C%2Fauthor%3E%3CAN%3E632721153%3C%2FAN%3E%3CDT%3EArticle%3C%2FDT%3E

91. Squeri R, Levita A, Intelisano R, Costa GB, Mancuso G, Grasso L, et al. Correct management and low rate of contagiousness of healthcare workers in a University Hospital in Southern Italy: from contact tracing to serological investigation. Acta Biomed Ateneo Parmense. 07 20;91(9-S):79–86.

92. Pinarlik F, Genc Z, Kapmaz M, Tekin S, Ergonul O. Risk groups for SARS-CoV-2 infection among healthcare workers: Community versus hospital transmission. Infectious Disease Reports. 2021;13(3):724–9.

93. Nygren D, Noren J, De Marinis Y, Holmberg A, Fraenkel CJ, Rasmussen M. Association between SARS-CoV-2 and exposure risks in health care workers and university employees-a cross-sectional study. Infectious Diseases. 2021;53(6):460–8.

94. Bahrs C, Kimmig A, Weis S, Ankert J, Hagel S, Maschmann J, et al. Prospective surveillance study in a 1,400-bed university hospital: COVID-19 exposure at home was the main risk factor for SARS-CoV-2 point seroprevalence among hospital staff. Transboundary and emerging diseases. 2022;69(2):720–30.

95. Heinzerling A, Stuckey MJ, Scheuer T, Xu K, Perkins KM, Resseger H, et al. Transmission of COVID-19 to Health Care Personnel During Exposures to a Hospitalized Patient - Solano County, California, February 2020. MMWR Morb Mortal Wkly Rep. Apr 17;69(15):472–6.

96. Contejean A, Leporrier J, Canoui E, Alby-Laurent F, Lafont E, Beaudeau L, et al. Comparing Dynamics and Determinants of Severe Acute Respiratory Syndrome Coronavirus 2 Transmissions among Healthcare Workers of Adult and Pediatric Settings in Central Paris. Clinical Infectious Diseases. Jan 15;72(2):257–64.

97. Aydin M, Altunal LN, Özel AS. Transmission and Clinical Characteristic of COVID-19 in Healthcare Workers: Cross-sectional Study. Türkiye Klinikleri Tıp Bilimleri Dergisi. 2021;41(3):219–24.

98. Wang X, Zhou Q, He Y, Liu L, Ma X, Wei X, et al. Nosocomial outbreak of COVID-19 pneumonia in Wuhan, China. Eur Respir J. 2020 Jun;55(6):2000544.

99. Su WL, Tzeng IS, Yang MC, Lin SJ, Wu PS, Chao YC. Masks prevent hospital-acquired COVID-19: A single hospital experience in Taiwan. Journal of Internal Medicine of Taiwan. Feb;32(1):32–9.

100. Leeman DS, Ma TSG, Pathiraja MM, Taylor JA, Adnan TZ, Baltas I, et al. Severe acute respiratory coronavirus virus 2 (SARS-CoV-2) nosocomial transmission dynamics, a retrospective cohort study of two healthcare-associated coronavirus disease 2019 (COVID-19) clusters in a district hospital in England during March and April 2020. Infect Control Hosp Epidemiol. 2021 Nov 22;1–7.

101. Kevin AB, Aaron J, Nick D, Adrienne KC, Kevin LS, Gary EG, et al. Association Between Nursing Home Crowding and COVID-19 Infection and Mortality in Ontario, Canada. medRxiv [Internet]. 2020; Available from: http://www.epistemonikos.org/documents/efae1e711cf6c5a360e619410f40602352898231

102. Kain D, Stall N, Brown K, McCreight L, Rea E, Kamal M, et al. A Longitudinal, Clinical, and Spatial Epidemiologic Analysis of a Large COVID-19 Long-Term Care Home Outbreak. Journal of the American Medical Directors Association. 2021 Oct;22(10):2003-2008.e2.

103. Fisman DN, Bogoch I, Lapointe-Shaw L, McCready J, Tuite AR. Risk Factors Associated With Mortality Among Residents With Coronavirus Disease 2019 (COVID-19) in Long-term Care Facilities in Ontario, Canada. JAMA Netw Open. 2020 Jul 22;3(7):e2015957.

104. Tang S, Sanchez Perez M, Saavedra-Campos M, Paranthaman K, Myers R, Fok J, et al. Mass testing after a single suspected or confirmed case of COVID-19 in London care homes, April-May 2020: implications for policy and practice. Age Ageing. 05 05;50(3):649–56.

105. MacCannell T, Batson J, Bonin B, Kc A, Quenelle R, Strong B, et al. Genomic epidemiology and transmission dynamics of SARS-CoV-2 in congregate healthcare facilities in Santa Clara County, California. Clin Infect Dis. Jul 30;30:30.

106. Walker LJ, Codreanu TA, Armstrong PK, Goodwin S, Trewin A, Spencer E, et al. SARS-CoV-2 infections among Australian passengers on the Diamond Princess cruise ship: A retrospective cohort study. PLoS ONE [Internet]. Sep;16(9 September) (no pagination). Available from: https://journals.plos.org/plosone/article/file?id=10.1371/journal.pone.0255401&type=printable https://login.proxy.bib.uottawa.ca/login?url=http://ovidsp.ovid.com/ovidweb.cgi?T=JS&CSC=Y&NEWS=N&PAGE=fulltext&D=emexb&AN=2014484567 https://ocul-uo.primo.exlibrisgroup.com/discovery/openurl?institution=01OCUL_UO&vid=01OCUL_UO:UO_DEFAULT&?sid=OVID:embase&id=pmid:&id=doi:10.1371%2Fjournal.pone.0255401&issn=1932-6203&isbn=&volume=16&issue=9+September&spage=e0255401&pages=&date=2021&title=PLoS+ONE&atitle=SARS-CoV-2+infections+among+Australian+passengers+on+the+Diamond+Princess+cruise+ship%3A+A+retrospective+cohort+study&aulast=Walker&pid=%3Cauthor%3EWalker+L.J.%3BCodreanu+T.A.%3BArmstrong+P.K.%3BGoodwin+S.%3BTrewin+A.%3BSpencer+E.%3BColquhoun+S.M.%3BStephens+D.M.%3BBaird+R.W.%3BDouglas+N.M.%3BCribb+D.%3BOwen+R.%3BKelly+P.%3BKirk+M.D.%3C%2Fauthor%3E%3CAN%3E2014484567%3C%2FAN%3E%3CDT%3EArticle%3C%2FDT%3E

107. Lan FY, Wei CF, Hsu YT, Christiani DC, Kales SN. Work-related COVID-19 transmission in six Asian countries/areas: A follow-up study. PLoS ONE. 2020;15(5):e0233588.

108. Ogata T, Ujiie S, Ogawa H, Nagata M, Seki A, Tanaka H. Settings of coronavirus disease 2019 transmission during community outbreak in Tsuchiura, Japan, November 2020. [Nihon koshu eisei zasshi] Japanese journal of public health. Oct;68(10):677–81.

109. Meyer CN. Transmission, start of symptom and morbidity among Danish COVID-19 patients admitted to hospital. Dan Med J. Aug;67(9):06.

110. xiaoke X, Xiaofan L, Lin W, Sheikh Taslim ALI, Zhanwei D, Paolo B, et al. Close contacts and household transmission of SARS-CoV-2 in China: a content analysis based on local Heath Commissions’ public disclosures. 2020 Mar; Available from: https://medrxiv.org/cgi/content/short/2020.03.02.20029868

111. Le TQM, Takemura T, Moi ML, Nabeshima T, Nguyen LKH, Hoang VMP, et al. Severe Acute Respiratory Syndrome Coronavirus 2 Shedding by Travelers, Vietnam, 2020. Emerg Infect Dis. 7AD;26(7):1624–6.

112. Afonso ET, Marques SM, Costa LDC, Fortes PM, Peixoto F, Bichuetti‐Silva DC, et al. Secondary household transmission of SARS‐CoV‐2 among children and adolescents: Clinical and epidemiological aspects. Pediatric Pulmonology. 2022 Jan;57(1):162–75.

113. Schepers M, Zanger P, Jahn K, Konig J, Strauch K, Gianicolo E. Multi-household social gatherings contribute to the second SARS-CoV-2 wave in Rhineland-Palatinate, Germany, August to November 2020. J Infect. Jan 23;23:23.

114. Choi A, Masse LC, Bardwell S, Zhao Y, Xu YXZ, Markarian A, et al. Symptomatic and asymptomatic transmission of SARS-CoV-2 in K-12 schools, British Columbia, April to June 2021. medRxiv [Internet]. 2021;20. Available from: https://www.medrxiv.org/ https://login.proxy.bib.uottawa.ca/login?url=http://ovidsp.ovid.com/ovidweb.cgi?T=JS&CSC=Y&NEWS=N&PAGE=fulltext&D=empp&AN=2015874057 https://ocul-uo.primo.exlibrisgroup.com/discovery/openurl?institution=01OCUL_UO&vid=01OCUL_UO:UO_DEFAULT&?sid=OVID:embase&id=pmid:&id=doi:10.1101%2F2021.11.15.21266284&issn=0166-3542&isbn=&volume=&issue=&spage=&pages=&date=2021&title=medRxiv&atitle=Symptomatic+and+asymptomatic+transmission+of+SARS-CoV-2+in+K-12+schools%2C+British+Columbia%2C+April+to+June+2021&aulast=Choi&pid=%3Cauthor%3EChoi+A.%3BMasse+L.C.%3BBardwell+S.%3BZhao+Y.%3BXu+Y.X.Z.%3BMarkarian+A.%3BCoombs+D.%3BWatts+A.%3BMacdonald+A.%3BDhillon+N.%3BIrvine+M.%3BO%27Reilly+C.%3BLavoie+P.M.%3BGoldfarb+D.%3C%2Fauthor%3E%3CAN%3E2015874057%3C%2FAN%3E%3CDT%3EPreprint%3C%2FDT%3E

115. Zhang H, Hong C, Zheng Q, Zhou P, Zhu Y, Zhang Z, et al. A multi-family cluster of COVID-19 associated with asymptomatic and pre-symptomatic transmission in Jixi City, Heilongjiang, China, 2020. Emerg. Dec;9(1):2509–14.

116. Natapov L, Schwartz D, Herman HD, Markovich DD, Yellon D, Jarallah M, et al. Risk of SARS-CoV-2 transmission following exposure during dental treatment - A national cohort study. J Dent. Aug 26;113:103791.

117. Schreiber S, Faude O, Gartner B, Meyer T, Egger F. Risk of SARS-CoV-2 transmission from on-field player contacts in amateur, youth and professional football (soccer). BJSM online. Oct 18;18:18.

118. Yorck Olaf S, Montassar T, Khalid H, Asmaa Al M, Ibrahim Al H, Peter C, et al. Resuming professional football during the Covid-19 pandemic in a country with high infection ratesA prospective cohort study. 2020 Nov; Available from: https://medrxiv.org/cgi/content/short/2020.11.17.20233023

119. Ben J, Gemma CP, Simon PTK, Brendan P, Brian H, Matthew C, et al. SARS-CoV-2 transmission during team-sport: Do players develop COVID-19 after participating in rugby league matches with SARS-CoV-2 positive players? 2020 Nov; Available from: https://medrxiv.org/cgi/content/short/2020.11.03.20225284

120. Robinson PG, Murray A, Close G, Kinane DF. Assessing the risk of SARS-CoV-2 transmission in international professional golf. BMJ Open Sport Exerc Med. 2021;7(2):e001109.

121. Drogosz M, Pellegren J, Creegan E, Vedachalam V, Quilliam DN, Cooper T, et al. Implications of Sports on COVID-19 cases in Rhode Island School-aged Athletes. R I Med. 06 01;104(5):51–4.

122. Drezner JA, Drezner SM, Magner KN, Ayala JT. COVID-19 Surveillance in Youth Soccer During Small Group Training: A Safe Return to Sports Activity. Sports health. Jan;13(1):15–7.

123. Paul ML. Estimating the Burden of COVID-19 Symptoms Among Participants at the 2020 USA Curling Club Nationals Tournament. 2020 Oct; Available from: https://medrxiv.org/cgi/content/short/2020.10.08.20209437

124. Pauser J, Schwarz C, Morgan J, Jantsch J, Brem M. SARS-CoV-2 transmission during an indoor professional sporting event. Scientific reports. Oct 20;11(1):20723.

125. Munch PK, Espenhain L, Hansen CH, Muller L, Krause TG, Ethelberg S. Societal activities associated with SARS-CoV-2 infection - A case-control study in Denmark, November 2020. Epidemiology and Infection [Internet]. 2021; Available from: http://journals.cambridge.org/action/displayJournal?jid=HYG https://login.proxy.bib.uottawa.ca/login?url=http://ovidsp.ovid.com/ovidweb.cgi?T=JS&CSC=Y&NEWS=N&PAGE=fulltext&D=emexa&AN=2015713118 https://ocul-uo.primo.exlibrisgroup.com/discovery/openurl?institution=01OCUL_UO&vid=01OCUL_UO:UO_DEFAULT&?sid=OVID:embase&id=pmid:&id=doi:10.1017%2FS0950268821002478&issn=0950-2688&isbn=&volume=&issue=&spage=&pages=&date=2021&title=Epidemiology+and+Infection&atitle=Societal+activities+associated+with+SARS-CoV-2+infection+-+A+case-control+study+in+Denmark%2C+November+2020&aulast=Munch&pid=%3Cauthor%3EMunch+P.K.%3BEspenhain+L.%3BHansen+C.H.%3BMuller+L.%3BKrause+T.G.%3BEthelberg+S.%3C%2Fauthor%3E%3CAN%3E2015713118%3C%2FAN%3E%3CDT%3EArticle%3C%2FDT%3E

126. D’Agostino EM, Armstrong SC, Humphreys L, Coffman S, Sinclair G, Permar SR, et al. Symptomatic SARS-CoV-2 Transmission in Youth and Staff Attending Day Camps. Pediatrics. 4;147(4):04.

127. Ramirez DWE, Klinkhammer MD, Rowland LC. COVID-19 Transmission during Transportation of 1st to 12th Grade Students: Experience of an Independent School in Virginia. J Sch Health. 2021/07/22 ed. Sep;91(9):678–82.

128. Toyokawa T, Shimada T, Hayamizu T, Sekizuka T, Zukeyama Y, Yasuda M, et al. Transmission of SARS‐CoV‐2 during a 2‐h domestic flight to Okinawa, Japan, March 2020. Influenza Resp Viruses. 2022 Jan;16(1):63–71.

129. Lunney M, Ronksley PE, Weaver RG, Barnieh L, Blue N, Avey MT, et al. COVID-19 infection among international travellers: A prospective analysis. BMJ Open [Internet]. Jun 24;11(6) (no pagination). Available from: http://bmjopen.bmj.com/content/early/by/section https://login.proxy.bib.uottawa.ca/login?url=http://ovidsp.ovid.com/ovidweb.cgi?T=JS&CSC=Y&NEWS=N&PAGE=fulltext&D=emexb&AN=635357627 https://ocul-uo.primo.exlibrisgroup.com/discovery/openurl?institution=01OCUL_UO&vid=01OCUL_UO:UO_DEFAULT&?sid=OVID:embase&id=pmid:34168036&id=doi:10.1136%2Fbmjopen-2021-050667&issn=2044-6055&isbn=&volume=11&issue=6&spage=e050667&pages=&date=2021&title=BMJ+Open&atitle=COVID-19+infection+among+international+travellers%3A+A+prospective+analysis&aulast=Lunney&pid=%3Cauthor%3ELunney+M.%3BRonksley+P.E.%3BWeaver+R.G.%3BBarnieh+L.%3BBlue+N.%3BAvey+M.T.%3BRolland-Harris+E.%3BKhan+F.M.%3BPang+J.X.Q.%3BRafferty+E.%3BScory+T.D.%3BSvenson+L.W.%3BRodin+R.%3BTonelli+M.%3C%2Fauthor%3E%3CAN%3E635357627%3C%2FAN%3E%3CDT%3EReview%3C%2FDT%3E

130. Hu M, Wang J, Lin H, Ruktanonchai CW, Xu C, Meng B, et al. Risk of SARS-CoV-2 Transmission among Air Passengers in China. Clin Infect Dis. Sep 21;21:21.

131. Guner AE, Surmeli A, Kural K, Sahin E, Alkan P, Kocayigit E, et al. First known COVID-19 case and contact tracing efforts in Istanbul, Turkey. Turk. 08 30;51(4):1653–8.

132. Blomquist PB, Bolt H, Packer S, Schaefer U, Platt S, Dabrera G, et al. Risk of symptomatic COVID-19 due to aircraft transmission: a retrospective cohort study of contact-traced flights during England’s containment phase. Influenza other respi. 5;15(3):336–44.

133. Ng OT, Marimuthu K, Koh V, Pang J, Linn KZ, Sun J, et al. SARS-CoV-2 seroprevalence and transmission risk factors among high-risk close contacts: a retrospective cohort study. The Lancet Infectious Diseases. 2021 Mar 1;21(3):333–43.

134. Zhong P, Guo S, Chen T. Correlation between travellers departing from Wuhan before the Spring Festival and subsequent spread of COVID-19 to all provinces in China. Journal of Travel Medicine. 2020 May 18;27(3):taaa036.

135. Sundar V, Bhaskar E. Low secondary transmission rates of SARS-CoV-2 infection among contacts of construction laborers at open air environment. Germs. Mar;11(1):128–31.

136. Porter KA, Ramaswamy M, Koloski T, Castrodale L, McLaughlin J. COVID-19 Among Workers in the Seafood Processing Industry: Implications for Prevention Measures - Alaska, March-October 2020. MMWR Morb Mortal Wkly Rep. Apr 30;70(17):622–6.

137. Pokora R, Kutschbach S, Weigl M, Braun D, Epple A, Lorenz E, et al. Investigation of superspreading COVID-19 outbreak events in meat and poultry processing plants in Germany: A cross-sectional study. PLoS ONE. 2021;16(6):e0242456.

138. Fan-Yun L, Chih-Fu W, Yu-Tien H, David CC, Stefanos NK. Work-related Covid-19 transmission. 2020 Apr; Available from: https://medrxiv.org/cgi/content/short/2020.04.08.20058297

139. Lan FY, Suharlim C, Kales SN, Yang J. Association between SARS-CoV-2 infection, exposure risk and mental health among a cohort of essential retail workers in the USA. Occup Environ Med. 4;78(4):237–43.
